# Maturation-associated gene expression profiles during normal human bone marrow erythropoiesis

**DOI:** 10.1038/s41420-019-0151-0

**Published:** 2019-02-28

**Authors:** Fabiana V. Mello, Marcelo G. P. Land, Elaine. S. Costa, Cristina Teodósio, María-Luz Sanchez, Paloma Bárcena, Rodrigo T. Peres, Carlos E. Pedreira, Liliane R. Alves, Alberto Orfao

**Affiliations:** 1 Clinical Medicine Postgraduate Programme, College of Medicine, Federal University of Rio de Janeiro (UFRJ), Rio de Janeiro, Brazil; 2 Cytometry Service, Institute of Paediatrics and Puericulture Martagão Gesteira (IPPMG), Federal University of Rio de Janeiro (UFRJ), Rio de Janeiro, Brazil; 3 Department of Immunohematology and Blood Transfusion, Leiden University Medical Centre, Leiden, The Netherlands; 4 Department of Medicine and Cytometry Service (Nucleus), Cancer Research Centre (IBMCC, USAL-CSIC) and CIBERONC, Institute for Biomedical Research of Salamanca (IBSAL), University of Salamanca (USAL), Salamanca, Spain; 5 Department of Mathematics, CEFET-RJ, Rio de Janeiro, Brazil; 6 Systems & Computing Department (PESC), COPPE - Engineering Graduate Program, Federal University of Rio de Janeiro (UFRJ), Rio de Janeiro, Brazil; 7 Pharmacy Service, National Institute of Cancer - INCa, Rio de Janeiro, Brazil

## Abstract

Erythropoiesis has been extensively studied using in vitro and in vivo animal models. Despite this, there is still limited data about the gene expression profiles (GEP) of primary (ex vivo) normal human bone marrow (BM) erythroid maturation. We investigated the GEP of nucleated red blood cell (NRBC) precursors during normal human BM erythropoiesis. Three maturation-associated populations of NRBC were identified and purified from (fresh) normal human BM by flow cytometry and the GEP of each purified cell population directly analyzed using DNA-oligonucleotide microarrays. Overall, 6569 genes (19% of the genes investigated) were expressed in ≥1 stage of BM erythropoiesis at stable (e.g., genes involved in DNA process, cell signaling, protein organization and hemoglobin production) or variable amounts (e.g., genes related to cell differentiation, apoptosis, metabolism), the latter showing a tendency to either decrease from stage 1 to 3 (genes associated with regulation of erythroid differentiation and survival, e.g., *SPI1*, *STAT5A*) or increase from stage 2 to stage 3 (genes associated with autophagy, erythroid functions such as heme production, e.g., *ALAS1*, ALAS2), iron metabolism (e.g., *ISCA1, SLC11A2*), protection from oxidative stress (e.g., *UCP2*, *PARK7*), and NRBC enucleation (e.g., *ID2*, *RB1*). Interestingly, genes involved in apoptosis (e.g., *CASP8, P2RX1*) and immune response (e.g., *FOXO3, TRAF6*) were also upregulated in the last stage (stage 3) of maturation of NRBC precursors. Our results confirm and extend on previous observations and providing a frame of reference for better understanding the critical steps of human erythroid maturation and its potential alteration in patients with different clonal and non-clonal erythropoietic disorders.

## Introduction

Erythrocytes are currently recognized as bone marrow (BM)-derived blood cells specialized in producing hemoglobin for the transportation of oxygen throughout the body, via peripheral blood (PB). After birth, human erythropoiesis occurs in specialized hematopoietic stem cell (HSC) niches enriched in mesenchymal cells and other stromal cells in the BM^1^. Following commitment into the erythroid lineage, progressive expansion of the early erythroid committed progenitors occurs, leading to the generation of burst-forming unit-erythroid progenitors (BFU-E) and colony-forming unit-erythroid precursors (CFU-E)^2^. Subsequently, erythroid precursors sequentially differentiate into morphologically recognizable (nucleated) erythroblasts, and both non-nucleated reticulocytes and erythrocytes, which are released into PB^2^. During maturation of erythroblasts, progressive decrease in cell size with concomitant nuclear condensation and pyknosis, associated with a decrease in the RNA contents occurs, prior to final extrusion of the remaining nucleus^3,4^ required to generate mature (non-nucleated) reticulocytes and erythrocytes^2^.

At present, it is well-known that maturation of early hematopoietic precursors into fully mature erythrocytes involves complex signaling networks (associated with, e.g., cell–cell interactions and cytokine and hormone signaling), that lead to intracellular production of hemoglobin and other erythroid cell components^5^. Early studies on erythroid differentiation have elucidated the composition of the erythroid cell membrane and the key elements of the red cell metabolism^6^. Subsequently, several other in vitro and in vivo animal model studies have delineated the different steps of erythroid maturation both in physiological and in pathological conditions^7^. More recent studies have investigated and identified specific genes, cell signaling pathways and proteins that play a critical role during erythropoiesis, particularly those that are (directly or indirectly) related to the production of heme and hemoglobin^8,9^. Despite all the above, there is still limited data about the changes on the ex vivo gene expression profiles (GEP) of normal human BM erythroid precursors at distinct stages of maturation, and their correlation with the in vitro and in vivo animal model findings^10^.

In the present study, we investigated the GEP of purified nucleated erythroid precursors at three different stages of maturation, directly derived from primary (fresh) normal human BM, in the absence of further in vitro culture. Our results provide a frame of reference for better understanding the critical steps of in vivo erythroid maturation, and the potential identification of aberrant GEP in patients with different (clonal and non-clonal) red cell disorders.

## Results

### Identification of erythroid precursors and their different maturation stages in normal human BM

**Fig. 1:**
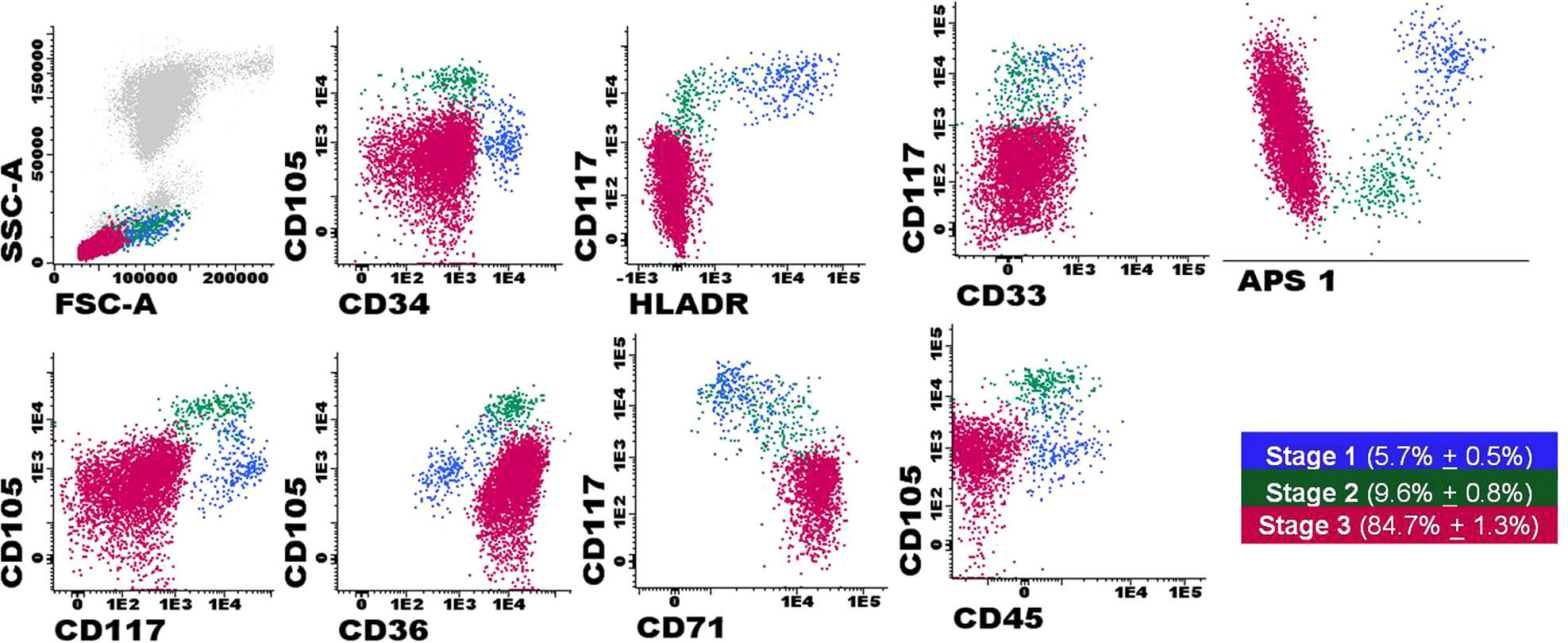
Immunophenotypic profile of normal human BM NRBC at distinct stages of maturation. Stage 1 NRBC precursors were identified as being CD34^+^ HLADR^+^ CD117^low^ CD36^low^ CD45^low^ CD105^+^ CD33^–^ CD71^low^ (blue dots); intermediate stage 2 NRBC were CD34^−^ HLADR^−^ CD117^−^ CD36^+^ CD45^−^ CD105^high^ CD33^–^ CD71^+^ (green dots); and the more mature stage 3 NRBC precursors were defined as CD34^−^ HLADR^−^ CD117^−^ CD36^high^ CD45^−^ CD105^+/−^ CD33^–^ CD71^high^ cells (pink dots)

Overall, three different stages of maturation of NRBC were defined and identified in all BM samples analyzed (Fig. 1), and nucleated cells included from each of such maturation stages, were subsequently FACS-purified. Thus, the earliest stage of erythroid maturation corresponded to BM CD34^+^ precursors that showed progressively greater expression levels of CD71 and CD105 (stage 1). Subsequently, (stage 2) erythroid precursors showed strong CD71 and co-expression of CD105 and CD36, while they had already downregulated CD34, CD45, CD117, and HLADR expression to undetectable levels (Fig. 1). Further, maturation of erythroid precursors (toward stage 3) was associated with downregulation of CD105, while strong positivity for both CD71 and CD36 was retained (Fig. 1). NRBC precursors from all three stages of maturation were systematically negative for CD33 (Fig. 1).

**Fig. 2:**
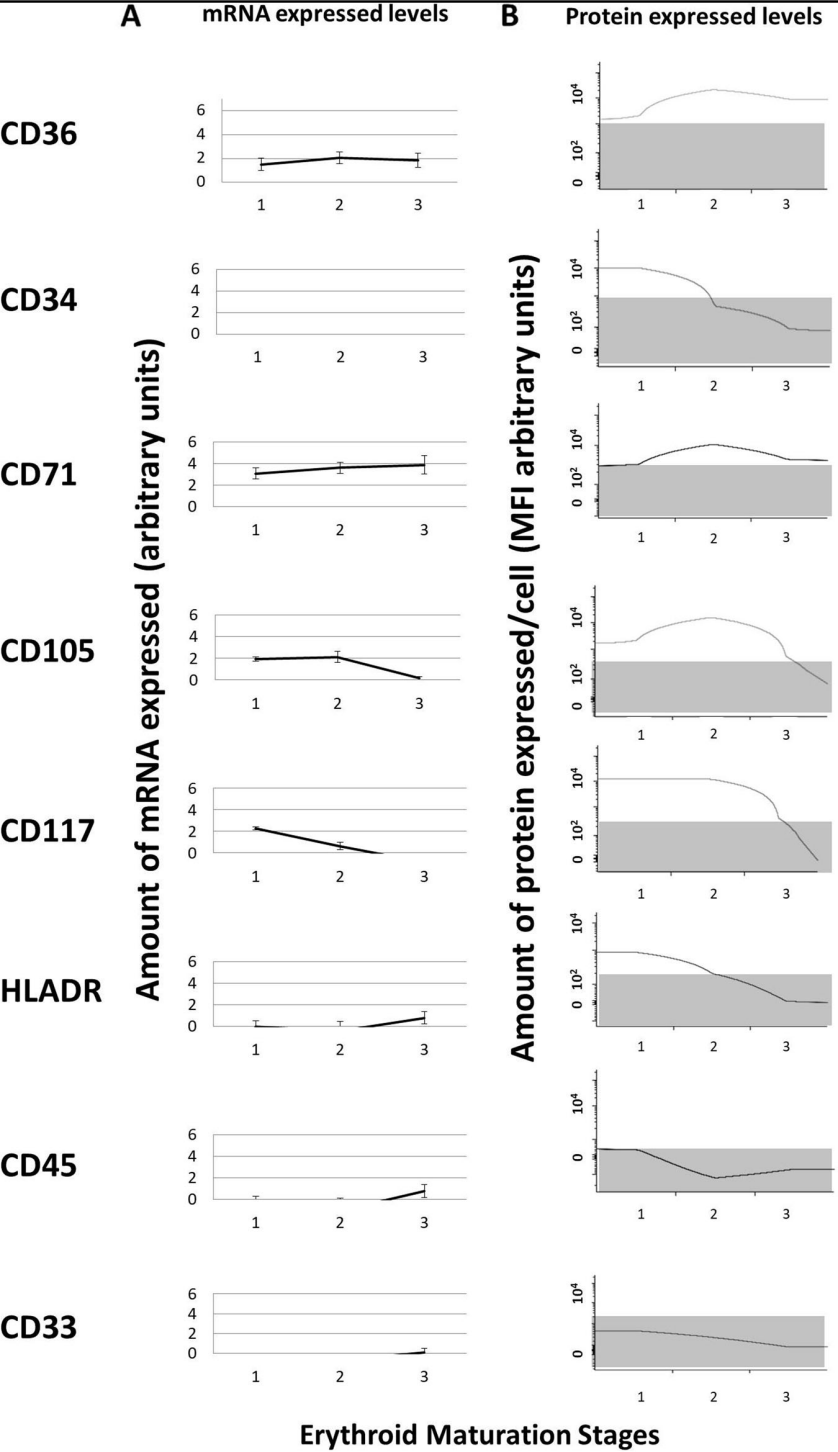
Pattern of expression of proteins (and their corresponding mRNA levels) used to delineate the different stages of maturation of NRBC in human BM. In panel **a**, the intensity of the fluorescence signal obtained by microarray analysis of GEP mRNA levels for those eight immunophenotypic markers used to purify BM NRBC precursors, are shown, while in panel **b**, median fluorescence intensity (MFI) protein expression values, as assessed by multiparameter flow cytometry (arbitrary units scaled from 0 to 2.5 × 10^5^ fluorescence channels), are displayed. In panel **b**, the gray areas highlight regions defined as having no protein expression by flow cytometry

Protein expression levels for the above listed phenotypic markers (as assessed by flow cytometry), was compared with mRNA expression levels for the corresponding genes, showing highly consistent results (Fig. 2), and, as could be expected, intracellular decrease in mRNA levels frequently preceded that observed in the amount of cell surface membrane protein, as illustrated for, e.g., CD117 in Fig. 2. Thus, *CD33, CD34, CD45*, and *HLADR* expression levels were virtually not detected at the mRNA level for any of the three maturation-associated populations of NRBC analyzed, while expression of the CD34, CD45, and HLADR proteins was restricted to the earliest stage of maturation of NRBC precursors, and that of CD33 was systematically absent. In contrast, CD71 and CD36 showed parallel and progressively greater amounts of both mRNA and protein along the erythroid maturation.

### Global transcriptional profile of normal human BM NRBC precursors

**Fig. 3:**
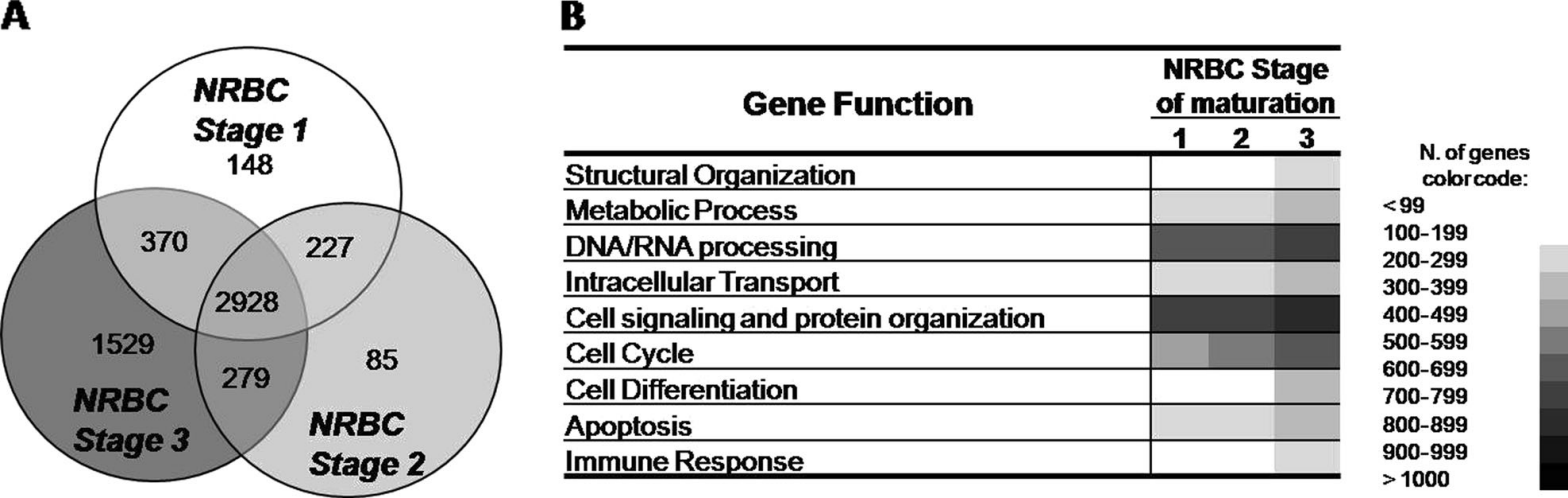
Overall GEP of human BM erythroid precursors at different stages of maturation according to the number of genes expressed, within distinct functional groups. **a** Venn diagram in the left show the number of genes simultaneously and differentially expressed by each population of NRBC precursors analyzed. **b** In the right, color codes are used to differentiate among distinct numbers of genes expressed per maturation stage and group of genes

From all 33,927 genes analyzed, 6569 genes (19%) were expressed in ≥ 1 of the three populations of NRBC analyzed. Almost half of the expressed genes (*n* = 2928 genes) were expressed across all three maturation-associated subpopulations of NRBC precursors evaluated; in contrast, 148, 85, and 1529 genes showed an expression profile restricted to the early (stage 1), intermediate (stage 2), or late stages (stage 3) of maturation of NRBC, respectively. Another 227, 279, and 307 genes were expressed in stage 1 plus stage 2, stage 2 plus stage 3, and stage 1 plus stage 3 NRBC, respectively (Fig. 3a). According to their biological role, those 6569 genes found to be expressed in NRBC could be classified into nine distinct functional gene groups, that included genes involved in: (*i*) structural cell organization, (*ii*) metabolic processes, (*iii*) DNA/RNA processing, (*iv*) intracellular transport, (*v*) cell signaling and protein organization; (*vi*) cycle cell, (*vii*) cell differentiation, (*viii*) apoptosis, and (*ix*) the immune response.

As expected, a high number of genes expressed by BM NRBC precursors were functionally related to DNA/RNA processing (e.g., *HIST1H1E, HIST1H1B*, and *HIST1H2BM* histones) and cell signaling and protein organization (e.g., the *RPS20, RPL19*, and *RPL30* ribosomal protein genes), and they were expressed across all maturation stages of NRBC precursors, although the number of expressed genes within both functional groups slightly increased from stage 2 to stage 3 NRBC precursors (Fig. 3). A GEP similar to that of this later gene group was (i.e., stable GEP during the first two stages of maturation, followed by increased expression in stage 3 NRBC precursors) also found throughout the whole human BM erythroid maturation, but for a lower number of genes, for genes related to (*i*) cell differentiation (e.g., *RPS6, MED1, HCLS1*), (*ii*) metabolic processes, (*iii*) structural organization of the cell (e.g., *EPB42, PLEK2, PDLIM7, ROCK1*), (*iv*) intracellular transport, and (*v*) apoptosis (e.g., *P2RX1, DDIT3, RBM38, CUL4A*). In contrast, the number of cell cycle-associated genes expressed at the RNA level already increased in the transition from stage 1 to stage 2 (e.g., *CDC20, SIPA1, DTL)* human BM erythroid precursors, whereas histone-binding transcriptional activators showed either stable (e.g., *KAT2A*) or increased (e.g., CY*BRD1*) mRNA levels at the last stage of maturation of NRBC precursors. Genes involved in the regulation of erythroid differentiation (e.g., the *CDK6* and *STAT5A* genes) and anti-apoptotic (i.e., survival) mechanisms (e.g., *DDX42*, *MEX3D,* and *SERBP1* genes) were mostly expressed among stage 1 NRBC, while genes involved in the immune response were expressed at relatively low numbers, predominantly in stage 3 precursors (Fig. 3).

### GEP of erythroid lineage-associated markers during normal human BM erythropoiesis

Overall, maturation of NRBC in human BM was associated with modulation of erythroid differentiation-associated GEP. Thus, transcriptional factors involved in erythroid specification of hematopoietic stem cells and erythroid differentiation such as the *GATA1, STAT2, FOG1*, and *KLF1* genes, were expressed across all maturation stages, in the absence of *PU.1* expression (multipotentiality transcription factor), while expression of the *STAT5A* gene involved in EpoR signaling progressively decreased with maturation, being absent in stage 3 NRBC (Table 1). Similarly, expression of the *GFI1B* and *TAL1* genes increased in stage 2 NRBC, and either remained stable (*GFI1B* gene) or increased further (*TAL1* gene) thereafter (Table 1). In contrast, *JAK2* and *PTPN6* were upregulated only in stage 3 NRBC (Table 1). In turn, genes involved in the synthesis of heme such as *ALAS2* and *ALAS1* reached their maximum levels of expression at the more mature (stage 3) NRBC precursors, whereas expression of enzymes involved in degradation of heme (e.g., the *HO1* and *HO2* genes) was absent or very low across all three erythroid maturation stages analyzed (Table 1).

**Table 1:** Genes differentially expressed during erythropoiesis distributed according to their biological functions into genes associated with cell differentiation, apoptosis and immune response

Gene function	Genes expressed differentially per erythroid maturation stage					
	Expressed in all stages (1–3)	Only stage 1	Both stages 1 + 2	Only stage 2	Both stages 2 + 3	Only stage 3
Cell diferentiation						
Hematopoiesis & regulation of cell differentiation	*ADD2, ASH2L, BCL11A, CD164, CNN2, CTNNB1, FOG1, GATA1, GFI1B, GLRX5, HIPK1, IKZF1, MKNK2, PICALM, RAF1, ROGDI, RUNX1, SP3, SPI1, STAT2, TAL1*	*DICER1, PPP2CA, PPP2R1A, ROD1, SOX9, UNCX GATA2, NKX2-5, TAZ, ZBTB16*	*CDK6, KIT*	*RPA1*	*SP1, TACC3*	*FES, LYN, PPP2CA, PPP2R1A, ROD1, SIK1, SOX9, SPOCK2, UNCX, EBP, NKX2-5, SOD2, SOX6, TAZ, TIPARP, TTC7A, TXNRD2, ZBTB16*
Primitive hematopoiesis	*–*	*STK3, STK4, THOC5*	*–*	*–*	*–*	*–*
Erythrocyte differentiation	*ACIN1, ADD1, ALAS2, CITED2, FOG1, GATA1, KLF1, KLF2, MAEA, MLL5, RCOR1, RPS14, RPS19, SFXN1, SLC25A38, STAT2, TAL1, TIMP1, ZFPM1*	*–*	*STAT5A*	*EPOR*	*EPB42, MED1, RPS6, SLC11A2, SRF*	*BCL6, BPGM, DYRK3, ERCC2, HCLS1, HIPK2, JAK2, JMJD6, LYN, NCKAP1L, PTPN6,SOX6 TRIM10*
Heme metabolism	*ALAD, ALAS2, BLVRB, CPOX, FECH, FXN, HMBS, SLC25A38, SLC25A39, SPTA1, TMEM14C, TSPO, UROD, UROS*	*–*	*BLVRA*	*–*	*ALAS1, SLC11A2*	*EIF2AK1, HMOX2, PPOX*
Iron homeostasis	*CPOX, FXN, SFXN1, TFRC, UROD*	*–*	*–*	*–*	*CYBRD1, SLC11A2*	*CIAO1, FBXL5, FTL, ISCA1, ISCA2, ISCU, LCN2, SLC11A1, SLC22A17, SLC25A28, SLC25A37, SOD2*
Erythroblast enucleation	*MAEA*	*–*	*–*	*–*	*MED1*	*ID2, RB1, SP1, SP3*
Oxygen homeostasis/ response to hypoxia or to oxidative stress	*ACTN4, ADRBK1, AGTRAP, ATPIF1,ALAS2, ARNT, CAT, CITED2, CREBBP, CRYAA, CST3, DUSP1, ECE1, ENG, EP300, GAB1, GPX1, GPX4, HIF1A, HMBS, HSP90B1, HYOU1, LIAS, MAP2K1, MLH1, MMP8, MMP9, MT3, NAPRT1, NDUFA12, NDUFA6, NDUFS2, NDUFS8, NOL3, NPPC, PEBP1, PML, PNKP, PPP1R15B, PRDX3, PRDX6, PRNP, RAF1, SCAP, SHC1, STAT5B, STK25, TFRC, TSC2, TXNIP, UBQLN1, UCP2, USF1, XRCC1, WRN*	*BTG3, DDIT3, ERCC3, NUDT1, SMAD3, UCN*	*ERCC1, PKLR, TXN2*	*LONP1, STK25*	*EPX, GCLC, OXSR1, PAK1, PRDX2, PRDX5, SLC11A2, SMAD4, TGFB1*	*ADM, DDIT3, EGLN1, EGLN2, ERCC2, HMOX2, IPCEF1, MSRA, MTF1, NAPRT1, NFKB1, NR4A2, NRF1, NUDT1, PARK7, PKM2, PTGS2, PTK2B, RGS14, SERPINA1, SIRT1, SOD2, THBS1, TNFRSF1A, TPM4, UBE2B, UCN, VEGFA, VNN1*
Apoptosis						
Induction of apoptosis	*AKT1, AATF, ARHGAP4, ARHGEF2, BAD, BAX, BID, BCLAF1, BNIP3L, CASP2, CDKN1B, CEBPB, C14orf153, CUL3, CUL4A, DAP, DPF2, DUSP1,DYNLL2, DEDD2, FEM1B, FASTK, HRK, HRAS, HTRA2, ITM2B, KLF10, KAT2A, KAT2B, LGALS1, MAPK1, NDUFS3, NME3, NCSTN, NET1, PDCD5, PSENEN, PML, MTCH1, PPP2CA, PPP2R1A, PPP3R1, PTEN, RBM38, RPS3, RUNX3, SART1, SERINC3, SHISA5, SIVA1, SMNDC1, STK17B, STK24, SENP1, SKI, SPN, SQSTM1, TGM2, TNFRSF12A, TNFRSF25, TP53, TP53BP2, TRAF7,USP7, UBA52, UBB, UBC, QRICH1, YWHAE, YWHAB*	*BBC3, ERKC3, HIP1, LEF1, MADD, PLAGL2, SMAD3, STK4, TERF1, TP53INP1*	*BTG1, MAGED1, NACC1*	*EI24, SOS1, DYNLL1*	*MAP3K5, NMT1, PYCARD, SOS2, TGFB1*	*APP, ABR, AKAP13, ARHGEF12, ARHGEF18, BBC3, BCL2L11, BTG1, CFLAR, CIDEB, CARD9, CASP8, DAPK2, DUSP2, DEDD, ERCC2, HIP1, HIPK2, JMY, LGALS12, LGD3, MADD, MAL, P2RX1, PLAGL2, PREX1, SAP30BP, SMPD2, SORT1, STK4, THBS1, TIAL1, TNFRSF1A, TNFSF8, TP53INP1, TRAF2, TRAF6, TRIM35, VAV1*
Cellular component involved in apoptosis	*ACTIN1, ACTN4, ADD1, CDH1, CALR, CTNNB1, FNTA, H1F0, HMGB1, HMGB2, KPNB1, LMNA, PSMA1, PSMA2, PSMA7, PSMB2, PSMB3, PSMB4, PSMB6, PSMB7, PSMC2, PSMC3, PSMC4, PSMD1, PSMD11, PSMD3, PSMD7, PSMD8, PSME1, PSME2, PSME3, PSME4, PSMF1, PSMG2 RTN4, STK24, VIM*	*PSMA6, PSMB1*	*HSPD1, PSMC5*	*LMNB1*	*ATG1, ATG2, ATG4, ATG9, DBNL, mTOR, PPIF, RB1CC1*	*BCAP31, BIRC2, DNM1L, IL6R, GSN, KPNA1, PRKCD, ROCK1, SATB1, PSMB10*
Anti-apoptosis	*AATF, AKT1, ANXA1, ARHGDIA, ATPIF1, AZU1, BAG4, BIRC6, BRAF, CBX4, CDC20, CFL1, CITED2, CRYAA, C19orf2, CSTB, DAD1, DTL, FOXC1, FOXC2, FOXO1, GPX1, GSTP1, HDAC1, HDAC3, HSP90B1, HSPA5, HSPA9, HSPB1, IER3, IGFIR, IRAK1, MCL1, MKL1, MYB88, NANOS3, NFKBIA, NOL3, NPM1, PHB, PRNP, RELA, RIPK2, SIPA1, SH3GLB1, SNCA, SOD1, SON, SQSTM1, SYVN1, TPT1, UBA52, UBB, UBC, VCP, WRN*	*BCL3, INHBB*	*CEBPB, DDX42, MEX3D, PGAP2, SERBP1, SUPV3L1*	*TMX1*	*CDKN2D, PRDX2, SEMA4D*	*ANXA5, BAG1, BCL3, BCL2A1, BCL2L1, BNIP2, CTSB, CEBPB, CFLAR, DDAH2, GCLC, HBXIP, IFI6, INHBB, MAPK8IP1, MYO18A, NFKB1, NR3C1, NOS1AP, PIM2, POLB, PDPK1, PROK2, SOD2, TAX1BP1, THBS1, TNFAIP3, TNFRSF18, TNFRSF4, TCF7, SIRT1, TRAF6, VEGFA, VNN1*
Immune response						
Immune response	*STAT1, STAT3, STAT5B*	*INHBB*	*_*	*_*	*_*	*AQP9, ARHGDIB, B2M, BCAP31, BPI, C1QBP, C5AR1, CSF3R, CST3, CD7, CD48, CD74, CAECAM8, CHIT1, COL4A3BP, CSF3, CST7, CTSG, CTSS, FCAR, FCGRT, FOXO3, FYB, GPR44, GTPBP1, HLA-B, HLA-C, HLA-DMA, HLA-DPA1, HLA-DPB1, HLA-DRA, HLA-E, HLA-F, IFI6, IFITM2, IGF1R, IGLL1, IGSF6, IL6, IL6R, IL1R2, IL4R, ILF2, IRF8, INHBB, KCNN4, LILRA3, LAT, LSP1, LCP2, LST1, LTB, MADCAM1, MICB, MLF2, NFIL3, NOTCH1, PRG2, PRG3, PTGER4, S1PR4, SEMA4D, SPN, TCF12, TCF7, TNFRSF14, TNFSF8, TNIP1, VAV1, WAS, XBP1*
Inflammatory response	*_*	*_*	*_*	*_*	*_*	*ABCF1, ADAM8, AGER, AIF1, AKT1, ANXA1, AOAH, AOC3, AZU1, BCL6, C1orf38, C3AR1, CCL5, CCR7, CD97, CEBPB, CHST2, F11R, FOS, HCK, HDAC4, HDAC5, HNRNPA0, IGFBP4, IL17C, IL17D, IL18RAP, IL8, ITCH, ITGAL, KDM6B, JAK2, LAT, LIAS, LTA4H, LTB4R, LYN, LYZ, MAP2K3, MAPKAPK2, MMP25, MYD88, NDST1, NFAM1, NFATC3, NFKB1, NFKBID, NFKBIZ, NFX1, OLR1, ORM1, PARP4, PARK7, PIK3CD, PIK3CG, PLAA, PRDX5, PROK2, PTX3, PTGS2, PXK, RELA, RXRA, S100A12, S100A8, S100A9, SERPINA1, SGMS1, SBNO2, SLC11A1, SEMA7A, TBK1, TGFB1, THBS1, TNFRSF1A, TNFRSF4, TOLLIP, UCN, VNN1*
Innate immune response	*AKIRIN2, ATF2, CAPZA1, CD46, CHID1, CNPY3, CREB1, CREBBP, CRISP3, DDOST, DUSP7, ECSIT, ELK1, EP300, FOS, HMGB1, HSP90AB1, IFITM2, IRAK1, IRF3, JUN, LCN2, MAP2K1, MAP2K2, MAP2K3, MAP3K1, MAP3K5, MAPKAPK3, MAVS, MYD88, NFKBIA, NFKBIB, PCBP2, PIN1, POLR3E, POLR3K, PRKCSH, RPS6KA1, S100A12, SRPK1, TOLLIP, TRIM25, UBB, UBC, UBE2L6, UBE2N*	*ITCH, MAPKAPK2, PELI1, SYK, UBA7*	*CSF1, RELA, RIPK2, TXNIP, UBA52*	*PIK3R4, POLR3H, TXN*	*CAPZA2, MAPK1, PGLYRP1, PYCARD*	*AGER, APOBEC3A, APP, BCL2L1, CARD9, CLEC4D, DUSP6, GZMM, HCK, IFITM1, IL18RAP, IRF7, ITCH, JAK2, LGALS3, LILRA5, LYN, MAPK14, MAPK3, MAPKAPK2, MEF2A, NFKB1, NFKB2, NLRC5, OTUD5, PELI1, PIK3C3, PIK3CD, PSTPIP1, RELA, RIPK2, RPS6KA3, SIGIRR, SYK, TAX1BP1, TBK1, TMEM173, TNFAIP3, TRAF2, TRAF6, TXNIP, UBA52, UNC93B1, VNN1*

Similarly, several genes associated with the homeostasis of iron (e.g*., FBXL5, FTL*) were predominantly, or exclusively, expressed at the latest stage of maturation of NRBC (stage 3) here analyzed; in contrast, expression of other genes related to iron homeostasis/mitochondrial transportation (e.g., *SLC25A38, SCL25A39,* and *SFXN1*), was stable along all erythroid maturation stages. A similarly stable GEP was observed for most genes associated with the oxidative stress response (e.g., *UPC2, PRDX3, TXNIP, DUSP1, GAB1,* and *GPX1*), with only a few genes showing expression restricted to stage 3 NRBC (e.g., *PARK7*, *EGLN1,* and *SOD2*) (Table 1). *MMP8* and *MMP9* were also expressed in all maturation stages of NRBC, but their levels were gradually upregulated in stage 2 and stage 3 NRBC. Finally, hemoglobin genes (e.g., alpha, beta, delta, mu, gamma, and theta1) were already expressed from the earliest stages of maturation (stage 1), across all erythroid populations analyzed (Table 1 and Supplementary Figure 3).

### Expression profile of genes related to apoptosis, autophagy, and enucleation of NRBC precursors

Concerning apoptosis-associated genes, *PML* and *CASP-2* were both expressed across the three NRBC stages of maturation analyzed, whereas *CASP-3* and *CASP-9* were absent and *CASP8* expression was restricted to the last maturation stage (Table 1). In turn, genes involved in mitochondrial and ribosomal clearance such as the *ATG2, ATG4, ATG9, mTOR, ATG1* (*ULK1*) and *RB1CC1* genes, were progressively upregulated during erythroid maturation, and genes associated with NRBC enucleation (e.g., *MED1*, *MAEA*, *ID2*, *RB1*, *SP1*, and *SP3*) showed a predominant or restricted expression in stage 3 NRBC (Table 1). Interestingly, the *MAEA* gene—macrophage erythroblast attach gene—coding for a protein that mediates the linkage between erythroblasts and macrophages in the enucleation process during terminal erythroid maturation, displayed a stable expression across all NRBC maturation stages (Table 1).

#### GEP for immune response-associated markers during normal human BM erythropoiesis

Interestingly, several genes associated with the immune response (e.g., *FOXO3, BPI, CAECAM8, S100A8, S100A9, VNN1*, and *THBS1* genes) were upregulated at the last stage of maturation of NRBC here analyzed. Genes involved in response to proinflammatory cytokines and in activation of the IkappaB kinase (IKK) within the NF-kappaB pathway (e.g., *TRAF6* gene), showed restricted expression to more mature (stage 3) NRBC. In turn, genes of the *STAT* family, such as *STAT5B, STAT1*, and *STAT3*, were expressed across all maturation stages, although the later two genes showed slightly decreased levels in stage 2 *vs*. both stage 1 and 3 NRBC, and the *GDF15* gene was absent in all NRBC maturation stages analyzed (Table 1).

## Discussion

Up till now, only a few studies have investigated the GEP of normal human BM erythroid precursors^10–12^. However, in these such studies, analysis of the GEP of in vitro differentiated erythroid precursor cells was performed^13^, which might not directly reflect the real in vivo GEP profiles. Here, we evaluated the ex vivo GEP of distinct maturation-associated subsets of erythroid precursors directly isolated from (fresh) normal human BM. Based on a previously established 8-color antibody combination validated by the EuroFlow consortium^14^ for the identification of well-defined stages of maturation of NRBC in human BM, three distinct maturation-associated subsets of NRBC were identified for their further isolation and subsequent analysis of their GEP. As previously demonstrated, the three purified NRBC populations corresponded to NRBC precursors presenting morphologic features of proerythroblasts, basophilic, and both polychromatic/orthochromatic erythroblasts, respectively (data not shown)^15^. Of note, the isolated NRBC populations showed a high purity as confirmed by both flow cytometry and the absence of expression of genes related to other hematopoietic cell lineages, together with strong expression of erythroid-specific and erythroid-associated genes.

Overall, our results suggest that from the less differentiated to the more mature erythroid precursors, less requirements for molecules involved in cell division (e.g., absence/low *SMARCB1, PFDN1, TRNP1* expression) exist, in parallel to preserved expression of genes related to cell cycle arrest (e.g., *RBM38, CUL4A, KAT2B*), and an increased expression of genes involved in both cell cycle regulation (e.g., *CDC20, SIPA1, DTL*) and cell differentiation (e.g., *RPS6, MED1, HCLS1*), structural organization (e.g., *PLEK2, PDLIM7, ROCK1*), and signal transduction^16^ (e.g., *P2RX1*); all these later genes were expressed in parallel to genes involved in apoptosis and the immune response as discussed below.

At present, it is well-established that Epo signaling via the EpoR/Jak2/Stat5 pathway that coordinates differentiation, proliferation, and survival of erythroid precursors^17,18^, is critical for the production of erythroid cells^19^. Overall, our results showed *EPOR* gene expression throughout the whole human BM erythroid maturation, with greater levels at intermediate and late stages of differentiation; these results are in contrast with murine studies that show strongly decreased Epo levels at the more advanced stages of erythropoiesis^20^. Previous studies have shown that the expression of the above genes is strongly modulated by oxygen availability^19^. Thus, hypoxia alters progression through erythropoiesis, via modulation of the *EPOR* signaling pathway and induction of specific erythroid transcription factors involved in signaling for the production and accumulation of hemoglobin during erythropoiesis^21^, such as the *GATA1, FOG1, TAL1*, *KLF1,* and *GFI1B* genes^22,23^. In this regard, *GATA-1* is currently considered to be the major transcriptional factor for erythroid and megakaryocytic differentiation, via repression of genes involved in progenitor multipotentiality like *PU.1*^2,16^. Besides, *GATA-1* also promotes G1 cell cycle arrest during terminal erythroid differentiation via repression of mitogenic genes^24,25^ and upregulation of anti-apoptotic genes^26^ and other transcriptional factors (e.g., *FOG-1* and *KLF-1*) involved in the biosynthesis of globins and heme^27^, which are critical for an efficient production of erythroid cells. In line with this, here we found strong and stable expression across all NRBC maturation stages of the *GATA-1* as well as the *FOG-1*, *STAT2*, *STAT5B*, and *KLF1* transcription factors, similar to what has been previously described in in vitro human models^28^. However, despite similarly stable GEP across the whole erythropoiesis were identified for a high number of genes, an overall tendency toward downregulation of multiple genes between stages 1 and 2, together with upregulation of other genes in stage 3 *vs*. stage 2 NRBC precursors, was observed.

In this regard, as could be expected, genes that code for enzymatic complexes involved in the synthesis of heme such as the *ALAS1* and *ALAS2* genes^29^ were highly expressed across all erytropoietic maturation stages analyzed. Thus, *ALAS2* expression progressively increased during erythroid maturation, while increase in the expression of *ALAS1* was less pronounced, delayed and restricted to the more mature (stage 3) erythroid precursors. Similarly, genes involved in iron assembly, transport and homeostasis (e.g., *ISCA1* and *ISCA2*), as well as in iron transfer and transport (e.g., *SLC11A2*, *SLC25A39,* and *SLC25A38*) progressively increased during erythroid maturation. In turn, proteases involved in extracellular matrix digestion, such as MMP8 and MMP9, were also found to be progressively upregulated in stage 2 and 3 NRBC precursors. *MMP9* expression is known to be suppressed in the presence of heme oxygenase-1 (HO-1)^30^, an enzyme involved in the catabolism of heme with antioxidant and anti-inflammatory functions^31^, found to be absent throughout erythropoiesis, in parallel to low gene expression levels (restricted to latest maturation stage) of the (constitutive) HO-2 isoform. These results are in line with previous observations from our group showing absence of the HO-1 protein on normal human BM erythroblasts^31^, despite the abundance of its strongest inducer (i.e., heme)^32^. Altogether, these results suggest differential heme-dependent regulation of HO-1 expression in erythroid cells, in order to prevent potentially redundant cycles of heme biosynthesis and degradation.

Because of significant production of pro-oxidant molecules during erythropoiesis such as heme and hemoglobin^33^ erythroblasts also require a tight regulation of the redox machinery. Such oxidative stress might contribute to progressively higher expression levels of genes involved in the metabolism of radical oxygen species (ROS), such as the *UCP2* and *PARK7* oxidative stress response genes. Other genes, found to be expressed throughout erythropoiesis, that are also involved in protecting cells from oxidative stress, included the *PRDX3, TXNIP, DUSP1, GAB1*, and *GPX1* genes, which code for relevant antioxidant enzymes that act on hydrogen peroxide detoxification and protection of hemoglobin and other oxidative breakdown-associated proteins^34,35^.

Interestingly, an overall similar GEP was observed for both apoptosis and cell differentiation-associated genes suggesting a direct link between them. Thus, several genes within both functional groups were expressed in stage 1 precursors and decreased thereafter (e.g., *KAT2A* and *PML*), while other genes (e.g., *PTPN6* and CY*BRD1*) were upregulated at the latest maturation stage, including the *EPB42* (Erythrocyte Membrane Protein Band 4.2) gene involved in mechanical regulation and shaping of erythrocytes^36^, which progressively increased its expression from stage 2 to stage 3 NRBC. Apoptosis is a physiologic process essential for normal tissue development and differentiation^37^, which is typically associated with caspase activation, collapse of the nuclear envelope, DNA break-up into small fragments, and changes in cell shape and surface, leading to the exposure of signaling molecules to phagocytic cells^37^. Several caspases (e.g., *CASP-3, CASP-8, CASP-9*) have been found to be expressed during* in vitro* erythroblast differentiation^38^. Here we found continuous and stable expression of *CASP-2* and putative expression of *CASP-8* at the latest stage of maturation of NRBC precursors, in the absence of both *CASP-3* and *CASP-9*; these observations suggest the involvement of death receptors instead of mitochondrial signaling pathways^39^ in erythropoietic apoptotic signaling cascades. Other apoptosis-associated genes, such as the *TP53INP1* gene coding for the tp53inp1 tumor suppressor protein involved in the regulation of autophagy^40^, a critical process in erythropoiesis enucleation and removal of mitochondrial and other organelles^41^, were also expressed on both early and late (erythroid) maturation stages. In turn, the *ATG2, ATG4, ATG9, mTOR, ATG1* (*ULK1*), and *RB1CC1* genes, which are critical for mitochondrial and ribosomal clearance (e.g., *mTOR* and its downstream target *ULK1* gene) and for autophagosome fusion (e.g., *ATG4*)^42^ and complex formation (e.g., *ULK1* and *RB1CC1*) during terminal erythroid differentiation^43^, were progressively upregulated during erythroid maturation. Of note, expression of *ULK1* was inversely related to that of *PML*, the later gene acting as an inhibitor of *mTOR*^44^. Other genes involved in the enucleation of erythrocytes such as the *ID2* and *RB1* genes, showed an expression profile restricted to the latest stage of maturation, in parallel to genes associated with oxygen homeostasis such as the *EGLN1* and *SOD2* genes.

A relative unexplored aspect of erythropoiesis is its relationship with immune response-associated genes. Several studies have highlighted the potential role of genes associated with the immune system, like *FOXO3*, in modulating the production and differentiation of erythroid precursors^45^. In addition to upregulation of *FOXO3* at the more mature NRBC maturation stages, in line with previous observations^46^, functional analysis of such maturation stage-associated GEPs showed upregulation of several cell differentiation and immune response-associated genes at intermediate and late stages of maturation. Among other genes, these included genes that are triggered by (*i*) environmental stress conditions, leading to an increased cell permeability (e.g., *BPI*) and adhesion (e.g., *CAECAM8*) and (*ii*) the innate host defense (*CRISP3*)^47–49^. In turn, expression of other genes involved in response to proinflammatory cytokines and in the activation of the IkappaB kinase (*IKK*) within the NF-kappaB pathway, such as the *TRAF6* gene, was restricted to the more mature stage 3 erythroid precursors; these findings are in contrast with previous (human) in vitro findings showing downregulation of TRAF6 during erythroid maturation^10^. In parallel to TRAF6, the DDIT3 transcription factor, involved in pro-apoptotic signaling associated to cell stress responses, was also upregulated in the more mature (stage 3) NRBC both here and in previous in vitro studies^10^.

Regarding the STAT gene family, slightly different GEP *vs.* previously in vitro data were found here^10,50^. Thus, our results showed a transiently lower *STAT1* and *STAT3* expression at stage 2 NRBC which recovered later on, while* in vitro* studies showed a continuous progressively lower expression of these genes^10^. Similarly, while *STAT2* expression remained stable here, it appeared to be downregulated in previous in vitro studies^10^. In contrast, similar GEP were found here and in previous in vitro studies for the *STAT5A* (gene expression levels progressively downregulated along erythroid differentiation, being absent in the more mature erythroid precursors), and *STAT5B* (expressed at similar levels throughout the whole erythropoiesis). These differences observed between our ex vivo and previous in vitro studies in the expression pattern of *STAT5* gene might be due to the different in vitro and in vivo activation conditions (e.g., erythropoietin doses). In line with this hypothesis, distinct ex vivo (absence of expression) and in vitro (upregulation) GEP^10^ were also observed for the *GDF15* gene, a gene whose hyperexpression has been associated with both ineffective erythropoiesis or stressed erythropoiesis^51^. Altogether, these findings further support the relevance and complementarity of both types of in vitro and ex vivo studies.

In summary, here we provide for the first time detailed information about the ex vivo GEP of normal BM human erythroid precursor cells at different maturation stages. The results presented extend on and complement previous in vitro data, contributing to a better understanding of erythroid maturation and providing a frame of reference for the study of erythropoiesis in both neoplastic and non-neoplastic disease conditions.

## Methods

### BM samples

BM aspirated samples were obtained from healthy volunteers (2 males and 3 females; age range: 11–53 years). Prior to entering the study, each individual gave his written informed consent to participate according to the Declaration of Helsinki. The study was approved by the local ethics committee of the Cancer Research Centre (Salamanca, Spain).

### Isolation of distinct subsets of normal human BM erythroid precursors

Fresh human BM samples were incubated with an ammonium chloride buffer to lyse mature non-nucleated red cells, washed twice in phosphate buffered saline (PBS), and stained with an 8-color combination of monoclonal antibodies, strictly following the EuroFlow standard operating procedures (SOPs)^14^. To stain BM cells, the following antibody reagents were used: CD34 (clone 8G12) conjugated with peridinin chlorophyll protein/cyanine 5.5 (Becton Dickinson Biosciences (BD), San Jose, CA), CD45 (clone HI30) conjugated with pacific orange (ExBio, Vestec, Czech Republic), CD36 (clone SLB-IVC7) conjugated with fluorescein isothiocyanate (BD), CD105 (clone 1G2) conjugated with phycoerythrin (Beckman Coulter (BC), Brea, CA), HLADR (clone L243) conjugated with pacific blue (BioLegend, San Diego, CA), CD117 (clone 104D2D1) conjugated with phycoerythrin/cyanine 7 (BC), CD33 (clone P67,6) conjugated with allophycocyanine (BD), and CD71 (clone M-A712) conjugated with allophycocyanine/cyanine 7 (BD). Stained cells were subsequently centrifuged (250 × *g* for 10 min), the cell pellet washed once (250 × *g* for 10 min) in 2 mL of PBS and resuspended in 0.5 mL of PBS. Stained samples were measured in either a FACSAria I or a FACSAria III flow cytometers (BD) equipped with the FACSDiva software (BD). Prior to sorting, 10^5^ cells per BM sample were measured in the flow cytometer and used to define the gating regions (Fig. 1) for subsequent isolation of the distinct maturation-associated subsets of BM erythroid precursors. Overall, three distinct subpopulations of nucleated red blood cell precursors (NRBC) were identified and FACS-sorted, including (from the less differentiated to the more mature cells): (1) CD34^+^ HLADR^+^ CD117^low^ CD36^low^ CD45^low^ CD105^+^ CD33^−^ CD71^low^ (stage 1) erythroid precursors (5.7% ± 0.5% of all NRBC); (2) CD34^−^ HLADR^−^ CD117^−^ CD36^+^ CD45^−^ CD105^high^ CD33^−^ CD71^+^ (stage 2) NRBC (9.6% ± 0.8% of all NRBC); and (3) CD34^−^ HLADR^−^ CD117^−^ CD36^high^ CD45^−^ CD105^+/−^ CD33^−^ CD71^high^ (stage 3) red cell precursors (84.7% ± 1.3% of all NRBC). For data analysis, the Infinicyt software program (Cytognos SL, Salamanca, Spain) was used. For each cell population, between 5600 and 500,000 cells were sorted directly into PBS, immediately centrifugated, and resuspended in 100 μL of RA1 buffer (NucleoSpin® RNA XS kit, Macherey-Nagel, Düren, Germany). The purity of the isolated cell populations (> 97%) was confirmed through re-analysis of ≈ 500 sorted cells per cell fraction and sample.

### RNA extraction and gene expression profiling studies

GEPs were assessed on identical amounts of total mRNA extracted from each of the three purified subpopulations of BM NRBC. For this purpose, total mRNA was extracted using the NucleoSpin RNA XS kit, strictly following the instructions of the manufacturer. The quality and concentration of the extracted mRNA were evaluated using the Agilent 2100 Bioanalyzer and the RNA6000 Nanochips and Picochips (Agilent Technologies, Santa Clara, CA). GEPs were determined using the Affymetrix GeneChip Human Gene 1.0 ST Array (Affymetrix), strictly following the procedures recommended by the manufacturer.

For GEP data analysis, raw data (Affymetrix.CEL files) was normalized using the robust multi-array average (RMA) algorithm, which included background correction, quantile normalization, log2 transformation, and probe set summarization of GEP raw data. The Affymetrix GeneChip Human Gene 1.0 ST Array negative (*n* = 1195) and positive (*n* = 2904) control probes were measured in parallel; cut-off values for positive gene expression signals were set at the 1st lower quartile of the positive control gene probe signals (Supplementary Figure 1).

Genes that were differentially expressed between distinct maturation-associated subsets of BM erythroid precursors were ranked based on the comparison between their expression values in two consecutive stages of (erythroid) maturation, using the “Gene Set Enrichment Analysis” (GSEA) software^52^. Subsequently, the GeneCodis (Gene Annotation Co-occurrence Discovery; Madrid, Spain)^53^ annotation tool was used for gene ontology and functional enrichment analyses for those genes that were found to be expressed either at stable or variable levels during human BM erythroid maturation (Supplementary Figure 2).

### Quality control of GEP of purified BM nucleated erythroid precursors at different stages of maturation

As an internal control for the GEP results obtained for each of the distinct cell populations, we evaluated the expression of genes that are known to be either specific or characteristic of erythroid cells *vs.* other hematopoietic cell lineages. Thus, hemoglobin genes (i.e., *HBA, HBB, HBD, HBM, HBG,* and *HBQ1*) were strongly expressed in the three distinct populations of maturation-associated erythroid precursors, while those genes coding for the embryonic hemoglobin chains (*HBE1* and *HBZ*) were absent. In contrast, genes related with the neutrophil (e.g., *CD16a* or *FcγRIIIa*), lymphocyte (e.g., *CD19* B-lymphocyte antigen), and monocytic (e.g., *CD14* or myeloid cell-specific leucine-rich glycoprotein) cell lineages, were systematically absent in all different FACS-sorted maturation-associated subsets of BM erythroid precursors analyzed (Supplementary Figure 3). In order to validate microarray data, gene expression for the *HMOX1, HMOX2*, *GYPA*, *ALAS1*, and *ALAS2* genes was investigated in parallel for all purified maturation stage-associated nucleated red cell populations by real-time PCR (ABI 7500 Real-Time PCR System, Applied Biosystems) using the SYBR Green I double-stranded DNA-specific fluorophore (Power SYBR^®^ Green PCR Master Mix; Applied Biosystems) (Supplementary Figure 4). cDNAs were amplified using 40 cycles of PCR (denaturation for 15″ at 95 °C; annealing and extension for 60″ at 60 °C). Real-time PCR results were normalized by GAPDH mRNA expression levels, and Ct values were calculated using 2^−ΔΔCt^, where ΔΔCt = ΔCt “treatment” − ΔCt control.

## Supplementary information

Supplemental Material File #1

Supplementary Figure 1

Supplementary Figure 2

Supplementary Figure 3

Supplementary Figure 4

Supplementary Table
