# Supplementary material for: Maturation-associated gene expression profiles during normal human bone marrow erythropoiesis
Source: Cell Death Discov. 2019 Feb 28;5:69. doi: 10.1038/s41420-019-0151-0 (PMC6395734; doi:10.1038/s41420-019-0151-0)
Supplement: Supplementary file 1 — Supplemental Material File #1 [file 41420_2019_151_MOESM1_ESM.docx]

**LEGENDS TO SUPPLEMENTARY FIGURES:**

**Supplementary Figure 1. Cutoff values used to define positive gene expression fluorescence intensity levels (arbitrary units).** Cutoff values were based on the fluorescence intensity of both the negative (n=2,904) and positive (n=1,195) control genes in the arrays evaluated for the distinct maturation associated subsets of NRBC (i.e. cut-off were set at first quartile of the positive control genes).

**Supplementary Figure 2. Graphical representation of the pairwise comparison of the gene expression levels detected between two consecutive stages of maturation of human BM erythroid precursor cells.** The following comparisons are shown in the scatterplots: stage 1 *vs.* stage 2 (panel A), stage 2 *vs.* stage 3 (panel B), stage 1 *vs.* stage 3 (panel C); (D) heatmap of genes expressed during erythroid maturation classified according to their biological functions.

**Supplementary Figure 3.** **Gene expression profile that characterizes erythroid lineage cells. (A)** Hemoglobin genes/expression during erythroid maturation (Hemoglobin δ, μ and ξ were expressed at all three stages while hemoglobin ζ and ε were not); **(B)** GEP for the *CD71*, *CD105*, *CD36* (positive) and *CD33* (negative) markers used to identify the FACS-sorted subsets of NRBC. **(C)**GEP observed for *EPO* – Erythropoietin; *EPOR* – Erythropoietin receptor; *GYPA* –Glycophorin and *CD163*; **(D)** No expression of genes characteristic of the neutrophil (*CD16a*), lymphocyte (*CD19*) and monocyte (*CD14*) lineages, was found.

**Supplementary Figure 4.** **Gene expression profile that characterizes erythroid lineage cells comparing microarray and RT-PCR assays. (A)***HMOX1 -* Heme-oxygenase 1 was not observed in both techniques used; **(B-E)** *HMOX2* - Heme-oxygenase 2, *GYPA* - Glycophorin A, *ALAS1* and *ALAS2* – 5’-Aminolevulinate Synthase 1 and 2 were expressed in low levels at stage 1 and stage 2, however, in the more mature stage (Stage 3), they were expressed in greater quantity. The profiles are very similar between the two techniques.

**Supplementary Table 1. Detailed description of genes expressed during erythroid maturation classified according to their biological functions.**
