# Supplementary figures and images for: Maturation-associated gene expression profiles during normal human bone marrow erythropoiesis
Source: Cell Death Discov. 2019 Feb 28;5:69. doi: 10.1038/s41420-019-0151-0 (PMC6395734; doi:10.1038/s41420-019-0151-0)

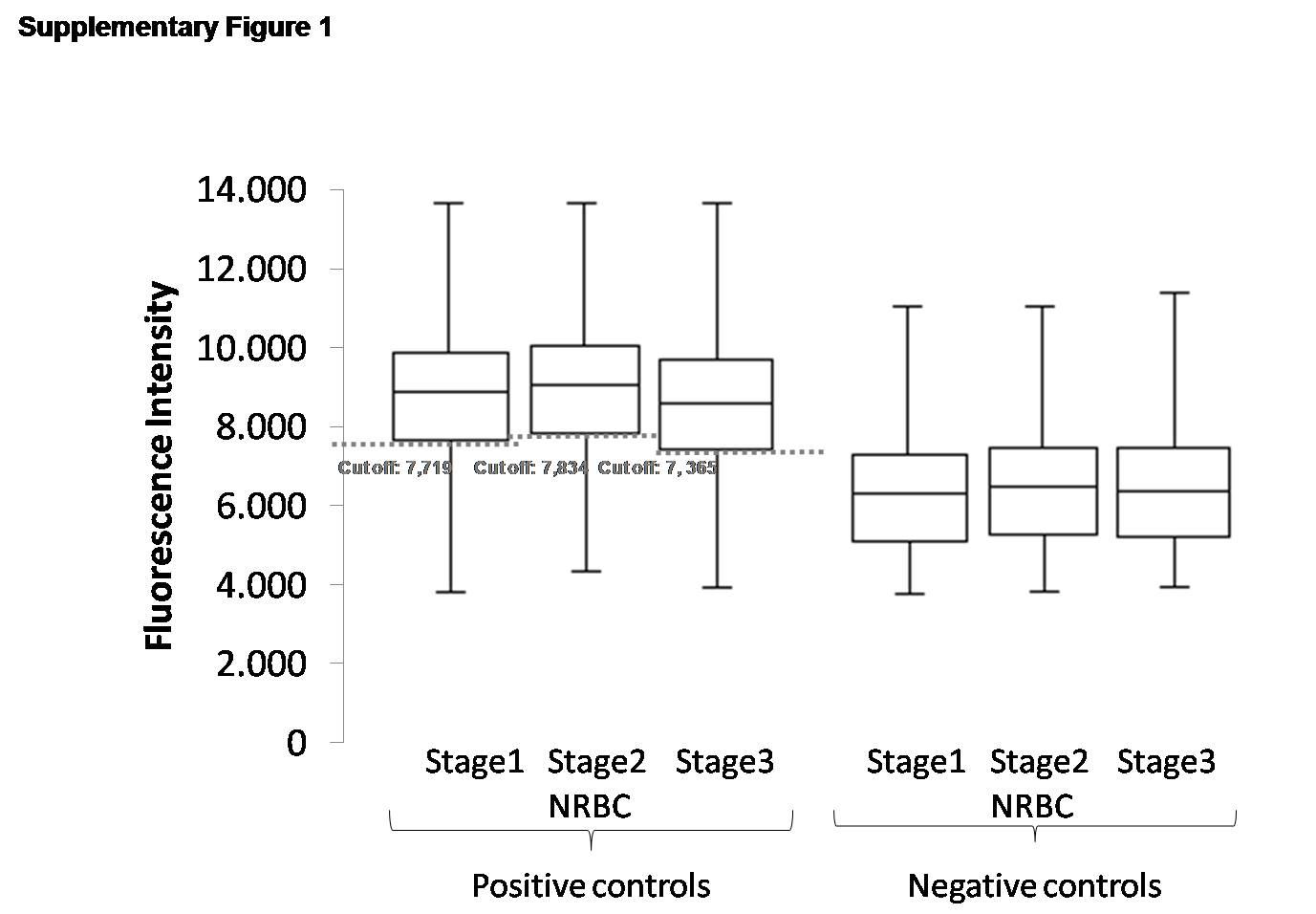

Supplement: Supplementary file 2 — Supplementary Figure 1 [file 41420_2019_151_MOESM2_ESM.jpg]

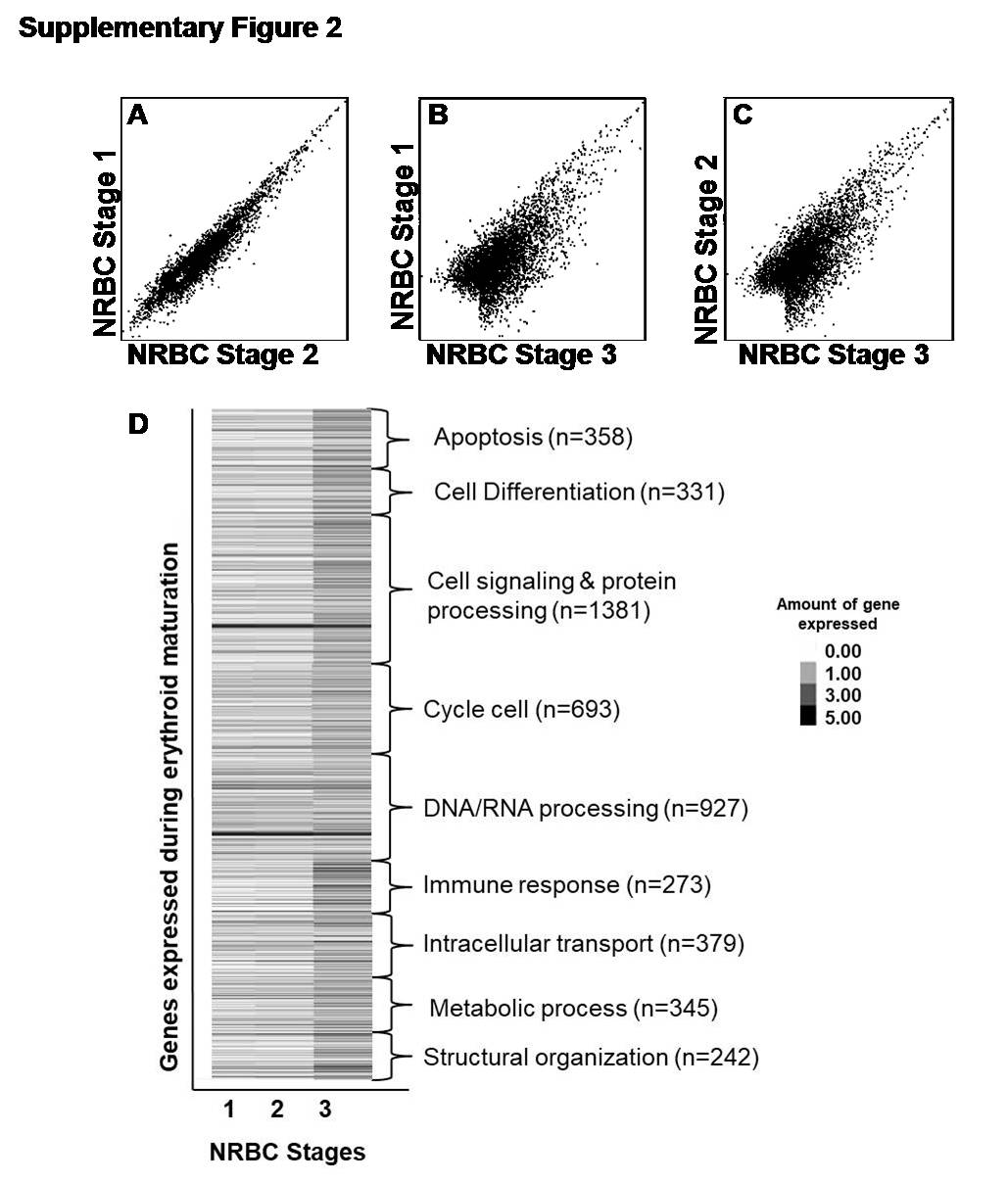

Supplement: Supplementary file 3 — Supplementary Figure 2 [file 41420_2019_151_MOESM3_ESM.jpg]

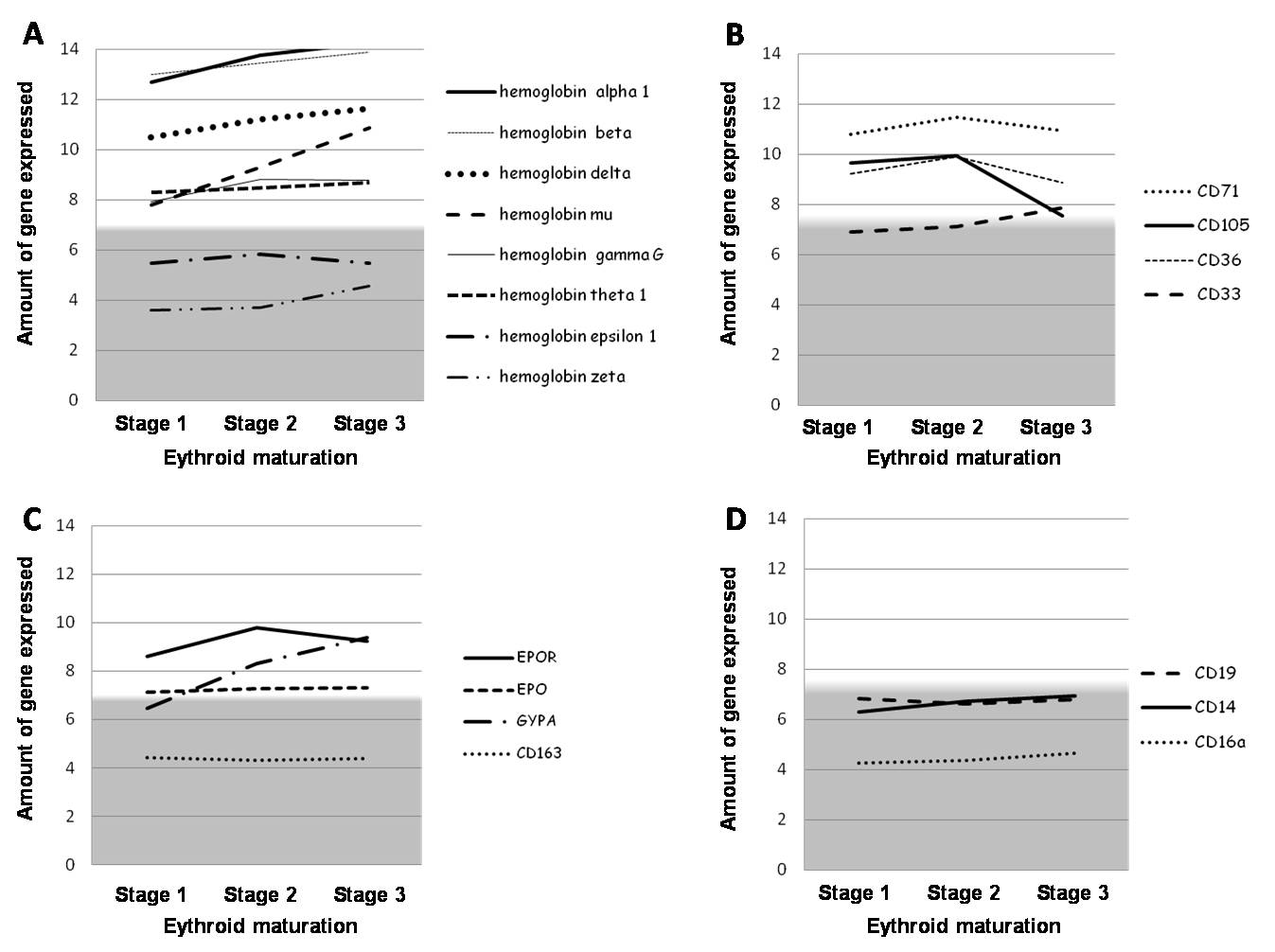

Supplement: Supplementary file 4 — Supplementary Figure 3 [file 41420_2019_151_MOESM4_ESM.jpg]

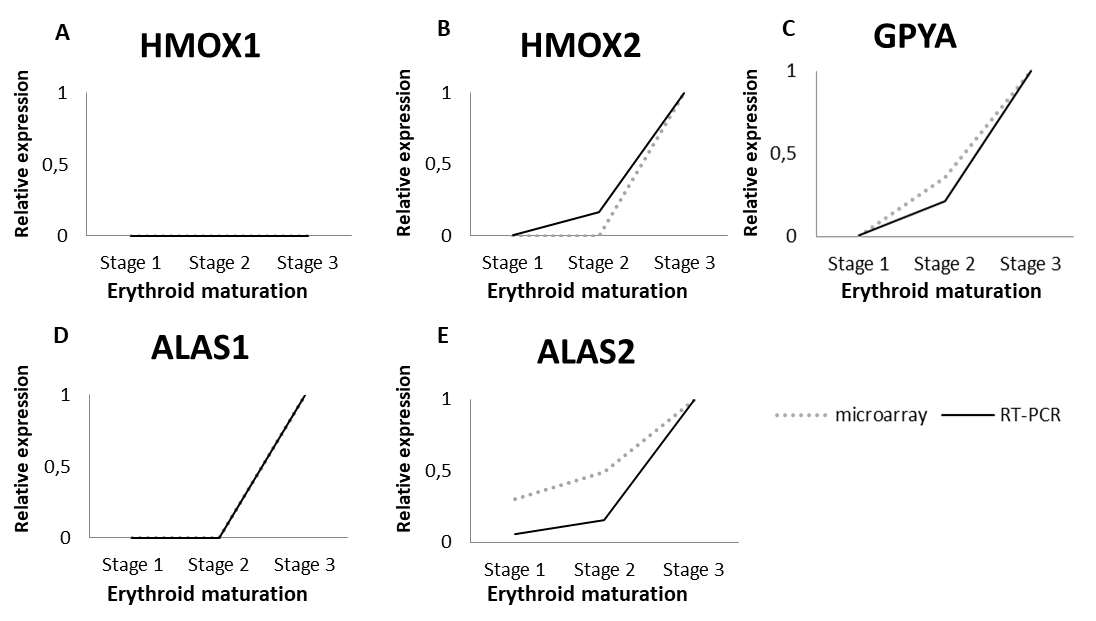

Supplement: Supplementary file 5 — Supplementary Figure 4 [file 41420_2019_151_MOESM5_ESM.png]
