# Supplementary material for: Maturation-associated gene expression profiles during normal human bone marrow erythropoiesis
Source: Cell Death Discov. 2019 Feb 28;5:69. doi: 10.1038/s41420-019-0151-0 (PMC6395734; doi:10.1038/s41420-019-0151-0)
Supplement: Supplementary file 6 — Supplementary Table [file 41420_2019_151_MOESM6_ESM.docx]

| Ensembl ID | Gene | NRBC Stage1 | NRBC Stage2 | NRBC  Stage3 | Biological Function |
| --- | --- | --- | --- | --- | --- |
| ENSG00000201492 | AATF | 0,649128 | 0,261702 | 0,564653 | apoptosis |
| ENSG00000202474 | ABR | -1,1632 | -1,18525 | 0,410738 | apoptosis |
| ENSG00000240649 | ACIN1 | 0,84472 | 0,636077 | 1,132513 | apoptosis |
| ENSG00000183355 | ACTN1 | 0,687377 | 1,066761 | 2,748633 | apoptosis |
| ENSG00000123908 | ACTN4 | 2,437775 | 2,628628 | 3,074963 | apoptosis |
| ENSG00000239735 | ADD1 | 0,724013 | 1,137125 | 1,665387 | apoptosis |
| ENSG00000196364 | ADRB1 | -0,12117 | -0,43203 | 0,225187 | apoptosis |
| ENSG00000188403 | AEN | -0,00977 | 0,12017 | -0,07209 | apoptosis |
| ENSG00000100412 | AIMP2 | 0,385726 | 0,541809 | -0,20368 | apoptosis |
| ENSG00000142513 | AKAP13 | -0,9579 | -1,08135 | 0,731816 | apoptosis |
| ENSG00000181786 | AKT1 | 1,744731 | 1,7493 | 1,843017 | apoptosis |
| ENSG00000243173 | ANXA1 | 1,757694 | 1,972089 | 5,086203 | apoptosis |
| ENSG00000181437 | ANXA5 | -1,53662 | -1,37492 | 0,347057 | apoptosis |
| ENSG00000089053 | APP | -0,82592 | -0,80844 | 0,718683 | apoptosis |
| ENSG00000196510 | ARF6 | 1,411891 | 1,36479 | 1,772039 | apoptosis |
| ENSG00000105186 | ARHGAP4 | 0,622939 | 0,568716 | 2,220368 | apoptosis |
| ENSG00000164236 | ARHGDIA | 0,964977 | 0,789008 | 2,077855 | apoptosis |
| ENSG00000100124 | ARHGEF12 | -0,695 | -0,29776 | 2,094691 | apoptosis |
| ENSG00000156381 | ARHGEF18 | -0,34164 | -0,27071 | 0,746291 | apoptosis |
| ENSG00000064999 | ARHGEF2 | 0,277696 | 0,558861 | 1,424291 | apoptosis |
| ENSG00000104880 | ATPIF1 | 0,852117 | 1,200774 | 0,579499 | apoptosis |
| ENSG00000213465 | AXIN1 | -0,2756 | -0,5623 | 0,152226 | apoptosis |
| ENSG00000182196 | AZU1 | 0,236817 | 1,109149 | 2,958423 | apoptosis |
| ENSG00000130429 | BAD | 0,166576 | 0,094479 | 0,559147 | apoptosis |
| ENSG00000163466 | BAG1 | -0,17388 | -0,23385 | 0,458101 | apoptosis |
| ENSG00000111229 | BAG4 | 0,652208 | 0,129469 | 0,732698 | apoptosis |
| ENSG00000173409 | BAX | 0,734194 | 0,704375 | 0,974735 | apoptosis |
| ENSG00000148331 | BBC3 | 0,055892 | -0,01567 | 0,555735 | apoptosis |
| ENSG00000100325 | BCAP31 | -0,20421 | -0,28482 | 0,320774 | apoptosis |
| ENSG00000166183 | BCL2A1 | -1,95841 | -1,72993 | 0,514297 | apoptosis |
| ENSG00000169696 | BCL2L1 | -0,04684 | -0,00566 | 3,185443 | apoptosis |
| ENSG00000171456 | BCL2L11 | -0,37861 | -0,50432 | 0,011678 | apoptosis |
| ENSG00000143970 | BCL3 | 0,033871 | -0,10051 | 0,855052 | apoptosis |
| ENSG00000115966 | BCL7C | 0,195389 | -0,05242 | 0,346364 | apoptosis |
| ENSG00000128272 | BCLAF1 | 1,64084 | 1,163531 | 1,802967 | apoptosis |
| ENSG00000130734 | BID | 0,845608 | 0,802377 | 1,525496 | apoptosis |
| ENSG00000119787 | BIRC2 | -0,70432 | -0,51922 | 0,445813 | apoptosis |
| ENSG00000166454 | BIRC6 | 0,089037 | 0,022185 | 0,286536 | apoptosis |
| ENSG00000177556 | BLCAP | 0,708741 | 0,685111 | 1,143647 | apoptosis |
| ENSG00000058668 | BNIP2 | -0,95945 | -0,81709 | 0,358793 | apoptosis |
| ENSG00000152234 | BNIP3L | 1,191324 | 1,653536 | 3,453933 | apoptosis |
| ENSG00000116459 | BRAF | 0,373469 | 0,374479 | 1,144173 | apoptosis |
| ENSG00000167283 | BRE | 0,04066 | -0,05278 | 0,07321 | apoptosis |
| ENSG00000113732 | BTG1 | 1,665762 | 1,49265 | 3,740333 | apoptosis |
| ENSG00000113732 | BUB1B | 0,44896 | 0,577264 | 0,444271 | apoptosis |
| ENSG00000158470 | C14orf153 | -1,70896 | -1,63154 | -1,2676 | apoptosis |
| ENSG00000166619 | C19orf2 | 0,465723 | 0,468383 | 0,109928 | apoptosis |
| ENSG00000183019 | CALR | 2,767475 | 2,733513 | 2,998293 | apoptosis |
| ENSG00000143751 | CARD9 | -0,19037 | -0,37764 | 0,141074 | apoptosis |
| ENSG00000094975 | CASP2 | 0,625451 | 0,6815 | 1,111503 | apoptosis |
| ENSG00000157870 | CASP8 | -0,39152 | -0,54729 | 1,044953 | apoptosis |
| ENSG00000174407 | CBX4 | 0,317664 | 0,077387 | 1,390457 | apoptosis |
| ENSG00000177410 | CCAR1 | 2,308055 | 2,078237 | 1,506589 | apoptosis |
| ENSG00000116161 | CDH1 | 1,554601 | 1,34436 | 0,719785 | apoptosis |
| ENSG00000164047 | CDKN1B | 0,448234 | 0,29123 | 2,116854 | apoptosis |
| ENSG00000127022 | CDKN2D | -0,07326 | 0,164599 | 1,268876 | apoptosis |
| ENSG00000127022 | CEBPB | 0,224206 | 0,215422 | 2,296169 | apoptosis |
| ENSG00000014216 | CFL1 | 2,473145 | 2,359402 | 3,317503 | apoptosis |
| ENSG00000131375 | CFLAR | -1,47228 | -1,30957 | 0,413442 | apoptosis |
| ENSG00000122565 | CIB1 | 0,072248 | 0,147856 | 0,861052 | apoptosis |
| ENSG00000122565 | CIDEB | -0,07001 | -0,24291 | 0,099129 | apoptosis |
| ENSG00000176476 | CITED2 | 1,887047 | 1,831663 | 2,996803 | apoptosis |
| ENSG00000160799 | CKAP2 | 0,81978 | 0,979541 | 1,233522 | apoptosis |
| ENSG00000136807 | CREB1 | 0,284631 | 0,585755 | 1,217715 | apoptosis |
| ENSG00000183307 | CRYAA | 0,727103 | 0,200046 | 0,738935 | apoptosis |
| ENSG00000100162 | CSE1L | 0,476326 | 0,600227 | -0,08929 | apoptosis |
| ENSG00000172757 | CSNK2A2 | 1,451911 | 1,349229 | 1,20129 | apoptosis |
| ENSG00000172757 | CSRNP1 | -0,79158 | -0,57929 | 0,61965 | apoptosis |
| ENSG00000163320 | CST3 | 2,721195 | 1,881301 | 2,009755 | apoptosis |
| ENSG00000106153 | CSTB | 1,040834 | 1,135719 | 1,641894 | apoptosis |
| ENSG00000110721 | CTNNB1 | 1,019751 | 0,511799 | 1,89983 | apoptosis |
| ENSG00000104472 | CTSB | -0,10535 | -0,03604 | 2,120365 | apoptosis |
| ENSG00000145354 | CUL3 | 0,976359 | 0,747572 | 1,376645 | apoptosis |
| ENSG00000122966 | CUL4A | 0,210534 | 0,156687 | 0,868145 | apoptosis |
| ENSG00000011021 | CYFIP2 | -1,47923 | -1,09309 | 1,641694 | apoptosis |
| ENSG00000113282 | DAD1 | 1,438411 | 1,917436 | 1,85037 | apoptosis |
| ENSG00000013441 | DAP | 0,443065 | 0,385974 | 0,864945 | apoptosis |
| ENSG00000176444 | DAPK2 | -0,38745 | -0,46475 | 1,001209 | apoptosis |
| ENSG00000128973 | DBNL | -0,12249 | 0,029895 | 1,175338 | apoptosis |
| ENSG00000125107 | DDAH2 | -0,54722 | -0,75321 | 0,084501 | apoptosis |
| ENSG00000080573 | DDX41 | 0,970734 | 1,530957 | 0,710129 | apoptosis |
| ENSG00000142173 | DDX42 | 0,651851 | 0,078068 | 0,167585 | apoptosis |
| ENSG00000049089 | DDX47 | 0,425636 | 0,109541 | -0,18362 | apoptosis |
| ENSG00000165644 | DEDD | -0,12953 | -0,0658 | 0,204942 | apoptosis |
| ENSG00000122218 | DEDD2 | 0,699057 | 0,968203 | 2,331395 | apoptosis |
| ENSG00000121671 | DNM1L | -0,34237 | -0,36953 | 0,026625 | apoptosis |
| ENSG00000103653 | DPF2 | 1,667846 | 1,477829 | 1,180001 | apoptosis |
| ENSG00000230673 | DUSP1 | 2,877685 | 2,997073 | 3,321373 | apoptosis |
| ENSG00000144579 | DUSP2 | -0,16114 | -0,24549 | 0,572057 | apoptosis |
| ENSG00000196188 | DYNLL1 | -0,09546 | 0,07288 | -0,82347 | apoptosis |
| ENSG00000100448 | DYNLL2 | 1,567453 | 1,270732 | 1,571947 | apoptosis |
| ENSG00000142544 | E2F1 | 1,398456 | 1,577908 | 1,057178 | apoptosis |
| ENSG00000178531 | E2F2 | -1,12901 | -0,46658 | 1,597879 | apoptosis |
| ENSG00000154639 | ECE1 | 0,497982 | 0,747598 | 1,667194 | apoptosis |
| ENSG00000197380 | EI24 | -0,05268 | 0,132618 | -0,12651 | apoptosis |
| ENSG00000165359 | ELMO1 | 0,488732 | -0,11256 | 0,508145 | apoptosis |
| ENSG00000123136 | ELMO2 | -0,62755 | -0,68833 | -0,01911 | apoptosis |
| ENSG00000108654 | EP300 | 0,856371 | 0,804801 | 2,852863 | apoptosis |
| ENSG00000184014 | ERCC2 | -0,18662 | -0,07666 | 0,218708 | apoptosis |
| ENSG00000136986 | ERCC3 | 0,278499 | -0,13482 | -0,61082 | apoptosis |
| ENSG00000162496 | ESPL1 | -0,89472 | -0,09935 | 0,322015 | apoptosis |
| ENSG00000117395 | FAM82A2 | 0,101918 | 0,748071 | 1,236806 | apoptosis |
| ENSG00000104529 | FASTK | 0,421196 | 0,557636 | 0,550092 | apoptosis |
| ENSG00000100353 | FEM1B | 0,501567 | 0,32881 | 0,655353 | apoptosis |
| ENSG00000100353 | FGD3 | -0,10844 | -0,20795 | 1,368281 | apoptosis |
| ENSG00000175390 | FIS1 | -0,04383 | 0,138107 | 0,56021 | apoptosis |
| ENSG00000084623 | FKBP8 | 1,873371 | 1,976211 | 3,276233 | apoptosis |
| ENSG00000110321 | FNTA | 0,934363 | 0,440675 | 0,486616 | apoptosis |
| ENSG00000110321 | FOXC1 | 0,557946 | 0,357164 | 0,652288 | apoptosis |
| ENSG00000110321 | FOXC2 | 0,648688 | 0,237607 | 0,586436 | apoptosis |
| ENSG00000110321 | FOXO1 | 0,97159 | 0,668033 | 1,43042 | apoptosis |
| ENSG00000110321 | FOXO3 | 0,632537 | 0,465749 | 1,572566 | apoptosis |
| ENSG00000197774 | FXR1 | 0,654492 | 0,208052 | 0,667139 | apoptosis |
| ENSG00000135638 | FZD5 | -1,11953 | -0,73711 | 0,483326 | apoptosis |
| ENSG00000166947 | GABARAP | 1,545243 | 2,14376 | 3,300593 | apoptosis |
| ENSG00000072134 | GADD45A | -0,75855 | -0,57711 | 1,2342 | apoptosis |
| ENSG00000049283 | GADD45B | -0,02859 | -0,20438 | 0,605152 | apoptosis |
| ENSG00000187266 | GADD45G | -1,38471 | -1,0714 | 0,359524 | apoptosis |
| ENSG00000144488 | GCLC | -0,18691 | 0,344415 | 2,373697 | apoptosis |
| ENSG00000130201 | GHITM | 0,113436 | 0,24808 | 0,746855 | apoptosis |
| ENSG00000158769 | GLO1 | -0,13046 | 0,118308 | -0,1771 | apoptosis |
| ENSG00000143409 | GPX1 | 1,048289 | 1,01409 | 1,663225 | apoptosis |
| ENSG00000140525 | GSN | -0,37817 | -0,15377 | 2,14367 | apoptosis |
| ENSG00000149806 | GSTP1 | 1,582459 | 1,540584 | 2,045008 | apoptosis |
| ENSG00000132004 | GZMM | -0,23524 | -0,51996 | 0,566286 | apoptosis |
| ENSG00000186431 | H1F0 | 3,044165 | 3,390778 | 4,005253 | apoptosis |
| ENSG00000119321 | HBXIP | -0,69385 | -0,60035 | 0,050935 | apoptosis |
| ENSG00000196924 | HDAC1 | 0,330075 | 0,330649 | 0,501639 | apoptosis |
| ENSG00000132589 | HDAC3 | 1,193067 | 1,621457 | 1,08631 | apoptosis |
| ENSG00000136877 | HINT2 | 0,408953 | 0,4184 | -0,03205 | apoptosis |
| ENSG00000171051 | HIP1 | -2,22226 | -2,47624 | -1,57939 | apoptosis |
| ENSG00000165879 | HIPK1 | 2,392855 | 2,245453 | 3,259653 | apoptosis |
| ENSG00000181274 | HIPK2 | -0,21543 | -0,39928 | 1,541122 | apoptosis |
| ENSG00000175857 | HMGB1 | 0,325895 | 0,184994 | 0,305622 | apoptosis |
| ENSG00000106105 | HMGB2 | 2,196495 | 2,415733 | 2,817743 | apoptosis |
| ENSG00000005955 | HRAS | 0,691947 | 0,46913 | 0,939411 | apoptosis |
| ENSG00000231643 | HRK | 0,840291 | 0,772821 | 1,283202 | apoptosis |
| ENSG00000101216 | HSP90B1 | 3,048385 | 2,909708 | 2,489153 | apoptosis |
| ENSG00000173540 | HSPA5 | 2,784725 | 3,009378 | 2,727353 | apoptosis |
| ENSG00000163655 | HSPA9 | 1,736692 | 1,51933 | 1,105319 | apoptosis |
| ENSG00000146535 | HSPB1 | 1,756866 | 2,218378 | 1,186864 | apoptosis |
| ENSG00000060558 | HSPD1 | 1,251503 | 1,363158 | -0,16699 | apoptosis |
| ENSG00000087460 | HTRA2 | 0,415052 | 0,756521 | 1,13352 | apoptosis |
| ENSG00000172354 | HTT | 0,112716 | -0,16812 | 0,343767 | apoptosis |
| ENSG00000197858 | IER3 | 0,517105 | 0,644293 | 1,876402 | apoptosis |
| ENSG00000068394 | IFI6 | -0,3496 | -0,44692 | 0,347403 | apoptosis |
| ENSG00000158292 | IGF1R | 0,31585 | 0,358973 | 1,46631 | apoptosis |
| ENSG00000235581 | IL6R | -1,20592 | -1,03196 | 0,094357 | apoptosis |
| ENSG00000184674 | INHBB | 0,055211 | -0,19449 | 0,159783 | apoptosis |
| ENSG00000170180 | IRAK1 | 0,255255 | 0,605751 | 0,614264 | apoptosis |
| ENSG00000206177 | ITM2B | 1,784463 | 1,8473 | 2,928933 | apoptosis |
| ENSG00000164818 | JAK2 | -1,06168 | -0,16865 | 0,033743 | apoptosis |
| ENSG00000126107 | JMJD6 | -0,29757 | -0,50427 | 0,9667 | apoptosis |
| ENSG00000136929 | JMY | -0,52612 | -0,9664 | 0,243982 | apoptosis |
| ENSG00000156875 | KAT2A | 1,357626 | 0,842454 | 0,031347 | apoptosis |
| ENSG00000198558 | KIAA0141 | -0,13251 | -0,28785 | 0,385038 | apoptosis |
| ENSG00000180448 | KIAA1967 | 0,13799 | 0,078646 | 0,563139 | apoptosis |
| ENSG00000135486 | KIF1B | -1,33452 | -1,44043 | 0,333004 | apoptosis |
| ENSG00000092199 | KLF10 | 0,362196 | 0,278023 | 0,614082 | apoptosis |
| ENSG00000169045 | KPNA1 | -0,63512 | -0,29379 | 0,254994 | apoptosis |
| ENSG00000165119 | KPNB1 | 0,770783 | 0,862003 | 0,638906 | apoptosis |
| ENSG00000199090 | LCN2 | 1,45788 | 2,190947 | 4,837443 | apoptosis |
| ENSG00000072506 | LEF1 | 0,325708 | -0,57681 | -0,62726 | apoptosis |
| ENSG00000096384 | LGALS1 | 0,924479 | 0,88422 | 1,977342 | apoptosis |
| ENSG00000096384 | LGALS12 | -0,80253 | -0,90923 | 0,035967 | apoptosis |
| ENSG00000115317 | LITAF | -0,23089 | 0,157891 | 2,685643 | apoptosis |
| ENSG00000105376 | LMNA | 0,736682 | 1,02773 | 0,158468 | apoptosis |
| ENSG00000167862 | LMNB1 | -0,38064 | 0,039765 | -0,07181 | apoptosis |
| ENSG00000143621 | LUC7L3 | 0,339805 | -0,17878 | 0,320947 | apoptosis |
| ENSG00000166333 | LYN | -0,33403 | -0,36232 | 1,632926 | apoptosis |
| ENSG00000169592 | MADD | 0,141432 | -0,14142 | 0,368801 | apoptosis |
| ENSG00000173404 | MAGED1 | 0,258481 | 0,398759 | -1,12117 | apoptosis |
| ENSG00000164880 | MAL | -0,26928 | -0,4408 | 0,0402 | apoptosis |
| ENSG00000205339 | MAP1S | -0,03046 | 0,010036 | 0,465843 | apoptosis |
| ENSG00000090376 | MAP3K5 | -0,10312 | 0,03711 | 0,040707 | apoptosis |
| ENSG00000185507 | MAPK1 | 0,453091 | 1,116785 | 2,384759 | apoptosis |
| ENSG00000135070 | MAPK8IP1 | -1,44066 | -1,7262 | -1,33451 | apoptosis |
| ENSG00000211459 | MCL1 | 1,242523 | 1,411873 | 3,248893 | apoptosis |
| ENSG00000092051 | MDM4 | 0,953677 | 0,203691 | 0,821608 | apoptosis |
| ENSG00000065427 | MEF2A | -0,32384 | -0,4377 | 0,380704 | apoptosis |
| ENSG00000065427 | MEF2D | 0,184979 | 0,158576 | 1,398387 | apoptosis |
| ENSG00000165572 | MEX3D | 0,311403 | 0,106184 | 0,612643 | apoptosis |
| ENSG00000178342 | MFSD10 | -0,02161 | 0,128508 | 1,956124 | apoptosis |
| ENSG00000079999 | MKL1 | 0,17743 | 0,09753 | 0,955148 | apoptosis |
| ENSG00000121774 | MKNK2 | 1,262593 | 2,025401 | 2,877363 | apoptosis |
| ENSG00000121774 | MLL | 0,526996 | 0,775153 | 0,788817 | apoptosis |
| ENSG00000100647 | MNT | -0,06838 | -0,05576 | 0,719809 | apoptosis |
| ENSG00000109265 | MPO | -0,1946 | 1,540662 | 3,079153 | apoptosis |
| ENSG00000157404 | MRPL41 | 0,621883 | 0,493538 | 0,652395 | apoptosis |
| ENSG00000221864 | MTCH1 | 0,349596 | 0,834738 | 1,884286 | apoptosis |
| ENSG00000172155 | MYD88 | 0,191777 | 0,35892 | 1,574111 | apoptosis |
| ENSG00000043462 | MYO18A | -0,90231 | -0,39809 | 0,473523 | apoptosis |
| ENSG00000050426 | NACC1 | 0,168994 | 0,004071 | -0,05041 | apoptosis |
| ENSG00000104826 | NANOS3 | 0,371363 | 0,17527 | 0,806903 | apoptosis |
| ENSG00000153395 | NCSTN | 0,183853 | 0,025015 | 0,378231 | apoptosis |
| ENSG00000116212 | NDUFS3 | 0,500228 | 0,382887 | 0,302493 | apoptosis |
| ENSG00000170860 | NET1 | 1,319872 | 0,526525 | 0,154022 | apoptosis |
| ENSG00000108848 | NFKB1 | -0,33621 | -0,73829 | 0,464338 | apoptosis |
| ENSG00000147507 | NFKBIA | 2,952725 | 2,516024 | 3,730603 | apoptosis |
| ENSG00000156009 | NISCH | 0,442668 | 0,41199 | 0,586256 | apoptosis |
| ENSG00000140400 | NME3 | 0,614515 | 0,132294 | 0,326884 | apoptosis |
| ENSG00000126934 | NMT1 | -0,02084 | 0,372623 | 0,094485 | apoptosis |
| ENSG00000169967 | NOL3 | 0,496521 | 0,103498 | 0,558145 | apoptosis |
| ENSG00000012983 | NOS1AP | -0,34009 | -0,58731 | 0,168036 | apoptosis |
| ENSG00000101367 | NPM1 | -1,54685 | -1,66528 | -1,30858 | apoptosis |
| ENSG00000075413 | NR3C1 | -0,57173 | -0,24055 | 0,660994 | apoptosis |
| ENSG00000143797 | NTN1 | 0,355061 | 0,239243 | 0,319586 | apoptosis |
| ENSG00000197006 | OPA1 | 0,26387 | -0,36007 | 0,08863 | apoptosis |
| ENSG00000141503 | P2RX1 | -0,18116 | -0,63936 | 1,387692 | apoptosis |
| ENSG00000075975 | PACS2 | 0,004303 | -0,08868 | 0,01658 | apoptosis |
| ENSG00000130382 | PAK1 | -0,23112 | 0,430701 | 0,482848 | apoptosis |
| ENSG00000171843 | PAK4 | 0,089085 | -0,03057 | 0,114496 | apoptosis |
| ENSG00000128309 | PCNT | 0,088698 | 0,054556 | 1,180306 | apoptosis |
| ENSG00000115364 | PDCD1 | -0,61083 | -0,83643 | 0,023693 | apoptosis |
| ENSG00000112651 | PDCD4 | 1,025643 | 1,48013 | 1,439636 | apoptosis |
| ENSG00000242485 | PDCD5 | 0,821516 | 0,461893 | 0,564272 | apoptosis |
| ENSG00000197345 | PDCD6IP | 0,579414 | 0,522718 | 0,505793 | apoptosis |
| ENSG00000116221 | PDPK1 | -0,11665 | -0,05766 | 1,372814 | apoptosis |
| ENSG00000188895 | PGAP2 | 0,186109 | 0,027364 | -0,0658 | apoptosis |
| ENSG00000087250 | PHB | 1,875405 | 1,713283 | 0,332783 | apoptosis |
| ENSG00000147649 | PHF17 | 0,06586 | 0,11819 | 0,316096 | apoptosis |
| ENSG00000132613 | PHLPP1 | 0,008999 | -0,34066 | 0,142729 | apoptosis |
| ENSG00000142347 | PIM2 | -0,29292 | -0,38477 | 1,310457 | apoptosis |
| ENSG00000196531 | PLAGL2 | 0,134596 | -0,02784 | 0,866389 | apoptosis |
| ENSG00000134440 | PLK3 | -0,95772 | -1,429 | 0,148211 | apoptosis |
| ENSG00000121579 | PML | 0,500705 | 0,126281 | 0,002846 | apoptosis |
| ENSG00000071051 | POLB | -0,5859 | -0,75435 | 0,060368 | apoptosis |
| ENSG00000167792 | PPIF | -0,15868 | 0,104689 | 0,345681 | apoptosis |
| ENSG00000129559 | PPM1F | -0,75703 | -0,79978 | 0,103081 | apoptosis |
| ENSG00000123405 | PPP1R15A | 1,501226 | 1,882269 | 3,280573 | apoptosis |
| ENSG00000104825 | PPP2CA | 0,801002 | 0,89556 | 1,927892 | apoptosis |
| ENSG00000167604 | PPP2R1A | 1,738742 | 1,342914 | 1,426215 | apoptosis |
| ENSG00000100138 | PPP3R1 | -1,30461 | -1,65844 | -1,25722 | apoptosis |
| ENSG00000136448 | PRDX2 | -0,30753 | 0,202068 | 0,428842 | apoptosis |
| ENSG00000166197 | PREX1 | -0,31078 | -0,09072 | 1,832533 | apoptosis |
| ENSG00000147140 | PRKCB | -0,69932 | -0,70973 | 1,571204 | apoptosis |
| ENSG00000147140 | PRKCD | -1,26456 | -0,81925 | 1,869165 | apoptosis |
| ENSG00000087269 | PRNP | 0,713078 | 0,902652 | 0,975146 | apoptosis |
| ENSG00000111641 | PROK2 | -0,45496 | -0,31675 | 1,875448 | apoptosis |
| ENSG00000106459 | PSENEN | 1,096793 | 0,845242 | 1,067685 | apoptosis |
| ENSG00000154146 | PSMA1 | 0,511412 | 0,307757 | 0,075845 | apoptosis |
| ENSG00000171119 | PSMA2 | 0,539221 | 0,590382 | 0,710682 | apoptosis |
| ENSG00000165671 | PSMA6 | 0,188446 | -0,02429 | -0,07836 | apoptosis |
| ENSG00000088833 | PSMA7 | 0,111888 | 0,600691 | 0,916087 | apoptosis |
| ENSG00000117697 | PSMB1 | 0,124262 | -0,34835 | -0,36765 | apoptosis |
| ENSG00000107672 | PSMB10 | -0,65802 | -0,82371 | 0,048595 | apoptosis |
| ENSG00000037474 | PSMB2 | 1,87463 | 1,698844 | 1,47286 | apoptosis |
| ENSG00000130305 | PSMB3 | 1,202671 | 1,410919 | 1,311083 | apoptosis |
| ENSG00000125458 | PSMB4 | 1,850704 | 1,364658 | 1,8872 | apoptosis |
| ENSG00000076685 | PSMB6 | 1,125764 | 1,287579 | 0,717843 | apoptosis |
| ENSG00000168268 | PSMB7 | 1,006554 | 0,885053 | 0,62839 | apoptosis |
| ENSG00000205309 | PSMC2 | 0,54618 | 0,754569 | 0,539639 | apoptosis |
| ENSG00000065320 | PSMC3 | 0,937396 | 0,919445 | 0,428637 | apoptosis |
| ENSG00000196358 | PSMC4 | 0,912199 | 0,982313 | 0,745915 | apoptosis |
| ENSG00000013374 | PSMC5 | 0,397263 | 0,242087 | -0,23687 | apoptosis |
| ENSG00000095906 | PSMD1 | 0,44281 | 0,717374 | 0,525304 | apoptosis |
| ENSG00000104805 | PSMD11 | 0,557114 | 0,632246 | 0,252652 | apoptosis |
| ENSG00000070081 | PSMD3 | 1,03587 | 0,972807 | 0,752389 | apoptosis |
| ENSG00000069275 | PSMD7 | 1,412024 | 1,606718 | 1,649406 | apoptosis |
| ENSG00000090273 | PSMD8 | 1,95467 | 1,976999 | 1,544596 | apoptosis |
| ENSG00000015676 | PSME1 | 0,935881 | 0,9253 | 1,146034 | apoptosis |
| ENSG00000106268 | PSME2 | 1,077808 | 0,306033 | 0,230474 | apoptosis |
| ENSG00000168101 | PSME3 | 0,012481 | 0,146398 | 0,011534 | apoptosis |
| ENSG00000167005 | PSME4 | 0,778421 | 0,064242 | 1,319399 | apoptosis |
| ENSG00000149761 | PSMF1 | 1,470156 | 1,960602 | 2,593608 | apoptosis |
| ENSG00000083635 | PSMG2 | 1,336038 | 0,922283 | 1,168609 | apoptosis |
| ENSG00000138750 | PTEN | 1,534163 | 1,737146 | 2,702853 | apoptosis |
| ENSG00000162231 | PTK2B | -0,54877 | -0,49445 | 1,579712 | apoptosis |
| ENSG00000139579 | PTPN6 | 0,034339 | -0,08319 | 2,089326 | apoptosis |
| ENSG00000102837 | PUF60 | 0,941452 | 0,899724 | 1,035907 | apoptosis |
| ENSG00000085840 | PYCARD | -0,21847 | 0,024743 | 1,930272 | apoptosis |
| ENSG00000140961 | QRICH1 | 1,757151 | 1,538841 | 1,413757 | apoptosis |
| ENSG00000090060 | RAD21 | 0,668707 | 0,413374 | 1,062095 | apoptosis |
| ENSG00000116288 | RAF1 | 1,003384 | 0,785839 | 1,551141 | apoptosis |
| ENSG00000116288 | RALB | -1,66578 | -1,81732 | 0,319172 | apoptosis |
| ENSG00000168300 | RASSF5 | 0,148928 | 0,21367 | 1,669929 | apoptosis |
| ENSG00000143870 | RBM25 | 1,252346 | 0,924001 | 1,01961 | apoptosis |
| ENSG00000142657 | RELA | 0,519434 | 0,336723 | 0,898974 | apoptosis |
| ENSG00000130024 | RFFL | 0,025995 | 0,509045 | 2,243988 | apoptosis |
| ENSG00000143393 | RHOB | 0,29789 | -0,05702 | 0,351152 | apoptosis |
| ENSG00000087111 | RHOT1 | -1,22694 | -0,72763 | 0,012795 | apoptosis |
| ENSG00000124155 | RHOT2 | 0,462678 | 0,89399 | 1,151633 | apoptosis |
| ENSG00000141506 | RIPK2 | 0,679384 | 0,450826 | 0,974048 | apoptosis |
| ENSG00000127564 | RNF130 | 1,466652 | 0,758475 | 1,436557 | apoptosis |
| ENSG00000145287 | RNF144B | -1,63605 | -1,66028 | 0,040598 | apoptosis |
| ENSG00000106397 | ROCK1 | -0,40211 | -0,50675 | 1,130564 | apoptosis |
| ENSG00000071677 | RPS3 | 4,827735 | 4,821038 | 4,425813 | apoptosis |
| ENSG00000112739 | RRAGA | -0,08599 | -0,3131 | 0,089701 | apoptosis |
| ENSG00000174231 | RTN3 | -1,10769 | -1,31819 | 0,109619 | apoptosis |
| ENSG00000174231 | RTN4 | 0,341362 | 0,286563 | 1,355419 | apoptosis |
| ENSG00000174231 | RUNX3 | 0,339367 | 0,116317 | 1,181805 | apoptosis |
| ENSG00000174231 | RYBP | 0,620252 | 0,326969 | 0,635635 | apoptosis |
| ENSG00000176532 | SAP30BP | -0,27529 | -0,40171 | 0,022917 | apoptosis |
| ENSG00000172382 | SART1 | 0,371706 | 0,966803 | 0,902506 | apoptosis |
| ENSG00000105227 | SATB1 | -0,21383 | -0,39214 | 0,530808 | apoptosis |
| ENSG00000125384 | SEMA4D | -1,4626 | -1,17799 | -0,67523 | apoptosis |
| ENSG00000148334 | SENP1 | 0,179096 | 0,053053 | 0,121331 | apoptosis |
| ENSG00000179295 | SERBP1 | 0,106989 | 0,122587 | -0,34795 | apoptosis |
| ENSG00000111679 | SERINC3 | 1,527802 | 1,620494 | 2,130207 | apoptosis |
| ENSG00000169228 | SGMS1 | -1,05828 | -0,51946 | 0,903358 | apoptosis |
| ENSG00000167964 | SGPP1 | 0,695937 | 0,332094 | 0,333513 | apoptosis |
| ENSG00000196624 | SH3GLB1 | 0,623894 | 0,100914 | 0,098512 | apoptosis |
| ENSG00000111540 | SH3KBP1 | -0,23431 | -0,17776 | 1,110092 | apoptosis |
| ENSG00000075785 | SHB | 0,42463 | 0,058444 | 0,332621 | apoptosis |
| ENSG00000144840 | SHISA5 | 0,37027 | 0,465149 | 1,079262 | apoptosis |
| ENSG00000116473 | SIRT1 | -0,41654 | -0,40145 | 0,127446 | apoptosis |
| ENSG00000109756 | SIVA1 | 0,888423 | 1,039602 | 1,124981 | apoptosis |
| ENSG00000068831 | SKI | 0,934097 | 0,912557 | 1,37916 | apoptosis |
| ENSG00000146587 | SLC11A2 | -0,05335 | 0,704304 | 1,590904 | apoptosis |
| ENSG00000139194 | SLC25A6 | 4,233195 | 4,037683 | 3,699853 | apoptosis |
| ENSG00000076043 | SLC40A1 | 1,300528 | 0,951146 | 1,607948 | apoptosis |
| ENSG00000100263 | SLC9A3R1 | 0,906412 | 0,74364 | 1,838424 | apoptosis |
| ENSG00000067560 | SLK | 0,717091 | 0,577121 | 0,623118 | apoptosis |
| ENSG00000067560 | SLTM | 0,805143 | 0,449032 | 0,469282 | apoptosis |
| ENSG00000067560 | SMAD3 | 0,18712 | -0,24852 | -0,31358 | apoptosis |
| ENSG00000199916 | SMNDC1 | -1,27574 | -0,85571 | -0,90243 | apoptosis |
| ENSG00000169397 | SMPD2 | -1,06686 | -0,77199 | 0,593819 | apoptosis |
| ENSG00000121848 | SNCA | 1,016413 | 1,509554 | 3,699533 | apoptosis |
| ENSG00000197067 | SOD1 | 1,296588 | 1,526291 | 0,918932 | apoptosis |
| ENSG00000225178 | SOD2 | -0,26943 | -0,43738 | 0,91811 | apoptosis |
| ENSG00000212695 | SON | 2,034219 | 1,811847 | 2,728343 | apoptosis |
| ENSG00000178654 | SORT1 | -2,01824 | -1,41772 | 1,437253 | apoptosis |
| ENSG00000234498 | SOS1 | -0,60838 | 0,045255 | -0,94173 | apoptosis |
| ENSG00000223416 | SOS2 | -0,63815 | 0,065346 | 1,756731 | apoptosis |
| ENSG00000223803 | SOX9 | 0,785073 | 0,250017 | 0,964454 | apoptosis |
| ENSG00000235244 | SPN | 1,443186 | 1,370412 | 0,861922 | apoptosis |
| ENSG00000177133 | SQSTM1 | 0,830946 | 0,802821 | 1,488165 | apoptosis |
| ENSG00000236741 | SRGN | 2,948015 | 3,258253 | 5,274383 | apoptosis |
| ENSG00000131469 | STEAP3 | 0,33235 | 0,693943 | 0,49398 | apoptosis |
| ENSG00000108107 | STK17B | 1,66504 | 1,490502 | 3,250983 | apoptosis |
| ENSG00000108107 | STK24 | 0,893669 | 1,155937 | 1,700665 | apoptosis |
| ENSG00000162244 | STK3 | 0,777724 | 0,33534 | 0,886687 | apoptosis |
| ENSG00000156482 | STK4 | 0,080806 | -0,04391 | 1,745589 | apoptosis |
| ENSG00000164587 | SUPV3L1 | 0,780034 | 0,403875 | -0,26269 | apoptosis |
| ENSG00000232818 | SYVN1 | 0,408459 | 0,594434 | 1,463481 | apoptosis |
| ENSG00000170889 | TAOK2 | -0,09706 | -0,49007 | 0,010464 | apoptosis |
| ENSG00000160214 | TAX1BP1 | -0,34622 | -0,30181 | 1,051889 | apoptosis |
| ENSG00000142230 | TCF7 | -0,95762 | -1,04753 | 0,025032 | apoptosis |
| ENSG00000105063 | TERF1 | 0,191327 | -0,38878 | -0,35518 | apoptosis |
| ENSG00000175467 | TGFB1 | -0,06435 | 0,069337 | 1,570909 | apoptosis |
| ENSG00000175467 | TGFBR2 | -0,74445 | -0,60434 | 1,100291 | apoptosis |
| ENSG00000075856 | TGM2 | 1,24576 | 0,990112 | 1,026158 | apoptosis |
| ENSG00000075856 | THBS1 | -2,0025 | -1,02608 | 0,866713 | apoptosis |
| ENSG00000075856 | THOC1 | 0,545195 | 0,219667 | 0,075193 | apoptosis |
| ENSG00000126524 | TIAL1 | -0,06491 | -0,50759 | 0,274613 | apoptosis |
| ENSG00000168385 | TMEM173 | -0,12359 | -0,45058 | 0,188147 | apoptosis |
| ENSG00000111897 | TMEM85 | 0,92733 | 0,987022 | 0,679223 | apoptosis |
| ENSG00000107290 | TMX1 | -0,13274 | 0,160064 | -0,0811 | apoptosis |
| ENSG00000183431 | TNFAIP3 | -0,53885 | -0,48525 | 1,760881 | apoptosis |
| ENSG00000115524 | TNFRSF12A | 0,411636 | 0,206564 | 0,988535 | apoptosis |
| ENSG00000169976 | TNFRSF18 | -0,14766 | -0,33642 | 0,005053 | apoptosis |
| ENSG00000198089 | TNFRSF1A | -0,41858 | -0,41417 | 0,322465 | apoptosis |
| ENSG00000116560 | TNFRSF1B | -4,41574 | -4,20394 | -3,90817 | apoptosis |
| ENSG00000136450 | TNFRSF25 | 0,707273 | 0,3719 | 0,96923 | apoptosis |
| ENSG00000116754 | TNFRSF4 | -0,11847 | -0,26804 | 0,32678 | apoptosis |
| ENSG00000064607 | TNFSF8 | -1,64945 | -1,50688 | 0,39284 | apoptosis |
| ENSG00000148341 | TP53 | 1,540879 | 1,614751 | 0,599299 | apoptosis |
| ENSG00000147010 | TP53BP2 | 0,30671 | 0,265172 | 0,792671 | apoptosis |
| ENSG00000107338 | TP53INP1 | 1,863642 | -0,26417 | 0,988881 | apoptosis |
| ENSG00000142178 | TPT1 | 0,737078 | 0,578504 | 1,186176 | apoptosis |
| ENSG00000160584 | TPX2 | 0,145378 | 0,872411 | 0,583284 | apoptosis |
| ENSG00000105738 | TRAF2 | -0,18352 | -0,10462 | 0,134143 | apoptosis |
| ENSG00000198053 | TRAF6 | 0,005267 | -0,52772 | 0,376704 | apoptosis |
| ENSG00000101307 | TRAF7 | 0,917669 | 0,742206 | 0,586289 | apoptosis |
| ENSG00000140199 | TRIM35 | -0,052 | -0,20029 | 0,024036 | apoptosis |
| ENSG00000181035 | TSPO | 1,48347 | 1,803591 | 3,162573 | apoptosis |
| ENSG00000139613 | UBA52 | 2,730905 | 2,673263 | 2,979033 | apoptosis |
| ENSG00000198887 | UBB | 4,588115 | 4,657338 | 5,213323 | apoptosis |
| ENSG00000196787 | UBC | 5,120515 | 4,831483 | 5,873363 | apoptosis |
| ENSG00000104976 | UBE2Z | 0,326131 | 0,370039 | 0,413484 | apoptosis |
| ENSG00000197157 | UBE4B | 0,241935 | 0,129989 | 0,746908 | apoptosis |
| ENSG00000207233 | UBQLN1 | 1,520216 | 1,323566 | 1,973455 | apoptosis |
| ENSG00000100603 | USP7 | 0,767803 | 1,069808 | 2,939913 | apoptosis |
| ENSG00000130340 | VAV1 | -1,14382 | -0,91728 | 0,384009 | apoptosis |
| ENSG00000159140 | VCP | 1,44419 | 1,6991 | 1,847994 | apoptosis |
| ENSG00000115904 | VEGFA | -0,97489 | -0,62991 | 0,206981 | apoptosis |
| ENSG00000134595 | VIM | 1,041998 | 1,617704 | 4,327433 | apoptosis |
| ENSG00000110693 | VNN1 | -2,57556 | -2,0549 | 0,394496 | apoptosis |
| ENSG00000105866 | VPS24 | -0,25663 | -0,0259 | 0,39173 | apoptosis |
| ENSG00000138385 | WRN | 0,833839 | 0,453612 | 0,861978 | apoptosis |
| ENSG00000136738 | YARS | 0,372833 | 0,028811 | -0,18955 | apoptosis |
| ENSG00000167323 | YWHAB | 1,294485 | 1,656333 | 2,169249 | apoptosis |
| ENSG00000168439 | YWHAE | 2,514555 | 2,513953 | 1,73775 | apoptosis |
| ENSG00000148175 | ZBTB16 | 0,03977 | -0,27793 | 0,138939 | apoptosis |
| ENSG00000104915 | ZC3H12A | -0,35679 | -0,19938 | 0,959631 | apoptosis |
| ENSG00000200649 | AAAS | 0,514471 | 0,751675 | 0,341378 | intracellular transport |
| ENSG00000201812 | ABCA13 | -2,0543 | -1,31373 | 1,941409 | intracellular transport |
| ENSG00000202092 | ABCB10 | 1,064352 | 1,87897 | 2,404745 | intracellular transport |
| ENSG00000202248 | ABCB7 | -0,67568 | -0,46731 | 0,232039 | intracellular transport |
| ENSG00000212265 | ABCB8 | -0,08928 | 0,053151 | -0,24369 | intracellular transport |
| ENSG00000212499 | ABCC1 | 0,38322 | -0,02619 | 0,33209 | intracellular transport |
| ENSG00000222208 | ABCC4 | -0,5776 | -0,37135 | 0,292002 | intracellular transport |
| ENSG00000199585 | ABCC5 | -0,66821 | 0,057164 | -0,38959 | intracellular transport |
| ENSG00000180610 | ACBD3 | 0,058735 | -0,36102 | 0,33589 | intracellular transport |
| ENSG00000239935 | ADD2 | 1,916837 | 2,801753 | 1,385947 | intracellular transport |
| ENSG00000185294 | AGFG1 | 1,806102 | 1,894934 | 2,983283 | intracellular transport |
| ENSG00000181786 | AKT1 | 1,744731 | 1,7493 | 1,843017 | intracellular transport |
| ENSG00000130402 | AKT2 | 0,845838 | 1,114623 | 1,466699 | intracellular transport |
| ENSG00000242597 | ANKH | -0,04696 | 0,260566 | 0,532317 | intracellular transport |
| ENSG00000242275 | ANO10 | -1,20191 | -0,71536 | 0,328976 | intracellular transport |
| ENSG00000006534 | AP1B1 | 1,009712 | 1,053115 | 0,739508 | intracellular transport |
| ENSG00000149925 | AP1G1 | 0,833657 | 0,480922 | 1,764698 | intracellular transport |
| ENSG00000182858 | AP1G2 | -0,07682 | -0,17588 | 0,042247 | intracellular transport |
| ENSG00000091542 | AP1M1 | -0,21895 | -0,22314 | 0,670692 | intracellular transport |
| ENSG00000012779 | AP2A1 | 1,226376 | 1,889858 | 2,397944 | intracellular transport |
| ENSG00000132965 | AP2B1 | 0,656444 | 1,387529 | 1,374814 | intracellular transport |
| ENSG00000163295 | AP2M1 | 1,167 | 1,457802 | 1,605599 | intracellular transport |
| ENSG00000163286 | AP2S1 | 0,870725 | 1,091071 | 1,155227 | intracellular transport |
| ENSG00000155749 | AP3D1 | 1,098045 | 1,300829 | 1,122697 | intracellular transport |
| ENSG00000110497 | AP3S1 | -0,12298 | -0,01228 | 1,847352 | intracellular transport |
| ENSG00000123505 | AP3S2 | -0,06276 | -0,13399 | 0,564534 | intracellular transport |
| ENSG00000089053 | APOC1 | 1,053643 | 0,108114 | -0,77566 | intracellular transport |
| ENSG00000089053 | AQP1 | -0,60447 | 0,526029 | 1,980077 | intracellular transport |
| ENSG00000089053 | AQP3 | -0,66735 | -0,41735 | 0,987632 | intracellular transport |
| ENSG00000089053 | AQP9 | -2,23794 | -1,8757 | 0,533126 | intracellular transport |
| ENSG00000064999 | ARHGEF2 | 0,277696 | 0,558861 | 1,424291 | intracellular transport |
| ENSG00000128383 | ASNA1 | 1,153345 | 1,412191 | 1,309607 | intracellular transport |
| ENSG00000244509 | ASPSCR1 | 0,26717 | 0,227953 | 0,543834 | intracellular transport |
| ENSG00000143761 | ATOX1 | -0,08206 | -0,26577 | 0,1681 | intracellular transport |
| ENSG00000143761 | ATP11B | 0,032482 | -0,21264 | 1,335572 | intracellular transport |
| ENSG00000143761 | ATP1B2 | 0,211138 | 0,88431 | 1,094578 | intracellular transport |
| ENSG00000143761 | ATP2A2 | 0,762305 | 0,207232 | 0,192807 | intracellular transport |
| ENSG00000143761 | ATP2A3 | -0,37413 | -0,05989 | 1,944912 | intracellular transport |
| ENSG00000143761 | ATP2B4 | 0,407696 | 1,177625 | 0,528868 | intracellular transport |
| ENSG00000134287 | ATP5A1 | 1,410902 | 1,390718 | 0,492555 | intracellular transport |
| ENSG00000134287 | ATP5B | 2,648765 | 2,491138 | 2,225232 | intracellular transport |
| ENSG00000134287 | ATP5C1 | 0,759726 | 0,645546 | 0,067222 | intracellular transport |
| ENSG00000134287 | ATP5D | 1,228063 | 1,351332 | 1,282373 | intracellular transport |
| ENSG00000134287 | ATP5E | 0,629059 | 0,728904 | 0,47356 | intracellular transport |
| ENSG00000134287 | ATP5EP2 | 0,954788 | 0,909038 | 0,725725 | intracellular transport |
| ENSG00000134287 | ATP5F1 | 0,855353 | 0,699196 | 0,723771 | intracellular transport |
| ENSG00000134287 | ATP5G1 | 3,042195 | 3,146863 | 1,211637 | intracellular transport |
| ENSG00000168374 | ATP5G2 | 1,571351 | 1,233949 | 1,325645 | intracellular transport |
| ENSG00000004059 | ATP5H | 0,884735 | 0,871697 | 0,826748 | intracellular transport |
| ENSG00000165527 | ATP5I | 0,987092 | 0,978266 | 0,826565 | intracellular transport |
| ENSG00000101199 | ATP5J2 | 1,261065 | 1,086918 | 0,973626 | intracellular transport |
| ENSG00000149182 | ATP5L | 1,10603 | 0,979927 | 1,09232 | intracellular transport |
| ENSG00000066777 | ATP5O | 1,635497 | 1,39386 | 0,983243 | intracellular transport |
| ENSG00000134884 | ATP6AP1 | 1,140433 | 1,119582 | 2,146997 | intracellular transport |
| ENSG00000187951 | ATP6V0A1 | -0,19945 | 0,752059 | 2,221426 | intracellular transport |
| ENSG00000075884 | ATP6V0B | -0,64926 | 0,093235 | 2,16316 | intracellular transport |
| ENSG00000213390 | ATP6V0C | 2,613645 | 2,856068 | 3,727933 | intracellular transport |
| ENSG00000225485 | ATP6V0D1 | 0,812819 | 1,554646 | 2,221935 | intracellular transport |
| ENSG00000225485 | ATP6V0E1 | -0,53148 | 0,671495 | 1,105413 | intracellular transport |
| ENSG00000145819 | ATP6V0E2 | -0,12928 | -0,28042 | 0,094801 | intracellular transport |
| ENSG00000186517 | ATP6V1B2 | 0,176324 | 0,342704 | 1,404466 | intracellular transport |
| ENSG00000089820 | ATP6V1E1 | 0,215027 | -0,11625 | 0,257189 | intracellular transport |
| ENSG00000123329 | ATP6V1F | 1,174345 | 1,154549 | 1,619964 | intracellular transport |
| ENSG00000141522 | ATP6V1G1 | 1,638073 | 1,160542 | 1,784083 | intracellular transport |
| ENSG00000100325 | BCAP31 | -0,20421 | -0,28482 | 0,320774 | intracellular transport |
| ENSG00000144848 | BICD2 | -0,56638 | -0,41911 | 0,013116 | intracellular transport |
| ENSG00000104043 | C11orf2 | 1,028609 | 1,009835 | 1,480223 | intracellular transport |
| ENSG00000099840 | CA2 | 2,722795 | 3,375063 | 3,206753 | intracellular transport |
| ENSG00000105700 | CACNG8 | 0,301374 | 0,253597 | 0,760294 | intracellular transport |
| ENSG00000160679 | CASC3 | 0,734485 | 0,498638 | 1,124382 | intracellular transport |
| ENSG00000173769 | CD36 | 1,447629 | 2,079766 | 1,79 | intracellular transport |
| ENSG00000112308 | CD74 | 2,334515 | 1,855249 | 3,374823 | intracellular transport |
| ENSG00000077549 | CHKA | 0,389236 | -0,14491 | 1,089945 | intracellular transport |
| ENSG00000198624 | CLCN3 | -1,42323 | -1,06672 | 0,970733 | intracellular transport |
| ENSG00000186166 | CLCN6 | -0,55766 | -0,51537 | 0,202705 | intracellular transport |
| ENSG00000186166 | CLCN7 | 0,229917 | 0,205176 | 0,517606 | intracellular transport |
| ENSG00000134057 | CLIC1 | 1,954507 | 1,82936 | 2,747553 | intracellular transport |
| ENSG00000157456 | CLIC4 | 0,962842 | 0,63525 | 0,754271 | intracellular transport |
| ENSG00000135083 | CLN3 | -0,32254 | -0,39398 | 0,111373 | intracellular transport |
| ENSG00000163660 | CLN8 | -0,80243 | -1,14025 | 0,080366 | intracellular transport |
| ENSG00000082258 | CLTA | -0,05339 | 0,146293 | -0,02393 | intracellular transport |
| ENSG00000108100 | CLTB | 0,757189 | 0,734865 | 1,288443 | intracellular transport |
| ENSG00000126353 | CLTC | 1,588052 | 1,855365 | 2,076391 | intracellular transport |
| ENSG00000151014 | CLTCL1 | -1,56052 | -1,44192 | 0,163322 | intracellular transport |
| ENSG00000135535 | CNNM2 | -0,09426 | 0,100555 | 0,014727 | intracellular transport |
| ENSG00000110848 | COL4A3BP | -0,90944 | -0,89378 | 0,444541 | intracellular transport |
| ENSG00000140326 | COPA | 1,391149 | 1,181891 | 1,437304 | intracellular transport |
| ENSG00000079335 | COPG | 0,434987 | 0,519982 | 0,471962 | intracellular transport |
| ENSG00000135446 | CPT1A | 1,259766 | 1,277313 | 1,180012 | intracellular transport |
| ENSG00000176749 | CPT1B | -0,27506 | -0,24273 | 0,017656 | intracellular transport |
| ENSG00000108465 | CRAT | -0,20526 | -0,09486 | 0,189538 | intracellular transport |
| ENSG00000100162 | CSE1L | 0,476326 | 0,600227 | -0,08929 | intracellular transport |
| ENSG00000111666 | CTSA | 1,021083 | 1,403701 | 2,420642 | intracellular transport |
| ENSG00000179862 | CUX1 | 0,841667 | 0,472921 | 0,882495 | intracellular transport |
| ENSG00000175216 | CYB5A | 0,075696 | -0,02698 | -0,59211 | intracellular transport |
| ENSG00000109572 | CYC1 | 1,121475 | 1,440033 | 1,00772 | intracellular transport |
| ENSG00000111605 | DIRC2 | -2,11175 | -1,12762 | 1,140287 | intracellular transport |
| ENSG00000164733 | DYNC1H1 | 1,295057 | 0,942178 | 0,659223 | intracellular transport |
| ENSG00000117984 | DYNC1LI2 | -0,29356 | -0,75745 | 0,06638 | intracellular transport |
| ENSG00000196188 | DYNLL1 | -0,09546 | 0,07288 | -0,82347 | intracellular transport |
| ENSG00000100448 | DYNLL2 | 1,567453 | 1,270732 | 1,571947 | intracellular transport |
| ENSG00000135047 | DYNLRB1 | 0,743738 | 0,224627 | 0,369618 | intracellular transport |
| ENSG00000100014 | EHD1 | -0,56298 | -0,49449 | 0,672156 | intracellular transport |
| ENSG00000179958 | EIF4A3 | 0,607979 | 0,794265 | 0,935664 | intracellular transport |
| ENSG00000108654 | ENY2 | -0,0046 | -0,10439 | 0,303654 | intracellular transport |
| ENSG00000149091 | ERO1L | -1,89065 | -1,69303 | 0,734189 | intracellular transport |
| ENSG00000108272 | ERP29 | 0,184268 | 0,418245 | 0,630483 | intracellular transport |
| ENSG00000100697 | ETFB | -0,02719 | 0,06507 | -0,11392 | intracellular transport |
| ENSG00000213551 | FADS2 | 2,051675 | 1,865706 | -0,46029 | intracellular transport |
| ENSG00000183403 | FADS3 | 0,426916 | -0,07889 | -0,10076 | intracellular transport |
| ENSG00000141627 | FAM38A | 1,607 | 1,615081 | 0,743236 | intracellular transport |
| ENSG00000175390 | FKBP15 | -0,38769 | -0,02001 | 0,079638 | intracellular transport |
| ENSG00000102119 | FXN | 2,153658 | 1,963684 | 0,976789 | intracellular transport |
| ENSG00000186998 | FXYD5 | 1,070138 | 0,417032 | 1,473569 | intracellular transport |
| ENSG00000138185 | G3BP1 | 1,961153 | 1,751842 | 0,999124 | intracellular transport |
| ENSG00000197217 | G3BP2 | -0,04314 | -0,14778 | 0,20391 | intracellular transport |
| ENSG00000185862 | GGA1 | -0,02523 | -0,1702 | 0,14089 | intracellular transport |
| ENSG00000196405 | GGA2 | 0,618639 | 0,714154 | 0,850681 | intracellular transport |
| ENSG00000167880 | GGA3 | 0,37577 | 0,301775 | 0,649221 | intracellular transport |
| ENSG00000134824 | GLRX | 0,511747 | 0,658459 | 0,051312 | intracellular transport |
| ENSG00000103876 | GLTP | -0,83235 | -0,88555 | 0,143993 | intracellular transport |
| ENSG00000185519 | GOLIM4 | 0,233498 | 0,123082 | -1,1122 | intracellular transport |
| ENSG00000189292 | GOT2 | 1,623185 | 1,636472 | 0,829975 | intracellular transport |
| ENSG00000148343 | GRIN3B | 0,107055 | -0,27234 | 0,189084 | intracellular transport |
| ENSG00000115641 | HBD | 3,093385 | 3,194913 | 4,372023 | intracellular transport |
| ENSG00000183386 | HBM | -0,4826 | 1,156735 | 3,527333 | intracellular transport |
| ENSG00000145216 | HBQ1 | 0,866723 | 0,654717 | 1,353351 | intracellular transport |
| ENSG00000162076 | HDAC6 | -0,1784 | -0,20888 | 0,382153 | intracellular transport |
| ENSG00000187239 | HDLBP | 1,838399 | 1,796049 | 1,787915 | intracellular transport |
| ENSG00000075426 | HERC1 | 0,511558 | 0,14033 | 2,703283 | intracellular transport |
| ENSG00000164916 | HGS | 0,511575 | 0,642715 | 1,158253 | intracellular transport |
| ENSG00000139112 | HK1 | 0,064225 | 0,501201 | 0,523355 | intracellular transport |
| ENSG00000034713 | HK2 | -0,59847 | -0,76009 | 0,855401 | intracellular transport |
| ENSG00000204681 | HK3 | -1,51912 | -1,45259 | 0,731748 | intracellular transport |
| ENSG00000148288 | HNRNPA1 | -1,68402 | -1,02078 | -1,44357 | intracellular transport |
| ENSG00000143774 | IPO4 | 0,989534 | 1,006333 | 0,02273 | intracellular transport |
| ENSG00000143774 | IPO5 | 1,020034 | 0,818817 | 0,321374 | intracellular transport |
| ENSG00000143774 | IPO7 | 0,482683 | 0,510289 | -0,22301 | intracellular transport |
| ENSG00000143774 | IPO9 | -0,19402 | 0,446269 | 0,125967 | intracellular transport |
| ENSG00000169567 | KCNAB2 | -0,55223 | -0,63887 | 1,737942 | intracellular transport |
| ENSG00000169567 | KCNC1 | -0,05814 | -0,20618 | 0,054905 | intracellular transport |
| ENSG00000169567 | KCNC3 | -0,35817 | -0,50426 | 0,04467 | intracellular transport |
| ENSG00000169567 | KCNG2 | 0,032609 | -0,04855 | 0,334042 | intracellular transport |
| ENSG00000169567 | KCNH2 | 1,903053 | 2,145131 | 0,618575 | intracellular transport |
| ENSG00000137133 | KCNJ12 | 0,113639 | -0,09039 | 0,220326 | intracellular transport |
| ENSG00000127946 | KCNK12 | -0,18765 | -0,0862 | 0,056152 | intracellular transport |
| ENSG00000163349 | KCNK15 | 0,081039 | -0,14369 | 0,429487 | intracellular transport |
| ENSG00000064393 | KCNN4 | -0,15229 | 0,100442 | 0,116121 | intracellular transport |
| ENSG00000137259 | KDELR2 | -0,40092 | 0,45932 | 0,338369 | intracellular transport |
| ENSG00000197061 | KHSRP | 1,588595 | 1,483525 | 0,939855 | intracellular transport |
| ENSG00000177733 | KIF13A | 0,701635 | -0,24969 | -0,17587 | intracellular transport |
| ENSG00000135486 | KIF1B | -1,33452 | -1,44043 | 0,333004 | intracellular transport |
| ENSG00000169045 | KPNA1 | -0,63512 | -0,29379 | 0,254994 | intracellular transport |
| ENSG00000126945 | KPNA2 | 1,391184 | 1,682661 | 1,623127 | intracellular transport |
| ENSG00000096746 | KPNA3 | 0,052518 | -0,0188 | -0,26812 | intracellular transport |
| ENSG00000165119 | KPNA6 | 0,588505 | 0,529508 | 1,215213 | intracellular transport |
| ENSG00000152795 | LAPTM4A | 2,401685 | 1,683888 | 2,805353 | intracellular transport |
| ENSG00000103942 | LAPTM5 | 2,419755 | 2,367668 | 4,493093 | intracellular transport |
| ENSG00000127483 | LASP1 | 0,669546 | 0,89005 | 2,152189 | intracellular transport |
| ENSG00000199090 | LCN2 | 1,45788 | 2,190947 | 4,837443 | intracellular transport |
| ENSG00000214706 | LRP10 | -0,28186 | 0,139702 | 2,729033 | intracellular transport |
| ENSG00000197081 | LRPPRC | 0,827049 | 0,433306 | -0,91199 | intracellular transport |
| ENSG00000141753 | LRRC26 | 0,875282 | 0,750088 | 1,09205 | intracellular transport |
| ENSG00000143621 | LTF | 0,986328 | 2,287191 | 5,094793 | intracellular transport |
| ENSG00000149503 | M6PR | 0,461652 | 0,647402 | -0,02159 | intracellular transport |
| ENSG00000178342 | MFSD10 | -0,02161 | 0,128508 | 1,956124 | intracellular transport |
| ENSG00000055118 | MFSD2B | -0,28083 | -0,21123 | 0,922339 | intracellular transport |
| ENSG00000121774 | MLC1 | 1,538703 | 1,042417 | 0,368968 | intracellular transport |
| ENSG00000105610 | MRPL45 | 1,305515 | 1,507031 | 0,315348 | intracellular transport |
| ENSG00000221864 | MTCH1 | 0,349596 | 0,834738 | 1,884286 | intracellular transport |
| ENSG00000212933 | MTCH2 | 0,133048 | 0,369319 | 0,221061 | intracellular transport |
| ENSG00000121897 | NAPA | 0,275328 | 0,614521 | 1,42629 | intracellular transport |
| ENSG00000169223 | NCBP1 | 0,408811 | 0,306647 | 0,242028 | intracellular transport |
| ENSG00000123684 | NDEL1 | 1,047764 | 0,723226 | 2,430355 | intracellular transport |
| ENSG00000171236 | NDUFA10 | 0,351358 | 0,346853 | 0,050113 | intracellular transport |
| ENSG00000198799 | NDUFA12 | 0,584111 | 0,340766 | 0,228029 | intracellular transport |
| ENSG00000118308 | NDUFA2 | 1,593479 | 1,982222 | 1,625772 | intracellular transport |
| ENSG00000197324 | NDUFA3 | 1,372522 | 1,099667 | 1,212728 | intracellular transport |
| ENSG00000130881 | NDUFA6 | 0,724493 | 0,993385 | 0,896746 | intracellular transport |
| ENSG00000157193 | NDUFA7 | 0,961973 | 0,7532 | 0,886435 | intracellular transport |
| ENSG00000163956 | NDUFA9 | 0,115586 | 0,251834 | 0,401898 | intracellular transport |
| ENSG00000177021 | NDUFB11 | 2,260779 | 2,139431 | 1,697462 | intracellular transport |
| ENSG00000175489 | NDUFB7 | 0,706287 | 0,421161 | 0,76606 | intracellular transport |
| ENSG00000184709 | NDUFB8 | 1,68417 | 1,688839 | 1,223056 | intracellular transport |
| ENSG00000174004 | NDUFB9 | 1,176436 | 1,29252 | 0,693958 | intracellular transport |
| ENSG00000185158 | NDUFC2 | 1,086789 | 1,291449 | 0,661048 | intracellular transport |
| ENSG00000132128 | NDUFS2 | 0,40684 | 0,203542 | 0,654889 | intracellular transport |
| ENSG00000116212 | NDUFS3 | 0,500228 | 0,382887 | 0,302493 | intracellular transport |
| ENSG00000130764 | NDUFS5 | 2,975805 | 2,580988 | 1,394329 | intracellular transport |
| ENSG00000180979 | NDUFS6 | 0,186616 | 0,010406 | 0,005953 | intracellular transport |
| ENSG00000163428 | NDUFS7 | 0,673187 | 0,811882 | 0,77879 | intracellular transport |
| ENSG00000108829 | NDUFS8 | 0,914866 | 0,68611 | 0,791149 | intracellular transport |
| ENSG00000104866 | NDUFV1 | 1,379462 | 1,458776 | 0,794606 | intracellular transport |
| ENSG00000136802 | NDUFV3 | 0,896884 | 1,018359 | 1,023185 | intracellular transport |
| ENSG00000034152 | NNT | -0,1371 | 0,281206 | 0,205411 | intracellular transport |
| ENSG00000101367 | NPM1 | -1,54685 | -1,66528 | -1,30858 | intracellular transport |
| ENSG00000198625 | NUP153 | 1,631077 | 1,125477 | 1,668134 | intracellular transport |
| ENSG00000112159 | NUP155 | 0,258202 | 0,244464 | -0,03279 | intracellular transport |
| ENSG00000151376 | NUP188 | 0,388135 | 0,608854 | -0,01737 | intracellular transport |
| ENSG00000124733 | NUP210 | 1,947645 | 2,167353 | 1,336694 | intracellular transport |
| ENSG00000169057 | NUP214 | 1,36269 | 1,173023 | 1,312334 | intracellular transport |
| ENSG00000125686 | NUP43 | 0,188264 | 0,330487 | -0,80236 | intracellular transport |
| ENSG00000184634 | NUP50 | -0,80769 | -0,69665 | 0,136984 | intracellular transport |
| ENSG00000108510 | NUP54 | 0,055795 | -0,39548 | -0,71648 | intracellular transport |
| ENSG00000123066 | NUP85 | 1,226301 | 1,041264 | 0,595631 | intracellular transport |
| ENSG00000180182 | NUP88 | 1,238035 | 1,037386 | 1,578455 | intracellular transport |
| ENSG00000099917 | NUP93 | 1,141312 | 1,022695 | 0,321576 | intracellular transport |
| ENSG00000175221 | NUP98 | 1,152875 | 0,943034 | 1,949397 | intracellular transport |
| ENSG00000152944 | NUPL1 | 0,204015 | -0,00465 | 0,160986 | intracellular transport |
| ENSG00000008838 | NUTF2 | 2,771885 | 2,621853 | 2,2632 | intracellular transport |
| ENSG00000104973 | NXF1 | 1,847462 | 1,595601 | 2,692623 | intracellular transport |
| ENSG00000100060 | ORAI1 | 0,600351 | 0,305652 | 0,825127 | intracellular transport |
| ENSG00000151690 | ORM1 | -2,67334 | -2,15477 | 0,256297 | intracellular transport |
| ENSG00000179087 | OSBP | 0,565777 | 0,046101 | 0,524076 | intracellular transport |
| ENSG00000128268 | OSBP2 | -1,01027 | -0,232 | 1,386015 | intracellular transport |
| ENSG00000161013 | OSBPL2 | -0,92945 | -0,50724 | 0,641305 | intracellular transport |
| ENSG00000198408 | OSBPL8 | -0,25269 | -0,02598 | 0,384494 | intracellular transport |
| ENSG00000102858 | OSBPL9 | 0,897014 | 0,554896 | 1,106136 | intracellular transport |
| ENSG00000141503 | P2RX1 | -0,18116 | -0,63936 | 1,387692 | intracellular transport |
| ENSG00000100427 | PACSIN2 | 0,044295 | 0,044778 | 0,361352 | intracellular transport |
| ENSG00000168288 | PANX2 | 0,382833 | 0,072321 | 0,521876 | intracellular transport |
| ENSG00000130675 | PARP4 | 0,69475 | 0,302467 | 1,069393 | intracellular transport |
| ENSG00000186732 | PCNA | 0,942073 | 1,465449 | 0,787595 | intracellular transport |
| ENSG00000166902 | PCTP | -0,69451 | -0,52237 | 0,267627 | intracellular transport |
| ENSG00000136504 | PITPNA | 0,650854 | 1,044585 | 1,100761 | intracellular transport |
| ENSG00000136504 | PITPNM1 | -1,01326 | -1,21031 | 0,466702 | intracellular transport |
| ENSG00000134440 | PLP2 | 1,576838 | 1,79282 | 3,398393 | intracellular transport |
| ENSG00000115216 | PSAP | 0,795106 | 1,084593 | 2,040301 | intracellular transport |
| ENSG00000164329 | RACGAP1 | -0,05123 | 0,629578 | 0,573696 | intracellular transport |
| ENSG00000178685 | RAMP1 | 0,431256 | 0,252724 | 0,712037 | intracellular transport |
| ENSG00000059378 | RAN | 1,30845 | 1,361538 | 1,097822 | intracellular transport |
| ENSG00000089220 | RBM8A | 0,422869 | 0,646854 | 0,46506 | intracellular transport |
| ENSG00000133027 | RBP5 | -0,78447 | -1,0426 | 0,10531 | intracellular transport |
| ENSG00000130024 | RFFL | 0,025995 | 0,509045 | 2,243988 | intracellular transport |
| ENSG00000077684 | RFT1 | -0,76362 | -0,97792 | 0,011983 | intracellular transport |
| ENSG00000102893 | RHAG | 1,319807 | 1,916621 | 3,344773 | intracellular transport |
| ENSG00000164244 | SAR1A | 1,782811 | 1,432488 | 2,268158 | intracellular transport |
| ENSG00000013275 | SDHA | 2,401545 | 2,374158 | 1,647689 | intracellular transport |
| ENSG00000173692 | SDHB | 0,495523 | 0,496895 | 0,378836 | intracellular transport |
| ENSG00000108344 | SEC13 | 0,273425 | 0,154361 | 0,247931 | intracellular transport |
| ENSG00000103035 | SEC14L1 | -1,12072 | -0,78066 | 0,994938 | intracellular transport |
| ENSG00000099341 | SEC14L4 | -0,29897 | 0,723473 | 1,032987 | intracellular transport |
| ENSG00000068878 | SEC24A | -0,84742 | -0,6429 | 0,03622 | intracellular transport |
| ENSG00000125818 | SEC24B | 0,351039 | 0,263025 | 0,662986 | intracellular transport |
| ENSG00000183527 | SEC24C | -0,14641 | -0,54315 | 0,101161 | intracellular transport |
| ENSG00000188647 | SEH1L | 0,277839 | -0,14677 | -0,98123 | intracellular transport |
| ENSG00000144118 | SIGMAR1 | 1,436099 | 1,625491 | 1,097407 | intracellular transport |
| ENSG00000173976 | SLC10A3 | 0,818552 | 0,510375 | 0,986694 | intracellular transport |
| ENSG00000023287 | SLC11A1 | -1,02402 | -0,82734 | 0,740286 | intracellular transport |
| ENSG00000146587 | SLC11A2 | -0,05335 | 0,704304 | 1,590904 | intracellular transport |
| ENSG00000162521 | SLC12A4 | -0,20006 | 0,366282 | -0,70249 | intracellular transport |
| ENSG00000122257 | SLC12A6 | 2,265769 | 1,345517 | 1,539914 | intracellular transport |
| ENSG00000102054 | SLC12A7 | -1,01214 | -0,49269 | 0,422349 | intracellular transport |
| ENSG00000080839 | SLC15A4 | -0,80885 | -0,72211 | 1,976258 | intracellular transport |
| ENSG00000213079 | SLC19A1 | 0,494319 | 0,366406 | 0,777835 | intracellular transport |
| ENSG00000119446 | SLC1A5 | -0,5065 | 0,107216 | 1,643874 | intracellular transport |
| ENSG00000122965 | SLC20A1 | 0,110789 | 0,464574 | 1,095106 | intracellular transport |
| ENSG00000086589 | SLC22A15 | -1,97858 | -1,93266 | 0,293366 | intracellular transport |
| ENSG00000100461 | SLC22A16 | -1,15152 | -0,50152 | 0,15621 | intracellular transport |
| ENSG00000119707 | SLC22A17 | -0,31381 | -0,57366 | 0,02649 | intracellular transport |
| ENSG00000139746 | SLC22A23 | -0,15916 | 0,240126 | 1,956784 | intracellular transport |
| ENSG00000102317 | SLC25A1 | 0,482487 | 0,296475 | 0,120318 | intracellular transport |
| ENSG00000184863 | SLC25A11 | 0,653006 | 0,932578 | 0,820157 | intracellular transport |
| ENSG00000132819 | SLC25A28 | -0,02012 | -0,00266 | 0,541381 | intracellular transport |
| ENSG00000131051 | SLC25A29 | 0,115711 | -0,18263 | 0,321449 | intracellular transport |
| ENSG00000173933 | SLC25A3 | 3,157035 | 2,983933 | 2,972593 | intracellular transport |
| ENSG00000126254 | SLC25A36 | -0,0495 | -0,37588 | 0,21858 | intracellular transport |
| ENSG00000173914 | SLC25A37 | 1,614622 | 2,623533 | 4,612743 | intracellular transport |
| ENSG00000003756 | SLC25A38 | 0,008809 | 0,00433 | 0,322055 | intracellular transport |
| ENSG00000131795 | SLC25A39 | 1,873131 | 2,721703 | 4,257323 | intracellular transport |
| ENSG00000170748 | SLC25A5 | 2,835955 | 2,654373 | 2,491889 | intracellular transport |
| ENSG00000166831 | SLC26A6 | -1,11568 | -0,6618 | 0,226346 | intracellular transport |
| ENSG00000168214 | SLC29A1 | 1,392746 | 1,821041 | 1,345108 | intracellular transport |
| ENSG00000135870 | SLC2A1 | 0,961291 | 2,512187 | 5,284353 | intracellular transport |
| ENSG00000179051 | SLC2A3 | -0,25878 | 0,380177 | 2,646583 | intracellular transport |
| ENSG00000120158 | SLC2A4 | -0,51434 | -0,35094 | 1,623122 | intracellular transport |
| ENSG00000204356 | SLC31A1 | 0,083536 | 1,129572 | 0,839923 | intracellular transport |
| ENSG00000100918 | SLC31A2 | -1,40697 | -1,42706 | 0,998073 | intracellular transport |
| ENSG00000160957 | SLC35A4 | 0,140832 | 0,294047 | -0,07599 | intracellular transport |
| ENSG00000168476 | SLC35B1 | 0,199381 | 0,101649 | 0,472857 | intracellular transport |
| ENSG00000129625 | SLC35C2 | -0,84517 | -0,39476 | 0,323454 | intracellular transport |
| ENSG00000162924 | SLC35E1 | -0,08805 | -0,11445 | 0,562869 | intracellular transport |
| ENSG00000173039 | SLC36A1 | -0,91935 | -0,31402 | 1,432275 | intracellular transport |
| ENSG00000104856 | SLC37A4 | 0,200044 | 0,883384 | 0,377441 | intracellular transport |
| ENSG00000181826 | SLC38A1 | -0,5341 | -0,43415 | 0,804322 | intracellular transport |
| ENSG00000054967 | SLC38A10 | 0,318759 | 0,306374 | 0,873768 | intracellular transport |
| ENSG00000214022 | SLC38A2 | 1,010319 | 1,021089 | 2,162311 | intracellular transport |
| ENSG00000157916 | SLC38A5 | -0,13537 | 0,594315 | 0,34862 | intracellular transport |
| ENSG00000084093 | SLC39A8 | 1,90434 | 1,654317 | 1,016647 | intracellular transport |
| ENSG00000104918 | SLC39A9 | -0,41697 | 0,093722 | 0,112023 | intracellular transport |
| ENSG00000079313 | SLC3A2 | 1,115888 | 1,063204 | 1,431069 | intracellular transport |
| ENSG00000076043 | SLC40A1 | 1,300528 | 0,951146 | 1,607948 | intracellular transport |
| ENSG00000163918 | SLC43A1 | 0,990793 | 2,039228 | 1,853918 | intracellular transport |
| ENSG00000092871 | SLC43A2 | -0,76743 | -1,04908 | 0,165576 | intracellular transport |
| ENSG00000168411 | SLC48A1 | 0,057072 | 0,358267 | 0,265571 | intracellular transport |
| ENSG00000181827 | SLC4A2 | -0,04063 | 0,034935 | 0,303053 | intracellular transport |
| ENSG00000064490 | SLC4A3 | -0,48193 | -0,53393 | 0,138939 | intracellular transport |
| ENSG00000148908 | SLC5A3 | -0,34568 | 0,153534 | -0,93852 | intracellular transport |
| ENSG00000169220 | SLC6A6 | -1,08606 | -1,26219 | 0,881688 | intracellular transport |
| ENSG00000171700 | SLC6A8 | 1,192583 | 1,327112 | 2,191439 | intracellular transport |
| ENSG00000116741 | SLC6A9 | -0,4903 | -0,47406 | 1,58475 | intracellular transport |
| ENSG00000112077 | SLC7A1 | -0,1778 | 0,748818 | 0,833358 | intracellular transport |
| ENSG00000144468 | SLC7A5 | -0,03973 | -0,14987 | 3,187873 | intracellular transport |
| ENSG00000005486 | SLC9A1 | -1,24389 | -1,3217 | 0,161989 | intracellular transport |
| ENSG00000103269 | SLC9A8 | -0,927 | -0,87202 | 0,720037 | intracellular transport |
| ENSG00000188672 | SLCO3A1 | -1,30187 | -1,03409 | 0,934552 | intracellular transport |
| ENSG00000187010 | SLCO4C1 | -2,49273 | -2,49433 | 0,132188 | intracellular transport |
| ENSG00000067560 | SMAD3 | 0,18712 | -0,24852 | -0,31358 | intracellular transport |
| ENSG00000237872 | SNX17 | 0,80289 | 0,342225 | 0,947993 | intracellular transport |
| ENSG00000231167 | SNX2 | -0,1573 | -0,23954 | 0,320901 | intracellular transport |
| ENSG00000237214 | SNX8 | 0,195561 | 0,42525 | 0,380531 | intracellular transport |
| ENSG00000213866 | SNX9 | -0,18861 | 0,160675 | 0,516621 | intracellular transport |
| ENSG00000136149 | SORL1 | -1,49426 | -0,77317 | 1,96007 | intracellular transport |
| ENSG00000178654 | SORT1 | -2,01824 | -1,41772 | 1,437253 | intracellular transport |
| ENSG00000233778 | SPNS1 | 0,648022 | 0,934043 | 0,784591 | intracellular transport |
| ENSG00000189316 | SPNS2 | -0,26912 | -0,4668 | 0,34302 | intracellular transport |
| ENSG00000196756 | SRI | 1,201872 | 1,498434 | 0,986217 | intracellular transport |
| ENSG00000177173 | SRPR | 0,870668 | 0,852898 | 1,163814 | intracellular transport |
| ENSG00000197958 | SSR4 | 0,20335 | 0,041202 | 0,379715 | intracellular transport |
| ENSG00000063177 | STAM | 2,098835 | 1,539117 | 3,011633 | intracellular transport |
| ENSG00000063177 | STARD3 | 0,621222 | 0,714943 | 0,862334 | intracellular transport |
| ENSG00000131469 | STEAP3 | 0,33235 | 0,693943 | 0,49398 | intracellular transport |
| ENSG00000165502 | STX10 | 0,296406 | 0,154652 | 0,931149 | intracellular transport |
| ENSG00000174444 | STX16 | 0,172218 | -0,03533 | 0,712645 | intracellular transport |
| ENSG00000174444 | STX3 | -1,8682 | -1,31349 | 1,062095 | intracellular transport |
| ENSG00000161016 | STX4 | 0,089021 | -0,13011 | 0,498264 | intracellular transport |
| ENSG00000089157 | STX5 | 0,181836 | -0,25881 | 0,842768 | intracellular transport |
| ENSG00000235508 | SYTL1 | -0,59544 | -0,73948 | 0,097292 | intracellular transport |
| ENSG00000130254 | TCN1 | -1,32265 | -0,47872 | 2,945433 | intracellular transport |
| ENSG00000130590 | TCOF1 | 0,554152 | 0,177147 | -0,00576 | intracellular transport |
| ENSG00000175467 | TGFBRAP1 | 0,667055 | 0,652533 | 0,063561 | intracellular transport |
| ENSG00000075856 | THOC1 | 0,545195 | 0,219667 | 0,075193 | intracellular transport |
| ENSG00000075856 | THOC2 | 0,249897 | -0,09802 | -0,19833 | intracellular transport |
| ENSG00000075856 | THOC4 | 2,279205 | 2,529663 | 1,831115 | intracellular transport |
| ENSG00000075856 | THOC5 | 0,21578 | -0,36165 | -0,32644 | intracellular transport |
| ENSG00000075856 | THOC6 | 1,779647 | 1,568566 | 1,050358 | intracellular transport |
| ENSG00000064932 | TIMM17B | -0,06498 | -0,18995 | 0,034228 | intracellular transport |
| ENSG00000126461 | TIMM44 | 0,520251 | 0,378068 | 0,496542 | intracellular transport |
| ENSG00000099194 | TLK1 | 0,30825 | 0,318421 | 0,352889 | intracellular transport |
| ENSG00000136021 | TM9SF2 | -0,10277 | 0,89139 | 1,704061 | intracellular transport |
| ENSG00000093183 | TMED10 | 1,330297 | 1,317146 | 1,437162 | intracellular transport |
| ENSG00000113615 | TMED2 | 2,672355 | 2,516758 | 2,56275 | intracellular transport |
| ENSG00000138802 | TMED4 | 0,880497 | 0,904436 | 2,20986 | intracellular transport |
| ENSG00000176986 | TMED5 | -1,19744 | -1,03832 | 0,586278 | intracellular transport |
| ENSG00000107290 | TMX1 | -0,13274 | 0,160064 | -0,0811 | intracellular transport |
| ENSG00000099995 | TMX4 | -0,16169 | -0,17967 | 0,689454 | intracellular transport |
| ENSG00000139218 | TNPO1 | 0,209317 | 0,064318 | 0,605369 | intracellular transport |
| ENSG00000112081 | TNPO2 | 1,587448 | 1,339129 | 1,153715 | intracellular transport |
| ENSG00000111786 | TOM1 | -0,09549 | 0,020659 | 0,882859 | intracellular transport |
| ENSG00000111786 | TOM1L2 | -0,47608 | -0,28159 | 0,021168 | intracellular transport |
| ENSG00000198964 | TOMM7 | 0,620023 | 0,58212 | 0,72805 | intracellular transport |
| ENSG00000164054 | TPCN2 | -0,17261 | -0,3393 | 0,128715 | intracellular transport |
| ENSG00000149577 | TPR | 0,333972 | 0,100931 | -0,06355 | intracellular transport |
| ENSG00000096717 | TRAK2 | -0,68714 | 0,447823 | 2,774453 | intracellular transport |
| ENSG00000170577 | TRAPPC4 | 0,17944 | 0,399723 | 0,522662 | intracellular transport |
| ENSG00000100075 | TRPM2 | -0,701 | -0,77107 | 0,484287 | intracellular transport |
| ENSG00000127526 | TTYH3 | -0,40092 | -0,9064 | 0,264777 | intracellular transport |
| ENSG00000137700 | TUBA1A | -0,27724 | -0,35483 | 0,774084 | intracellular transport |
| ENSG00000111371 | TUBA1B | 2,774675 | 2,923088 | 2,903473 | intracellular transport |
| ENSG00000157637 | TUBA1C | 1,121784 | 1,605301 | 1,23729 | intracellular transport |
| ENSG00000134294 | TUBB | 4,167375 | 4,484843 | 4,386993 | intracellular transport |
| ENSG00000164889 | TXN | -0,06932 | 0,387357 | -0,01184 | intracellular transport |
| ENSG00000114923 | TXN2 | 0,265646 | 0,179162 | -0,43704 | intracellular transport |
| ENSG00000196517 | TXNL1 | 0,878734 | 0,610648 | 0,929876 | intracellular transport |
| ENSG00000207098 | UCP2 | 1,482449 | 2,498933 | 3,184333 | intracellular transport |
| ENSG00000206928 | UNC93B1 | -0,20818 | -0,40492 | 1,068301 | intracellular transport |
| ENSG00000239035 | UPF3A | 1,192158 | 1,30125 | 1,445956 | intracellular transport |
| ENSG00000201199 | UQCRC2 | 1,232927 | 1,119762 | 0,689704 | intracellular transport |
| ENSG00000199675 | UQCRQ | 0,048608 | -0,17303 | -0,41147 | intracellular transport |
| ENSG00000159140 | VCP | 1,44419 | 1,6991 | 1,847994 | intracellular transport |
| ENSG00000107742 | VTI1A | -0,38979 | -0,50278 | 0,020865 | intracellular transport |
| ENSG00000133226 | WNK1 | 0,94233 | 1,485558 | 3,653813 | intracellular transport |
| ENSG00000124783 | XK | -0,30128 | 0,154213 | 0,695146 | intracellular transport |
| ENSG00000180879 | XPO1 | 0,25478 | 0,333269 | -0,03788 | intracellular transport |
| ENSG00000149136 | XPO6 | 1,492558 | 1,739263 | 2,011932 | intracellular transport |
| ENSG00000173465 | XPO7 | -0,19121 | 0,241507 | 3,870203 | intracellular transport |
| ENSG00000200002 | XPOT | 0,161233 | -0,33145 | -0,65168 | intracellular transport |
| ENSG00000118046 | YWHAH | 0,703602 | 1,140443 | 0,95818 | intracellular transport |
| ENSG00000092201 | ZDHHC17 | -1,14773 | -1,11579 | 1,193873 | intracellular transport |
| ENSG00000200649 | AAAS | 0,514471 | 0,751675 | 0,341378 | cell signaling & protein processing |
| ENSG00000201035 | AAK1 | -0,23668 | -0,54473 | 0,515482 | cell signaling & protein processing |
| ENSG00000201369 | AARS | -0,23556 | 0,353308 | -0,26459 | cell signaling & protein processing |
| ENSG00000202248 | ABCB7 | -0,67568 | -0,46731 | 0,232039 | cell signaling & protein processing |
| ENSG00000200873 | ABHD5 | 0,611242 | 1,246371 | 2,404884 | cell signaling & protein processing |
| ENSG00000201041 | ABI1 | -0,33454 | -0,84902 | 0,887995 | cell signaling & protein processing |
| ENSG00000201274 | ABL1 | 0,628283 | 0,264633 | -0,03905 | cell signaling & protein processing |
| ENSG00000202474 | ABR | -1,1632 | -1,18525 | 0,410738 | cell signaling & protein processing |
| ENSG00000239846 | ACAA1 | -0,31362 | -0,40902 | 0,325382 | cell signaling & protein processing |
| ENSG00000172809 | ACADM | -0,33898 | 0,070605 | -0,55345 | cell signaling & protein processing |
| ENSG00000213373 | ACADS | -0,01472 | 0,071806 | 0,194801 | cell signaling & protein processing |
| ENSG00000224411 | ACADVL | 1,962135 | 1,70295 | 1,680903 | cell signaling & protein processing |
| ENSG00000241883 | ACHE | -0,74433 | -0,4186 | 0,234686 | cell signaling & protein processing |
| ENSG00000239768 | ACLY | 1,153139 | 0,906135 | 0,630473 | cell signaling & protein processing |
| ENSG00000243301 | ACOT8 | -0,34547 | -0,31498 | 0,024091 | cell signaling & protein processing |
| ENSG00000242000 | ACSL1 | -0,90803 | -0,57693 | 1,393108 | cell signaling & protein processing |
| ENSG00000241310 | ACSL5 | 0,210023 | 0,367712 | -0,64107 | cell signaling & protein processing |
| ENSG00000243748 | ACSL6 | -1,76314 | -1,7098 | 1,480113 | cell signaling & protein processing |
| ENSG00000241141 | ACTG1 | 2,938815 | 2,798658 | 4,431253 | cell signaling & protein processing |
| ENSG00000123908 | ACTN4 | 2,437775 | 2,628628 | 3,074963 | cell signaling & protein processing |
| ENSG00000239966 | ACTR2 | 1,019979 | 1,326904 | 2,136704 | cell signaling & protein processing |
| ENSG00000212789 | ACTR3 | 1,008999 | 1,478907 | 2,972893 | cell signaling & protein processing |
| ENSG00000242064 | ADAM10 | -0,65581 | -0,08317 | 0,020097 | cell signaling & protein processing |
| ENSG00000241561 | ADAM15 | -0,43196 | -0,08531 | 1,008878 | cell signaling & protein processing |
| ENSG00000240545 | ADAM33 | -0,18226 | -0,32758 | 0,340987 | cell signaling & protein processing |
| ENSG00000213726 | ADAM8 | -0,3791 | -0,57777 | 0,670667 | cell signaling & protein processing |
| ENSG00000240831 | ADAM9 | -0,7148 | -1,34083 | 0,630983 | cell signaling & protein processing |
| ENSG00000185900 | ADAMTS7 | 0,740861 | 0,345159 | 0,875797 | cell signaling & protein processing |
| ENSG00000236562 | ADCY7 | 0,46659 | 0,806491 | 0,198703 | cell signaling & protein processing |
| ENSG00000113141 | ADM | -0,15634 | -0,51955 | 0,702664 | cell signaling & protein processing |
| ENSG00000113141 | ADM2 | 0,055621 | -0,32438 | 0,208907 | cell signaling & protein processing |
| ENSG00000113141 | ADNP | -0,00844 | 0,1121 | 0,424782 | cell signaling & protein processing |
| ENSG00000180843 | ADRBK1 | 0,469244 | 0,514096 | 1,669784 | cell signaling & protein processing |
| ENSG00000197825 | ADRM1 | 1,255297 | 1,274716 | 1,277891 | cell signaling & protein processing |
| ENSG00000240619 | AES | 1,165322 | 1,109215 | 1,260814 | cell signaling & protein processing |
| ENSG00000234742 | AGAP2 | -0,68557 | -0,71433 | 0,675942 | cell signaling & protein processing |
| ENSG00000234386 | AGER | -0,26758 | -0,54054 | 0,298349 | cell signaling & protein processing |
| ENSG00000151539 | AGPAT1 | 0,422867 | 0,602745 | 0,058247 | cell signaling & protein processing |
| ENSG00000060971 | AGPAT2 | -0,38924 | -0,41838 | 0,444509 | cell signaling & protein processing |
| ENSG00000117054 | AGTPBP1 | -1,24081 | -1,06234 | 1,126669 | cell signaling & protein processing |
| ENSG00000114331 | AHCYL1 | 0,562929 | 0,05574 | -0,17555 | cell signaling & protein processing |
| ENSG00000182827 | AHSA1 | 0,096385 | 0,048949 | -0,11089 | cell signaling & protein processing |
| ENSG00000100813 | AIF1 | -1,52785 | -1,04022 | 1,092618 | cell signaling & protein processing |
| ENSG00000142513 | AKAP13 | -0,9579 | -1,08135 | 0,731816 | cell signaling & protein processing |
| ENSG00000181786 | AKT1 | 1,744731 | 1,7493 | 1,843017 | cell signaling & protein processing |
| ENSG00000130402 | AKT2 | 0,845838 | 1,114623 | 1,466699 | cell signaling & protein processing |
| ENSG00000011243 | ALAS2 | 0,434754 | 2,2882 | 4,423633 | cell signaling & protein processing |
| ENSG00000162482 | ALDOA | 1,684066 | 1,497649 | 2,144422 | cell signaling & protein processing |
| ENSG00000142208 | ALG12 | 0,233699 | -0,08423 | 0,230555 | cell signaling & protein processing |
| ENSG00000204673 | ALG3 | 0,939102 | 1,040432 | 0,444572 | cell signaling & protein processing |
| ENSG00000215400 | ALPI | -0,23004 | -0,60957 | 0,10734 | cell signaling & protein processing |
| ENSG00000227849 | ALPPL2 | 0,204743 | -0,28555 | 0,157363 | cell signaling & protein processing |
| ENSG00000243194 | AMFR | 0,329549 | 0,444698 | 0,794869 | cell signaling & protein processing |
| ENSG00000241447 | AMN | 0,290926 | 0,055993 | 0,634268 | cell signaling & protein processing |
| ENSG00000235771 | AMZ2 | -0,78263 | -0,59578 | 0,016914 | cell signaling & protein processing |
| ENSG00000230997 | ANK1 | 2,372535 | 2,786528 | 3,679803 | cell signaling & protein processing |
| ENSG00000243451 | ANP32A | 1,507006 | 1,398521 | 1,677815 | cell signaling & protein processing |
| ENSG00000243482 | ANPEP | -1,11249 | -0,27443 | 1,555631 | cell signaling & protein processing |
| ENSG00000243173 | ANXA1 | 1,757694 | 1,972089 | 5,086203 | cell signaling & protein processing |
| ENSG00000244066 | ANXA7 | -0,08201 | -0,68386 | 0,36166 | cell signaling & protein processing |
| ENSG00000006534 | AP1B1 | 1,009712 | 1,053115 | 0,739508 | cell signaling & protein processing |
| ENSG00000149925 | AP1G1 | 0,833657 | 0,480922 | 1,764698 | cell signaling & protein processing |
| ENSG00000091542 | AP1M1 | -0,21895 | -0,22314 | 0,670692 | cell signaling & protein processing |
| ENSG00000012779 | AP2A1 | 1,226376 | 1,889858 | 2,397944 | cell signaling & protein processing |
| ENSG00000163295 | AP2M1 | 1,167 | 1,457802 | 1,605599 | cell signaling & protein processing |
| ENSG00000163286 | AP2S1 | 0,870725 | 1,091071 | 1,155227 | cell signaling & protein processing |
| ENSG00000159461 | APEH | 0,422867 | 0,668121 | 0,39407 | cell signaling & protein processing |
| ENSG00000089053 | APP | -0,82592 | -0,80844 | 0,718683 | cell signaling & protein processing |
| ENSG00000089053 | AQP1 | -0,60447 | 0,526029 | 1,980077 | cell signaling & protein processing |
| ENSG00000089053 | ARAF | 0,925322 | 0,659303 | 0,976474 | cell signaling & protein processing |
| ENSG00000089053 | ARAP1 | -0,11931 | 0,053797 | 1,564385 | cell signaling & protein processing |
| ENSG00000089053 | ARC | 0,02053 | -0,52985 | -0,10022 | cell signaling & protein processing |
| ENSG00000089053 | ARF1 | 2,678225 | 2,527058 | 3,286393 | cell signaling & protein processing |
| ENSG00000089053 | ARF3 | 2,090208 | 1,82361 | 2,370996 | cell signaling & protein processing |
| ENSG00000089053 | ARF4 | 0,190856 | 0,391653 | 1,402299 | cell signaling & protein processing |
| ENSG00000089053 | ARF5 | 0,341049 | 0,579516 | 1,025313 | cell signaling & protein processing |
| ENSG00000196510 | ARF6 | 1,411891 | 1,36479 | 1,772039 | cell signaling & protein processing |
| ENSG00000029534 | ARFGAP1 | -0,21206 | -0,20979 | 0,269387 | cell signaling & protein processing |
| ENSG00000185722 | ARFGAP2 | 0,519798 | 0,309853 | 0,893088 | cell signaling & protein processing |
| ENSG00000154122 | ARFGEF1 | -0,8486 | -0,54732 | 1,174756 | cell signaling & protein processing |
| ENSG00000131503 | ARG1 | -1,8672 | -1,62827 | 2,182534 | cell signaling & protein processing |
| ENSG00000167522 | ARHGAP15 | -0,4898 | -0,16298 | 0,998484 | cell signaling & protein processing |
| ENSG00000164236 | ARHGDIA | 0,964977 | 0,789008 | 2,077855 | cell signaling & protein processing |
| ENSG00000213337 | ARHGDIB | 1,467802 | 1,340886 | 3,782263 | cell signaling & protein processing |
| ENSG00000100124 | ARHGEF12 | -0,695 | -0,29776 | 2,094691 | cell signaling & protein processing |
| ENSG00000156381 | ARHGEF18 | -0,34164 | -0,27071 | 0,746291 | cell signaling & protein processing |
| ENSG00000064999 | ARHGEF2 | 0,277696 | 0,558861 | 1,424291 | cell signaling & protein processing |
| ENSG00000135046 | ARIH2 | 0,296214 | 0,285038 | 0,4683 | cell signaling & protein processing |
| ENSG00000244554 | ARPC1B | 1,485772 | 1,133811 | 2,902253 | cell signaling & protein processing |
| ENSG00000243605 | ARPC2 | 1,891483 | 1,920197 | 3,808353 | cell signaling & protein processing |
| ENSG00000243172 | ARPC3 | 0,461133 | 0,322378 | 1,699319 | cell signaling & protein processing |
| ENSG00000197001 | ARPC5 | 0,563942 | 0,234348 | 1,467766 | cell signaling & protein processing |
| ENSG00000166747 | ARRB1 | 0,437125 | 0,261784 | -0,09405 | cell signaling & protein processing |
| ENSG00000213983 | ARRB2 | 0,289847 | 0,835993 | 2,792753 | cell signaling & protein processing |
| ENSG00000006125 | ART4 | -2,22866 | -2,18293 | 1,158286 | cell signaling & protein processing |
| ENSG00000157823 | ASB6 | -0,15645 | -0,12118 | 0,561985 | cell signaling & protein processing |
| ENSG00000077420 | ASB8 | -0,21274 | 0,302877 | 0,964537 | cell signaling & protein processing |
| ENSG00000103569 | ATF6 | 0,772547 | 0,576092 | 0,729438 | cell signaling & protein processing |
| ENSG00000186635 | ATG16L2 | -0,66971 | -0,59064 | 0,959725 | cell signaling & protein processing |
| ENSG00000143761 | ATG3 | 1,439676 | 1,511094 | 1,321513 | cell signaling & protein processing |
| ENSG00000143761 | ATG4D | 0,916919 | 1,330535 | 2,238608 | cell signaling & protein processing |
| ENSG00000143761 | ATG9A | -0,05097 | 0,420196 | 1,559349 | cell signaling & protein processing |
| ENSG00000143761 | ATP1A1 | 1,448474 | 1,801546 | 1,631183 | cell signaling & protein processing |
| ENSG00000134287 | ATP5A1 | 1,410902 | 1,390718 | 0,492555 | cell signaling & protein processing |
| ENSG00000134287 | ATP5B | 2,648765 | 2,491138 | 2,225232 | cell signaling & protein processing |
| ENSG00000134287 | ATP5C1 | 0,759726 | 0,645546 | 0,067222 | cell signaling & protein processing |
| ENSG00000134287 | ATP5D | 1,228063 | 1,351332 | 1,282373 | cell signaling & protein processing |
| ENSG00000134287 | ATP5E | 0,629059 | 0,728904 | 0,47356 | cell signaling & protein processing |
| ENSG00000134287 | ATP5F1 | 0,855353 | 0,699196 | 0,723771 | cell signaling & protein processing |
| ENSG00000134287 | ATP5G1 | 3,042195 | 3,146863 | 1,211637 | cell signaling & protein processing |
| ENSG00000004059 | ATP5H | 0,884735 | 0,871697 | 0,826748 | cell signaling & protein processing |
| ENSG00000165527 | ATP5I | 0,987092 | 0,978266 | 0,826565 | cell signaling & protein processing |
| ENSG00000101199 | ATP5J2 | 1,261065 | 1,086918 | 0,973626 | cell signaling & protein processing |
| ENSG00000149182 | ATP5L | 1,10603 | 0,979927 | 1,09232 | cell signaling & protein processing |
| ENSG00000066777 | ATP5O | 1,635497 | 1,39386 | 0,983243 | cell signaling & protein processing |
| ENSG00000187951 | ATP6V0A1 | -0,19945 | 0,752059 | 2,221426 | cell signaling & protein processing |
| ENSG00000075884 | ATP6V0B | -0,64926 | 0,093235 | 2,16316 | cell signaling & protein processing |
| ENSG00000213390 | ATP6V0C | 2,613645 | 2,856068 | 3,727933 | cell signaling & protein processing |
| ENSG00000225485 | ATP6V0D1 | 0,812819 | 1,554646 | 2,221935 | cell signaling & protein processing |
| ENSG00000225485 | ATP6V0E1 | -0,53148 | 0,671495 | 1,105413 | cell signaling & protein processing |
| ENSG00000145819 | ATP6V0E2 | -0,12928 | -0,28042 | 0,094801 | cell signaling & protein processing |
| ENSG00000186517 | ATP6V1B2 | 0,176324 | 0,342704 | 1,404466 | cell signaling & protein processing |
| ENSG00000089820 | ATP6V1E1 | 0,215027 | -0,11625 | 0,257189 | cell signaling & protein processing |
| ENSG00000123329 | ATP6V1F | 1,174345 | 1,154549 | 1,619964 | cell signaling & protein processing |
| ENSG00000141522 | ATP6V1G1 | 1,638073 | 1,160542 | 1,784083 | cell signaling & protein processing |
| ENSG00000177479 | AURKB | 0,883562 | 1,249247 | 1,1735 | cell signaling & protein processing |
| ENSG00000213465 | AXIN1 | -0,2756 | -0,5623 | 0,152226 | cell signaling & protein processing |
| ENSG00000182196 | AZU1 | 0,236817 | 1,109149 | 2,958423 | cell signaling & protein processing |
| ENSG00000105676 | B4GALT1 | -0,66414 | -0,77718 | 0,675326 | cell signaling & protein processing |
| ENSG00000114098 | B4GALT2 | 0,998003 | 1,00475 | 0,748936 | cell signaling & protein processing |
| ENSG00000198960 | B4GALT3 | 0,235646 | 0,047294 | 1,211958 | cell signaling & protein processing |
| ENSG00000145050 | B4GALT5 | 0,409457 | 1,266682 | 3,065813 | cell signaling & protein processing |
| ENSG00000130429 | BAD | 0,166576 | 0,094479 | 0,559147 | cell signaling & protein processing |
| ENSG00000163466 | BAG1 | -0,17388 | -0,23385 | 0,458101 | cell signaling & protein processing |
| ENSG00000111229 | BAG4 | 0,652208 | 0,129469 | 0,732698 | cell signaling & protein processing |
| ENSG00000137486 | BAP1 | 0,836435 | 1,078119 | 1,187603 | cell signaling & protein processing |
| ENSG00000148331 | BBC3 | 0,055892 | -0,01567 | 0,555735 | cell signaling & protein processing |
| ENSG00000143970 | BCL3 | 0,033871 | -0,10051 | 0,855052 | cell signaling & protein processing |
| ENSG00000138138 | BCL6 | -2,02711 | -1,43335 | 1,082549 | cell signaling & protein processing |
| ENSG00000170653 | BCR | 0,041941 | -0,42103 | 0,59849 | cell signaling & protein processing |
| ENSG00000138363 | BIN3 | -0,7854 | -0,6886 | 0,348232 | cell signaling & protein processing |
| ENSG00000119787 | BIRC2 | -0,70432 | -0,51922 | 0,445813 | cell signaling & protein processing |
| ENSG00000068650 | BLMH | 0,086584 | -0,7692 | -1,38066 | cell signaling & protein processing |
| ENSG00000058063 | BLOC1S1 | 0,151288 | 0,214788 | 0,847529 | cell signaling & protein processing |
| ENSG00000105726 | BLOC1S3 | 1,693759 | 1,223926 | 1,786507 | cell signaling & protein processing |
| ENSG00000129244 | BLZF1 | -0,49527 | -0,47223 | 0,88866 | cell signaling & protein processing |
| ENSG00000116459 | BRAF | 0,373469 | 0,374479 | 1,144173 | cell signaling & protein processing |
| ENSG00000159199 | BRAP | 1,057069 | 0,362463 | 0,707607 | cell signaling & protein processing |
| ENSG00000241468 | BRD4 | 0,906906 | 0,97307 | 1,55134 | cell signaling & protein processing |
| ENSG00000117410 | BSG | 2,478835 | 2,997613 | 4,430553 | cell signaling & protein processing |
| ENSG00000113732 | BTG2 | 0,108047 | 0,416328 | 2,315779 | cell signaling & protein processing |
| ENSG00000113732 | BTRC | -1,24373 | -0,92234 | 0,321769 | cell signaling & protein processing |
| ENSG00000113732 | BUB1B | 0,44896 | 0,577264 | 0,444271 | cell signaling & protein processing |
| ENSG00000104043 | C11orf2 | 1,028609 | 1,009835 | 1,480223 | cell signaling & protein processing |
| ENSG00000163512 | C12orf51 | 0,158196 | 0,502465 | 2,683893 | cell signaling & protein processing |
| ENSG00000166619 | C19orf2 | 0,465723 | 0,468383 | 0,109928 | cell signaling & protein processing |
| ENSG00000087302 | C3AR1 | -2,18893 | -1,74095 | 0,495276 | cell signaling & protein processing |
| ENSG00000172366 | C5AR1 | -1,02772 | -0,40023 | 1,174554 | cell signaling & protein processing |
| ENSG00000105700 | CABIN1 | 0,889934 | 1,152878 | 0,453742 | cell signaling & protein processing |
| ENSG00000105700 | CAD | 0,025253 | 0,138237 | -0,63177 | cell signaling & protein processing |
| ENSG00000104979 | CALM2 | -0,15785 | 0,008678 | 0,01243 | cell signaling & protein processing |
| ENSG00000183019 | CALR | 2,767475 | 2,733513 | 2,998293 | cell signaling & protein processing |
| ENSG00000006015 | CAMK2G | -0,24589 | -0,3872 | 0,516296 | cell signaling & protein processing |
| ENSG00000105393 | CAMKK2 | -0,24527 | -0,26132 | 1,344421 | cell signaling & protein processing |
| ENSG00000197982 | CANX | 2,055311 | 1,723003 | 1,279802 | cell signaling & protein processing |
| ENSG00000162384 | CAP1 | 0,603849 | 0,756069 | 2,969983 | cell signaling & protein processing |
| ENSG00000057757 | CAPG | -1,33893 | -1,11196 | 0,87229 | cell signaling & protein processing |
| ENSG00000055070 | CAPN1 | 1,064977 | 1,233435 | 1,55977 | cell signaling & protein processing |
| ENSG00000143110 | CAPN7 | -0,83623 | -1,03233 | 0,046394 | cell signaling & protein processing |
| ENSG00000143793 | CAPS | 0,374043 | 0,173602 | 0,67215 | cell signaling & protein processing |
| ENSG00000130775 | CAPZA1 | 0,616311 | 0,507446 | 1,654698 | cell signaling & protein processing |
| ENSG00000143612 | CAPZA2 | -0,14992 | 0,032427 | 0,814163 | cell signaling & protein processing |
| ENSG00000162642 | CAPZB | -0,47225 | 0,089259 | 1,362343 | cell signaling & protein processing |
| ENSG00000143443 | CARHSP1 | 0,000795 | 0,363354 | 0,881751 | cell signaling & protein processing |
| ENSG00000162819 | CARM1 | 1,180119 | 1,43784 | 1,941211 | cell signaling & protein processing |
| ENSG00000094975 | CASP2 | 0,625451 | 0,6815 | 1,111503 | cell signaling & protein processing |
| ENSG00000157870 | CASP8 | -0,39152 | -0,54729 | 1,044953 | cell signaling & protein processing |
| ENSG00000172247 | CAT | 2,427965 | 2,529258 | 3,650483 | cell signaling & protein processing |
| ENSG00000223953 | CBL | -2,01032 | -1,66657 | 0,50953 | cell signaling & protein processing |
| ENSG00000100220 | CCDC88C | 0,132741 | 0,078741 | 0,580366 | cell signaling & protein processing |
| ENSG00000100220 | CCL5 | -1,72907 | -1,78776 | 0,118568 | cell signaling & protein processing |
| ENSG00000100220 | CCNB1 | -0,62496 | 0,029889 | -0,56356 | cell signaling & protein processing |
| ENSG00000100364 | CCND3 | 1,097833 | 1,711995 | 3,270123 | cell signaling & protein processing |
| ENSG00000168014 | CCNE1 | -0,5174 | 0,437797 | -0,26777 | cell signaling & protein processing |
| ENSG00000115649 | CCNT1 | 0,836382 | 0,391109 | 0,684281 | cell signaling & protein processing |
| ENSG00000115649 | CCR7 | -1,42372 | -0,95976 | 0,144359 | cell signaling & protein processing |
| ENSG00000138085 | CCT2 | 0,627972 | 0,726466 | 0,327176 | cell signaling & protein processing |
| ENSG00000158435 | CCT3 | 1,354265 | 1,259057 | 0,052855 | cell signaling & protein processing |
| ENSG00000168887 | CCT4 | 0,589545 | 0,807337 | 0,076138 | cell signaling & protein processing |
| ENSG00000182600 | CCT5 | 0,612724 | 0,48395 | -0,32044 | cell signaling & protein processing |
| ENSG00000171860 | CCT6A | 0,693635 | 0,685684 | -0,57816 | cell signaling & protein processing |
| ENSG00000113845 | CCT7 | 1,497005 | 1,598153 | 0,33789 | cell signaling & protein processing |
| ENSG00000183624 | CD2AP | -0,55671 | -0,4937 | 0,142684 | cell signaling & protein processing |
| ENSG00000173769 | CD36 | 1,447629 | 2,079766 | 1,79 | cell signaling & protein processing |
| ENSG00000112667 | CD59 | 0,7728 | 0,708067 | 1,366254 | cell signaling & protein processing |
| ENSG00000112308 | CD74 | 2,334515 | 1,855249 | 3,374823 | cell signaling & protein processing |
| ENSG00000146576 | CD81 | 1,633828 | 1,366139 | 1,403704 | cell signaling & protein processing |
| ENSG00000146826 | CD97 | 0,208697 | 0,481365 | 2,870933 | cell signaling & protein processing |
| ENSG00000160813 | CDA | -0,55824 | 0,042579 | 2,813753 | cell signaling & protein processing |
| ENSG00000130193 | CDC25B | -0,5142 | -0,34155 | 0,787994 | cell signaling & protein processing |
| ENSG00000137038 | CDC34 | 0,438722 | 0,795453 | 2,33751 | cell signaling & protein processing |
| ENSG00000198454 | CDC37 | 0,925044 | 0,925414 | 1,114869 | cell signaling & protein processing |
| ENSG00000171159 | CDC42 | 0,822311 | 0,676139 | 1,412197 | cell signaling & protein processing |
| ENSG00000197191 | CDC42BPA | 0,406904 | 0,038031 | -0,914 | cell signaling & protein processing |
| ENSG00000135045 | CDC42EP3 | -1,94039 | -1,76248 | 0,117727 | cell signaling & protein processing |
| ENSG00000106771 | CDC42EP4 | -0,39123 | -0,28763 | 0,033473 | cell signaling & protein processing |
| ENSG00000136819 | CDC42EP5 | 0,642379 | 0,401042 | 0,666056 | cell signaling & protein processing |
| ENSG00000196642 | CDC42SE1 | 0,111865 | -0,42553 | 0,916389 | cell signaling & protein processing |
| ENSG00000165233 | CDC42SE2 | 1,076142 | 0,343671 | 1,164027 | cell signaling & protein processing |
| ENSG00000135932 | CDC73 | 1,306844 | 0,986858 | 1,56656 | cell signaling & protein processing |
| ENSG00000136436 | CDK2 | 0,31564 | 0,90757 | 0,262399 | cell signaling & protein processing |
| ENSG00000138172 | CDK2AP1 | 2,387445 | 1,856849 | 1,588838 | cell signaling & protein processing |
| ENSG00000143933 | CDK4 | 2,003161 | 2,230078 | 0,758548 | cell signaling & protein processing |
| ENSG00000160014 | CDK5R1 | -0,42972 | -0,41698 | 0,071149 | cell signaling & protein processing |
| ENSG00000163888 | CDK6 | 1,513185 | 1,222627 | -0,72135 | cell signaling & protein processing |
| ENSG00000110931 | CDK9 | 1,054163 | 0,974569 | 2,007733 | cell signaling & protein processing |
| ENSG00000164047 | CDKN1B | 0,448234 | 0,29123 | 2,116854 | cell signaling & protein processing |
| ENSG00000127022 | CENPF | 0,675305 | 1,061737 | 0,850759 | cell signaling & protein processing |
| ENSG00000131236 | CETN2 | 0,064315 | 0,535359 | 0,363212 | cell signaling & protein processing |
| ENSG00000131375 | CFLAR | -1,47228 | -1,30957 | 0,413442 | cell signaling & protein processing |
| ENSG00000126247 | CHAF1A | 1,039531 | 1,39772 | 0,842912 | cell signaling & protein processing |
| ENSG00000126247 | CHD9 | 0,190418 | -0,15372 | 0,519193 | cell signaling & protein processing |
| ENSG00000135387 | CHFR | -0,39174 | -0,29283 | 0,574574 | cell signaling & protein processing |
| ENSG00000110888 | CHI3L1 | -0,41893 | 0,235396 | 3,390973 | cell signaling & protein processing |
| ENSG00000187796 | CHMP1A | 0,445823 | 0,501365 | 1,044277 | cell signaling & protein processing |
| ENSG00000153048 | CHMP2A | 1,393108 | 1,678634 | 2,934233 | cell signaling & protein processing |
| ENSG00000142453 | CHMP4B | 0,003267 | 0,159658 | 0,603706 | cell signaling & protein processing |
| ENSG00000122565 | CIDEB | -0,07001 | -0,24291 | 0,099129 | cell signaling & protein processing |
| ENSG00000060339 | CIT | 0,448096 | 0,71722 | 0,536321 | cell signaling & protein processing |
| ENSG00000104983 | CLCF1 | -0,23545 | -0,50414 | 0,263607 | cell signaling & protein processing |
| ENSG00000118971 | CLINT1 | 1,497823 | 1,139747 | 2,337402 | cell signaling & protein processing |
| ENSG00000166946 | CLK1 | 1,318394 | 1,052675 | 2,209405 | cell signaling & protein processing |
| ENSG00000105173 | CLK2 | 0,499811 | 0,497799 | 0,630309 | cell signaling & protein processing |
| ENSG00000118816 | CLK3 | -0,83181 | -0,62788 | 0,87979 | cell signaling & protein processing |
| ENSG00000107443 | CLK4 | -0,72512 | -1,22331 | 0,162557 | cell signaling & protein processing |
| ENSG00000135083 | CLN3 | -0,32254 | -0,39398 | 0,111373 | cell signaling & protein processing |
| ENSG00000090061 | CLN6 | 0,797756 | 0,747702 | 0,728321 | cell signaling & protein processing |
| ENSG00000163660 | CLN8 | -0,80243 | -1,14025 | 0,080366 | cell signaling & protein processing |
| ENSG00000221978 | CLPP | 0,629877 | 0,625994 | 0,633334 | cell signaling & protein processing |
| ENSG00000082258 | CLTA | -0,05339 | 0,146293 | -0,02393 | cell signaling & protein processing |
| ENSG00000126353 | CLTC | 1,588052 | 1,855365 | 2,076391 | cell signaling & protein processing |
| ENSG00000151014 | CLTCL1 | -1,56052 | -1,44192 | 0,163322 | cell signaling & protein processing |
| ENSG00000115484 | CMTM6 | -1,7398 | -1,5824 | 0,314086 | cell signaling & protein processing |
| ENSG00000150753 | CMTM7 | -0,61202 | -0,59403 | 0,891459 | cell signaling & protein processing |
| ENSG00000135624 | CNIH4 | -1,11449 | -1,10995 | 0,616581 | cell signaling & protein processing |
| ENSG00000105383 | CNOT4 | -0,62537 | -0,71215 | 0,032188 | cell signaling & protein processing |
| ENSG00000169442 | COG1 | 0,076536 | -0,24844 | 0,330958 | cell signaling & protein processing |
| ENSG00000143119 | COG5 | -0,03038 | 0,131274 | -0,30089 | cell signaling & protein processing |
| ENSG00000110848 | COL4A3BP | -0,90944 | -0,89378 | 0,444541 | cell signaling & protein processing |
| ENSG00000019582 | COL6A2 | 0,510787 | 0,241045 | 0,821649 | cell signaling & protein processing |
| ENSG00000140326 | COPA | 1,391149 | 1,181891 | 1,437304 | cell signaling & protein processing |
| ENSG00000151465 | COPE | 1,379088 | 1,424794 | 1,60752 | cell signaling & protein processing |
| ENSG00000079335 | COPG | 0,434987 | 0,519982 | 0,471962 | cell signaling & protein processing |
| ENSG00000163171 | COX4I1 | 1,706594 | 1,432379 | 1,680475 | cell signaling & protein processing |
| ENSG00000179604 | COX5A | 2,670345 | 2,462488 | 1,82491 | cell signaling & protein processing |
| ENSG00000167617 | COX5B | 2,012208 | 2,358928 | 2,105592 | cell signaling & protein processing |
| ENSG00000158985 | COX6B1 | 1,570718 | 1,433599 | 1,52093 | cell signaling & protein processing |
| ENSG00000093009 | COX7A2L | -0,37421 | -0,06442 | 0,348818 | cell signaling & protein processing |
| ENSG00000096401 | COX8A | 1,168068 | 1,65175 | 1,754054 | cell signaling & protein processing |
| ENSG00000134371 | CPD | -0,47024 | -0,23808 | 1,430564 | cell signaling & protein processing |
| ENSG00000135446 | CPT1A | 1,259766 | 1,277313 | 1,180012 | cell signaling & protein processing |
| ENSG00000176749 | CPT1B | -0,27506 | -0,24273 | 0,017656 | cell signaling & protein processing |
| ENSG00000171450 | CPVL | -0,46262 | 0,1122 | -1,02874 | cell signaling & protein processing |
| ENSG00000108465 | CRAT | -0,20526 | -0,09486 | 0,189538 | cell signaling & protein processing |
| ENSG00000105810 | CRB3 | 0,396987 | 0,077987 | 0,359841 | cell signaling & protein processing |
| ENSG00000136807 | CREB1 | 0,284631 | 0,585755 | 1,217715 | cell signaling & protein processing |
| ENSG00000129757 | CREBBP | 0,872804 | 1,050262 | 2,530726 | cell signaling & protein processing |
| ENSG00000167513 | CRK | -0,30603 | -0,33767 | 0,288635 | cell signaling & protein processing |
| ENSG00000091527 | CRKL | 0,97521 | 0,610516 | 1,164787 | cell signaling & protein processing |
| ENSG00000221869 | CRY2 | -0,38586 | -0,46954 | 0,220293 | cell signaling & protein processing |
| ENSG00000183307 | CRYAA | 0,727103 | 0,200046 | 0,738935 | cell signaling & protein processing |
| ENSG00000197766 | CSK | -0,1904 | -0,33353 | 0,168987 | cell signaling & protein processing |
| ENSG00000172757 | CSNK1D | 1,708628 | 1,451727 | 2,009397 | cell signaling & protein processing |
| ENSG00000172757 | CSNK1E | 0,365984 | -0,16636 | 0,052773 | cell signaling & protein processing |
| ENSG00000172757 | CSNK1G2 | 0,543262 | 0,36206 | 0,771294 | cell signaling & protein processing |
| ENSG00000172757 | CSNK2A1 | 1,078124 | 1,043054 | 0,786296 | cell signaling & protein processing |
| ENSG00000163320 | CST3 | 2,721195 | 1,881301 | 2,009755 | cell signaling & protein processing |
| ENSG00000173575 | CTBP1 | 1,575714 | 1,70446 | 1,794402 | cell signaling & protein processing |
| ENSG00000085872 | CTDP1 | 0,387707 | 0,281251 | 1,211592 | cell signaling & protein processing |
| ENSG00000072609 | CTDSP1 | 0,714288 | 0,640658 | 1,336659 | cell signaling & protein processing |
| ENSG00000133048 | CTDSP2 | 0,876601 | 0,744893 | 1,46396 | cell signaling & protein processing |
| ENSG00000110721 | CTNNB1 | 1,019751 | 0,511799 | 1,89983 | cell signaling & protein processing |
| ENSG00000111666 | CTSA | 1,021083 | 1,403701 | 2,420642 | cell signaling & protein processing |
| ENSG00000104472 | CTSB | -0,10535 | -0,03604 | 2,120365 | cell signaling & protein processing |
| ENSG00000171310 | CTSD | 1,420984 | 2,131276 | 3,721303 | cell signaling & protein processing |
| ENSG00000136213 | CTSE | 0,121419 | 1,971008 | 1,496192 | cell signaling & protein processing |
| ENSG00000169105 | CTSG | -0,58122 | 0,865539 | 2,676653 | cell signaling & protein processing |
| ENSG00000182022 | CTSL1 | -1,87319 | -1,18181 | 0,89326 | cell signaling & protein processing |
| ENSG00000175040 | CTSS | -1,57181 | -1,47176 | 1,397607 | cell signaling & protein processing |
| ENSG00000131873 | CTSW | 0,348685 | -0,54708 | -0,07751 | cell signaling & protein processing |
| ENSG00000127586 | CTSZ | 0,179837 | 0,51287 | 1,369826 | cell signaling & protein processing |
| ENSG00000164442 | CUTA | 1,357564 | 1,319742 | 0,998292 | cell signaling & protein processing |
| ENSG00000123975 | CYB5R4 | -1,28982 | -1,19345 | 0,468049 | cell signaling & protein processing |
| ENSG00000175505 | CYBRD1 | -0,6764 | 0,3734 | 1,040409 | cell signaling & protein processing |
| ENSG00000109572 | CYC1 | 1,121475 | 1,440033 | 1,00772 | cell signaling & protein processing |
| ENSG00000080822 | CYTH2 | 0,94165 | 0,672698 | 0,663006 | cell signaling & protein processing |
| ENSG00000213719 | D2HGDH | -0,03251 | -0,22913 | 0,064552 | cell signaling & protein processing |
| ENSG00000113282 | DAD1 | 1,438411 | 1,917436 | 1,85037 | cell signaling & protein processing |
| ENSG00000176444 | DAPK2 | -0,38745 | -0,46475 | 1,001209 | cell signaling & protein processing |
| ENSG00000232553 | DARS | 0,530385 | 0,756522 | 0,000845 | cell signaling & protein processing |
| ENSG00000128973 | DBNL | -0,12249 | 0,029895 | 1,175338 | cell signaling & protein processing |
| ENSG00000080802 | DDI2 | 1,027401 | 1,382496 | 2,401464 | cell signaling & protein processing |
| ENSG00000173786 | DDOST | 1,16228 | 0,912348 | 0,85626 | cell signaling & protein processing |
| ENSG00000166685 | DDX19B | 0,658916 | 0,039647 | -0,18805 | cell signaling & protein processing |
| ENSG00000142173 | DDX42 | 0,651851 | 0,078068 | 0,167585 | cell signaling & protein processing |
| ENSG00000122218 | DEDD2 | 0,699057 | 0,968203 | 2,331395 | cell signaling & protein processing |
| ENSG00000181789 | DEF8 | -0,90635 | -0,76377 | 0,110689 | cell signaling & protein processing |
| ENSG00000103426 | DERL1 | -0,59632 | 0,017225 | 1,152875 | cell signaling & protein processing |
| ENSG00000006695 | DGAT1 | 0,327272 | 0,611511 | 1,73132 | cell signaling & protein processing |
| ENSG00000133983 | DGAT2 | -0,18944 | -0,4946 | 0,026269 | cell signaling & protein processing |
| ENSG00000156885 | DGKA | -0,27619 | 0,302137 | 0,73364 | cell signaling & protein processing |
| ENSG00000126267 | DGKD | -0,69779 | -0,43658 | 1,61257 | cell signaling & protein processing |
| ENSG00000115944 | DGKZ | 0,072067 | -0,21111 | 0,4071 | cell signaling & protein processing |
| ENSG00000127054 | DIAPH1 | 0,077705 | -0,0221 | 0,831172 | cell signaling & protein processing |
| ENSG00000110090 | DLG1 | -0,15683 | 0,0433 | 0,736947 | cell signaling & protein processing |
| ENSG00000118260 | DNAJA1 | 0,391792 | 0,612195 | 0,509662 | cell signaling & protein processing |
| ENSG00000182158 | DNAJA4 | 1,151832 | 1,72791 | 1,299575 | cell signaling & protein processing |
| ENSG00000005339 | DNAJB1 | 0,00727 | 0,930266 | 1,646377 | cell signaling & protein processing |
| ENSG00000213676 | DNAJB11 | 0,963331 | 0,601881 | 1,25688 | cell signaling & protein processing |
| ENSG00000143162 | DNAJB14 | -0,34177 | -1,41282 | 0,732163 | cell signaling & protein processing |
| ENSG00000096006 | DNAJB2 | -1,22731 | -1,0584 | 0,354143 | cell signaling & protein processing |
| ENSG00000167193 | DNAJB6 | 0,736072 | -0,1129 | 1,223455 | cell signaling & protein processing |
| ENSG00000099942 | DNAJC1 | 0,270914 | -0,30916 | 0,313122 | cell signaling & protein processing |
| ENSG00000167258 | DNAJC11 | 0,080347 | 0,208551 | -1,37277 | cell signaling & protein processing |
| ENSG00000176390 | DNAJC2 | 0,641007 | -0,06697 | -0,15489 | cell signaling & protein processing |
| ENSG00000215908 | DNAJC3 | -1,44751 | -1,59442 | 0,037705 | cell signaling & protein processing |
| ENSG00000170275 | DNAJC5 | -0,18735 | 0,127643 | 0,909833 | cell signaling & protein processing |
| ENSG00000105662 | DNAJC6 | -0,72019 | -0,20132 | 0,126553 | cell signaling & protein processing |
| ENSG00000160741 | DNAJC9 | 0,33522 | 0,134848 | 0,004357 | cell signaling & protein processing |
| ENSG00000160202 | DNM2 | 1,979092 | 1,938032 | 2,46743 | cell signaling & protein processing |
| ENSG00000060138 | DOC2B | -1,13896 | -0,68397 | 0,124099 | cell signaling & protein processing |
| ENSG00000124207 | DOCK2 | -0,4339 | -0,59401 | 1,532582 | cell signaling & protein processing |
| ENSG00000108342 | DOHH | 0,822711 | 0,755136 | 1,101255 | cell signaling & protein processing |
| ENSG00000119535 | DOK1 | -0,43792 | -0,46148 | 0,231274 | cell signaling & protein processing |
| ENSG00000213923 | DPM1 | -0,3922 | -0,04883 | 0,184193 | cell signaling & protein processing |
| ENSG00000133275 | DPM2 | -1,00332 | -0,63019 | 0,459994 | cell signaling & protein processing |
| ENSG00000101266 | DPM3 | 0,739668 | 0,575539 | 0,970279 | cell signaling & protein processing |
| ENSG00000070770 | DPP7 | 2,248197 | 2,190048 | 1,903501 | cell signaling & protein processing |
| ENSG00000101439 | DRD4 | 0,138397 | -0,21516 | 0,019828 | cell signaling & protein processing |
| ENSG00000236002 | DTL | 1,217166 | 1,667 | 0,58631 | cell signaling & protein processing |
| ENSG00000159692 | DTX1 | -0,80124 | -0,91784 | 0,10791 | cell signaling & protein processing |
| ENSG00000230673 | DUSP1 | 2,877685 | 2,997073 | 3,321373 | cell signaling & protein processing |
| ENSG00000144579 | DUSP2 | -0,16114 | -0,24549 | 0,572057 | cell signaling & protein processing |
| ENSG00000137770 | DUSP6 | -1,54955 | -1,01247 | 1,319974 | cell signaling & protein processing |
| ENSG00000150281 | DUSP7 | 0,240088 | 0,337013 | 0,463548 | cell signaling & protein processing |
| ENSG00000044115 | DUSP8 | 0,232758 | -0,13046 | 0,563808 | cell signaling & protein processing |
| ENSG00000168036 | DUSP9 | 0,22239 | -0,12359 | 0,283792 | cell signaling & protein processing |
| ENSG00000178585 | DVL1 | 0,734218 | 0,465856 | 0,573778 | cell signaling & protein processing |
| ENSG00000132792 | DVL2 | -0,27072 | -0,02269 | 0,232275 | cell signaling & protein processing |
| ENSG00000198561 | DVL3 | 0,458286 | 0,356591 | 0,848535 | cell signaling & protein processing |
| ENSG00000163131 | DYRK1A | 0,670617 | 0,484349 | 1,551153 | cell signaling & protein processing |
| ENSG00000172543 | DYRK1B | 0,754269 | 0,670996 | 1,127811 | cell signaling & protein processing |
| ENSG00000101160 | DYRK3 | -0,77249 | -0,77889 | 0,299603 | cell signaling & protein processing |
| ENSG00000142544 | E2F1 | 1,398456 | 1,577908 | 1,057178 | cell signaling & protein processing |
| ENSG00000154639 | ECE1 | 0,497982 | 0,747598 | 1,667194 | cell signaling & protein processing |
| ENSG00000226574 | ECHS1 | 0,204615 | 0,003085 | -0,01466 | cell signaling & protein processing |
| ENSG00000166347 | EDEM1 | -1,22159 | -1,04044 | 0,487988 | cell signaling & protein processing |
| ENSG00000159348 | EDEM3 | -1,5168 | -0,64448 | 0,095069 | cell signaling & protein processing |
| ENSG00000065615 | EEF1A1 | -0,33338 | -0,44043 | 0,34373 | cell signaling & protein processing |
| ENSG00000165168 | EEF1D | 2,104668 | 2,324098 | 2,415045 | cell signaling & protein processing |
| ENSG00000071967 | EEF2 | 3,959995 | 3,697318 | 3,320283 | cell signaling & protein processing |
| ENSG00000179091 | EEF2K | 0,270673 | 0,32836 | -0,14908 | cell signaling & protein processing |
| ENSG00000170891 | EGR1 | 0,299051 | 0,680065 | 1,483668 | cell signaling & protein processing |
| ENSG00000100014 | EHD1 | -0,56298 | -0,49449 | 0,672156 | cell signaling & protein processing |
| ENSG00000129562 | EIF1AX | 0,801819 | 0,726532 | 0,729828 | cell signaling & protein processing |
| ENSG00000164535 | EIF2A | 0,975273 | 0,895524 | 0,0523 | cell signaling & protein processing |
| ENSG00000112977 | EIF2AK1 | 1,720386 | 2,033376 | 3,690533 | cell signaling & protein processing |
| ENSG00000035664 | EIF2AK2 | -0,20741 | -0,80803 | 0,128387 | cell signaling & protein processing |
| ENSG00000115866 | EIF2B1 | 0,462407 | 0,127278 | -0,17606 | cell signaling & protein processing |
| ENSG00000003249 | EIF2S2 | 0,069042 | 0,406101 | 0,428023 | cell signaling & protein processing |
| ENSG00000136279 | EIF2S3 | 2,343645 | 1,895206 | 2,064589 | cell signaling & protein processing |
| ENSG00000105516 | EIF3A | 2,696185 | 2,713828 | 2,053979 | cell signaling & protein processing |
| ENSG00000132017 | EIF3B | 1,853965 | 1,711303 | 1,439433 | cell signaling & protein processing |
| ENSG00000139990 | EIF3D | 1,746211 | 1,657655 | 0,845489 | cell signaling & protein processing |
| ENSG00000136485 | EIF3E | 0,11304 | 0,319856 | -0,04913 | cell signaling & protein processing |
| ENSG00000172992 | EIF3F | 1,329571 | 1,334545 | 0,469693 | cell signaling & protein processing |
| ENSG00000167969 | EIF3G | 1,775135 | 1,769634 | 1,376269 | cell signaling & protein processing |
| ENSG00000156136 | EIF3H | 1,554534 | 1,397512 | 1,258489 | cell signaling & protein processing |
| ENSG00000162290 | EIF3I | 1,227978 | 1,246447 | 0,602966 | cell signaling & protein processing |
| ENSG00000172795 | EIF3J | 0,162776 | -0,32604 | 0,042093 | cell signaling & protein processing |
| ENSG00000204843 | EIF3K | 1,895609 | 1,807172 | 1,630036 | cell signaling & protein processing |
| ENSG00000132912 | EIF4A1 | 2,308925 | 2,283523 | 1,649035 | cell signaling & protein processing |
| ENSG00000166847 | EIF4A2 | 1,10195 | 1,030298 | 1,452521 | cell signaling & protein processing |
| ENSG00000130311 | EIF4EBP1 | 1,316937 | 1,083514 | 0,734489 | cell signaling & protein processing |
| ENSG00000167986 | EIF4G1 | 0,726335 | 0,756476 | 0,477349 | cell signaling & protein processing |
| ENSG00000175197 | EIF5 | 0,238497 | 0,180459 | 1,89628 | cell signaling & protein processing |
| ENSG00000165359 | ELMO1 | 0,488732 | -0,11256 | 0,508145 | cell signaling & protein processing |
| ENSG00000183258 | ELOVL1 | -0,43482 | 0,858747 | 1,190234 | cell signaling & protein processing |
| ENSG00000198231 | ELOVL5 | 2,312965 | 2,089903 | 2,169616 | cell signaling & protein processing |
| ENSG00000108654 | EMD | 0,277702 | 0,615527 | 0,889751 | cell signaling & protein processing |
| ENSG00000108654 | EP300 | 0,856371 | 0,804801 | 2,852863 | cell signaling & protein processing |
| ENSG00000123064 | EPB42 | -0,01791 | 1,462161 | 3,120203 | cell signaling & protein processing |
| ENSG00000177030 | EPHB4 | 0,98692 | 0,997667 | 0,015585 | cell signaling & protein processing |
| ENSG00000242612 | EPN1 | 0,228559 | 0,166575 | 1,11953 | cell signaling & protein processing |
| ENSG00000158796 | EPN2 | -0,78138 | -0,79236 | 0,065912 | cell signaling & protein processing |
| ENSG00000140995 | EPS15 | 1,35944 | 1,073918 | 1,092304 | cell signaling & protein processing |
| ENSG00000164821 | EPS15L1 | 1,383216 | 1,172906 | 0,779322 | cell signaling & protein processing |
| ENSG00000105339 | ERAP1 | -0,54673 | -0,75799 | 0,110539 | cell signaling & protein processing |
| ENSG00000184014 | ERCC2 | -0,18662 | -0,07666 | 0,218708 | cell signaling & protein processing |
| ENSG00000136986 | ERCC3 | 0,278499 | -0,13482 | -0,61082 | cell signaling & protein processing |
| ENSG00000149091 | ERO1L | -1,89065 | -1,69303 | 0,734189 | cell signaling & protein processing |
| ENSG00000108272 | ERP29 | 0,184268 | 0,418245 | 0,630483 | cell signaling & protein processing |
| ENSG00000167536 | ERP44 | -0,35016 | 0,097881 | 0,687581 | cell signaling & protein processing |
| ENSG00000162496 | ESPL1 | -0,89472 | -0,09935 | 0,322015 | cell signaling & protein processing |
| ENSG00000131504 | ETF1 | 0,248962 | -0,08558 | 0,37735 | cell signaling & protein processing |
| ENSG00000100697 | ETFB | -0,02719 | 0,06507 | -0,11392 | cell signaling & protein processing |
| ENSG00000006377 | EVL | -0,08061 | -0,22465 | 0,216842 | cell signaling & protein processing |
| ENSG00000086061 | EXOC3 | 0,332481 | 0,007674 | 0,629865 | cell signaling & protein processing |
| ENSG00000090520 | EXOC7 | 0,706445 | 0,465151 | 1,009999 | cell signaling & protein processing |
| ENSG00000087470 | FAF1 | 0,34645 | -0,11893 | 0,314086 | cell signaling & protein processing |
| ENSG00000133884 | FAM109A | -0,11736 | -0,30269 | 0,118884 | cell signaling & protein processing |
| ENSG00000108963 | FAM109B | -0,24309 | -0,19009 | 0,047995 | cell signaling & protein processing |
| ENSG00000108591 | FAM125A | -0,1914 | -0,23947 | 0,062313 | cell signaling & protein processing |
| ENSG00000175826 | FAM160A2 | -0,74809 | -0,50537 | 0,664501 | cell signaling & protein processing |
| ENSG00000038358 | FANCA | 0,138764 | 0,055469 | -0,29043 | cell signaling & protein processing |
| ENSG00000228502 | FASN | 0,450025 | 0,267953 | 0,027448 | cell signaling & protein processing |
| ENSG00000104529 | FASTK | 0,421196 | 0,557636 | 0,550092 | cell signaling & protein processing |
| ENSG00000167658 | FAU | -3,69451 | -4,30436 | -3,89507 | cell signaling & protein processing |
| ENSG00000171570 | FBXO2 | -0,04906 | -0,33073 | 0,189342 | cell signaling & protein processing |
| ENSG00000120738 | FBXO3 | -0,36309 | -0,27445 | 0,212699 | cell signaling & protein processing |
| ENSG00000114784 | FBXW2 | 1,091 | 1,214475 | 1,593761 | cell signaling & protein processing |
| ENSG00000092847 | FCER1G | -1,10922 | -0,91724 | 1,888377 | cell signaling & protein processing |
| ENSG00000100353 | FES | -0,98478 | -0,85539 | 0,102736 | cell signaling & protein processing |
| ENSG00000100353 | FGD3 | -0,10844 | -0,20795 | 1,368281 | cell signaling & protein processing |
| ENSG00000100353 | FGFRL1 | 0,43533 | 0,342499 | 0,572103 | cell signaling & protein processing |
| ENSG00000100353 | FGR | 0,059686 | 0,172039 | 2,608432 | cell signaling & protein processing |
| ENSG00000100353 | FHL2 | 0,084234 | -0,04738 | -0,0175 | cell signaling & protein processing |
| ENSG00000175390 | FKBP15 | -0,38769 | -0,02001 | 0,079638 | cell signaling & protein processing |
| ENSG00000130811 | FKBP4 | 1,455881 | 1,724355 | 0,057205 | cell signaling & protein processing |
| ENSG00000147677 | FKBP5 | 1,589342 | 0,701393 | 0,437461 | cell signaling & protein processing |
| ENSG00000084623 | FKBP8 | 1,873371 | 1,976211 | 3,276233 | cell signaling & protein processing |
| ENSG00000178982 | FLI1 | -1,45192 | -1,38012 | 0,283339 | cell signaling & protein processing |
| ENSG00000149100 | FLNA | 1,376735 | 0,740553 | 2,307608 | cell signaling & protein processing |
| ENSG00000187840 | FMNL1 | 0,507097 | 0,36222 | 2,125274 | cell signaling & protein processing |
| ENSG00000110321 | FNBP1 | -1,17167 | -1,19199 | 0,540682 | cell signaling & protein processing |
| ENSG00000110321 | FOS | 1,041568 | 0,989906 | 1,95571 | cell signaling & protein processing |
| ENSG00000110321 | FOSL1 | 0,337462 | -0,01494 | 0,548994 | cell signaling & protein processing |
| ENSG00000105656 | FTL | 2,889705 | 3,057693 | 4,182573 | cell signaling & protein processing |
| ENSG00000062598 | FURIN | 0,89902 | 0,551051 | 1,992159 | cell signaling & protein processing |
| ENSG00000012660 | FUZ | 0,206431 | 0,26944 | 0,321594 | cell signaling & protein processing |
| ENSG00000170571 | FXC1 | 0,415186 | 0,15352 | 0,674416 | cell signaling & protein processing |
| ENSG00000102119 | FXN | 2,153658 | 1,963684 | 0,976789 | cell signaling & protein processing |
| ENSG00000142227 | FYB | -2,46681 | -2,12534 | 0,773448 | cell signaling & protein processing |
| ENSG00000074800 | FZR1 | 0,251097 | 0,416797 | 2,04755 | cell signaling & protein processing |
| ENSG00000197217 | G3BP2 | -0,04314 | -0,14778 | 0,20391 | cell signaling & protein processing |
| ENSG00000100393 | GAB1 | 0,423881 | 0,832003 | 0,893612 | cell signaling & protein processing |
| ENSG00000166947 | GABARAP | 1,545243 | 2,14376 | 3,300593 | cell signaling & protein processing |
| ENSG00000120616 | GABARAPL2 | 0,823671 | 0,97914 | 2,175724 | cell signaling & protein processing |
| ENSG00000049283 | GADD45B | -0,02859 | -0,20438 | 0,605152 | cell signaling & protein processing |
| ENSG00000187266 | GADD45G | -1,38471 | -1,0714 | 0,359524 | cell signaling & protein processing |
| ENSG00000163161 | GANAB | 1,204793 | 1,267737 | 0,881975 | cell signaling & protein processing |
| ENSG00000124882 | GAPDH | 4,741975 | 4,569798 | 5,475943 | cell signaling & protein processing |
| ENSG00000125991 | GAPVD1 | -0,69039 | -0,5781 | 1,52345 | cell signaling & protein processing |
| ENSG00000107566 | GBA | -0,76884 | -0,52919 | 0,668551 | cell signaling & protein processing |
| ENSG00000197930 | GBF1 | 0,57587 | 0,682652 | 1,170077 | cell signaling & protein processing |
| ENSG00000135476 | GCA | -2,00952 | -1,36043 | 1,986043 | cell signaling & protein processing |
| ENSG00000120705 | GDI1 | 0,46968 | 0,613941 | 2,237572 | cell signaling & protein processing |
| ENSG00000105379 | GDI2 | -0,18802 | 0,293832 | 0,646745 | cell signaling & protein processing |
| ENSG00000182473 | GIGYF2 | 0,100938 | 0,179945 | 0,673032 | cell signaling & protein processing |
| ENSG00000108799 | GLIPR1 | -1,96406 | -1,75466 | 1,277085 | cell signaling & protein processing |
| ENSG00000153443 | GLUL | 1,597591 | 1,770431 | 2,423323 | cell signaling & protein processing |
| ENSG00000145569 | GMFG | 1,389331 | 1,234866 | 3,155773 | cell signaling & protein processing |
| ENSG00000205217 | GMIP | -1,48965 | -1,09031 | 0,962035 | cell signaling & protein processing |
| ENSG00000065809 | GMPPB | -0,63334 | -0,64626 | 0,315222 | cell signaling & protein processing |
| ENSG00000136379 | GNA12 | 0,179977 | 0,248845 | 0,333739 | cell signaling & protein processing |
| ENSG00000198324 | GNA13 | 0,558148 | 0,461584 | 1,38732 | cell signaling & protein processing |
| ENSG00000177096 | GNA15 | 1,08225 | 0,672307 | 0,811934 | cell signaling & protein processing |
| ENSG00000232150 | GNAI2 | 1,530631 | 1,474584 | 3,364273 | cell signaling & protein processing |
| ENSG00000125898 | GNAQ | 1,721139 | 1,454566 | 2,253245 | cell signaling & protein processing |
| ENSG00000184731 | GNAS | 1,636058 | 1,513349 | 1,921752 | cell signaling & protein processing |
| ENSG00000151553 | GPAA1 | 0,6408 | 1,237322 | 1,471258 | cell signaling & protein processing |
| ENSG00000172538 | GPI | 1,851985 | 1,553755 | 2,881413 | cell signaling & protein processing |
| ENSG00000183114 | GPR44 | -0,05644 | -0,26903 | 0,265301 | cell signaling & protein processing |
| ENSG00000071859 | GPS1 | 0,984084 | 1,042273 | 0,88984 | cell signaling & protein processing |
| ENSG00000189319 | GPS2 | 0,508589 | 0,250237 | 0,979796 | cell signaling & protein processing |
| ENSG00000180043 | GRB2 | -0,03481 | -0,04055 | 0,7967 | cell signaling & protein processing |
| ENSG00000187741 | GSK3A | 1,206481 | 1,40354 | 1,961201 | cell signaling & protein processing |
| ENSG00000144554 | GSK3B | -0,38672 | -0,12804 | 0,299063 | cell signaling & protein processing |
| ENSG00000179115 | GSPT1 | 1,304549 | 1,536653 | 1,992368 | cell signaling & protein processing |
| ENSG00000132004 | GZMM | -0,23524 | -0,51996 | 0,566286 | cell signaling & protein processing |
| ENSG00000182511 | HADH | 0,703008 | 0,385275 | -0,20334 | cell signaling & protein processing |
| ENSG00000163497 | HADHA | 1,371545 | 1,515034 | 1,30862 | cell signaling & protein processing |
| ENSG00000127084 | HADHB | 0,117433 | 0,242237 | 0,638232 | cell signaling & protein processing |
| ENSG00000004478 | HCFC1 | 2,249269 | 2,145853 | 1,86364 | cell signaling & protein processing |
| ENSG00000105701 | HCK | -1,01274 | -0,98611 | 0,876603 | cell signaling & protein processing |
| ENSG00000154803 | HCLS1 | -0,53287 | -0,45725 | 1,293277 | cell signaling & protein processing |
| ENSG00000196924 | HDAC1 | 0,330075 | 0,330649 | 0,501639 | cell signaling & protein processing |
| ENSG00000132589 | HDAC3 | 1,193067 | 1,621457 | 1,08631 | cell signaling & protein processing |
| ENSG00000162076 | HDAC6 | -0,1784 | -0,20888 | 0,382153 | cell signaling & protein processing |
| ENSG00000167363 | HDGF | 2,943285 | 3,172653 | 3,520583 | cell signaling & protein processing |
| ENSG00000168522 | HECTD1 | 0,141782 | -0,10841 | 0,302407 | cell signaling & protein processing |
| ENSG00000170345 | HECTD3 | 0,147757 | 0,75255 | 1,159631 | cell signaling & protein processing |
| ENSG00000075426 | HERC1 | 0,511558 | 0,14033 | 2,703283 | cell signaling & protein processing |
| ENSG00000204612 | HERC3 | -1,20224 | -0,50761 | 0,673937 | cell signaling & protein processing |
| ENSG00000164916 | HGS | 0,511575 | 0,642715 | 1,158253 | cell signaling & protein processing |
| ENSG00000118689 | HIF1A | 0,710207 | 0,763259 | 2,997173 | cell signaling & protein processing |
| ENSG00000171051 | HIP1 | -2,22226 | -2,47624 | -1,57939 | cell signaling & protein processing |
| ENSG00000181274 | HIPK2 | -0,21543 | -0,39928 | 1,541122 | cell signaling & protein processing |
| ENSG00000099860 | HLA-DMA | 0,555582 | -0,13835 | 0,563832 | cell signaling & protein processing |
| ENSG00000089597 | HMGA1 | 2,349515 | 2,266652 | 2,045224 | cell signaling & protein processing |
| ENSG00000167491 | HMGXB3 | 0,464579 | 0,222009 | 0,323616 | cell signaling & protein processing |
| ENSG00000111780 | HMHA1 | -0,28807 | -0,11279 | 1,590236 | cell signaling & protein processing |
| ENSG00000111780 | HMOX2 | -0,3745 | -0,68397 | 0,079601 | cell signaling & protein processing |
| ENSG00000057608 | HOMER3 | 0,326442 | 0,095425 | 0,726645 | cell signaling & protein processing |
| ENSG00000162676 | HP | -2,31322 | -1,60079 | 2,044272 | cell signaling & protein processing |
| ENSG00000125447 | HPS4 | 1,081227 | 0,631066 | 0,270812 | cell signaling & protein processing |
| ENSG00000005955 | HRAS | 0,691947 | 0,46913 | 0,939411 | cell signaling & protein processing |
| ENSG00000135821 | HSF1 | 0,745171 | 0,832862 | 1,074844 | cell signaling & protein processing |
| ENSG00000196743 | HSP90AA1 | 2,814285 | 3,197468 | 2,508311 | cell signaling & protein processing |
| ENSG00000087338 | HSP90AB1 | 2,070254 | 2,237733 | 0,857948 | cell signaling & protein processing |
| ENSG00000101216 | HSP90B1 | 3,048385 | 2,909708 | 2,489153 | cell signaling & protein processing |
| ENSG00000089639 | HSPA4 | -0,02549 | 0,00214 | -0,26358 | cell signaling & protein processing |
| ENSG00000137198 | HSPA8 | 4,062365 | 4,496873 | 3,797413 | cell signaling & protein processing |
| ENSG00000163655 | HSPA9 | 1,736692 | 1,51933 | 1,105319 | cell signaling & protein processing |
| ENSG00000146535 | HSPB1 | 1,756866 | 2,218378 | 1,186864 | cell signaling & protein processing |
| ENSG00000120063 | HSPBP1 | 0,143632 | 0,134069 | 0,311159 | cell signaling & protein processing |
| ENSG00000060558 | HSPD1 | 1,251503 | 1,363158 | -0,16699 | cell signaling & protein processing |
| ENSG00000156052 | HTRA1 | -0,35553 | -0,53125 | 0,265224 | cell signaling & protein processing |
| ENSG00000087460 | HTRA2 | 0,415052 | 0,756521 | 1,13352 | cell signaling & protein processing |
| ENSG00000078369 | HTRA3 | -0,19831 | -0,29221 | 0,644043 | cell signaling & protein processing |
| ENSG00000204628 | HUWE1 | 1,446388 | 1,300887 | 1,563605 | cell signaling & protein processing |
| ENSG00000135677 | ID2 | -0,09467 | -1,20065 | 1,031978 | cell signaling & protein processing |
| ENSG00000167110 | IDE | -0,09294 | 0,102234 | -0,46436 | cell signaling & protein processing |
| ENSG00000105220 | IFI30 | -0,62031 | -0,22142 | 1,347855 | cell signaling & protein processing |
| ENSG00000125734 | IFITM1 | -0,26667 | -0,30007 | 1,275023 | cell signaling & protein processing |
| ENSG00000180269 | IFNGR2 | 0,461921 | 0,494793 | 1,459947 | cell signaling & protein processing |
| ENSG00000158292 | IGF1R | 0,31585 | 0,358973 | 1,46631 | cell signaling & protein processing |
| ENSG00000170128 | IGF2R | -1,05824 | -0,50596 | 1,171451 | cell signaling & protein processing |
| ENSG00000132522 | IGSF6 | -1,23381 | -0,8264 | 1,235488 | cell signaling & protein processing |
| ENSG00000233276 | IL16 | -1,41289 | -1,48065 | 0,038229 | cell signaling & protein processing |
| ENSG00000167468 | IL17C | -0,1045 | -0,33275 | 0,195349 | cell signaling & protein processing |
| ENSG00000177885 | IL17RA | -0,28673 | 0,006261 | 2,699893 | cell signaling & protein processing |
| ENSG00000116032 | IL18RAP | -3,06464 | -2,43451 | 1,112563 | cell signaling & protein processing |
| ENSG00000228963 | IL8 | 0,47711 | 0,622657 | 3,389943 | cell signaling & protein processing |
| ENSG00000082701 | ILK | -0,11427 | 0,504137 | 1,331286 | cell signaling & protein processing |
| ENSG00000077809 | INPP5D | 1,54198 | 1,612301 | 2,205292 | cell signaling & protein processing |
| ENSG00000122034 | INPP5K | -0,27741 | -0,48862 | 1,337832 | cell signaling & protein processing |
| ENSG00000077235 | INPPL1 | 0,074672 | 0,026736 | 0,74223 | cell signaling & protein processing |
| ENSG00000155115 | INSR | -0,28683 | 0,113743 | -0,03698 | cell signaling & protein processing |
| ENSG00000143774 | IPO4 | 0,989534 | 1,006333 | 0,02273 | cell signaling & protein processing |
| ENSG00000143774 | IPO7 | 0,482683 | 0,510289 | -0,22301 | cell signaling & protein processing |
| ENSG00000143774 | IPO9 | -0,19402 | 0,446269 | 0,125967 | cell signaling & protein processing |
| ENSG00000170180 | IRAK1 | 0,255255 | 0,605751 | 0,614264 | cell signaling & protein processing |
| ENSG00000136732 | IRAK3 | -1,74114 | -1,64273 | 1,993968 | cell signaling & protein processing |
| ENSG00000197465 | IRF1 | 0,481171 | -0,28255 | 1,405287 | cell signaling & protein processing |
| ENSG00000188486 | IRF9 | -0,12335 | -0,48296 | 0,732454 | cell signaling & protein processing |
| ENSG00000113648 | IRS2 | 0,25944 | 0,406175 | 1,879572 | cell signaling & protein processing |
| ENSG00000163041 | ISOC2 | 1,082325 | 1,234036 | 0,677925 | cell signaling & protein processing |
| ENSG00000188375 | ITCH | 0,327569 | -0,15833 | 0,42053 | cell signaling & protein processing |
| ENSG00000103253 | ITGAL | -1,8003 | -1,8497 | 0,332699 | cell signaling & protein processing |
| ENSG00000134248 | ITSN2 | -0,84003 | -0,8657 | 0,477737 | cell signaling & protein processing |
| ENSG00000119285 | JAK1 | 0,742578 | 0,88128 | 1,53803 | cell signaling & protein processing |
| ENSG00000164818 | JAK2 | -1,06168 | -0,16865 | 0,033743 | cell signaling & protein processing |
| ENSG00000126107 | JMJD6 | -0,29757 | -0,50427 | 0,9667 | cell signaling & protein processing |
| ENSG00000103657 | JOSD1 | 0,945107 | 0,600568 | 0,675509 | cell signaling & protein processing |
| ENSG00000138641 | JOSD2 | -0,36274 | -0,34856 | 0,164693 | cell signaling & protein processing |
| ENSG00000144485 | JUN | 0,378418 | 0,385137 | 1,170919 | cell signaling & protein processing |
| ENSG00000186834 | JUNB | 1,779187 | 2,149203 | 3,675993 | cell signaling & protein processing |
| ENSG00000177374 | KATNB1 | -0,00085 | 0,090193 | 0,105423 | cell signaling & protein processing |
| ENSG00000112727 | KEAP1 | 0,598741 | 0,671107 | 0,443047 | cell signaling & protein processing |
| ENSG00000197153 | KEL | 2,001507 | 2,746088 | 2,705333 | cell signaling & protein processing |
| ENSG00000196176 | KHDRBS1 | 2,958055 | 2,828553 | 2,734203 | cell signaling & protein processing |
| ENSG00000156515 | KIAA0317 | 0,981145 | 0,986185 | 0,997479 | cell signaling & protein processing |
| ENSG00000189159 | KIDINS220 | -0,33249 | -0,14929 | 0,570818 | cell signaling & protein processing |
| ENSG00000092199 | KIT | 2,165599 | 0,463205 | -0,75781 | cell signaling & protein processing |
| ENSG00000138668 | KLF2 | 0,97086 | 0,779447 | 1,475877 | cell signaling & protein processing |
| ENSG00000169045 | KPNA1 | -0,63512 | -0,29379 | 0,254994 | cell signaling & protein processing |
| ENSG00000096746 | KPNA3 | 0,052518 | -0,0188 | -0,26812 | cell signaling & protein processing |
| ENSG00000165119 | KPNA6 | 0,588505 | 0,529508 | 1,215213 | cell signaling & protein processing |
| ENSG00000165119 | KPNB1 | 0,770783 | 0,862003 | 0,638906 | cell signaling & protein processing |
| ENSG00000099783 | KSR1 | -0,99934 | -1,09686 | 0,214574 | cell signaling & protein processing |
| ENSG00000107521 | LAT | -0,0739 | -0,63458 | 0,435872 | cell signaling & protein processing |
| ENSG00000100099 | LATS2 | -0,20217 | -0,32941 | 0,150006 | cell signaling & protein processing |
| ENSG00000207709 | LDHA | 1,592729 | 1,743112 | 3,088213 | cell signaling & protein processing |
| ENSG00000113013 | LIMK2 | -1,13 | -0,75233 | 0,008695 | cell signaling & protein processing |
| ENSG00000120694 | LIN7A | -2,70711 | -2,24796 | 0,241634 | cell signaling & protein processing |
| ENSG00000178922 | LMAN1 | 0,921307 | -0,01632 | -0,5448 | cell signaling & protein processing |
| ENSG00000149428 | LMAN2 | 2,505055 | 2,507828 | 2,408388 | cell signaling & protein processing |
| ENSG00000105376 | LMNA | 0,736682 | 1,02773 | 0,158468 | cell signaling & protein processing |
| ENSG00000182054 | LMTK2 | -0,72181 | -0,4374 | 0,649546 | cell signaling & protein processing |
| ENSG00000067829 | LONP1 | -0,1004 | 0,109067 | -0,34177 | cell signaling & protein processing |
| ENSG00000067064 | LONP2 | 0,452106 | 0,185746 | 0,678279 | cell signaling & protein processing |
| ENSG00000127415 | LPAR2 | -0,43327 | -0,57942 | 0,120838 | cell signaling & protein processing |
| ENSG00000137331 | LPCAT2 | -1,83781 | -1,72232 | 1,435923 | cell signaling & protein processing |
| ENSG00000188483 | LPIN2 | -0,15066 | 0,051206 | 1,82502 | cell signaling & protein processing |
| ENSG00000214706 | LRP10 | -0,28186 | 0,139702 | 2,729033 | cell signaling & protein processing |
| ENSG00000109083 | LRP3 | -0,4948 | -0,67183 | 0,028187 | cell signaling & protein processing |
| ENSG00000140443 | LRP8 | 0,371794 | 0,082237 | -0,06143 | cell signaling & protein processing |
| ENSG00000073792 | LRPAP1 | 1,351468 | 1,454041 | 2,149867 | cell signaling & protein processing |
| ENSG00000115590 | LSG1 | -0,02325 | 0,035973 | 0,007329 | cell signaling & protein processing |
| ENSG00000163464 | LSP1 | 0,099341 | 0,415317 | 1,979967 | cell signaling & protein processing |
| ENSG00000143621 | LST1 | 0,702751 | 0,4892 | 2,768503 | cell signaling & protein processing |
| ENSG00000143621 | LTA4H | 0,106109 | 0,552946 | 2,266382 | cell signaling & protein processing |
| ENSG00000143621 | LTB4R | -0,88738 | -0,88366 | 0,003994 | cell signaling & protein processing |
| ENSG00000143621 | LTF | 0,986328 | 2,287191 | 5,094793 | cell signaling & protein processing |
| ENSG00000166333 | LYN | -0,33403 | -0,36232 | 1,632926 | cell signaling & protein processing |
| ENSG00000136718 | LYST | -1,51682 | -1,26007 | 0,393802 | cell signaling & protein processing |
| ENSG00000169592 | MADD | 0,141432 | -0,14142 | 0,368801 | cell signaling & protein processing |
| ENSG00000132376 | MAFA | -0,07662 | -0,30702 | 0,095468 | cell signaling & protein processing |
| ENSG00000143624 | MAN1A1 | -0,84521 | -0,29055 | 2,294818 | cell signaling & protein processing |
| ENSG00000185085 | MAN2A1 | 0,070969 | -0,17824 | 1,213032 | cell signaling & protein processing |
| ENSG00000176095 | MAN2A2 | 2,240793 | 2,103938 | 2,839423 | cell signaling & protein processing |
| ENSG00000068745 | MAN2B1 | 0,023487 | 0,171773 | 1,411414 | cell signaling & protein processing |
| ENSG00000198700 | MAP2K1 | 0,256413 | 0,11135 | 0,759268 | cell signaling & protein processing |
| ENSG00000106012 | MAP2K2 | 1,798829 | 1,738887 | 1,588758 | cell signaling & protein processing |
| ENSG00000140575 | MAP2K3 | 0,041426 | 0,865917 | 3,080833 | cell signaling & protein processing |
| ENSG00000145703 | MAP3K1 | -0,30771 | -0,31125 | 0,397946 | cell signaling & protein processing |
| ENSG00000144711 | MAP3K11 | -0,04336 | -0,07834 | 0,790828 | cell signaling & protein processing |
| ENSG00000120645 | MAP3K2 | -1,574 | -1,22557 | 0,152805 | cell signaling & protein processing |
| ENSG00000143164 | MAP3K3 | -0,63098 | -0,82691 | 0,382345 | cell signaling & protein processing |
| ENSG00000184216 | MAP3K4 | 0,644134 | 0,563906 | 0,377128 | cell signaling & protein processing |
| ENSG00000090376 | MAP3K5 | -0,10312 | 0,03711 | 0,040707 | cell signaling & protein processing |
| ENSG00000168310 | MAP3K8 | -0,69898 | -0,75612 | 0,385625 | cell signaling & protein processing |
| ENSG00000170604 | MAP4K4 | -0,31051 | 0,211825 | 0,722489 | cell signaling & protein processing |
| ENSG00000168264 | MAP4K5 | -0,05331 | -0,82209 | 0,461174 | cell signaling & protein processing |
| ENSG00000185507 | MAPK1 | 0,453091 | 1,116785 | 2,384759 | cell signaling & protein processing |
| ENSG00000140968 | MAPK14 | -0,34239 | -0,34944 | 0,8961 | cell signaling & protein processing |
| ENSG00000185950 | MAPK3 | -0,15226 | -0,02108 | 1,102017 | cell signaling & protein processing |
| ENSG00000165898 | MAPK8IP3 | -0,24719 | -0,20801 | 0,415402 | cell signaling & protein processing |
| ENSG00000136003 | MAPKAPK2 | 0,110108 | -0,088 | 1,366485 | cell signaling & protein processing |
| ENSG00000172183 | MAPKAPK3 | 0,373107 | 0,406058 | 1,493582 | cell signaling & protein processing |
| ENSG00000078747 | MARCH2 | -0,27144 | -0,31992 | 1,567118 | cell signaling & protein processing |
| ENSG00000167930 | MARCH3 | -0,74663 | -0,0956 | 2,872043 | cell signaling & protein processing |
| ENSG00000161638 | MARCH8 | 0,343945 | 0,779274 | 3,522063 | cell signaling & protein processing |
| ENSG00000169896 | MARK2 | -0,39949 | -0,09403 | 0,670777 | cell signaling & protein processing |
| ENSG00000140678 | MARK3 | 0,021531 | 0,296437 | 2,027593 | cell signaling & protein processing |
| ENSG00000077009 | MAST2 | -0,35074 | -0,34762 | 0,175862 | cell signaling & protein processing |
| ENSG00000136156 | MASTL | -0,11281 | 0,042623 | 0,527508 | cell signaling & protein processing |
| ENSG00000198399 | MAX | -0,04679 | -0,33111 | 1,274517 | cell signaling & protein processing |
| ENSG00000210082 | MBTPS1 | 0,196244 | 0,08552 | 0,658259 | cell signaling & protein processing |
| ENSG00000211459 | MCL1 | 1,242523 | 1,411873 | 3,248893 | cell signaling & protein processing |
| ENSG00000171988 | MCOLN1 | -0,18022 | 0,087514 | 1,319601 | cell signaling & protein processing |
| ENSG00000070495 | MCRS1 | 0,353826 | 0,328398 | 0,345443 | cell signaling & protein processing |
| ENSG00000161999 | MDFIC | -0,20801 | -0,30711 | 0,344364 | cell signaling & protein processing |
| ENSG00000161677 | MDM2 | 0,195265 | 0,104547 | 0,823845 | cell signaling & protein processing |
| ENSG00000092051 | MDM4 | 0,953677 | 0,203691 | 0,821608 | cell signaling & protein processing |
| ENSG00000167476 | MDN1 | 0,527559 | 0,431007 | -0,3838 | cell signaling & protein processing |
| ENSG00000143543 | MED1 | -0,13325 | 0,145915 | 0,031828 | cell signaling & protein processing |
| ENSG00000065427 | MEF2A | -0,32384 | -0,4377 | 0,380704 | cell signaling & protein processing |
| ENSG00000065427 | METAP2 | 1,751411 | 1,85966 | 0,470604 | cell signaling & protein processing |
| ENSG00000104783 | MGAT3 | 0,864344 | 1,10686 | 1,379502 | cell signaling & protein processing |
| ENSG00000237941 | MGAT4B | 1,520339 | 1,424922 | 0,939561 | cell signaling & protein processing |
| ENSG00000004487 | MICALL2 | 0,319199 | 0,593558 | 0,614092 | cell signaling & protein processing |
| ENSG00000120733 | MID1IP1 | -0,19539 | 0,136027 | 1,821412 | cell signaling & protein processing |
| ENSG00000117139 | MINK1 | -0,04096 | 0,112986 | 0,792326 | cell signaling & protein processing |
| ENSG00000132510 | MKI67IP | 0,451657 | 0,574198 | 0,253743 | cell signaling & protein processing |
| ENSG00000121774 | MKNK1 | 0,153773 | 0,126728 | 1,10089 | cell signaling & protein processing |
| ENSG00000121774 | MKNK2 | 1,262593 | 2,025401 | 2,877363 | cell signaling & protein processing |
| ENSG00000121774 | MLEC | 0,006135 | 0,16211 | -0,20408 | cell signaling & protein processing |
| ENSG00000121774 | MLL | 0,526996 | 0,775153 | 0,788817 | cell signaling & protein processing |
| ENSG00000121774 | MLL3 | 1,155791 | 1,070641 | 1,779327 | cell signaling & protein processing |
| ENSG00000100441 | MLST8 | 0,981771 | 0,811624 | 0,781079 | cell signaling & protein processing |
| ENSG00000226777 | MME | -2,27232 | -1,96401 | 0,01496 | cell signaling & protein processing |
| ENSG00000081791 | MMP25 | -0,48274 | -0,1915 | 1,020694 | cell signaling & protein processing |
| ENSG00000182149 | MMP8 | -0,46318 | 1,220591 | 4,793243 | cell signaling & protein processing |
| ENSG00000131149 | MMP9 | 0,619537 | 1,557727 | 4,460793 | cell signaling & protein processing |
| ENSG00000166398 | MOBKL1A | -0,13361 | -0,07407 | 1,988338 | cell signaling & protein processing |
| ENSG00000166783 | MOGS | 1,288744 | 1,213607 | 0,625263 | cell signaling & protein processing |
| ENSG00000159658 | MON2 | -1,08159 | -0,54499 | 0,206593 | cell signaling & protein processing |
| ENSG00000135709 | MORC3 | -0,99877 | -0,77622 | 0,684701 | cell signaling & protein processing |
| ENSG00000214655 | MPDU1 | 2,165036 | 2,281405 | 1,82863 | cell signaling & protein processing |
| ENSG00000005238 | MPV17 | 0,444922 | 0,517555 | 0,100701 | cell signaling & protein processing |
| ENSG00000111615 | MSN | 2,049295 | 1,921874 | 3,292803 | cell signaling & protein processing |
| ENSG00000111057 | MSRA | -0,93298 | -0,98974 | 0,405146 | cell signaling & protein processing |
| ENSG00000141068 | MTMR3 | -0,04269 | 0,419349 | 2,570492 | cell signaling & protein processing |
| ENSG00000100395 | MTMR4 | 0,161914 | 0,274862 | 0,464349 | cell signaling & protein processing |
| ENSG00000167613 | MTOR | 0,033583 | 0,142173 | 0,194849 | cell signaling & protein processing |
| ENSG00000132434 | MTX1 | 0,333874 | 0,440734 | 0,339712 | cell signaling & protein processing |
| ENSG00000143418 | MXI1 | 0,035474 | 0,437064 | 1,666207 | cell signaling & protein processing |
| ENSG00000188779 | MYC | 2,287605 | 1,903519 | 0,140081 | cell signaling & protein processing |
| ENSG00000172155 | MYD88 | 0,191777 | 0,35892 | 1,574111 | cell signaling & protein processing |
| ENSG00000186226 | MYH10 | 0,4479 | 0,678829 | -0,34274 | cell signaling & protein processing |
| ENSG00000240386 | MYH9 | 1,056786 | 1,408656 | 2,973563 | cell signaling & protein processing |
| ENSG00000187173 | MYL12B | 1,598163 | 1,970953 | 2,632713 | cell signaling & protein processing |
| ENSG00000136167 | MYLIP | 0,214458 | 0,414861 | 2,190483 | cell signaling & protein processing |
| ENSG00000134333 | MYO1F | -1,37456 | -0,37181 | 2,362477 | cell signaling & protein processing |
| ENSG00000243709 | MYSM1 | 0,622741 | 0,177666 | 0,192581 | cell signaling & protein processing |
| ENSG00000168924 | NACA | 2,724915 | 2,571813 | 1,880253 | cell signaling & protein processing |
| ENSG00000133317 | NADK | 0,132192 | 0,343801 | 1,325025 | cell signaling & protein processing |
| ENSG00000121897 | NAPA | 0,275328 | 0,614521 | 1,42629 | cell signaling & protein processing |
| ENSG00000187116 | NASP | 0,878185 | 0,5217 | -0,23268 | cell signaling & protein processing |
| ENSG00000105983 | NCCRP1 | 0,330833 | -0,30984 | 0,184697 | cell signaling & protein processing |
| ENSG00000100258 | NCF2 | -1,59955 | -0,9727 | 2,066562 | cell signaling & protein processing |
| ENSG00000176619 | NCKAP1L | -0,0682 | -0,19022 | 1,270295 | cell signaling & protein processing |
| ENSG00000164715 | NCLN | 1,063574 | 1,061344 | 1,062574 | cell signaling & protein processing |
| ENSG00000139517 | NCOA2 | -0,08831 | -0,01308 | 0,616751 | cell signaling & protein processing |
| ENSG00000102910 | NCOR1 | 1,640521 | 1,238188 | 1,450557 | cell signaling & protein processing |
| ENSG00000129038 | NCOR2 | 0,623922 | 0,470189 | 0,64309 | cell signaling & protein processing |
| ENSG00000153395 | NCSTN | 0,183853 | 0,025015 | 0,378231 | cell signaling & protein processing |
| ENSG00000101577 | NDFIP1 | -0,40536 | -0,26918 | 0,126689 | cell signaling & protein processing |
| ENSG00000198589 | NDRG1 | -0,41807 | -0,06813 | 1,096542 | cell signaling & protein processing |
| ENSG00000077454 | NDST1 | -0,80106 | -0,84075 | 0,524487 | cell signaling & protein processing |
| ENSG00000177595 | NDST2 | -0,05047 | 0,175268 | 1,416381 | cell signaling & protein processing |
| ENSG00000171236 | NDUFA10 | 0,351358 | 0,346853 | 0,050113 | cell signaling & protein processing |
| ENSG00000198799 | NDUFA12 | 0,584111 | 0,340766 | 0,228029 | cell signaling & protein processing |
| ENSG00000118308 | NDUFA2 | 1,593479 | 1,982222 | 1,625772 | cell signaling & protein processing |
| ENSG00000197324 | NDUFA3 | 1,372522 | 1,099667 | 1,212728 | cell signaling & protein processing |
| ENSG00000130881 | NDUFA6 | 0,724493 | 0,993385 | 0,896746 | cell signaling & protein processing |
| ENSG00000157193 | NDUFA7 | 0,961973 | 0,7532 | 0,886435 | cell signaling & protein processing |
| ENSG00000163956 | NDUFA9 | 0,115586 | 0,251834 | 0,401898 | cell signaling & protein processing |
| ENSG00000177021 | NDUFB11 | 2,260779 | 2,139431 | 1,697462 | cell signaling & protein processing |
| ENSG00000175489 | NDUFB7 | 0,706287 | 0,421161 | 0,76606 | cell signaling & protein processing |
| ENSG00000184709 | NDUFB8 | 1,68417 | 1,688839 | 1,223056 | cell signaling & protein processing |
| ENSG00000174004 | NDUFB9 | 1,176436 | 1,29252 | 0,693958 | cell signaling & protein processing |
| ENSG00000185158 | NDUFC2 | 1,086789 | 1,291449 | 0,661048 | cell signaling & protein processing |
| ENSG00000132128 | NDUFS2 | 0,40684 | 0,203542 | 0,654889 | cell signaling & protein processing |
| ENSG00000116212 | NDUFS3 | 0,500228 | 0,382887 | 0,302493 | cell signaling & protein processing |
| ENSG00000130764 | NDUFS5 | 2,975805 | 2,580988 | 1,394329 | cell signaling & protein processing |
| ENSG00000180979 | NDUFS6 | 0,186616 | 0,010406 | 0,005953 | cell signaling & protein processing |
| ENSG00000163428 | NDUFS7 | 0,673187 | 0,811882 | 0,77879 | cell signaling & protein processing |
| ENSG00000108829 | NDUFS8 | 0,914866 | 0,68611 | 0,791149 | cell signaling & protein processing |
| ENSG00000104866 | NDUFV1 | 1,379462 | 1,458776 | 0,794606 | cell signaling & protein processing |
| ENSG00000136802 | NDUFV3 | 0,896884 | 1,018359 | 1,023185 | cell signaling & protein processing |
| ENSG00000093167 | NECAP1 | 1,474802 | 1,001343 | 1,537572 | cell signaling & protein processing |
| ENSG00000161036 | NECAP2 | 1,390222 | 1,372183 | 0,96623 | cell signaling & protein processing |
| ENSG00000041802 | NEDD8 | 0,259468 | 0,655028 | 0,161426 | cell signaling & protein processing |
| ENSG00000170860 | NET1 | 1,319872 | 0,526525 | 0,154022 | cell signaling & protein processing |
| ENSG00000183011 | NFAM1 | 0,052239 | -0,00161 | 1,258151 | cell signaling & protein processing |
| ENSG00000160285 | NFATC1 | 0,036857 | -0,27535 | 0,098853 | cell signaling & protein processing |
| ENSG00000213903 | NFE2 | -0,31362 | 0,398597 | 1,835274 | cell signaling & protein processing |
| ENSG00000147507 | NFKBIA | 2,952725 | 2,516024 | 3,730603 | cell signaling & protein processing |
| ENSG00000011009 | NFKBIB | 0,312933 | 0,239054 | 0,625249 | cell signaling & protein processing |
| ENSG00000090382 | NFKBIE | -0,6315 | -0,628 | 0,295633 | cell signaling & protein processing |
| ENSG00000116670 | NFYC | -0,35416 | 0,104243 | 0,460239 | cell signaling & protein processing |
| ENSG00000197063 | NIPBL | -0,05781 | -0,20292 | 0,620525 | cell signaling & protein processing |
| ENSG00000177383 | NKTR | 0,863392 | 0,237463 | 0,359867 | cell signaling & protein processing |
| ENSG00000162889 | NPEPL1 | -0,14326 | -0,48309 | 0,15705 | cell signaling & protein processing |
| ENSG00000114738 | NPHS1 | -2,37209 | -2,57548 | -1,90023 | cell signaling & protein processing |
| ENSG00000109270 | NPLOC4 | 1,055101 | 1,190748 | 2,097271 | cell signaling & protein processing |
| ENSG00000101367 | NPM1 | -1,54685 | -1,66528 | -1,30858 | cell signaling & protein processing |
| ENSG00000155130 | NR1H2 | 0,196131 | 0,495323 | 1,330901 | cell signaling & protein processing |
| ENSG00000166986 | NR4A2 | -1,08426 | -1,28489 | 0,557853 | cell signaling & protein processing |
| ENSG00000120539 | NRD1 | 0,596015 | 1,08122 | 1,171698 | cell signaling & protein processing |
| ENSG00000125952 | NRTN | -0,00669 | -0,28391 | 0,222423 | cell signaling & protein processing |
| ENSG00000198625 | NUP153 | 1,631077 | 1,125477 | 1,668134 | cell signaling & protein processing |
| ENSG00000112159 | NUP155 | 0,258202 | 0,244464 | -0,03279 | cell signaling & protein processing |
| ENSG00000151376 | NUP188 | 0,388135 | 0,608854 | -0,01737 | cell signaling & protein processing |
| ENSG00000124733 | NUP210 | 1,947645 | 2,167353 | 1,336694 | cell signaling & protein processing |
| ENSG00000169057 | NUP214 | 1,36269 | 1,173023 | 1,312334 | cell signaling & protein processing |
| ENSG00000125686 | NUP43 | 0,188264 | 0,330487 | -0,80236 | cell signaling & protein processing |
| ENSG00000184634 | NUP50 | -0,80769 | -0,69665 | 0,136984 | cell signaling & protein processing |
| ENSG00000108510 | NUP54 | 0,055795 | -0,39548 | -0,71648 | cell signaling & protein processing |
| ENSG00000123066 | NUP85 | 1,226301 | 1,041264 | 0,595631 | cell signaling & protein processing |
| ENSG00000180182 | NUP88 | 1,238035 | 1,037386 | 1,578455 | cell signaling & protein processing |
| ENSG00000099917 | NUP93 | 1,141312 | 1,022695 | 0,321576 | cell signaling & protein processing |
| ENSG00000175221 | NUP98 | 1,152875 | 0,943034 | 1,949397 | cell signaling & protein processing |
| ENSG00000152944 | NUPL1 | 0,204015 | -0,00465 | 0,160986 | cell signaling & protein processing |
| ENSG00000008838 | NUTF2 | 2,771885 | 2,621853 | 2,2632 | cell signaling & protein processing |
| ENSG00000118579 | NXT1 | 0,150148 | -0,08091 | 0,3656 | cell signaling & protein processing |
| ENSG00000165792 | OLR1 | -2,41673 | -2,06071 | 1,037174 | cell signaling & protein processing |
| ENSG00000176624 | OPTN | -0,44314 | -0,42736 | 0,213045 | cell signaling & protein processing |
| ENSG00000164877 | OTUB1 | 1,198642 | 0,714133 | 1,229642 | cell signaling & protein processing |
| ENSG00000214922 | OTUD5 | -0,22233 | -0,0223 | 0,925303 | cell signaling & protein processing |
| ENSG00000167470 | OXA1L | 2,109469 | 2,097131 | 1,982869 | cell signaling & protein processing |
| ENSG00000198160 | OXSR1 | -0,18961 | 0,109531 | 0,654434 | cell signaling & protein processing |
| ENSG00000196588 | PA2G4 | 2,849175 | 2,657703 | 2,401057 | cell signaling & protein processing |
| ENSG00000128585 | PABPC1 | 3,043525 | 2,955188 | 3,333363 | cell signaling & protein processing |
| ENSG00000133606 | PACS1 | 0,225611 | 0,027921 | 0,568162 | cell signaling & protein processing |
| ENSG00000110917 | PADI2 | -1,00996 | -0,80117 | 1,823307 | cell signaling & protein processing |
| ENSG00000151725 | PADI4 | -1,4113 | -0,60005 | 2,588479 | cell signaling & protein processing |
| ENSG00000167548 | PAG1 | -1,73079 | -1,94257 | 1,199175 | cell signaling & protein processing |
| ENSG00000130382 | PAK1 | -0,23112 | 0,430701 | 0,482848 | cell signaling & protein processing |
| ENSG00000171843 | PAK4 | 0,089085 | -0,03057 | 0,114496 | cell signaling & protein processing |
| ENSG00000108292 | PALM | 0,111762 | -0,10751 | 0,363025 | cell signaling & protein processing |
| ENSG00000100985 | PARK7 | -1,20596 | -1,37703 | -1,91069 | cell signaling & protein processing |
| ENSG00000155229 | PARP1 | 0,41472 | 0,052587 | -0,58905 | cell signaling & protein processing |
| ENSG00000130675 | PARP4 | 0,69475 | 0,302467 | 1,069393 | cell signaling & protein processing |
| ENSG00000186205 | PCCB | 1,18717 | 0,741826 | -0,01792 | cell signaling & protein processing |
| ENSG00000051825 | PCMT1 | 0,506824 | 0,993618 | 1,580588 | cell signaling & protein processing |
| ENSG00000005381 | PCMTD1 | -2,3708 | -1,5768 | 0,493094 | cell signaling & protein processing |
| ENSG00000130830 | PCMTD2 | -1,55969 | -1,17494 | 0,106919 | cell signaling & protein processing |
| ENSG00000183093 | PCSK7 | 0,151693 | 0,351023 | 0,409814 | cell signaling & protein processing |
| ENSG00000197345 | PDCD6IP | 0,579414 | 0,522718 | 0,505793 | cell signaling & protein processing |
| ENSG00000086504 | PDIA4 | 0,457155 | 0,378411 | -0,19563 | cell signaling & protein processing |
| ENSG00000114686 | PDIA6 | 0,798153 | 0,473766 | -0,38401 | cell signaling & protein processing |
| ENSG00000171421 | PDLIM7 | -0,15339 | -0,31381 | 0,577821 | cell signaling & protein processing |
| ENSG00000116221 | PDPK1 | -0,11665 | -0,05766 | 1,372814 | cell signaling & protein processing |
| ENSG00000111639 | PDXP | 0,616772 | 1,04907 | 1,331409 | cell signaling & protein processing |
| ENSG00000162910 | PDZD8 | 1,345953 | 1,156669 | 1,654939 | cell signaling & protein processing |
| ENSG00000096080 | PEPD | 0,201803 | 0,193193 | -0,26203 | cell signaling & protein processing |
| ENSG00000131368 | PEX26 | 0,293217 | 0,130718 | 0,323702 | cell signaling & protein processing |
| ENSG00000074071 | PFDN1 | 0,617238 | 0,433099 | 0,370309 | cell signaling & protein processing |
| ENSG00000144029 | PFDN2 | 1,315037 | 1,137012 | 1,274933 | cell signaling & protein processing |
| ENSG00000125445 | PFDN6 | 0,883663 | 0,550767 | -0,04209 | cell signaling & protein processing |
| ENSG00000149516 | PFKL | 1,038312 | 1,036378 | 1,065418 | cell signaling & protein processing |
| ENSG00000135097 | PFN1 | 3,616885 | 3,599913 | 4,457043 | cell signaling & protein processing |
| ENSG00000188895 | PGAP2 | 0,186109 | 0,027364 | -0,0658 | cell signaling & protein processing |
| ENSG00000162006 | PGK1 | 1,209798 | 1,41763 | 2,797833 | cell signaling & protein processing |
| ENSG00000100330 | PHIP | 1,271597 | 0,624388 | 1,085994 | cell signaling & protein processing |
| ENSG00000108389 | PHKA2 | -0,17122 | -0,35299 | 0,233713 | cell signaling & protein processing |
| ENSG00000105887 | PHKG2 | 0,288028 | 0,712281 | 1,067551 | cell signaling & protein processing |
| ENSG00000213347 | PI4KB | 0,528764 | 0,42984 | 0,806075 | cell signaling & protein processing |
| ENSG00000162576 | PICALM | 0,789874 | 1,439453 | 1,958404 | cell signaling & protein processing |
| ENSG00000179820 | PIGQ | 0,726639 | 1,441217 | 2,291653 | cell signaling & protein processing |
| ENSG00000118513 | PIGS | 0,892568 | 0,706569 | 0,981174 | cell signaling & protein processing |
| ENSG00000132382 | PIGT | 0,639517 | 0,623638 | 0,897829 | cell signaling & protein processing |
| ENSG00000172936 | PIK3CD | -1,04216 | -0,88229 | 0,502601 | cell signaling & protein processing |
| ENSG00000133026 | PIK3CG | -1,03197 | -0,7522 | 2,046126 | cell signaling & protein processing |
| ENSG00000092841 | PIK3R4 | -0,12048 | 0,21886 | -0,11237 | cell signaling & protein processing |
| ENSG00000229596 | PIKFYVE | 0,03304 | -0,52715 | 0,048752 | cell signaling & protein processing |
| ENSG00000141140 | PIM1 | 0,278452 | 1,143462 | 4,226263 | cell signaling & protein processing |
| ENSG00000142347 | PIM2 | -0,29292 | -0,38477 | 1,310457 | cell signaling & protein processing |
| ENSG00000136286 | PIM3 | -0,19739 | -0,47558 | 0,224977 | cell signaling & protein processing |
| ENSG00000099331 | PIN1 | 0,641048 | 0,509241 | 0,673606 | cell signaling & protein processing |
| ENSG00000162601 | PIP5K1C | -0,24042 | -0,39147 | 0,028764 | cell signaling & protein processing |
| ENSG00000136504 | PITPNM1 | -1,01326 | -1,21031 | 0,466702 | cell signaling & protein processing |
| ENSG00000136504 | PITRM1 | 0,987062 | 0,644363 | 0,596604 | cell signaling & protein processing |
| ENSG00000136504 | PKD1 | -0,54903 | -0,28785 | 0,011165 | cell signaling & protein processing |
| ENSG00000136504 | PKLR | 1,268113 | 1,627613 | -0,2029 | cell signaling & protein processing |
| ENSG00000136504 | PKMYT1 | 0,1847 | 0,611707 | 0,658407 | cell signaling & protein processing |
| ENSG00000083168 | PKN1 | 0,802242 | 0,356765 | 1,236651 | cell signaling & protein processing |
| ENSG00000156650 | PKN2 | -0,13271 | -0,21136 | 0,710493 | cell signaling & protein processing |
| ENSG00000160877 | PLAUR | -0,82611 | -0,44375 | 1,799341 | cell signaling & protein processing |
| ENSG00000172890 | PLCB2 | -0,73553 | -0,78314 | 1,12993 | cell signaling & protein processing |
| ENSG00000124357 | PLCG2 | -1,033 | -1,12171 | 1,035409 | cell signaling & protein processing |
| ENSG00000108784 | PLCH2 | -0,38722 | -0,5123 | 0,042903 | cell signaling & protein processing |
| ENSG00000161653 | PLCL2 | -2,21904 | -2,35537 | 0,049654 | cell signaling & protein processing |
| ENSG00000187109 | PLDN | -0,20158 | -0,51262 | 0,226816 | cell signaling & protein processing |
| ENSG00000105402 | PLEK | -4,57632 | -4,61408 | -3,64006 | cell signaling & protein processing |
| ENSG00000147813 | PLEK2 | -0,92412 | -0,7598 | 0,60184 | cell signaling & protein processing |
| ENSG00000134440 | PLEKHM1 | -2,18382 | -1,77117 | 0,04751 | cell signaling & protein processing |
| ENSG00000134440 | PLEKHM3 | -0,37991 | -0,67061 | 0,513782 | cell signaling & protein processing |
| ENSG00000134440 | PLK1 | 1,012142 | 1,740949 | 1,367717 | cell signaling & protein processing |
| ENSG00000134440 | PLK3 | -0,95772 | -1,429 | 0,148211 | cell signaling & protein processing |
| ENSG00000134440 | PLK4 | 0,251506 | 0,123679 | -0,25045 | cell signaling & protein processing |
| ENSG00000134440 | PLOD3 | 0,023908 | 0,406373 | 0,363541 | cell signaling & protein processing |
| ENSG00000134440 | PLP2 | 1,576838 | 1,79282 | 3,398393 | cell signaling & protein processing |
| ENSG00000121579 | PML | 0,500705 | 0,126281 | 0,002846 | cell signaling & protein processing |
| ENSG00000122390 | PMPCA | 0,036167 | -0,3075 | -0,23004 | cell signaling & protein processing |
| ENSG00000151779 | PMPCB | 0,253841 | 0,27171 | 0,436674 | cell signaling & protein processing |
| ENSG00000158747 | PNKP | 0,881525 | 0,849257 | 1,229043 | cell signaling & protein processing |
| ENSG00000100365 | POLA2 | 0,41011 | 0,268199 | -0,07907 | cell signaling & protein processing |
| ENSG00000188566 | POLR2B | 0,537779 | 0,330284 | 0,010868 | cell signaling & protein processing |
| ENSG00000104419 | POLR2C | 0,099631 | -0,08693 | 0,027037 | cell signaling & protein processing |
| ENSG00000070614 | POLR2E | 1,735542 | 1,630723 | 1,305682 | cell signaling & protein processing |
| ENSG00000166507 | POLR2F | 0,631584 | 0,708456 | 0,467489 | cell signaling & protein processing |
| ENSG00000130414 | POLR2H | 0,060599 | 0,870182 | -0,14127 | cell signaling & protein processing |
| ENSG00000184752 | POLR2J | -0,21398 | -0,29109 | 0,14725 | cell signaling & protein processing |
| ENSG00000131495 | POLR2L | 2,366485 | 2,347468 | 2,388065 | cell signaling & protein processing |
| ENSG00000178057 | POMP | 1,12493 | 1,093427 | 0,9875 | cell signaling & protein processing |
| ENSG00000110717 | PPIB | 2,786865 | 2,445548 | 2,539218 | cell signaling & protein processing |
| ENSG00000167792 | PPIF | -0,15868 | 0,104689 | 0,345681 | cell signaling & protein processing |
| ENSG00000160194 | PPIL1 | 0,157582 | 0,123191 | -0,49437 | cell signaling & protein processing |
| ENSG00000123119 | PPIL2 | 0,28449 | 0,035108 | 0,152953 | cell signaling & protein processing |
| ENSG00000089818 | PPM1A | 0,492014 | -0,3291 | 0,608946 | cell signaling & protein processing |
| ENSG00000157191 | PPM1D | -0,07193 | -0,47157 | 0,031728 | cell signaling & protein processing |
| ENSG00000129559 | PPM1F | -0,75703 | -0,79978 | 0,103081 | cell signaling & protein processing |
| ENSG00000117691 | PPM1G | 1,22749 | 1,116597 | 0,65784 | cell signaling & protein processing |
| ENSG00000186575 | PPP1CA | 1,511916 | 1,739941 | 1,925138 | cell signaling & protein processing |
| ENSG00000235568 | PPP1CB | 0,475246 | 0,508929 | 1,449426 | cell signaling & protein processing |
| ENSG00000102908 | PPP1CC | 1,734339 | 1,59313 | 1,576234 | cell signaling & protein processing |
| ENSG00000131196 | PPP1R10 | -0,27095 | 0,199974 | 0,715824 | cell signaling & protein processing |
| ENSG00000176953 | PPP1R12A | 1,314625 | 1,052684 | 1,848561 | cell signaling & protein processing |
| ENSG00000116044 | PPP1R15B | 0,940046 | 1,217382 | 2,933373 | cell signaling & protein processing |
| ENSG00000104825 | PPP2CA | 0,801002 | 0,89556 | 1,927892 | cell signaling & protein processing |
| ENSG00000167604 | PPP2R1A | 1,738742 | 1,342914 | 1,426215 | cell signaling & protein processing |
| ENSG00000146232 | PPP2R2A | -0,79634 | -1,18377 | 0,005639 | cell signaling & protein processing |
| ENSG00000151092 | PPP3CA | -0,45153 | -0,43139 | 0,117643 | cell signaling & protein processing |
| ENSG00000100138 | PPP3R1 | -1,30461 | -1,65844 | -1,25722 | cell signaling & protein processing |
| ENSG00000171840 | PPP4R2 | -0,3125 | -0,41667 | 0,158892 | cell signaling & protein processing |
| ENSG00000132603 | PPP6C | 0,978 | 0,823862 | 1,48943 | cell signaling & protein processing |
| ENSG00000105374 | PPT1 | 0,174459 | 0,441306 | 1,340084 | cell signaling & protein processing |
| ENSG00000114857 | PPT2 | 0,4127 | 0,649785 | 0,061758 | cell signaling & protein processing |
| ENSG00000136448 | PRDX2 | -0,30753 | 0,202068 | 0,428842 | cell signaling & protein processing |
| ENSG00000112992 | PRDX3 | 1,01917 | 1,536892 | 0,648504 | cell signaling & protein processing |
| ENSG00000141101 | PRDX5 | -0,19594 | 0,150457 | 0,989923 | cell signaling & protein processing |
| ENSG00000140939 | PREB | -0,10743 | 0,119785 | -0,04668 | cell signaling & protein processing |
| ENSG00000147140 | PRKACA | -0,42229 | -0,41008 | 0,281813 | cell signaling & protein processing |
| ENSG00000147140 | PRKAG1 | -0,00852 | 0,651373 | 0,306271 | cell signaling & protein processing |
| ENSG00000147140 | PRKAR1B | -0,07102 | 0,063163 | -0,58548 | cell signaling & protein processing |
| ENSG00000147140 | PRKAR2A | -0,14429 | -0,22771 | 0,003649 | cell signaling & protein processing |
| ENSG00000147140 | PRKAR2B | 1,79859 | 1,576574 | 0,774779 | cell signaling & protein processing |
| ENSG00000147140 | PRKCB | -0,69932 | -0,70973 | 1,571204 | cell signaling & protein processing |
| ENSG00000147140 | PRKCD | -1,26456 | -0,81925 | 1,869165 | cell signaling & protein processing |
| ENSG00000147140 | PRKCSH | 2,588185 | 2,531373 | 2,427929 | cell signaling & protein processing |
| ENSG00000147140 | PRKD2 | -1,08162 | -0,62886 | 0,538332 | cell signaling & protein processing |
| ENSG00000147140 | PRKDC | 1,115189 | 1,01645 | 0,301299 | cell signaling & protein processing |
| ENSG00000147140 | PRMT1 | 0,643993 | 0,54411 | -0,06855 | cell signaling & protein processing |
| ENSG00000111641 | PROK2 | -0,45496 | -0,31675 | 1,875448 | cell signaling & protein processing |
| ENSG00000198929 | PRPF19 | 2,222108 | 2,143266 | 1,107646 | cell signaling & protein processing |
| ENSG00000149308 | PRPF40A | 0,902609 | 0,680701 | 0,585361 | cell signaling & protein processing |
| ENSG00000183979 | PRPF4B | 0,663915 | 0,363549 | 0,669659 | cell signaling & protein processing |
| ENSG00000124588 | PRSS21 | 0,024932 | 0,039247 | 0,465542 | cell signaling & protein processing |
| ENSG00000131408 | PRSS27 | 0,203802 | -0,05437 | 0,302591 | cell signaling & protein processing |
| ENSG00000177463 | PRSS33 | -0,22533 | -0,59728 | 0,007243 | cell signaling & protein processing |
| ENSG00000160113 | PRSS42 | 0,187336 | 0,071949 | 0,497674 | cell signaling & protein processing |
| ENSG00000153234 | PRTN3 | 1,388829 | 1,968159 | 3,224243 | cell signaling & protein processing |
| ENSG00000158458 | PSKH1 | 0,091622 | 0,035672 | 0,369703 | cell signaling & protein processing |
| ENSG00000154146 | PSMA1 | 0,511412 | 0,307757 | 0,075845 | cell signaling & protein processing |
| ENSG00000171119 | PSMA2 | 0,539221 | 0,590382 | 0,710682 | cell signaling & protein processing |
| ENSG00000165671 | PSMA6 | 0,188446 | -0,02429 | -0,07836 | cell signaling & protein processing |
| ENSG00000088833 | PSMA7 | 0,111888 | 0,600691 | 0,916087 | cell signaling & protein processing |
| ENSG00000117697 | PSMB1 | 0,124262 | -0,34835 | -0,36765 | cell signaling & protein processing |
| ENSG00000107672 | PSMB10 | -0,65802 | -0,82371 | 0,048595 | cell signaling & protein processing |
| ENSG00000037474 | PSMB2 | 1,87463 | 1,698844 | 1,47286 | cell signaling & protein processing |
| ENSG00000130305 | PSMB3 | 1,202671 | 1,410919 | 1,311083 | cell signaling & protein processing |
| ENSG00000125458 | PSMB4 | 1,850704 | 1,364658 | 1,8872 | cell signaling & protein processing |
| ENSG00000076685 | PSMB6 | 1,125764 | 1,287579 | 0,717843 | cell signaling & protein processing |
| ENSG00000168268 | PSMB7 | 1,006554 | 0,885053 | 0,62839 | cell signaling & protein processing |
| ENSG00000205309 | PSMC2 | 0,54618 | 0,754569 | 0,539639 | cell signaling & protein processing |
| ENSG00000065320 | PSMC3 | 0,937396 | 0,919445 | 0,428637 | cell signaling & protein processing |
| ENSG00000196358 | PSMC4 | 0,912199 | 0,982313 | 0,745915 | cell signaling & protein processing |
| ENSG00000013374 | PSMC5 | 0,397263 | 0,242087 | -0,23687 | cell signaling & protein processing |
| ENSG00000095906 | PSMD1 | 0,44281 | 0,717374 | 0,525304 | cell signaling & protein processing |
| ENSG00000104805 | PSMD11 | 0,557114 | 0,632246 | 0,252652 | cell signaling & protein processing |
| ENSG00000070081 | PSMD3 | 1,03587 | 0,972807 | 0,752389 | cell signaling & protein processing |
| ENSG00000069275 | PSMD7 | 1,412024 | 1,606718 | 1,649406 | cell signaling & protein processing |
| ENSG00000090273 | PSMD8 | 1,95467 | 1,976999 | 1,544596 | cell signaling & protein processing |
| ENSG00000015676 | PSME1 | 0,935881 | 0,9253 | 1,146034 | cell signaling & protein processing |
| ENSG00000106268 | PSME2 | 1,077808 | 0,306033 | 0,230474 | cell signaling & protein processing |
| ENSG00000168101 | PSME3 | 0,012481 | 0,146398 | 0,011534 | cell signaling & protein processing |
| ENSG00000167005 | PSME4 | 0,778421 | 0,064242 | 1,319399 | cell signaling & protein processing |
| ENSG00000149761 | PSMF1 | 1,470156 | 1,960602 | 2,593608 | cell signaling & protein processing |
| ENSG00000112664 | PSMG1 | 0,744371 | 0,521962 | 0,150893 | cell signaling & protein processing |
| ENSG00000083635 | PSMG2 | 1,336038 | 0,922283 | 1,168609 | cell signaling & protein processing |
| ENSG00000138750 | PTEN | 1,534163 | 1,737146 | 2,702853 | cell signaling & protein processing |
| ENSG00000137804 | PTGS2 | -2,3826 | -2,07263 | 0,15783 | cell signaling & protein processing |
| ENSG00000162231 | PTK2B | -0,54877 | -0,49445 | 1,579712 | cell signaling & protein processing |
| ENSG00000104904 | PTP4A2 | 3,112635 | 2,705953 | 2,861523 | cell signaling & protein processing |
| ENSG00000104904 | PTPMT1 | -0,01725 | -0,08245 | 0,015444 | cell signaling & protein processing |
| ENSG00000104904 | PTPN1 | 0,802706 | 0,346428 | 0,952795 | cell signaling & protein processing |
| ENSG00000104904 | PTPN18 | -0,82331 | -0,69732 | 0,420684 | cell signaling & protein processing |
| ENSG00000180304 | PTPN22 | -2,33856 | -2,47151 | 0,37721 | cell signaling & protein processing |
| ENSG00000139579 | PTPN6 | 0,034339 | -0,08319 | 2,089326 | cell signaling & protein processing |
| ENSG00000115758 | PTPN7 | -0,26989 | -0,12347 | 0,586628 | cell signaling & protein processing |
| ENSG00000136811 | PTPRC | -0,93829 | -1,17525 | 1,408099 | cell signaling & protein processing |
| ENSG00000181781 | PTPRE | -0,77862 | -0,40614 | 2,487508 | cell signaling & protein processing |
| ENSG00000119900 | PTTG1IP | -0,13898 | -0,55777 | 0,747033 | cell signaling & protein processing |
| ENSG00000173391 | PUM1 | 2,054204 | 1,301807 | 1,72243 | cell signaling & protein processing |
| ENSG00000182500 | PXK | -0,5312 | -0,27684 | 1,258485 | cell signaling & protein processing |
| ENSG00000175938 | PXN | -0,39638 | -0,55223 | 0,976398 | cell signaling & protein processing |
| ENSG00000085840 | PYCARD | -0,21847 | 0,024743 | 1,930272 | cell signaling & protein processing |
| ENSG00000229314 | PYCR1 | 0,121496 | 0,303212 | -0,26922 | cell signaling & protein processing |
| ENSG00000091039 | QPCT | -2,51388 | -2,2412 | 0,04009 | cell signaling & protein processing |
| ENSG00000165312 | RAB10 | 0,268153 | 1,2639 | 1,797089 | cell signaling & protein processing |
| ENSG00000068308 | RAB11A | 0,386392 | 0,181603 | 0,57803 | cell signaling & protein processing |
| ENSG00000115507 | RAB11B | 0,952369 | 0,963462 | 1,502615 | cell signaling & protein processing |
| ENSG00000155463 | RAB11FIP1 | -0,44816 | -0,08718 | 1,437169 | cell signaling & protein processing |
| ENSG00000172939 | RAB11FIP2 | 0,366063 | 0,006334 | 0,921189 | cell signaling & protein processing |
| ENSG00000101405 | RAB13 | -0,04059 | 0,040452 | 0,537748 | cell signaling & protein processing |
| ENSG00000108405 | RAB14 | 0,374683 | 0,507624 | 1,354338 | cell signaling & protein processing |
| ENSG00000181631 | RAB18 | -0,22627 | -0,81277 | 0,477542 | cell signaling & protein processing |
| ENSG00000175591 | RAB1A | -0,04733 | 0,214646 | 1,991064 | cell signaling & protein processing |
| ENSG00000182162 | RAB1B | 2,046324 | 2,101635 | 2,718853 | cell signaling & protein processing |
| ENSG00000185624 | RAB21 | -0,72169 | -0,51654 | 0,124517 | cell signaling & protein processing |
| ENSG00000170515 | RAB24 | -0,09452 | 0,138433 | 0,849005 | cell signaling & protein processing |
| ENSG00000070756 | RAB26 | -0,16792 | -0,48908 | 0,171964 | cell signaling & protein processing |
| ENSG00000090621 | RAB27A | -1,75173 | -1,55521 | 0,165172 | cell signaling & protein processing |
| ENSG00000100836 | RAB2B | -0,03047 | -0,58229 | 0,209167 | cell signaling & protein processing |
| ENSG00000175115 | RAB31 | -0,92535 | -0,4187 | 2,980693 | cell signaling & protein processing |
| ENSG00000179364 | RAB33B | -0,62452 | -0,57071 | 0,492975 | cell signaling & protein processing |
| ENSG00000100266 | RAB35 | 0,432079 | 0,608729 | 1,212366 | cell signaling & protein processing |
| ENSG00000117115 | RAB3D | -0,63783 | -0,78739 | 0,881465 | cell signaling & protein processing |
| ENSG00000006712 | RAB40C | -0,3397 | -0,13787 | 0,356812 | cell signaling & protein processing |
| ENSG00000168092 | RAB44 | -2,65764 | -2,49986 | -0,82297 | cell signaling & protein processing |
| ENSG00000079462 | RAB5A | 0,692891 | 0,319726 | 1,645281 | cell signaling & protein processing |
| ENSG00000076641 | RAB5B | 0,15447 | 0,261823 | 1,18128 | cell signaling & protein processing |
| ENSG00000128050 | RAB5C | 1,622443 | 1,91985 | 2,709793 | cell signaling & protein processing |
| ENSG00000120727 | RAB6A | -0,36853 | -0,47207 | 0,612415 | cell signaling & protein processing |
| ENSG00000149269 | RAB7A | 1,878233 | 2,201354 | 2,797863 | cell signaling & protein processing |
| ENSG00000130669 | RAB8B | -0,80064 | -0,54468 | 0,874492 | cell signaling & protein processing |
| ENSG00000120137 | RAC2 | 1,738668 | 1,730232 | 3,622503 | cell signaling & protein processing |
| ENSG00000073150 | RAC3 | 0,153894 | -0,0864 | 0,313046 | cell signaling & protein processing |
| ENSG00000090060 | RAD21 | 0,668707 | 0,413374 | 1,062095 | cell signaling & protein processing |
| ENSG00000116288 | RAF1 | 1,003384 | 0,785839 | 1,551141 | cell signaling & protein processing |
| ENSG00000178685 | RAMP1 | 0,431256 | 0,252724 | 0,712037 | cell signaling & protein processing |
| ENSG00000059378 | RAN | 1,30845 | 1,361538 | 1,097822 | cell signaling & protein processing |
| ENSG00000137817 | RANBP3 | 0,093504 | -0,14558 | 0,154425 | cell signaling & protein processing |
| ENSG00000151883 | RANBP9 | 1,532934 | 1,265797 | 2,507476 | cell signaling & protein processing |
| ENSG00000138964 | RAP1A | -1,53732 | -0,61405 | 0,003217 | cell signaling & protein processing |
| ENSG00000163346 | RAPGEF1 | -0,11402 | -0,18127 | 1,559926 | cell signaling & protein processing |
| ENSG00000169564 | RAPGEF2 | -0,35895 | 0,36778 | 2,631776 | cell signaling & protein processing |
| ENSG00000114054 | RARA | -0,5493 | -0,73365 | 0,167543 | cell signaling & protein processing |
| ENSG00000165494 | RASA3 | -0,5427 | -0,50997 | 0,092006 | cell signaling & protein processing |
| ENSG00000078674 | RASSF1 | 0,592479 | 0,36392 | 0,691969 | cell signaling & protein processing |
| ENSG00000168300 | RASSF5 | 0,148928 | 0,21367 | 1,669929 | cell signaling & protein processing |
| ENSG00000160299 | RB1 | -0,06454 | 0,664042 | 0,555391 | cell signaling & protein processing |
| ENSG00000100731 | RB1CC1 | 0,213485 | -1,0434 | 1,188518 | cell signaling & protein processing |
| ENSG00000102225 | RBCK1 | 0,703468 | 0,710596 | 1,146083 | cell signaling & protein processing |
| ENSG00000131828 | RBM22 | 0,084851 | -0,01885 | 0,964713 | cell signaling & protein processing |
| ENSG00000077684 | RFT1 | -0,76362 | -0,97792 | 0,011983 | cell signaling & protein processing |
| ENSG00000156873 | RHBDD1 | -0,25936 | 0,704789 | 1,43237 | cell signaling & protein processing |
| ENSG00000173868 | RHBDL1 | -0,41053 | -0,49566 | 0,029502 | cell signaling & protein processing |
| ENSG00000143393 | RHOB | 0,29789 | -0,05702 | 0,351152 | cell signaling & protein processing |
| ENSG00000007541 | RHOQ | 1,928414 | 1,703091 | 2,414778 | cell signaling & protein processing |
| ENSG00000087111 | RHOT1 | -1,22694 | -0,72763 | 0,012795 | cell signaling & protein processing |
| ENSG00000124155 | RHOT2 | 0,462678 | 0,89399 | 1,151633 | cell signaling & protein processing |
| ENSG00000078142 | RICTOR | 0,52517 | -0,43881 | 0,73838 | cell signaling & protein processing |
| ENSG00000105851 | RILP | 0,506483 | 0,584801 | 1,03596 | cell signaling & protein processing |
| ENSG00000145675 | RIN3 | 0,998873 | 1,055185 | 1,534583 | cell signaling & protein processing |
| ENSG00000196455 | RIOK3 | 0,643571 | 0,311775 | 2,679473 | cell signaling & protein processing |
| ENSG00000141506 | RIPK2 | 0,679384 | 0,450826 | 0,974048 | cell signaling & protein processing |
| ENSG00000198355 | RNASE2 | 0,24297 | 1,652884 | 4,828243 | cell signaling & protein processing |
| ENSG00000008710 | RNF115 | -0,35185 | -0,2588 | 0,414986 | cell signaling & protein processing |
| ENSG00000151176 | RNF167 | -0,15197 | -0,02033 | 0,681589 | cell signaling & protein processing |
| ENSG00000149527 | RNF187 | 1,550253 | 1,718687 | 1,902614 | cell signaling & protein processing |
| ENSG00000154822 | RNF19A | 0,732171 | 0,482481 | 2,343768 | cell signaling & protein processing |
| ENSG00000178209 | RNF220 | 0,443516 | 0,325922 | 0,43809 | cell signaling & protein processing |
| ENSG00000115762 | RNF31 | -0,29975 | -0,23631 | 0,236654 | cell signaling & protein processing |
| ENSG00000104886 | RNF4 | -0,06434 | -0,07566 | 0,244673 | cell signaling & protein processing |
| ENSG00000178385 | RNF5 | -0,43777 | -0,274 | -0,14255 | cell signaling & protein processing |
| ENSG00000166851 | RNPEP | 0,038595 | 0,075208 | 0,827642 | cell signaling & protein processing |
| ENSG00000173846 | RNPEPL1 | 0,639699 | 0,933886 | 1,42913 | cell signaling & protein processing |
| ENSG00000106397 | ROCK1 | -0,40211 | -0,50675 | 1,130564 | cell signaling & protein processing |
| ENSG00000136040 | ROMO1 | 0,138279 | 0,250951 | 0,580416 | cell signaling & protein processing |
| ENSG00000108179 | RP2 | -1,03949 | -1,07455 | 0,986317 | cell signaling & protein processing |
| ENSG00000184203 | RPAIN | 0,725385 | 0,650659 | 0,717317 | cell signaling & protein processing |
| ENSG00000115685 | RPL10 | 1,487297 | 1,970002 | 1,490544 | cell signaling & protein processing |
| ENSG00000117751 | RPL10A | 2,461475 | 2,492308 | 1,332826 | cell signaling & protein processing |
| ENSG00000113575 | RPL11 | 3,105635 | 2,891413 | 2,32817 | cell signaling & protein processing |
| ENSG00000105568 | RPL12 | 0,561664 | 0,169866 | 0,682661 | cell signaling & protein processing |
| ENSG00000066027 | RPL14 | 0,96644 | 0,986797 | 0,450059 | cell signaling & protein processing |
| ENSG00000068971 | RPL15 | 1,718632 | 1,685297 | 1,366837 | cell signaling & protein processing |
| ENSG00000078304 | RPL17 | -4,64583 | -4,56012 | -4,09235 | cell signaling & protein processing |
| ENSG00000112640 | RPL18 | 5,756405 | 5,540078 | 5,479143 | cell signaling & protein processing |
| ENSG00000154001 | RPL19 | 5,518985 | 5,240628 | 5,048943 | cell signaling & protein processing |
| ENSG00000138814 | RPL24 | -4,47807 | -4,54231 | -4,09242 | cell signaling & protein processing |
| ENSG00000221823 | RPL26 | 2,702075 | 2,119612 | 1,048884 | cell signaling & protein processing |
| ENSG00000149923 | RPL27 | 1,697551 | 1,21335 | 1,251479 | cell signaling & protein processing |
| ENSG00000163605 | RPL28 | -1,13406 | -1,97519 | -0,92747 | cell signaling & protein processing |
| ENSG00000119414 | RPL29 | 1,144395 | 1,107067 | 0,67892 | cell signaling & protein processing |
| ENSG00000100418 | RPL3 | 4,625325 | 4,309538 | 3,579053 | cell signaling & protein processing |
| ENSG00000148840 | RPL30 | 3,722925 | 3,794073 | 3,925593 | cell signaling & protein processing |
| ENSG00000131238 | RPL34 | 4,149665 | 3,731288 | 3,019623 | cell signaling & protein processing |
| ENSG00000122490 | RPL35 | 4,726805 | 4,582188 | 4,211033 | cell signaling & protein processing |
| ENSG00000040487 | RPL35A | 3,891405 | 3,419028 | 3,368583 | cell signaling & protein processing |
| ENSG00000133246 | RPL36 | 2,630695 | 2,369633 | 2,142328 | cell signaling & protein processing |
| ENSG00000198901 | RPL4 | 3,337385 | 3,551148 | 2,767173 | cell signaling & protein processing |
| ENSG00000143294 | RPL8 | 4,548485 | 4,382173 | 4,077463 | cell signaling & protein processing |
| ENSG00000130711 | RPLP0 | 3,595875 | 3,612738 | 3,235583 | cell signaling & protein processing |
| ENSG00000112238 | RPLP1 | 4,403385 | 4,085078 | 4,061953 | cell signaling & protein processing |
| ENSG00000116731 | RPLP2 | 4,015775 | 3,946113 | 3,912783 | cell signaling & protein processing |
| ENSG00000117450 | RPN1 | 1,578672 | 1,199412 | 1,560953 | cell signaling & protein processing |
| ENSG00000167815 | RPN2 | 1,515551 | 1,348408 | 0,949729 | cell signaling & protein processing |
| ENSG00000124126 | RPS12 | 1,62024 | 1,481268 | 0,830617 | cell signaling & protein processing |
| ENSG00000186652 | RPS13 | 1,295097 | 1,284299 | 0,874008 | cell signaling & protein processing |
| ENSG00000156575 | RPS14 | 3,494535 | 3,160178 | 3,034343 | cell signaling & protein processing |
| ENSG00000198056 | RPS15 | 3,214655 | 3,030423 | 2,597605 | cell signaling & protein processing |
| ENSG00000111725 | RPS16 | 2,491935 | 2,571248 | 2,16348 | cell signaling & protein processing |
| ENSG00000072062 | RPS19 | 3,741445 | 3,531678 | 3,290773 | cell signaling & protein processing |
| ENSG00000188191 | RPS20 | 4,407815 | 4,049693 | 3,948773 | cell signaling & protein processing |
| ENSG00000114302 | RPS23 | 1,199003 | 0,963922 | 0,696009 | cell signaling & protein processing |
| ENSG00000005249 | RPS24 | 3,914635 | 3,290918 | 2,761503 | cell signaling & protein processing |
| ENSG00000166501 | RPS25 | 4,701605 | 4,280983 | 4,036583 | cell signaling & protein processing |
| ENSG00000071677 | RPS3 | 4,827735 | 4,821038 | 4,425813 | cell signaling & protein processing |
| ENSG00000100462 | RPS4X | 3,631145 | 3,960428 | 2,431492 | cell signaling & protein processing |
| ENSG00000171867 | RPS5 | 3,835105 | 3,580203 | 2,636483 | cell signaling & protein processing |
| ENSG00000163421 | RPS6 | 4,170835 | 3,853483 | 3,175263 | cell signaling & protein processing |
| ENSG00000110107 | RPS6KA3 | -0,42137 | -0,13962 | 0,18504 | cell signaling & protein processing |
| ENSG00000117360 | RPS6KB1 | -0,00109 | -0,25632 | 0,017769 | cell signaling & protein processing |
| ENSG00000105618 | RPS6KB2 | 0,290322 | 0,580262 | 0,611862 | cell signaling & protein processing |
| ENSG00000134748 | RPS9 | 3,118245 | 3,159933 | 3,236063 | cell signaling & protein processing |
| ENSG00000112739 | RRAGA | -0,08599 | -0,3131 | 0,089701 | cell signaling & protein processing |
| ENSG00000101161 | RRAS2 | 1,170101 | 1,064988 | 1,532078 | cell signaling & protein processing |
| ENSG00000174231 | RSRC1 | -0,09908 | -0,39206 | 0,163222 | cell signaling & protein processing |
| ENSG00000174231 | RUNX3 | 0,339367 | 0,116317 | 1,181805 | cell signaling & protein processing |
| ENSG00000174231 | RUVBL2 | 0,826664 | 0,68257 | -0,29899 | cell signaling & protein processing |
| ENSG00000174231 | RXRA | 0,056242 | 0,639451 | 1,75434 | cell signaling & protein processing |
| ENSG00000174231 | RYK | 0,608825 | 0,251501 | -0,1404 | cell signaling & protein processing |
| ENSG00000174231 | S100A8 | 3,906835 | 4,004153 | 5,793893 | cell signaling & protein processing |
| ENSG00000174231 | S100A9 | 2,286125 | 2,513213 | 5,100173 | cell signaling & protein processing |
| ENSG00000174231 | S1PR2 | 0,19455 | -0,11085 | 0,495234 | cell signaling & protein processing |
| ENSG00000174231 | SAE1 | 1,192691 | 1,122414 | 1,077253 | cell signaling & protein processing |
| ENSG00000101911 | SAMM50 | 0,912979 | 1,149305 | 0,761184 | cell signaling & protein processing |
| ENSG00000125637 | SBF1 | 0,180391 | 0,382916 | 0,906171 | cell signaling & protein processing |
| ENSG00000129084 | SCAMP2 | 0,761814 | 0,62079 | 1,348437 | cell signaling & protein processing |
| ENSG00000106588 | SCAMP3 | 0,128597 | 0,073164 | 0,102666 | cell signaling & protein processing |
| ENSG00000008018 | SCARF1 | -0,07334 | -0,36129 | 0,40077 | cell signaling & protein processing |
| ENSG00000108294 | SCO2 | -0,11115 | -0,30893 | 0,31764 | cell signaling & protein processing |
| ENSG00000159377 | SCPEP1 | 0,377922 | 0,877228 | 1,092421 | cell signaling & protein processing |
| ENSG00000161057 | SDCBP | 0,817428 | 1,082678 | 2,12127 | cell signaling & protein processing |
| ENSG00000013275 | SDHA | 2,401545 | 2,374158 | 1,647689 | cell signaling & protein processing |
| ENSG00000173692 | SDHB | 0,495523 | 0,496895 | 0,378836 | cell signaling & protein processing |
| ENSG00000108671 | SEC11A | 0,203948 | 0,13341 | 0,676639 | cell signaling & protein processing |
| ENSG00000108344 | SEC13 | 0,273425 | 0,154361 | 0,247931 | cell signaling & protein processing |
| ENSG00000092010 | SEC16A | -0,43832 | -0,06644 | 0,563094 | cell signaling & protein processing |
| ENSG00000100911 | SEC22B | -0,25222 | -0,43369 | 0,179651 | cell signaling & protein processing |
| ENSG00000131467 | SEC22C | 0,057529 | 0,131282 | 0,440609 | cell signaling & protein processing |
| ENSG00000068878 | SEC24A | -0,84742 | -0,6429 | 0,03622 | cell signaling & protein processing |
| ENSG00000125818 | SEC24B | 0,351039 | 0,263025 | 0,662986 | cell signaling & protein processing |
| ENSG00000183527 | SEC24C | -0,14641 | -0,54315 | 0,101161 | cell signaling & protein processing |
| ENSG00000128789 | SEC31A | 1,108464 | 0,790327 | 0,98381 | cell signaling & protein processing |
| ENSG00000157778 | SEC61A1 | 0,989488 | 0,96979 | 1,115805 | cell signaling & protein processing |
| ENSG00000204538 | SEC61B | 1,918428 | 1,750005 | 1,849903 | cell signaling & protein processing |
| ENSG00000140368 | SEC63 | 1,238128 | 0,980499 | 1,732901 | cell signaling & protein processing |
| ENSG00000188647 | SEH1L | 0,277839 | -0,14677 | -0,98123 | cell signaling & protein processing |
| ENSG00000011304 | SELENBP1 | 1,135186 | 1,403837 | 3,792613 | cell signaling & protein processing |
| ENSG00000148334 | SENP1 | 0,179096 | 0,053053 | 0,121331 | cell signaling & protein processing |
| ENSG00000073756 | SENP3 | 0,078106 | -0,05167 | 0,085721 | cell signaling & protein processing |
| ENSG00000142538 | SENP5 | 0,389629 | -0,16857 | 0,661249 | cell signaling & protein processing |
| ENSG00000120899 | SEPHS1 | -0,0786 | -0,13156 | 0,273971 | cell signaling & protein processing |
| ENSG00000081237 | SERP1 | 0,81527 | 0,806091 | 1,400555 | cell signaling & protein processing |
| ENSG00000103490 | SF3B3 | 1,493885 | 1,834903 | 1,633354 | cell signaling & protein processing |
| ENSG00000174903 | SFT2D2 | -0,58511 | -0,38659 | 0,166961 | cell signaling & protein processing |
| ENSG00000169228 | SGMS1 | -1,05828 | -0,51946 | 0,903358 | cell signaling & protein processing |
| ENSG00000167964 | SGPP1 | 0,695937 | 0,332094 | 0,333513 | cell signaling & protein processing |
| ENSG00000168461 | SH2B3 | 0,138474 | 0,251453 | 1,007139 | cell signaling & protein processing |
| ENSG00000197562 | SH3GL1 | -0,08561 | 0,300856 | 1,259313 | cell signaling & protein processing |
| ENSG00000111540 | SH3KBP1 | -0,23431 | -0,17776 | 1,110092 | cell signaling & protein processing |
| ENSG00000166128 | SHC1 | 0,745853 | 0,714629 | 1,123673 | cell signaling & protein processing |
| ENSG00000105404 | SHFM1 | 0,991752 | 0,623368 | 0,236077 | cell signaling & protein processing |
| ENSG00000119318 | SIAH2 | 1,183588 | 1,223272 | 1,852194 | cell signaling & protein processing |
| ENSG00000160271 | SIK1 | -0,31737 | -0,43788 | 0,322453 | cell signaling & protein processing |
| ENSG00000125970 | SIK3 | 0,195374 | -0,04046 | 1,091231 | cell signaling & protein processing |
| ENSG00000132329 | SIN3A | 0,353628 | 0,43445 | 0,798914 | cell signaling & protein processing |
| ENSG00000132341 | SIPA1 | 0,012049 | 0,245423 | 1,750781 | cell signaling & protein processing |
| ENSG00000100401 | SIRPB1 | -1,83335 | -1,08115 | 1,738767 | cell signaling & protein processing |
| ENSG00000116473 | SIRT1 | -0,41654 | -0,40145 | 0,127446 | cell signaling & protein processing |
| ENSG00000132359 | SIRT2 | -0,16778 | 0,04837 | 0,494568 | cell signaling & protein processing |
| ENSG00000181467 | SIRT3 | 1,109982 | 0,92083 | 0,938108 | cell signaling & protein processing |
| ENSG00000123728 | SIRT6 | 0,066547 | -0,23009 | 0,340478 | cell signaling & protein processing |
| ENSG00000107263 | SIRT7 | -0,36497 | -0,34442 | 0,495263 | cell signaling & protein processing |
| ENSG00000158987 | SIX2 | 0,58316 | 0,365995 | 0,687199 | cell signaling & protein processing |
| ENSG00000105122 | SKAP2 | -1,07187 | -0,61923 | 0,660311 | cell signaling & protein processing |
| ENSG00000101265 | SKP2 | -0,16338 | 0,277476 | -0,30836 | cell signaling & protein processing |
| ENSG00000023287 | SLC11A1 | -1,02402 | -0,82734 | 0,740286 | cell signaling & protein processing |
| ENSG00000146587 | SLC11A2 | -0,05335 | 0,704304 | 1,590904 | cell signaling & protein processing |
| ENSG00000080839 | SLC15A4 | -0,80885 | -0,72211 | 1,976258 | cell signaling & protein processing |
| ENSG00000162775 | SLC17A9 | -0,5866 | -0,62781 | 0,009193 | cell signaling & protein processing |
| ENSG00000102317 | SLC25A1 | 0,482487 | 0,296475 | 0,120318 | cell signaling & protein processing |
| ENSG00000168214 | SLC29A1 | 1,392746 | 1,821041 | 1,345108 | cell signaling & protein processing |
| ENSG00000076043 | SLC40A1 | 1,300528 | 0,951146 | 1,607948 | cell signaling & protein processing |
| ENSG00000100263 | SLC9A3R1 | 0,906412 | 0,74364 | 1,838424 | cell signaling & protein processing |
| ENSG00000067560 | SLK | 0,717091 | 0,577121 | 0,623118 | cell signaling & protein processing |
| ENSG00000067560 | SLMAP | 0,358508 | -0,073 | -0,08318 | cell signaling & protein processing |
| ENSG00000067560 | SMAD3 | 0,18712 | -0,24852 | -0,31358 | cell signaling & protein processing |
| ENSG00000117000 | SMG1 | 0,054318 | 0,306982 | 1,222914 | cell signaling & protein processing |
| ENSG00000104889 | SMPD4 | 1,844659 | 1,95591 | 1,427342 | cell signaling & protein processing |
| ENSG00000219200 | SMURF1 | -0,20822 | -0,63264 | 0,2008 | cell signaling & protein processing |
| ENSG00000026297 | SNAP23 | -0,72782 | -0,37614 | 0,485819 | cell signaling & protein processing |
| ENSG00000022840 | SNAP29 | 0,142937 | 0,122302 | 0,418104 | cell signaling & protein processing |
| ENSG00000121848 | SNCA | 1,016413 | 1,509554 | 3,699533 | cell signaling & protein processing |
| ENSG00000113269 | SNF8 | 0,554586 | 0,284628 | 0,466159 | cell signaling & protein processing |
| ENSG00000205937 | SNRK | 0,630862 | 0,512681 | 1,835804 | cell signaling & protein processing |
| ENSG00000237872 | SNX17 | 0,80289 | 0,342225 | 0,947993 | cell signaling & protein processing |
| ENSG00000169253 | SNX18 | -0,34196 | -0,59642 | 0,764002 | cell signaling & protein processing |
| ENSG00000242299 | SNX19 | -0,37386 | -0,12243 | 0,492286 | cell signaling & protein processing |
| ENSG00000231167 | SNX2 | -0,1573 | -0,23954 | 0,320901 | cell signaling & protein processing |
| ENSG00000234589 | SNX22 | -0,24596 | 0,03665 | 0,815066 | cell signaling & protein processing |
| ENSG00000230626 | SNX3 | 3,116825 | 3,066073 | 3,752573 | cell signaling & protein processing |
| ENSG00000233406 | SNX5 | 1,646105 | 1,294566 | 0,811509 | cell signaling & protein processing |
| ENSG00000213866 | SNX9 | -0,18861 | 0,160675 | 0,516621 | cell signaling & protein processing |
| ENSG00000197067 | SOD1 | 1,296588 | 1,526291 | 0,918932 | cell signaling & protein processing |
| ENSG00000225178 | SOD2 | -0,26943 | -0,43738 | 0,91811 | cell signaling & protein processing |
| ENSG00000189339 | SOLH | 0,481215 | 0,42391 | 0,591419 | cell signaling & protein processing |
| ENSG00000136149 | SORL1 | -1,49426 | -0,77317 | 1,96007 | cell signaling & protein processing |
| ENSG00000178654 | SORT1 | -2,01824 | -1,41772 | 1,437253 | cell signaling & protein processing |
| ENSG00000229945 | SOX4 | 0,427385 | -0,00458 | 0,633189 | cell signaling & protein processing |
| ENSG00000223803 | SOX9 | 0,785073 | 0,250017 | 0,964454 | cell signaling & protein processing |
| ENSG00000232203 | SPCS1 | 0,840692 | 0,824482 | 0,775611 | cell signaling & protein processing |
| ENSG00000212664 | SPCS3 | 0,540104 | 0,403458 | 0,67417 | cell signaling & protein processing |
| ENSG00000243779 | SPG7 | -0,16166 | -0,33328 | 0,140865 | cell signaling & protein processing |
| ENSG00000235244 | SPN | 1,443186 | 1,370412 | 0,861922 | cell signaling & protein processing |
| ENSG00000234080 | SPSB2 | -0,21319 | -0,19711 | 0,524022 | cell signaling & protein processing |
| ENSG00000139239 | SPSB3 | 0,55076 | 0,546451 | 1,107783 | cell signaling & protein processing |
| ENSG00000139239 | SPTA1 | 1,470084 | 2,162233 | 3,578063 | cell signaling & protein processing |
| ENSG00000177133 | SQSTM1 | 0,830946 | 0,802821 | 1,488165 | cell signaling & protein processing |
| ENSG00000219682 | SREBF1 | 0,623009 | 1,164031 | 0,886621 | cell signaling & protein processing |
| ENSG00000228526 | SRF | 1,40743 | 1,427831 | 1,490989 | cell signaling & protein processing |
| ENSG00000185290 | SRPK1 | 1,187043 | 0,926124 | 1,748019 | cell signaling & protein processing |
| ENSG00000225573 | SRPK3 | -0,09758 | -0,29909 | 0,146891 | cell signaling & protein processing |
| ENSG00000198755 | SSBP3 | 1,917643 | 1,849203 | 1,903066 | cell signaling & protein processing |
| ENSG00000142676 | SSH2 | -0,86563 | -0,74392 | 0,07609 | cell signaling & protein processing |
| ENSG00000215472 | ST3GAL1 | -0,34021 | 0,374166 | 1,79817 | cell signaling & protein processing |
| ENSG00000063177 | ST3GAL2 | 0,604547 | 0,940554 | 1,275298 | cell signaling & protein processing |
| ENSG00000063177 | ST6GAL1 | -0,61143 | -0,18817 | 0,116545 | cell signaling & protein processing |
| ENSG00000063177 | STAM | 2,098835 | 1,539117 | 3,011633 | cell signaling & protein processing |
| ENSG00000108298 | STAT3 | 1,235879 | 1,052138 | 2,423877 | cell signaling & protein processing |
| ENSG00000108298 | STAT5A | 1,349442 | 0,847192 | -0,11737 | cell signaling & protein processing |
| ENSG00000114391 | STAT5B | 0,805869 | 0,878153 | 0,921595 | cell signaling & protein processing |
| ENSG00000131469 | STEAP3 | 0,33235 | 0,693943 | 0,49398 | cell signaling & protein processing |
| ENSG00000108107 | STK10 | -0,20316 | 0,009847 | 1,304592 | cell signaling & protein processing |
| ENSG00000108107 | STK11 | 0,439918 | 0,748099 | 1,675565 | cell signaling & protein processing |
| ENSG00000108107 | STK17B | 1,66504 | 1,490502 | 3,250983 | cell signaling & protein processing |
| ENSG00000108107 | STK24 | 0,893669 | 1,155937 | 1,700665 | cell signaling & protein processing |
| ENSG00000108107 | STK25 | 1,86226 | 1,700689 | 0,688308 | cell signaling & protein processing |
| ENSG00000162244 | STK3 | 0,777724 | 0,33534 | 0,886687 | cell signaling & protein processing |
| ENSG00000156482 | STK38 | -0,61713 | 0,097018 | 0,909335 | cell signaling & protein processing |
| ENSG00000156482 | STK4 | 0,080806 | -0,04391 | 1,745589 | cell signaling & protein processing |
| ENSG00000156482 | STMN1 | 1,752942 | 1,187602 | 0,566115 | cell signaling & protein processing |
| ENSG00000136942 | STT3A | 0,418936 | 0,452 | 0,294056 | cell signaling & protein processing |
| ENSG00000130255 | STUB1 | 0,746764 | 0,50745 | 0,289763 | cell signaling & protein processing |
| ENSG00000174444 | STX3 | -1,8682 | -1,31349 | 1,062095 | cell signaling & protein processing |
| ENSG00000161016 | STX4 | 0,089021 | -0,13011 | 0,498264 | cell signaling & protein processing |
| ENSG00000177600 | STXBP5 | -1,54324 | -1,34268 | 0,77999 | cell signaling & protein processing |
| ENSG00000101278 | SUPT16H | 0,545125 | 0,343605 | -0,70652 | cell signaling & protein processing |
| ENSG00000138326 | SYK | 0,24391 | -0,05709 | 1,227964 | cell signaling & protein processing |
| ENSG00000118181 | SYNGR1 | 2,254962 | 2,268796 | 1,066063 | cell signaling & protein processing |
| ENSG00000235508 | SYTL1 | -0,59544 | -0,73948 | 0,097292 | cell signaling & protein processing |
| ENSG00000149273 | TACC3 | -0,00652 | 0,447976 | 0,647874 | cell signaling & protein processing |
| ENSG00000178429 | TAF1 | 0,290288 | -0,03501 | 0,338649 | cell signaling & protein processing |
| ENSG00000170889 | TAOK2 | -0,09706 | -0,49007 | 0,010464 | cell signaling & protein processing |
| ENSG00000156990 | TAOK3 | -0,37664 | -0,27204 | 0,109349 | cell signaling & protein processing |
| ENSG00000186907 | TBCA | 1,117474 | 0,845076 | 1,048718 | cell signaling & protein processing |
| ENSG00000140660 | TBCB | -0,03822 | 0,197235 | 0,695005 | cell signaling & protein processing |
| ENSG00000159216 | TBCC | -0,04395 | 0,011241 | 0,271462 | cell signaling & protein processing |
| ENSG00000020633 | TBCD | 0,520982 | 0,696762 | 0,348373 | cell signaling & protein processing |
| ENSG00000183207 | TBK1 | -0,51179 | -0,70316 | 0,065558 | cell signaling & protein processing |
| ENSG00000186350 | TBL1XR1 | -0,72238 | -0,62353 | 0,391824 | cell signaling & protein processing |
| ENSG00000163785 | TBRG1 | 0,368097 | 0,319043 | 0,432619 | cell signaling & protein processing |
| ENSG00000196154 | TCEB2 | -4,42111 | -4,31751 | -3,90297 | cell signaling & protein processing |
| ENSG00000180739 | TCF3 | 1,14689 | 1,249409 | 1,196327 | cell signaling & protein processing |
| ENSG00000179134 | TCP1 | 2,369885 | 2,351488 | 2,038079 | cell signaling & protein processing |
| ENSG00000136715 | TECR | 2,052165 | 2,278183 | 2,001585 | cell signaling & protein processing |
| ENSG00000100239 | TERF2 | 0,007937 | 0,05059 | 0,225801 | cell signaling & protein processing |
| ENSG00000175467 | TFAP4 | 0,00599 | -0,44069 | -0,60174 | cell signaling & protein processing |
| ENSG00000175467 | TFR2 | 2,08106 | 1,574427 | 1,317695 | cell signaling & protein processing |
| ENSG00000175467 | TFRC | 2,842965 | 3,612313 | 4,130343 | cell signaling & protein processing |
| ENSG00000175467 | TGFB1 | -0,06435 | 0,069337 | 1,570909 | cell signaling & protein processing |
| ENSG00000175467 | TGFBR2 | -0,74445 | -0,60434 | 1,100291 | cell signaling & protein processing |
| ENSG00000175467 | TGIF1 | -0,996 | -1,28992 | -1,0016 | cell signaling & protein processing |
| ENSG00000075856 | THBS1 | -2,0025 | -1,02608 | 0,866713 | cell signaling & protein processing |
| ENSG00000122122 | THOP1 | 0,472024 | 0,213892 | 0,123161 | cell signaling & protein processing |
| ENSG00000140497 | TIMM50 | 1,191266 | 1,189644 | 0,662052 | cell signaling & protein processing |
| ENSG00000212530 | TIPARP | -0,5668 | -1,27299 | 0,20432 | cell signaling & protein processing |
| ENSG00000099194 | TLK1 | 0,30825 | 0,318421 | 0,352889 | cell signaling & protein processing |
| ENSG00000163156 | TLN1 | 1,597636 | 2,009866 | 2,104676 | cell signaling & protein processing |
| ENSG00000138802 | TMED4 | 0,880497 | 0,904436 | 2,20986 | cell signaling & protein processing |
| ENSG00000176986 | TMED5 | -1,19744 | -1,03832 | 0,586278 | cell signaling & protein processing |
| ENSG00000138674 | TMED9 | -0,03437 | -0,2567 | 0,069727 | cell signaling & protein processing |
| ENSG00000183431 | TNFAIP3 | -0,53885 | -0,48525 | 1,760881 | cell signaling & protein processing |
| ENSG00000115524 | TNFRSF12A | 0,411636 | 0,206564 | 0,988535 | cell signaling & protein processing |
| ENSG00000143368 | TNFRSF14 | -0,6107 | -0,26589 | 0,556472 | cell signaling & protein processing |
| ENSG00000116560 | TNFRSF1B | -4,41574 | -4,20394 | -3,90817 | cell signaling & protein processing |
| ENSG00000136450 | TNFRSF25 | 0,707273 | 0,3719 | 0,96923 | cell signaling & protein processing |
| ENSG00000104859 | TNK2 | 0,448254 | 0,35713 | 1,348269 | cell signaling & protein processing |
| ENSG00000161547 | TNKS2 | 0,258511 | -0,35667 | 0,611945 | cell signaling & protein processing |
| ENSG00000139218 | TNPO1 | 0,209317 | 0,064318 | 0,605369 | cell signaling & protein processing |
| ENSG00000116350 | TNPO3 | 0,618901 | 0,261682 | 0,46692 | cell signaling & protein processing |
| ENSG00000111786 | TNS1 | -0,61812 | -0,27577 | 0,791801 | cell signaling & protein processing |
| ENSG00000111786 | TOLLIP | 0,176162 | 0,246348 | 0,790199 | cell signaling & protein processing |
| ENSG00000111786 | TOM1 | -0,09549 | 0,020659 | 0,882859 | cell signaling & protein processing |
| ENSG00000164466 | TOMM40 | 0,517833 | 0,130256 | 0,079621 | cell signaling & protein processing |
| ENSG00000126821 | TOP1 | 1,83039 | 1,847226 | 3,656643 | cell signaling & protein processing |
| ENSG00000148341 | TP53 | 1,540879 | 1,614751 | 0,599299 | cell signaling & protein processing |
| ENSG00000160410 | TPM1 | 0,204145 | -0,05282 | -0,33722 | cell signaling & protein processing |
| ENSG00000182199 | TPM4 | -0,61123 | -0,50855 | 0,138222 | cell signaling & protein processing |
| ENSG00000108061 | TPP1 | 0,139398 | 0,764877 | 1,560782 | cell signaling & protein processing |
| ENSG00000181788 | TPP2 | 0,286871 | -0,06795 | 0,264256 | cell signaling & protein processing |
| ENSG00000149577 | TPR | 0,333972 | 0,100931 | -0,06355 | cell signaling & protein processing |
| ENSG00000105501 | TPSD1 | 0,664497 | 0,217449 | 0,741828 | cell signaling & protein processing |
| ENSG00000105738 | TRAF2 | -0,18352 | -0,10462 | 0,134143 | cell signaling & protein processing |
| ENSG00000198053 | TRAF6 | 0,005267 | -0,52772 | 0,376704 | cell signaling & protein processing |
| ENSG00000096717 | TRAK2 | -0,68714 | 0,447823 | 2,774453 | cell signaling & protein processing |
| ENSG00000068903 | TRAM1 | 0,049538 | -0,16923 | 0,555211 | cell signaling & protein processing |
| ENSG00000142082 | TRAP1 | 1,798663 | 1,812427 | -0,13568 | cell signaling & protein processing |
| ENSG00000157933 | TREM1 | -1,92613 | -1,22495 | 1,721473 | cell signaling & protein processing |
| ENSG00000145604 | TRIB1 | -0,20962 | 0,028627 | 3,110413 | cell signaling & protein processing |
| ENSG00000155926 | TRIB2 | 0,693654 | 0,114379 | -1,04885 | cell signaling & protein processing |
| ENSG00000145248 | TRIM17 | -0,4071 | -0,72388 | 0,15112 | cell signaling & protein processing |
| ENSG00000110911 | TRIM28 | 2,902785 | 2,430658 | 1,880387 | cell signaling & protein processing |
| ENSG00000101194 | TRIP12 | 0,167655 | 0,444409 | 1,114984 | cell signaling & protein processing |
| ENSG00000075415 | TSC1 | -0,86913 | -0,52824 | 0,56489 | cell signaling & protein processing |
| ENSG00000075415 | TSC2 | 0,363093 | 0,372683 | 0,697923 | cell signaling & protein processing |
| ENSG00000144659 | TSPAN4 | 0,611135 | 0,015879 | 0,234545 | cell signaling & protein processing |
| ENSG00000112759 | TSSK3 | -0,83169 | -1,12281 | 0,105886 | cell signaling & protein processing |
| ENSG00000170385 | TTC7A | -0,46499 | -0,26329 | 0,339026 | cell signaling & protein processing |
| ENSG00000136867 | TTLL12 | 1,701981 | 1,517311 | 0,811415 | cell signaling & protein processing |
| ENSG00000176087 | TTLL3 | -1,26699 | -1,18521 | 0,20232 | cell signaling & protein processing |
| ENSG00000121073 | TTLL5 | 0,381807 | 0,38474 | 0,102413 | cell signaling & protein processing |
| ENSG00000137700 | TUBA1A | -0,27724 | -0,35483 | 0,774084 | cell signaling & protein processing |
| ENSG00000111371 | TUBA1B | 2,774675 | 2,923088 | 2,903473 | cell signaling & protein processing |
| ENSG00000157637 | TUBA1C | 1,121784 | 1,605301 | 1,23729 | cell signaling & protein processing |
| ENSG00000134294 | TUBB | 4,167375 | 4,484843 | 4,386993 | cell signaling & protein processing |
| ENSG00000017483 | TUBB2A | 0,969177 | 0,832237 | 0,841476 | cell signaling & protein processing |
| ENSG00000141873 | TUBB2C | 3,176345 | 3,709963 | 3,923293 | cell signaling & protein processing |
| ENSG00000138821 | TUBB6 | 0,760156 | 0,955924 | 1,176196 | cell signaling & protein processing |
| ENSG00000029364 | TUBG1 | 2,308565 | 3,134313 | 2,066789 | cell signaling & protein processing |
| ENSG00000138449 | TUBGCP4 | -0,51009 | 0,123776 | -0,62109 | cell signaling & protein processing |
| ENSG00000211584 | TWF2 | -0,11241 | -0,01573 | 1,237472 | cell signaling & protein processing |
| ENSG00000164889 | TXN | -0,06932 | 0,387357 | -0,01184 | cell signaling & protein processing |
| ENSG00000114923 | TXN2 | 0,265646 | 0,179162 | -0,43704 | cell signaling & protein processing |
| ENSG00000130821 | TXNIP | 3,471265 | 3,051753 | 3,753223 | cell signaling & protein processing |
| ENSG00000090020 | TYK2 | 0,595137 | 0,629861 | 1,11028 | cell signaling & protein processing |
| ENSG00000109062 | TYMP | 0,007595 | 0,017003 | 0,639131 | cell signaling & protein processing |
| ENSG00000176463 | TYROBP | 1,725917 | 1,907858 | 4,353353 | cell signaling & protein processing |
| ENSG00000127616 | UAP1 | -0,37727 | -0,6413 | 0,24399 | cell signaling & protein processing |
| ENSG00000099956 | UBA1 | 1,925578 | 1,475205 | 1,972884 | cell signaling & protein processing |
| ENSG00000173473 | UBA2 | 0,664344 | 0,949232 | 0,765919 | cell signaling & protein processing |
| ENSG00000139613 | UBA52 | 2,730905 | 2,673263 | 2,979033 | cell signaling & protein processing |
| ENSG00000066117 | UBA7 | 0,142883 | -0,35918 | -0,28604 | cell signaling & protein processing |
| ENSG00000198887 | UBB | 4,588115 | 4,657338 | 5,213323 | cell signaling & protein processing |
| ENSG00000196787 | UBC | 5,120515 | 4,831483 | 5,873363 | cell signaling & protein processing |
| ENSG00000177427 | UBE2A | -0,23556 | -0,28984 | 0,566122 | cell signaling & protein processing |
| ENSG00000176994 | UBE2B | -0,97728 | -0,80408 | 0,421321 | cell signaling & protein processing |
| ENSG00000138041 | UBE2D2 | 0,257577 | -0,00353 | 0,426517 | cell signaling & protein processing |
| ENSG00000198952 | UBE2E1 | 0,731349 | 0,319062 | 0,50982 | cell signaling & protein processing |
| ENSG00000119953 | UBE2I | 0,084686 | 0,163065 | 0,436247 | cell signaling & protein processing |
| ENSG00000135587 | UBE2L6 | 0,878624 | 0,972105 | 1,005947 | cell signaling & protein processing |
| ENSG00000136699 | UBE2M | 0,388888 | 0,239452 | 0,406042 | cell signaling & protein processing |
| ENSG00000102172 | UBE2N | 0,416016 | 0,015038 | 0,579026 | cell signaling & protein processing |
| ENSG00000164975 | UBE3B | -0,70156 | -0,39954 | 0,079969 | cell signaling & protein processing |
| ENSG00000145335 | UBE3C | -0,86346 | -0,18053 | 0,032971 | cell signaling & protein processing |
| ENSG00000197157 | UBE4B | 0,241935 | 0,129989 | 0,746908 | cell signaling & protein processing |
| ENSG00000200792 | UBR2 | -1,07334 | -0,4199 | 1,816422 | cell signaling & protein processing |
| ENSG00000207475 | UBR5 | -0,20451 | -0,49092 | 0,340283 | cell signaling & protein processing |
| ENSG00000207098 | UCP2 | 1,482449 | 2,498933 | 3,184333 | cell signaling & protein processing |
| ENSG00000207174 | UHMK1 | -0,27106 | 0,030824 | 0,330397 | cell signaling & protein processing |
| ENSG00000202252 | UHRF2 | 0,710276 | 0,485159 | 0,579846 | cell signaling & protein processing |
| ENSG00000206874 | ULK1 | 0,601305 | 0,897391 | 2,404964 | cell signaling & protein processing |
| ENSG00000201199 | UQCRC2 | 1,232927 | 1,119762 | 0,689704 | cell signaling & protein processing |
| ENSG00000199675 | UQCRQ | 0,048608 | -0,17303 | -0,41147 | cell signaling & protein processing |
| ENSG00000144028 | USP10 | -0,10712 | 0,111895 | 0,094565 | cell signaling & protein processing |
| ENSG00000144028 | USP11 | -0,21627 | 0,044989 | -0,11665 | cell signaling & protein processing |
| ENSG00000144028 | USP12 | 0,357835 | 0,450334 | 1,107732 | cell signaling & protein processing |
| ENSG00000144028 | USP14 | 0,783142 | 0,729581 | 0,847167 | cell signaling & protein processing |
| ENSG00000144028 | USP15 | 1,120084 | 1,52727 | 2,797373 | cell signaling & protein processing |
| ENSG00000144028 | USP19 | 0,335381 | 0,335237 | 1,120963 | cell signaling & protein processing |
| ENSG00000144028 | USP20 | -1,17657 | -1,04558 | 0,599917 | cell signaling & protein processing |
| ENSG00000144028 | USP21 | 0,40135 | 0,406746 | 0,070516 | cell signaling & protein processing |
| ENSG00000144028 | USP22 | -0,27465 | -1,27138 | -1,22546 | cell signaling & protein processing |
| ENSG00000144028 | USP24 | 1,193977 | 1,173587 | 1,770981 | cell signaling & protein processing |
| ENSG00000144028 | USP25 | 0,106583 | 0,129983 | 0,349036 | cell signaling & protein processing |
| ENSG00000144028 | USP3 | 0,654207 | 0,389072 | 1,47259 | cell signaling & protein processing |
| ENSG00000161981 | USP30 | 0,15976 | -0,39472 | 0,055891 | cell signaling & protein processing |
| ENSG00000124380 | USP32 | -0,17926 | 0,81542 | 2,274076 | cell signaling & protein processing |
| ENSG00000104852 | USP34 | -0,30331 | -0,04752 | 0,423012 | cell signaling & protein processing |
| ENSG00000077312 | USP36 | 0,006856 | -0,18005 | 0,13843 | cell signaling & protein processing |
| ENSG00000125835 | USP38 | 0,081191 | -0,30865 | 0,660705 | cell signaling & protein processing |
| ENSG00000124562 | USP4 | -0,11311 | 0,079759 | 1,552489 | cell signaling & protein processing |
| ENSG00000125743 | USP42 | 0,318738 | -0,25521 | 0,879 | cell signaling & protein processing |
| ENSG00000172164 | USP5 | 0,085118 | -0,06794 | -0,33213 | cell signaling & protein processing |
| ENSG00000100603 | USP7 | 0,767803 | 1,069808 | 2,939913 | cell signaling & protein processing |
| ENSG00000115234 | USP9X | 0,497592 | 0,343836 | 1,70628 | cell signaling & protein processing |
| ENSG00000004777 | UXT | 0,391326 | 0,678029 | 0,483544 | cell signaling & protein processing |
| ENSG00000112335 | VAMP2 | -0,1674 | 0,053001 | 1,039189 | cell signaling & protein processing |
| ENSG00000112335 | VAMP3 | -0,03757 | -0,07263 | 1,399182 | cell signaling & protein processing |
| ENSG00000112335 | VAMP7 | 0,670127 | 0,504309 | 1,643373 | cell signaling & protein processing |
| ENSG00000112335 | VAMP8 | 1,120922 | 1,065193 | 1,50101 | cell signaling & protein processing |
| ENSG00000112335 | VAPA | 0,482728 | 0,064336 | 0,915272 | cell signaling & protein processing |
| ENSG00000130340 | VAV1 | -1,14382 | -0,91728 | 0,384009 | cell signaling & protein processing |
| ENSG00000103326 | VCL | -1,13894 | -1,76815 | 0,267802 | cell signaling & protein processing |
| ENSG00000140263 | VCPIP1 | 0,296116 | -0,07389 | 0,772308 | cell signaling & protein processing |
| ENSG00000115904 | VEGFA | -0,97489 | -0,62991 | 0,206981 | cell signaling & protein processing |
| ENSG00000134595 | VIM | 1,041998 | 1,617704 | 4,327433 | cell signaling & protein processing |
| ENSG00000124766 | VKORC1 | 0,499612 | 0,671985 | 0,757602 | cell signaling & protein processing |
| ENSG00000110693 | VNN1 | -2,57556 | -2,0549 | 0,394496 | cell signaling & protein processing |
| ENSG00000005513 | VNN2 | -2,91182 | -2,07674 | 0,288501 | cell signaling & protein processing |
| ENSG00000172845 | VPS13B | -0,76159 | -0,53476 | 0,722523 | cell signaling & protein processing |
| ENSG00000105866 | VPS24 | -0,25663 | -0,0259 | 0,39173 | cell signaling & protein processing |
| ENSG00000204335 | VPS25 | 0,086286 | 0,190888 | -0,04662 | cell signaling & protein processing |
| ENSG00000170374 | VPS26B | -0,74723 | -0,15806 | 0,520702 | cell signaling & protein processing |
| ENSG00000076382 | VPS28 | 0,921176 | 1,158027 | 1,612457 | cell signaling & protein processing |
| ENSG00000091640 | VPS35 | -0,16867 | 0,06128 | -0,30643 | cell signaling & protein processing |
| ENSG00000091640 | VPS37B | 1,262053 | 0,867989 | 0,429022 | cell signaling & protein processing |
| ENSG00000091640 | VPS37C | -0,23238 | -0,10786 | 0,145258 | cell signaling & protein processing |
| ENSG00000091640 | VPS37D | 0,201818 | 0,043588 | 0,557728 | cell signaling & protein processing |
| ENSG00000091640 | VPS39 | 0,295091 | 0,083784 | 0,570562 | cell signaling & protein processing |
| ENSG00000114902 | VPS4A | 0,404891 | 0,37212 | 0,211837 | cell signaling & protein processing |
| ENSG00000129128 | VPS4B | -0,77544 | -0,64368 | 0,069826 | cell signaling & protein processing |
| ENSG00000065526 | VPS52 | -0,14542 | 0,141521 | 0,231264 | cell signaling & protein processing |
| ENSG00000090487 | VPS53 | 0,076445 | 0,210073 | 0,672542 | cell signaling & protein processing |
| ENSG00000066336 | VRK1 | -0,4259 | 0,288776 | 0,66505 | cell signaling & protein processing |
| ENSG00000163554 | WAPAL | -0,15014 | 0,169955 | 0,573048 | cell signaling & protein processing |
| ENSG00000115306 | WAS | -0,40565 | -0,29186 | 1,589188 | cell signaling & protein processing |
| ENSG00000100596 | WASF2 | 0,331668 | 0,538407 | 1,534813 | cell signaling & protein processing |
| ENSG00000137767 | WASL | -0,3501 | -0,52045 | 0,061974 | cell signaling & protein processing |
| ENSG00000223097 | WDR48 | 0,02109 | -0,55658 | 0,014609 | cell signaling & protein processing |
| ENSG00000184343 | WIPF1 | 0,403937 | 0,614627 | 1,873069 | cell signaling & protein processing |
| ENSG00000133226 | WNK1 | 0,94233 | 1,485558 | 3,653813 | cell signaling & protein processing |
| ENSG00000087087 | WNT10B | -0,28551 | -0,4559 | 0,059043 | cell signaling & protein processing |
| ENSG00000145687 | WSB1 | -0,31291 | 0,483704 | 1,677877 | cell signaling & protein processing |
| ENSG00000157216 | WSB2 | -0,39089 | 0,374907 | 0,26766 | cell signaling & protein processing |
| ENSG00000141298 | WWP2 | -0,71247 | -0,50575 | 0,00973 | cell signaling & protein processing |
| ENSG00000163479 | XPNPEP1 | -0,01046 | -0,10616 | 0,026056 | cell signaling & protein processing |
| ENSG00000180879 | XPO1 | 0,25478 | 0,333269 | -0,03788 | cell signaling & protein processing |
| ENSG00000149136 | XPO6 | 1,492558 | 1,739263 | 2,011932 | cell signaling & protein processing |
| ENSG00000173465 | XPO7 | -0,19121 | 0,241507 | 3,870203 | cell signaling & protein processing |
| ENSG00000084090 | YIF1A | 0,045352 | 0,035743 | 0,145534 | cell signaling & protein processing |
| ENSG00000084090 | YKT6 | -0,46334 | -0,17158 | 0,415931 | cell signaling & protein processing |
| ENSG00000084090 | YME1L1 | 1,673317 | 1,070129 | 1,807441 | cell signaling & protein processing |
| ENSG00000084090 | YOD1 | 1,119351 | 0,836415 | 1,783837 | cell signaling & protein processing |
| ENSG00000167323 | YWHAB | 1,294485 | 1,656333 | 2,169249 | cell signaling & protein processing |
| ENSG00000168439 | YWHAE | 2,514555 | 2,513953 | 1,73775 | cell signaling & protein processing |
| ENSG00000072786 | YWHAG | 1,456389 | 1,315218 | 0,667055 | cell signaling & protein processing |
| ENSG00000081320 | YWHAQ | 2,678595 | 2,621478 | 2,498525 | cell signaling & protein processing |
| ENSG00000169689 | ZBTB33 | 0,2928 | 0,072158 | 0,580291 | cell signaling & protein processing |
| ENSG00000213246 | ZDHHC18 | 0,171245 | 0,357895 | 1,605775 | cell signaling & protein processing |
| ENSG00000119760 | ZDHHC3 | -0,04225 | -0,09345 | 0,656919 | cell signaling & protein processing |
| ENSG00000162298 | ZFYVE27 | -0,76611 | -0,70221 | 0,031319 | cell signaling & protein processing |
| ENSG00000196507 | ZRANB1 | -0,18343 | 0,131572 | 1,30673 | cell signaling & protein processing |
| ENSG00000201492 | AATF | 0,649128 | 0,261702 | 0,564653 | structural organization |
| ENSG00000199806 | ABCF1 | -4,31484 | -4,4672 | -4,21775 | structural organization |
| ENSG00000201041 | ABI1 | -0,33454 | -0,84902 | 0,887995 | structural organization |
| ENSG00000201274 | ABL1 | 0,628283 | 0,264633 | -0,03905 | structural organization |
| ENSG00000202474 | ABR | -1,1632 | -1,18525 | 0,410738 | structural organization |
| ENSG00000241141 | ACTG1 | 2,938815 | 2,798658 | 4,431253 | structural organization |
| ENSG00000183355 | ACTN1 | 0,687377 | 1,066761 | 2,748633 | structural organization |
| ENSG00000123908 | ACTN4 | 2,437775 | 2,628628 | 3,074963 | structural organization |
| ENSG00000239735 | ADD1 | 0,724013 | 1,137125 | 1,665387 | structural organization |
| ENSG00000239935 | ADD2 | 1,916837 | 2,801753 | 1,385947 | structural organization |
| ENSG00000100813 | AIF1 | -1,52785 | -1,04022 | 1,092618 | structural organization |
| ENSG00000181786 | AKT1 | 1,744731 | 1,7493 | 1,843017 | structural organization |
| ENSG00000130402 | AKT2 | 0,845838 | 1,114623 | 1,466699 | structural organization |
| ENSG00000162482 | ALDOA | 1,684066 | 1,497649 | 2,144422 | structural organization |
| ENSG00000230997 | ANK1 | 2,372535 | 2,786528 | 3,679803 | structural organization |
| ENSG00000089053 | ARC | 0,02053 | -0,52985 | -0,10022 | structural organization |
| ENSG00000172932 | ARHGAP26 | -1,11412 | -0,54028 | 0,657941 | structural organization |
| ENSG00000105186 | ARHGAP4 | 0,622939 | 0,568716 | 2,220368 | structural organization |
| ENSG00000213337 | ARHGDIB | 1,467802 | 1,340886 | 3,782263 | structural organization |
| ENSG00000156381 | ARHGEF18 | -0,34164 | -0,27071 | 0,746291 | structural organization |
| ENSG00000064999 | ARHGEF2 | 0,277696 | 0,558861 | 1,424291 | structural organization |
| ENSG00000122359 | ARL2 | 1,685634 | 1,665557 | 0,532962 | structural organization |
| ENSG00000138772 | ARL2BP | -0,0088 | 0,672156 | 0,618491 | structural organization |
| ENSG00000197001 | ARPC5 | 0,563942 | 0,234348 | 1,467766 | structural organization |
| ENSG00000143970 | BCL3 | 0,033871 | -0,10051 | 0,855052 | structural organization |
| ENSG00000138138 | BCL6 | -2,02711 | -1,43335 | 1,082549 | structural organization |
| ENSG00000170653 | BCR | 0,041941 | -0,42103 | 0,59849 | structural organization |
| ENSG00000138363 | BIN3 | -0,7854 | -0,6886 | 0,348232 | structural organization |
| ENSG00000074370 | BMS1 | 0,42556 | -0,30225 | -0,37012 | structural organization |
| ENSG00000165629 | BOP1 | 0,458613 | 0,908028 | 0,438028 | structural organization |
| ENSG00000162384 | CAP1 | 0,603849 | 0,756069 | 2,969983 | structural organization |
| ENSG00000130775 | CAPZA1 | 0,616311 | 0,507446 | 1,654698 | structural organization |
| ENSG00000143612 | CAPZA2 | -0,14992 | 0,032427 | 0,814163 | structural organization |
| ENSG00000162642 | CAPZB | -0,47225 | 0,089259 | 1,362343 | structural organization |
| ENSG00000100220 | CCDC88B | -0,63185 | -0,77073 | 0,361913 | structural organization |
| ENSG00000100220 | CCDC88C | 0,132741 | 0,078741 | 0,580366 | structural organization |
| ENSG00000134075 | CD151 | 0,202209 | 0,064565 | 0,434382 | structural organization |
| ENSG00000134986 | CD47 | 0,418721 | 0,599345 | 1,364788 | structural organization |
| ENSG00000171159 | CDC42 | 0,822311 | 0,676139 | 1,412197 | structural organization |
| ENSG00000197191 | CDC42BPA | 0,406904 | 0,038031 | -0,914 | structural organization |
| ENSG00000116161 | CDH1 | 1,554601 | 1,34436 | 0,719785 | structural organization |
| ENSG00000130643 | CDK5RAP2 | 0,161564 | 0,247605 | 0,023911 | structural organization |
| ENSG00000014216 | CFL1 | 2,473145 | 2,359402 | 3,317503 | structural organization |
| ENSG00000108296 | CLASP1 | 0,419837 | 0,329002 | 1,093833 | structural organization |
| ENSG00000186166 | CLDN15 | 0,014332 | 0,171642 | 0,458638 | structural organization |
| ENSG00000151014 | CLTCL1 | -1,56052 | -1,44192 | 0,163322 | structural organization |
| ENSG00000177697 | CNN2 | 0,873537 | 0,635483 | 2,832943 | structural organization |
| ENSG00000104894 | CNP | 0,843693 | 0,852792 | 0,867374 | structural organization |
| ENSG00000099804 | CORO1C | 1,050924 | 0,961336 | 1,232354 | structural organization |
| ENSG00000105401 | CORO7 | 0,457508 | 0,432611 | 1,650548 | structural organization |
| ENSG00000105810 | CRB3 | 0,396987 | 0,077987 | 0,359841 | structural organization |
| ENSG00000183307 | CRYAA | 0,727103 | 0,200046 | 0,738935 | structural organization |
| ENSG00000135837 | CSGALNACT1 | -2,59927 | -2,68883 | 0,009936 | structural organization |
| ENSG00000133063 | CTNNA1 | 0,106453 | 0,30814 | 0,110241 | structural organization |
| ENSG00000101421 | CTNND1 | 0,502371 | 0,209918 | -0,11173 | structural organization |
| ENSG00000148337 | CXADR | 0,70764 | -0,22354 | -0,92405 | structural organization |
| ENSG00000184113 | CYTH1 | -0,02897 | 0,183486 | 1,208569 | structural organization |
| ENSG00000080822 | CYTH2 | 0,94165 | 0,672698 | 0,663006 | structural organization |
| ENSG00000160318 | CYTH4 | -0,91418 | -0,97975 | 0,404849 | structural organization |
| ENSG00000105472 | CYTIP | -1,64497 | -2,01342 | 0,183501 | structural organization |
| ENSG00000134326 | DCTN2 | 0,510754 | 0,647256 | 0,395698 | structural organization |
| ENSG00000091317 | DCTN3 | -0,04326 | 0,54067 | 1,011584 | structural organization |
| ENSG00000110442 | DEAF1 | 0,489891 | 0,477898 | 0,433554 | structural organization |
| ENSG00000127054 | DIAPH1 | 0,077705 | -0,0221 | 0,831172 | structural organization |
| ENSG00000110090 | DLG1 | -0,15683 | 0,0433 | 0,736947 | structural organization |
| ENSG00000124207 | DOCK2 | -0,4339 | -0,59401 | 1,532582 | structural organization |
| ENSG00000196188 | DYNLL1 | -0,09546 | 0,07288 | -0,82347 | structural organization |
| ENSG00000100448 | DYNLL2 | 1,567453 | 1,270732 | 1,571947 | structural organization |
| ENSG00000036257 | EBNA1BP2 | 0,381497 | 0,430479 | -0,91673 | structural organization |
| ENSG00000164535 | EIF2A | 0,975273 | 0,895524 | 0,0523 | structural organization |
| ENSG00000244038 | EIF6 | 1,050813 | 0,854657 | 0,235407 | structural organization |
| ENSG00000165359 | ELMO1 | 0,488732 | -0,11256 | 0,508145 | structural organization |
| ENSG00000108654 | EML4 | -0,37726 | -0,6064 | 1,100151 | structural organization |
| ENSG00000108654 | ENG | 1,990459 | 2,254978 | 0,202363 | structural organization |
| ENSG00000108654 | EPB41 | 0,202798 | 0,665124 | 1,927227 | structural organization |
| ENSG00000136271 | EPB49 | -0,21564 | 0,384676 | 2,512695 | structural organization |
| ENSG00000119522 | ERAL1 | 0,700121 | 0,176531 | -0,31912 | structural organization |
| ENSG00000072849 | EREG | 0,904591 | 0,450723 | 1,178377 | structural organization |
| ENSG00000006377 | EVL | -0,08061 | -0,22465 | 0,216842 | structural organization |
| ENSG00000102580 | EZH1 | -0,58142 | -0,09212 | 1,228289 | structural organization |
| ENSG00000116675 | F11R | -0,09648 | -0,19133 | 0,636938 | structural organization |
| ENSG00000087470 | FAF1 | 0,34645 | -0,11893 | 0,314086 | structural organization |
| ENSG00000135720 | FAM40A | 0,010323 | -0,11208 | 0,421102 | structural organization |
| ENSG00000100353 | FES | -0,98478 | -0,85539 | 0,102736 | structural organization |
| ENSG00000100353 | FGD3 | -0,10844 | -0,20795 | 1,368281 | structural organization |
| ENSG00000100353 | FHDC1 | -2,0858 | -1,00435 | 2,22829 | structural organization |
| ENSG00000104408 | FHL3 | -0,22534 | -0,42113 | 0,205172 | structural organization |
| ENSG00000149100 | FLNA | 1,376735 | 0,740553 | 2,307608 | structural organization |
| ENSG00000187840 | FMNL1 | 0,507097 | 0,36222 | 2,125274 | structural organization |
| ENSG00000066044 | FSCN1 | 1,364417 | 0,647479 | 0,425136 | structural organization |
| ENSG00000102034 | FTCD | 0,086705 | -0,19119 | 0,342279 | structural organization |
| ENSG00000142227 | FYB | -2,46681 | -2,12534 | 0,773448 | structural organization |
| ENSG00000100393 | GAB1 | 0,423881 | 0,832003 | 0,893612 | structural organization |
| ENSG00000166947 | GABARAP | 1,545243 | 2,14376 | 3,300593 | structural organization |
| ENSG00000198324 | GNA13 | 0,558148 | 0,461584 | 1,38732 | structural organization |
| ENSG00000187866 | GNL2 | 1,048929 | 0,62283 | 0,241136 | structural organization |
| ENSG00000141971 | GNL3L | 0,191695 | -0,04756 | 0,573725 | structural organization |
| ENSG00000180043 | GRB2 | -0,03481 | -0,04055 | 0,7967 | structural organization |
| ENSG00000166595 | GSC2 | -0,04297 | -0,1882 | 0,109063 | structural organization |
| ENSG00000140525 | GSN | -0,37817 | -0,15377 | 2,14367 | structural organization |
| ENSG00000107130 | HIRA | 0,47426 | 0,483281 | 1,394074 | structural organization |
| ENSG00000005955 | HRAS | 0,691947 | 0,46913 | 0,939411 | structural organization |
| ENSG00000101216 | HSP90B1 | 3,048385 | 2,909708 | 2,489153 | structural organization |
| ENSG00000197858 | IER3 | 0,517105 | 0,644293 | 1,876402 | structural organization |
| ENSG00000163870 | IGF2BP2 | 0,864345 | 0,413753 | 0,762326 | structural organization |
| ENSG00000228963 | IL8 | 0,47711 | 0,622657 | 3,389943 | structural organization |
| ENSG00000082701 | ILK | -0,11427 | 0,504137 | 1,331286 | structural organization |
| ENSG00000084207 | INF2 | 0,531364 | 0,480179 | 0,152586 | structural organization |
| ENSG00000122034 | INPP5K | -0,27741 | -0,48862 | 1,337832 | structural organization |
| ENSG00000077235 | INPPL1 | 0,074672 | 0,026736 | 0,74223 | structural organization |
| ENSG00000163041 | ISG20L2 | -0,0546 | 0,058882 | 0,436056 | structural organization |
| ENSG00000092036 | ITGB1 | 1,598123 | 1,290912 | 1,042398 | structural organization |
| ENSG00000138668 | KLHL17 | -0,33287 | -0,48125 | 0,058042 | structural organization |
| ENSG00000165119 | KRT18 | 0,299882 | -0,08821 | 0,512881 | structural organization |
| ENSG00000198973 | LCP1 | 1,465895 | 1,678168 | 4,512233 | structural organization |
| ENSG00000096384 | LIG1 | 0,954649 | 1,11835 | 0,734964 | structural organization |
| ENSG00000106211 | LIMS1 | 0,716371 | 0,811275 | 1,210759 | structural organization |
| ENSG00000105376 | LMNA | 0,736682 | 1,02773 | 0,158468 | structural organization |
| ENSG00000143621 | LST1 | 0,702751 | 0,4892 | 2,768503 | structural organization |
| ENSG00000204084 | MAEA | 0,240075 | 0,650175 | 1,189599 | structural organization |
| ENSG00000144711 | MAP3K11 | -0,04336 | -0,07834 | 0,790828 | structural organization |
| ENSG00000104756 | MICAL1 | -1,50929 | -1,25454 | 1,114915 | structural organization |
| ENSG00000117139 | MINK1 | -0,04096 | 0,112986 | 0,792326 | structural organization |
| ENSG00000119682 | MNX1 | 0,18929 | 0,07002 | 0,367808 | structural organization |
| ENSG00000135709 | MORC3 | -0,99877 | -0,77622 | 0,684701 | structural organization |
| ENSG00000162456 | MRTO4 | 0,32252 | 0,274046 | -1,10369 | structural organization |
| ENSG00000186226 | MYH10 | 0,4479 | 0,678829 | -0,34274 | structural organization |
| ENSG00000240386 | MYH9 | 1,056786 | 1,408656 | 2,973563 | structural organization |
| ENSG00000113368 | NCK2 | -0,30579 | -0,15477 | 1,184009 | structural organization |
| ENSG00000123684 | NDEL1 | 1,047764 | 0,723226 | 2,430355 | structural organization |
| ENSG00000041802 | NEDD8 | 0,259468 | 0,655028 | 0,161426 | structural organization |
| ENSG00000130332 | NF2 | 0,265445 | 0,240491 | -0,04822 | structural organization |
| ENSG00000110514 | NHP2L1 | 0,581754 | 0,057995 | -0,27423 | structural organization |
| ENSG00000182759 | NIP7 | 0,483246 | 0,741436 | 0,267992 | structural organization |
| ENSG00000156009 | NISCH | 0,442668 | 0,41199 | 0,586256 | structural organization |
| ENSG00000101367 | NPM1 | -1,54685 | -1,66528 | -1,30858 | structural organization |
| ENSG00000143797 | NTN1 | 0,355061 | 0,239243 | 0,319586 | structural organization |
| ENSG00000165819 | ONECUT1 | -0,21397 | -0,19709 | 0,356187 | structural organization |
| ENSG00000100427 | PACSIN2 | 0,044295 | 0,044778 | 0,361352 | structural organization |
| ENSG00000130382 | PAK1 | -0,23112 | 0,430701 | 0,482848 | structural organization |
| ENSG00000171843 | PAK4 | 0,089085 | -0,03057 | 0,114496 | structural organization |
| ENSG00000108292 | PALM | 0,111762 | -0,10751 | 0,363025 | structural organization |
| ENSG00000172081 | PARVB | 2,020686 | 2,007719 | 1,092755 | structural organization |
| ENSG00000128309 | PCNT | 0,088698 | 0,054556 | 1,180306 | structural organization |
| ENSG00000171421 | PDLIM7 | -0,15339 | -0,31381 | 0,577821 | structural organization |
| ENSG00000116221 | PDPK1 | -0,11665 | -0,05766 | 1,372814 | structural organization |
| ENSG00000111639 | PDXP | 0,616772 | 1,04907 | 1,331409 | structural organization |
| ENSG00000162910 | PDZD8 | 1,345953 | 1,156669 | 1,654939 | structural organization |
| ENSG00000122140 | PES1 | 0,228878 | 0,332694 | -0,66016 | structural organization |
| ENSG00000135097 | PFN1 | 3,616885 | 3,599913 | 4,457043 | structural organization |
| ENSG00000100330 | PHIP | 1,271597 | 0,624388 | 1,085994 | structural organization |
| ENSG00000136504 | PITX1 | -0,29637 | -0,51349 | 0,086409 | structural organization |
| ENSG00000136504 | PKD1 | -0,54903 | -0,28785 | 0,011165 | structural organization |
| ENSG00000105402 | PLEK | -4,57632 | -4,61408 | -3,64006 | structural organization |
| ENSG00000147813 | PLEK2 | -0,92412 | -0,7598 | 0,60184 | structural organization |
| ENSG00000121579 | PML | 0,500705 | 0,126281 | 0,002846 | structural organization |
| ENSG00000235568 | PPP1CB | 0,475246 | 0,508929 | 1,449426 | structural organization |
| ENSG00000176953 | PPP1R12A | 1,314625 | 1,052684 | 1,848561 | structural organization |
| ENSG00000104825 | PPP2CA | 0,801002 | 0,89556 | 1,927892 | structural organization |
| ENSG00000167604 | PPP2R1A | 1,738742 | 1,342914 | 1,426215 | structural organization |
| ENSG00000131669 | PPP4C | 0,681341 | 0,906346 | 1,443857 | structural organization |
| ENSG00000149308 | PRPF40A | 0,902609 | 0,680701 | 0,585361 | structural organization |
| ENSG00000162231 | PTK2B | -0,54877 | -0,49445 | 1,579712 | structural organization |
| ENSG00000104904 | PTPN1 | 0,802706 | 0,346428 | 0,952795 | structural organization |
| ENSG00000104904 | PTPN11 | -0,01485 | -0,19278 | 0,059306 | structural organization |
| ENSG00000139579 | PTPN6 | 0,034339 | -0,08319 | 2,089326 | structural organization |
| ENSG00000105953 | PTPRJ | -1,26442 | -1,32197 | 0,895295 | structural organization |
| ENSG00000175938 | PXN | -0,39638 | -0,55223 | 0,976398 | structural organization |
| ENSG00000120137 | RAC2 | 1,738668 | 1,730232 | 3,622503 | structural organization |
| ENSG00000073150 | RAC3 | 0,153894 | -0,0864 | 0,313046 | structural organization |
| ENSG00000116288 | RAF1 | 1,003384 | 0,785839 | 1,551141 | structural organization |
| ENSG00000102699 | RANBP10 | 1,541513 | 1,89769 | 3,221703 | structural organization |
| ENSG00000151883 | RANBP9 | 1,532934 | 1,265797 | 2,507476 | structural organization |
| ENSG00000215193 | RCL1 | 0,315959 | 0,898525 | 1,214042 | structural organization |
| ENSG00000241973 | RHOA | 3,880905 | 3,782198 | 4,566043 | structural organization |
| ENSG00000033800 | RHOF | -0,30393 | -0,2517 | 1,324496 | structural organization |
| ENSG00000105229 | RHOG | 1,444602 | 1,81451 | 2,711483 | structural organization |
| ENSG00000078142 | RICTOR | 0,52517 | -0,43881 | 0,73838 | structural organization |
| ENSG00000154822 | RNF19A | 0,732171 | 0,482481 | 2,343768 | structural organization |
| ENSG00000106397 | ROCK1 | -0,40211 | -0,50675 | 1,130564 | structural organization |
| ENSG00000102007 | ROD1 | 0,586595 | 0,520093 | 1,180895 | structural organization |
| ENSG00000117751 | RPL10A | 2,461475 | 2,492308 | 1,332826 | structural organization |
| ENSG00000130711 | RPLP0 | 3,595875 | 3,612738 | 3,235583 | structural organization |
| ENSG00000174231 | RSL24D1 | -0,01625 | -0,49224 | 0,11309 | structural organization |
| ENSG00000174231 | RSU1 | 1,120966 | 1,238919 | 1,687029 | structural organization |
| ENSG00000174231 | RTN4 | 0,341362 | 0,286563 | 1,355419 | structural organization |
| ENSG00000174231 | S100A9 | 2,286125 | 2,513213 | 5,100173 | structural organization |
| ENSG00000161057 | SDCBP | 0,817428 | 1,082678 | 2,12127 | structural organization |
| ENSG00000111540 | SH3KBP1 | -0,23431 | -0,17776 | 1,110092 | structural organization |
| ENSG00000166128 | SHC1 | 0,745853 | 0,714629 | 1,123673 | structural organization |
| ENSG00000132341 | SIPA1 | 0,012049 | 0,245423 | 1,750781 | structural organization |
| ENSG00000141084 | SIPA1L1 | -1,93425 | -1,47516 | 0,098334 | structural organization |
| ENSG00000010017 | SIRPA | -0,78799 | -0,98075 | 0,551265 | structural organization |
| ENSG00000100401 | SIRPB1 | -1,83335 | -1,08115 | 1,738767 | structural organization |
| ENSG00000158987 | SIX2 | 0,58316 | 0,365995 | 0,687199 | structural organization |
| ENSG00000105122 | SKAP2 | -1,07187 | -0,61923 | 0,660311 | structural organization |
| ENSG00000076043 | SLC40A1 | 1,300528 | 0,951146 | 1,607948 | structural organization |
| ENSG00000100263 | SLC9A3R1 | 0,906412 | 0,74364 | 1,838424 | structural organization |
| ENSG00000212695 | SON | 2,034219 | 1,811847 | 2,728343 | structural organization |
| ENSG00000223803 | SOX9 | 0,785073 | 0,250017 | 0,964454 | structural organization |
| ENSG00000235244 | SPN | 1,443186 | 1,370412 | 0,861922 | structural organization |
| ENSG00000139239 | SPTA1 | 1,470084 | 2,162233 | 3,578063 | structural organization |
| ENSG00000242071 | SPTB | 0,254949 | 1,198173 | 2,157092 | structural organization |
| ENSG00000113811 | SPTBN1 | -1,77354 | -1,5444 | 0,297081 | structural organization |
| ENSG00000228526 | SRF | 1,40743 | 1,427831 | 1,490989 | structural organization |
| ENSG00000142676 | SSH2 | -0,86563 | -0,74392 | 0,07609 | structural organization |
| ENSG00000108298 | STAT5A | 1,349442 | 0,847192 | -0,11737 | structural organization |
| ENSG00000114391 | STAT5B | 0,805869 | 0,878153 | 0,921595 | structural organization |
| ENSG00000108107 | STK25 | 1,86226 | 1,700689 | 0,688308 | structural organization |
| ENSG00000110700 | SUPT7L | 0,008466 | -0,1329 | 0,238193 | structural organization |
| ENSG00000186468 | SVIL | -1,94489 | -1,70685 | 0,110865 | structural organization |
| ENSG00000196183 | TACC1 | 0,815187 | 0,986721 | 1,60053 | structural organization |
| ENSG00000149273 | TACC3 | -0,00652 | 0,447976 | 0,647874 | structural organization |
| ENSG00000198034 | TAF3 | 0,338532 | 0,376357 | 0,328293 | structural organization |
| ENSG00000083845 | TAF8 | 0,385221 | 0,066944 | 0,462005 | structural organization |
| ENSG00000170889 | TAOK2 | -0,09706 | -0,49007 | 0,010464 | structural organization |
| ENSG00000150459 | TEKT4 | -0,21436 | -0,41239 | 0,041194 | structural organization |
| ENSG00000104835 | TESK1 | 0,186649 | 0,28778 | 0,830499 | structural organization |
| ENSG00000175467 | TGFB1 | -0,06435 | 0,069337 | 1,570909 | structural organization |
| ENSG00000139718 | TMSB10 | 3,259605 | 2,069784 | 2,923933 | structural organization |
| ENSG00000111786 | TNXB | -0,71561 | -0,19852 | 0,520168 | structural organization |
| ENSG00000160410 | TPM1 | 0,204145 | -0,05282 | -0,33722 | structural organization |
| ENSG00000137700 | TUBA1A | -0,27724 | -0,35483 | 0,774084 | structural organization |
| ENSG00000111371 | TUBA1B | 2,774675 | 2,923088 | 2,903473 | structural organization |
| ENSG00000157637 | TUBA1C | 1,121784 | 1,605301 | 1,23729 | structural organization |
| ENSG00000134294 | TUBB | 4,167375 | 4,484843 | 4,386993 | structural organization |
| ENSG00000029364 | TUBG1 | 2,308565 | 3,134313 | 2,066789 | structural organization |
| ENSG00000168003 | TUBGCP3 | -0,22302 | 0,147072 | 0,191733 | structural organization |
| ENSG00000138449 | TUBGCP4 | -0,51009 | 0,123776 | -0,62109 | structural organization |
| ENSG00000133065 | TUBGCP6 | -0,10813 | 0,119281 | 0,456482 | structural organization |
| ENSG00000176463 | TYROBP | 1,725917 | 1,907858 | 4,353353 | structural organization |
| ENSG00000004777 | UXT | 0,391326 | 0,678029 | 0,483544 | structural organization |
| ENSG00000089006 | VASP | 0,417501 | 0,405874 | 1,917086 | structural organization |
| ENSG00000115306 | WAS | -0,40565 | -0,29186 | 1,589188 | structural organization |
| ENSG00000100596 | WASF2 | 0,331668 | 0,538407 | 1,534813 | structural organization |
| ENSG00000137767 | WASL | -0,3501 | -0,52045 | 0,061974 | structural organization |
| ENSG00000153037 | WHSC1 | -0,08639 | 0,430356 | 0,339302 | structural organization |
| ENSG00000184343 | WIPF1 | 0,403937 | 0,614627 | 1,873069 | structural organization |
| ENSG00000138434 | WTIP | 0,149442 | -0,14584 | 0,282074 | structural organization |
| ENSG00000166337 | ZMYM3 | 0,191924 | -0,34253 | -0,77428 | structural organization |
| ENSG00000166337 | ZMYM4 | 0,036719 | -0,08026 | -0,85744 | structural organization |
| ENSG00000196507 | ZRANB1 | -0,18343 | 0,131572 | 1,30673 | structural organization |
| ENSG00000212499 | ABCC1 | 0,38322 | -0,02619 | 0,33209 | metabolic process |
| ENSG00000200873 | ABHD5 | 0,611242 | 1,246371 | 2,404884 | metabolic process |
| ENSG00000239846 | ACAA1 | -0,31362 | -0,40902 | 0,325382 | metabolic process |
| ENSG00000244628 | ACAD8 | -0,81061 | -0,7381 | 0,157308 | metabolic process |
| ENSG00000153815 | ACAD9 | -0,03595 | 0,253903 | -0,4571 | metabolic process |
| ENSG00000172809 | ACADM | -0,33898 | 0,070605 | -0,55345 | metabolic process |
| ENSG00000213373 | ACADS | -0,01472 | 0,071806 | 0,194801 | metabolic process |
| ENSG00000224411 | ACADVL | 1,962135 | 1,70295 | 1,680903 | metabolic process |
| ENSG00000240649 | ACIN1 | 0,84472 | 0,636077 | 1,132513 | metabolic process |
| ENSG00000239768 | ACLY | 1,153139 | 0,906135 | 0,630473 | metabolic process |
| ENSG00000241897 | ACO2 | 0,869003 | 1,092961 | 1,170174 | metabolic process |
| ENSG00000240168 | ACOT7 | 0,009935 | -0,24956 | 0,157887 | metabolic process |
| ENSG00000243301 | ACOT8 | -0,34547 | -0,31498 | 0,024091 | metabolic process |
| ENSG00000236562 | ADCY7 | 0,46659 | 0,806491 | 0,198703 | metabolic process |
| ENSG00000113141 | ADIPOR1 | 1,704679 | 1,827704 | 3,301053 | metabolic process |
| ENSG00000242277 | ADPGK | 0,034525 | -0,36998 | 0,779282 | metabolic process |
| ENSG00000151539 | AGPAT1 | 0,422867 | 0,602745 | 0,058247 | metabolic process |
| ENSG00000060971 | AGPAT2 | -0,38924 | -0,41838 | 0,444509 | metabolic process |
| ENSG00000151498 | AGPAT4 | -2,10942 | -1,91842 | 0,779284 | metabolic process |
| ENSG00000181786 | AKT1 | 1,744731 | 1,7493 | 1,843017 | metabolic process |
| ENSG00000162482 | ALDOA | 1,684066 | 1,497649 | 2,144422 | metabolic process |
| ENSG00000241447 | AMN | 0,290926 | 0,055993 | 0,634268 | metabolic process |
| ENSG00000243482 | ANPEP | -1,11249 | -0,27443 | 1,555631 | metabolic process |
| ENSG00000243173 | ANXA1 | 1,757694 | 1,972089 | 5,086203 | metabolic process |
| ENSG00000243212 | AOAH | -2,17787 | -2,12423 | 0,676775 | metabolic process |
| ENSG00000104899 | APEX1 | 1,660278 | 1,881638 | 1,026232 | metabolic process |
| ENSG00000089053 | APOC1 | 1,053643 | 0,108114 | -0,77566 | metabolic process |
| ENSG00000089053 | AQP9 | -2,23794 | -1,8757 | 0,533126 | metabolic process |
| ENSG00000131503 | ARG1 | -1,8672 | -1,62827 | 2,182534 | metabolic process |
| ENSG00000161203 | ARV1 | -0,59665 | 0,077392 | -1,21117 | metabolic process |
| ENSG00000065000 | ASAH1 | -0,14612 | 0,155141 | 1,810379 | metabolic process |
| ENSG00000143761 | ATP1A1 | 1,448474 | 1,801546 | 1,631183 | metabolic process |
| ENSG00000143761 | ATP1B2 | 0,211138 | 0,88431 | 1,094578 | metabolic process |
| ENSG00000143761 | ATP2A2 | 0,762305 | 0,207232 | 0,192807 | metabolic process |
| ENSG00000143761 | ATP2A3 | -0,37413 | -0,05989 | 1,944912 | metabolic process |
| ENSG00000143761 | ATP2B4 | 0,407696 | 1,177625 | 0,528868 | metabolic process |
| ENSG00000134287 | ATP5A1 | 1,410902 | 1,390718 | 0,492555 | metabolic process |
| ENSG00000134287 | ATP5B | 2,648765 | 2,491138 | 2,225232 | metabolic process |
| ENSG00000134287 | ATP5C1 | 0,759726 | 0,645546 | 0,067222 | metabolic process |
| ENSG00000134287 | ATP5D | 1,228063 | 1,351332 | 1,282373 | metabolic process |
| ENSG00000134287 | ATP5E | 0,629059 | 0,728904 | 0,47356 | metabolic process |
| ENSG00000134287 | ATP5F1 | 0,855353 | 0,699196 | 0,723771 | metabolic process |
| ENSG00000004059 | ATP5H | 0,884735 | 0,871697 | 0,826748 | metabolic process |
| ENSG00000165527 | ATP5I | 0,987092 | 0,978266 | 0,826565 | metabolic process |
| ENSG00000101199 | ATP5J2 | 1,261065 | 1,086918 | 0,973626 | metabolic process |
| ENSG00000149182 | ATP5L | 1,10603 | 0,979927 | 1,09232 | metabolic process |
| ENSG00000066777 | ATP5O | 1,635497 | 1,39386 | 0,983243 | metabolic process |
| ENSG00000225485 | ATP6V0D1 | 0,812819 | 1,554646 | 2,221935 | metabolic process |
| ENSG00000141522 | ATP7A | -1,39929 | -0,60355 | 0,265894 | metabolic process |
| ENSG00000111348 | ATP7B | -0,5153 | 0,007061 | -1,23453 | metabolic process |
| ENSG00000104880 | ATPIF1 | 0,852117 | 1,200774 | 0,579499 | metabolic process |
| ENSG00000196843 | AUP1 | 2,010734 | 2,003725 | 2,165639 | metabolic process |
| ENSG00000170540 | AZIN1 | -0,66785 | 0,037509 | 1,108936 | metabolic process |
| ENSG00000134108 | B3GNT5 | -1,20818 | -1,31446 | 0,785187 | metabolic process |
| ENSG00000129691 | BCKDHA | 0,326132 | 0,280555 | 0,203693 | metabolic process |
| ENSG00000068650 | BLMH | 0,086584 | -0,7692 | -1,38066 | metabolic process |
| ENSG00000133657 | BLVRA | 0,057875 | 0,342839 | -0,08875 | metabolic process |
| ENSG00000099624 | BPGM | -0,67015 | -0,51739 | 1,920607 | metabolic process |
| ENSG00000103121 | C6orf108 | 0,892512 | 0,822074 | 0,118298 | metabolic process |
| ENSG00000104979 | CALM2 | -0,15785 | 0,008678 | 0,01243 | metabolic process |
| ENSG00000094975 | CASP2 | 0,625451 | 0,6815 | 1,111503 | metabolic process |
| ENSG00000157870 | CASP8 | -0,39152 | -0,54729 | 1,044953 | metabolic process |
| ENSG00000186825 | CCS | 0,205928 | 0,374123 | 1,782976 | metabolic process |
| ENSG00000173769 | CD36 | 1,447629 | 2,079766 | 1,79 | metabolic process |
| ENSG00000127022 | CENPV | 0,432841 | 0,370907 | -0,93183 | metabolic process |
| ENSG00000127022 | CES1 | 0,438423 | -0,21846 | 0,606523 | metabolic process |
| ENSG00000077549 | CHKA | 0,389236 | -0,14491 | 1,089945 | metabolic process |
| ENSG00000134905 | CHPT1 | 1,978573 | 2,109681 | 2,563682 | metabolic process |
| ENSG00000121691 | CHST2 | 0,500206 | 0,604689 | 1,658271 | metabolic process |
| ENSG00000104894 | CNP | 0,843693 | 0,852792 | 0,867374 | metabolic process |
| ENSG00000196352 | COIL | 0,592699 | 0,17892 | -0,22706 | metabolic process |
| ENSG00000163171 | COX4I1 | 1,706594 | 1,432379 | 1,680475 | metabolic process |
| ENSG00000197622 | COX6A2 | 0,041951 | -0,21679 | 0,274723 | metabolic process |
| ENSG00000158985 | COX6B1 | 1,570718 | 1,433599 | 1,52093 | metabolic process |
| ENSG00000096401 | COX8A | 1,168068 | 1,65175 | 1,754054 | metabolic process |
| ENSG00000164649 | CPNE3 | -0,15778 | 0,265004 | 2,615766 | metabolic process |
| ENSG00000134690 | CPOX | 0,073352 | 0,571183 | 1,300815 | metabolic process |
| ENSG00000135446 | CPT1A | 1,259766 | 1,277313 | 1,180012 | metabolic process |
| ENSG00000108465 | CRAT | -0,20526 | -0,09486 | 0,189538 | metabolic process |
| ENSG00000173575 | CTBP1 | 1,575714 | 1,70446 | 1,794402 | metabolic process |
| ENSG00000123975 | CYB5R4 | -1,28982 | -1,19345 | 0,468049 | metabolic process |
| ENSG00000105270 | DAGLB | -0,79544 | -0,92046 | 0,027774 | metabolic process |
| ENSG00000148842 | DCXR | 1,174704 | 1,636604 | 1,735035 | metabolic process |
| ENSG00000141030 | DEGS2 | 0,614561 | 0,506202 | 0,963553 | metabolic process |
| ENSG00000006695 | DGAT1 | 0,327272 | 0,611511 | 1,73132 | metabolic process |
| ENSG00000106066 | DLST | 0,967292 | 0,635878 | 0,879277 | metabolic process |
| ENSG00000102974 | DUS1L | 0,735826 | 0,944074 | 0,642643 | metabolic process |
| ENSG00000139842 | EBP | -0,18331 | -0,21746 | 0,06107 | metabolic process |
| ENSG00000121966 | ECH1 | 2,21292 | 2,462678 | 1,045897 | metabolic process |
| ENSG00000123136 | ELMO2 | -0,62755 | -0,68833 | -0,01911 | metabolic process |
| ENSG00000108654 | ENO1 | 2,214304 | 2,135031 | 2,767203 | metabolic process |
| ENSG00000108654 | ENTPD1 | -1,90182 | -1,47762 | 1,874152 | metabolic process |
| ENSG00000105339 | ERAP1 | -0,54673 | -0,75799 | 0,110539 | metabolic process |
| ENSG00000136986 | ERCC3 | 0,278499 | -0,13482 | -0,61082 | metabolic process |
| ENSG00000149091 | ERO1L | -1,89065 | -1,69303 | 0,734189 | metabolic process |
| ENSG00000162496 | ESPL1 | -0,89472 | -0,09935 | 0,322015 | metabolic process |
| ENSG00000213551 | FADS2 | 2,051675 | 1,865706 | -0,46029 | metabolic process |
| ENSG00000183403 | FADS3 | 0,426916 | -0,07889 | -0,10076 | metabolic process |
| ENSG00000228502 | FASN | 0,450025 | 0,267953 | 0,027448 | metabolic process |
| ENSG00000100353 | FECH | 0,741312 | 1,409279 | 2,834653 | metabolic process |
| ENSG00000100353 | FEN1 | 0,583033 | 1,080505 | 0,425803 | metabolic process |
| ENSG00000102119 | FXN | 2,153658 | 1,963684 | 0,976789 | metabolic process |
| ENSG00000120533 | G6PD | 0,544091 | 0,619389 | 2,207421 | metabolic process |
| ENSG00000124882 | GAPDH | 4,741975 | 4,569798 | 5,475943 | metabolic process |
| ENSG00000107566 | GBA | -0,76884 | -0,52919 | 0,668551 | metabolic process |
| ENSG00000089248 | GBGT1 | 0,863366 | 0,619971 | 0,460883 | metabolic process |
| ENSG00000139641 | GDE1 | -0,51646 | -0,87039 | 0,793098 | metabolic process |
| ENSG00000105755 | GDPD5 | -0,67426 | -0,49348 | 0,624095 | metabolic process |
| ENSG00000158769 | GLO1 | -0,13046 | 0,118308 | -0,1771 | metabolic process |
| ENSG00000183688 | GM2A | 0,007054 | -0,43852 | 0,166482 | metabolic process |
| ENSG00000184731 | GNAS | 1,636058 | 1,513349 | 1,921752 | metabolic process |
| ENSG00000121104 | GNB1 | 2,701015 | 2,793803 | 3,123313 | metabolic process |
| ENSG00000138439 | GNB2 | 2,127556 | 2,206365 | 2,614116 | metabolic process |
| ENSG00000048828 | GNG2 | -0,94799 | -1,14524 | 0,508175 | metabolic process |
| ENSG00000188938 | GNG5 | 1,68416 | 1,212268 | 1,886717 | metabolic process |
| ENSG00000189292 | GOT2 | 1,623185 | 1,636472 | 0,829975 | metabolic process |
| ENSG00000172538 | GPI | 1,851985 | 1,553755 | 2,881413 | metabolic process |
| ENSG00000039523 | GPX4 | 2,472475 | 2,080876 | 1,726283 | metabolic process |
| ENSG00000164896 | GSTM1 | 0,462051 | 0,317366 | -0,62908 | metabolic process |
| ENSG00000149806 | GSTP1 | 1,582459 | 1,540584 | 2,045008 | metabolic process |
| ENSG00000164117 | GYG1 | -1,61608 | -1,15324 | 1,826606 | metabolic process |
| ENSG00000159069 | GYS1 | -0,74588 | -0,60793 | 0,59744 | metabolic process |
| ENSG00000149781 | H6PD | -1,01227 | -0,99155 | 0,121818 | metabolic process |
| ENSG00000127084 | HADHB | 0,117433 | 0,242237 | 0,638232 | metabolic process |
| ENSG00000070388 | HAGH | 1,901599 | 2,328088 | 2,787573 | metabolic process |
| ENSG00000139112 | HK1 | 0,064225 | 0,501201 | 0,523355 | metabolic process |
| ENSG00000034713 | HK2 | -0,59847 | -0,76009 | 0,855401 | metabolic process |
| ENSG00000204681 | HK3 | -1,51912 | -1,45259 | 0,731748 | metabolic process |
| ENSG00000165678 | HS3ST6 | 0,638603 | 0,38238 | 0,714732 | metabolic process |
| ENSG00000130309 | HSD17B10 | 0,074858 | 0,38529 | 0,138968 | metabolic process |
| ENSG00000105373 | HSD3B7 | 0,193973 | 0,243914 | 0,119894 | metabolic process |
| ENSG00000196743 | HSP90AA1 | 2,814285 | 3,197468 | 2,508311 | metabolic process |
| ENSG00000173540 | HSPA5 | 2,784725 | 3,009378 | 2,727353 | metabolic process |
| ENSG00000137198 | HSPA8 | 4,062365 | 4,496873 | 3,797413 | metabolic process |
| ENSG00000060558 | HSPD1 | 1,251503 | 1,363158 | -0,16699 | metabolic process |
| ENSG00000147533 | IDH3B | 0,516057 | 0,177753 | 0,039251 | metabolic process |
| ENSG00000173905 | IDH3G | 0,958235 | 0,929997 | 1,418432 | metabolic process |
| ENSG00000104687 | IMPDH1 | 0,331972 | 0,114112 | 0,706929 | metabolic process |
| ENSG00000197448 | IMPDH2 | 1,565424 | 1,441749 | 0,109858 | metabolic process |
| ENSG00000169840 | INO80 | 0,099762 | -0,19205 | 0,095676 | metabolic process |
| ENSG00000077235 | INPPL1 | 0,074672 | 0,026736 | 0,74223 | metabolic process |
| ENSG00000115207 | INSIG1 | -0,66406 | -0,60706 | 0,267349 | metabolic process |
| ENSG00000143774 | IQGAP1 | -0,1517 | -0,07755 | 1,548713 | metabolic process |
| ENSG00000113648 | IRS2 | 0,25944 | 0,406175 | 1,879572 | metabolic process |
| ENSG00000086506 | ITPK1 | -0,54664 | -0,59668 | 0,16125 | metabolic process |
| ENSG00000156875 | KAT2A | 1,357626 | 0,842454 | 0,031347 | metabolic process |
| ENSG00000148110 | KAT2B | 0,078964 | 0,711434 | 2,296135 | metabolic process |
| ENSG00000169567 | KCNG2 | 0,032609 | -0,04855 | 0,334042 | metabolic process |
| ENSG00000197459 | KDM5A | -0,17244 | -0,54952 | 0,496858 | metabolic process |
| ENSG00000197903 | KDM5B | -0,30502 | -0,29566 | 0,431277 | metabolic process |
| ENSG00000196374 | KDM5C | 0,698363 | 0,584327 | 1,234765 | metabolic process |
| ENSG00000233822 | KDM5D | 0,070798 | -2,74616 | -2,34746 | metabolic process |
| ENSG00000135486 | KIF1B | -1,33452 | -1,44043 | 0,333004 | metabolic process |
| ENSG00000135116 | LBR | 0,546245 | 1,225372 | 1,681931 | metabolic process |
| ENSG00000208037 | LCLAT1 | -0,73595 | 0,075842 | -0,5974 | metabolic process |
| ENSG00000207709 | LDHA | 1,592729 | 1,743112 | 3,088213 | metabolic process |
| ENSG00000207550 | LDHB | 2,009538 | 1,716029 | 0,105582 | metabolic process |
| ENSG00000010404 | LOXL1 | 0,103415 | -0,09756 | 0,459991 | metabolic process |
| ENSG00000160888 | LPCAT1 | -0,82314 | -0,81675 | 0,351464 | metabolic process |
| ENSG00000137331 | LPCAT2 | -1,83781 | -1,72232 | 1,435923 | metabolic process |
| ENSG00000162783 | LPGAT1 | -1,99391 | -1,51325 | 0,397459 | metabolic process |
| ENSG00000188483 | LPIN2 | -0,15066 | 0,051206 | 1,82502 | metabolic process |
| ENSG00000214706 | LRP10 | -0,28186 | 0,139702 | 2,729033 | metabolic process |
| ENSG00000140443 | LRP8 | 0,371794 | 0,082237 | -0,06143 | metabolic process |
| ENSG00000143621 | LTF | 0,986328 | 2,287191 | 5,094793 | metabolic process |
| ENSG00000132305 | LYPLA2 | 1,147529 | 1,16668 | 1,557639 | metabolic process |
| ENSG00000143624 | MAN1A1 | -0,84521 | -0,29055 | 2,294818 | metabolic process |
| ENSG00000140968 | MAPK14 | -0,34239 | -0,34944 | 0,8961 | metabolic process |
| ENSG00000144668 | MARCKS | -0,36464 | -0,52028 | 0,524622 | metabolic process |
| ENSG00000210082 | MBTPS1 | 0,196244 | 0,08552 | 0,658259 | metabolic process |
| ENSG00000152409 | MDH2 | 2,136092 | 2,099418 | 1,19973 | metabolic process |
| ENSG00000167476 | MDN1 | 0,527559 | 0,431007 | -0,3838 | metabolic process |
| ENSG00000143543 | ME3 | 0,012594 | -0,29259 | -0,41434 | metabolic process |
| ENSG00000180901 | MGST3 | 1,157142 | 1,798741 | 2,171379 | metabolic process |
| ENSG00000104756 | MICAL1 | -1,50929 | -1,25454 | 1,114915 | metabolic process |
| ENSG00000012817 | MIOX | 0,118516 | -0,41585 | 0,26782 | metabolic process |
| ENSG00000121774 | MLH1 | 0,02107 | 0,370605 | 0,223641 | metabolic process |
| ENSG00000088247 | MLX | 0,006558 | 0,305766 | 0,808367 | metabolic process |
| ENSG00000081791 | MMP25 | -0,48274 | -0,1915 | 1,020694 | metabolic process |
| ENSG00000182149 | MMP8 | -0,46318 | 1,220591 | 4,793243 | metabolic process |
| ENSG00000131149 | MMP9 | 0,619537 | 1,557727 | 4,460793 | metabolic process |
| ENSG00000129933 | MOSC1 | -1,57924 | -1,57016 | 0,348405 | metabolic process |
| ENSG00000109265 | MPO | -0,1946 | 1,540662 | 3,079153 | metabolic process |
| ENSG00000111057 | MSRA | -0,93298 | -0,98974 | 0,405146 | metabolic process |
| ENSG00000204571 | MTHFD1 | 0,759156 | 0,713801 | -0,50318 | metabolic process |
| ENSG00000188779 | MYC | 2,287605 | 1,903519 | 0,140081 | metabolic process |
| ENSG00000240386 | MYH9 | 1,056786 | 1,408656 | 2,973563 | metabolic process |
| ENSG00000130164 | MYO9B | 0,330266 | 0,418206 | 1,675654 | metabolic process |
| ENSG00000167615 | NAA30 | -0,08621 | -0,05145 | 0,100056 | metabolic process |
| ENSG00000133317 | NADK | 0,132192 | 0,343801 | 1,325025 | metabolic process |
| ENSG00000116977 | NAGLU | 0,171445 | -0,08033 | 0,357163 | metabolic process |
| ENSG00000143486 | NAGS | -0,07331 | -0,23377 | 0,155078 | metabolic process |
| ENSG00000131042 | NAT10 | 0,61456 | 0,28322 | -0,81445 | metabolic process |
| ENSG00000136490 | NAT14 | 0,262945 | -0,37872 | 0,033164 | metabolic process |
| ENSG00000145012 | NDOR1 | 0,091543 | 0,06775 | 0,187632 | metabolic process |
| ENSG00000163956 | NDUFA9 | 0,115586 | 0,251834 | 0,401898 | metabolic process |
| ENSG00000132128 | NDUFS2 | 0,40684 | 0,203542 | 0,654889 | metabolic process |
| ENSG00000163428 | NDUFS7 | 0,673187 | 0,811882 | 0,77879 | metabolic process |
| ENSG00000104866 | NDUFV1 | 1,379462 | 1,458776 | 0,794606 | metabolic process |
| ENSG00000130520 | NEU1 | -0,7405 | -0,62826 | 0,315276 | metabolic process |
| ENSG00000156009 | NISCH | 0,442668 | 0,41199 | 0,586256 | metabolic process |
| ENSG00000034152 | NNT | -0,1371 | 0,281206 | 0,205411 | metabolic process |
| ENSG00000168906 | NRF1 | -0,4058 | -0,4498 | 0,090397 | metabolic process |
| ENSG00000068305 | OAZ1 | 1,961293 | 2,573547 | 3,732503 | metabolic process |
| ENSG00000064489 | OAZ2 | -0,53603 | -0,86206 | 0,204224 | metabolic process |
| ENSG00000106780 | ODC1 | 1,187781 | 1,399508 | 1,394526 | metabolic process |
| ENSG00000188095 | OGDH | 0,057518 | 0,187379 | 0,797983 | metabolic process |
| ENSG00000165819 | ONECUT1 | -0,21397 | -0,19709 | 0,356187 | metabolic process |
| ENSG00000167470 | OXA1L | 2,109469 | 2,097131 | 1,982869 | metabolic process |
| ENSG00000155438 | P4HB | 2,596785 | 2,733013 | 2,917943 | metabolic process |
| ENSG00000076242 | PAFAH1B2 | 0,246413 | 0,295063 | 1,200451 | metabolic process |
| ENSG00000118058 | PAFAH1B3 | 1,150884 | 1,116051 | 0,368204 | metabolic process |
| ENSG00000100985 | PARK7 | -1,20596 | -1,37703 | -1,91069 | metabolic process |
| ENSG00000143314 | PDHA1 | 0,096001 | 0,16142 | 0,230269 | metabolic process |
| ENSG00000182180 | PEMT | 0,192465 | 0,031291 | -0,04906 | metabolic process |
| ENSG00000096080 | PEPD | 0,201803 | 0,193193 | -0,26203 | metabolic process |
| ENSG00000053372 | PFKFB3 | -0,55202 | -0,26365 | 2,196499 | metabolic process |
| ENSG00000149516 | PFKL | 1,038312 | 1,036378 | 1,065418 | metabolic process |
| ENSG00000204410 | PFKM | -0,66182 | 0,049149 | -0,65817 | metabolic process |
| ENSG00000174579 | PGD | 1,636705 | 1,818731 | 3,316083 | metabolic process |
| ENSG00000162006 | PGK1 | 1,209798 | 1,41763 | 2,797833 | metabolic process |
| ENSG00000175806 | PGM2 | -0,17377 | 0,024269 | 0,093584 | metabolic process |
| ENSG00000108389 | PHKA2 | -0,17122 | -0,35299 | 0,233713 | metabolic process |
| ENSG00000198793 | PHKB | -0,1913 | -0,14402 | 0,119546 | metabolic process |
| ENSG00000105887 | PHKG2 | 0,288028 | 0,712281 | 1,067551 | metabolic process |
| ENSG00000205277 | PHOSPHO1 | -1,4047 | -0,91191 | 0,866265 | metabolic process |
| ENSG00000118680 | PIK3R1 | 0,168851 | -0,06475 | 1,171885 | metabolic process |
| ENSG00000136504 | PITPNA | 0,650854 | 1,044585 | 1,100761 | metabolic process |
| ENSG00000136504 | PITPNM1 | -1,01326 | -1,21031 | 0,466702 | metabolic process |
| ENSG00000136504 | PKLR | 1,268113 | 1,627613 | -0,2029 | metabolic process |
| ENSG00000136504 | PKM2 | -0,7698 | -0,03269 | 2,284863 | metabolic process |
| ENSG00000108784 | PLCH2 | -0,38722 | -0,5123 | 0,042903 | metabolic process |
| ENSG00000161653 | PLCL2 | -2,21904 | -2,35537 | 0,049654 | metabolic process |
| ENSG00000134440 | PLOD3 | 0,023908 | 0,406373 | 0,363541 | metabolic process |
| ENSG00000109805 | PNPLA2 | 0,843548 | 0,990302 | 2,623279 | metabolic process |
| ENSG00000146918 | PNPLA6 | -0,47859 | -0,04811 | 1,238433 | metabolic process |
| ENSG00000121152 | PNPLA8 | -1,93095 | -1,59277 | 0,249142 | metabolic process |
| ENSG00000151366 | PPA1 | 0,448988 | 0,52646 | -0,28445 | metabolic process |
| ENSG00000204386 | PPOX | -0,63862 | -0,30562 | 0,297148 | metabolic process |
| ENSG00000186575 | PPP1CA | 1,511916 | 1,739941 | 1,925138 | metabolic process |
| ENSG00000235568 | PPP1CB | 0,475246 | 0,508929 | 1,449426 | metabolic process |
| ENSG00000102908 | PPP1CC | 1,734339 | 1,59313 | 1,576234 | metabolic process |
| ENSG00000165030 | PPP1R2 | 1,12892 | 0,89793 | 2,207018 | metabolic process |
| ENSG00000160949 | PPP2R4 | 0,092616 | 0,030341 | -0,02746 | metabolic process |
| ENSG00000105374 | PPT1 | 0,174459 | 0,441306 | 1,340084 | metabolic process |
| ENSG00000114857 | PPT2 | 0,4127 | 0,649785 | 0,061758 | metabolic process |
| ENSG00000147140 | PRKACA | -0,42229 | -0,41008 | 0,281813 | metabolic process |
| ENSG00000147140 | PRKAR1B | -0,07102 | 0,063163 | -0,58548 | metabolic process |
| ENSG00000147140 | PRKAR2A | -0,14429 | -0,22771 | 0,003649 | metabolic process |
| ENSG00000147140 | PRKAR2B | 1,79859 | 1,576574 | 0,774779 | metabolic process |
| ENSG00000087269 | PRNP | 0,713078 | 0,902652 | 0,975146 | metabolic process |
| ENSG00000115216 | PSAP | 0,795106 | 1,084593 | 2,040301 | metabolic process |
| ENSG00000154146 | PSMA1 | 0,511412 | 0,307757 | 0,075845 | metabolic process |
| ENSG00000171119 | PSMA2 | 0,539221 | 0,590382 | 0,710682 | metabolic process |
| ENSG00000165671 | PSMA6 | 0,188446 | -0,02429 | -0,07836 | metabolic process |
| ENSG00000088833 | PSMA7 | 0,111888 | 0,600691 | 0,916087 | metabolic process |
| ENSG00000117697 | PSMB1 | 0,124262 | -0,34835 | -0,36765 | metabolic process |
| ENSG00000107672 | PSMB10 | -0,65802 | -0,82371 | 0,048595 | metabolic process |
| ENSG00000037474 | PSMB2 | 1,87463 | 1,698844 | 1,47286 | metabolic process |
| ENSG00000130305 | PSMB3 | 1,202671 | 1,410919 | 1,311083 | metabolic process |
| ENSG00000125458 | PSMB4 | 1,850704 | 1,364658 | 1,8872 | metabolic process |
| ENSG00000076685 | PSMB6 | 1,125764 | 1,287579 | 0,717843 | metabolic process |
| ENSG00000168268 | PSMB7 | 1,006554 | 0,885053 | 0,62839 | metabolic process |
| ENSG00000205309 | PSMC2 | 0,54618 | 0,754569 | 0,539639 | metabolic process |
| ENSG00000065320 | PSMC3 | 0,937396 | 0,919445 | 0,428637 | metabolic process |
| ENSG00000196358 | PSMC4 | 0,912199 | 0,982313 | 0,745915 | metabolic process |
| ENSG00000013374 | PSMC5 | 0,397263 | 0,242087 | -0,23687 | metabolic process |
| ENSG00000095906 | PSMD1 | 0,44281 | 0,717374 | 0,525304 | metabolic process |
| ENSG00000104805 | PSMD11 | 0,557114 | 0,632246 | 0,252652 | metabolic process |
| ENSG00000070081 | PSMD3 | 1,03587 | 0,972807 | 0,752389 | metabolic process |
| ENSG00000069275 | PSMD7 | 1,412024 | 1,606718 | 1,649406 | metabolic process |
| ENSG00000090273 | PSMD8 | 1,95467 | 1,976999 | 1,544596 | metabolic process |
| ENSG00000015676 | PSME1 | 0,935881 | 0,9253 | 1,146034 | metabolic process |
| ENSG00000106268 | PSME2 | 1,077808 | 0,306033 | 0,230474 | metabolic process |
| ENSG00000168101 | PSME3 | 0,012481 | 0,146398 | 0,011534 | metabolic process |
| ENSG00000167005 | PSME4 | 0,778421 | 0,064242 | 1,319399 | metabolic process |
| ENSG00000149761 | PSMF1 | 1,470156 | 1,960602 | 2,593608 | metabolic process |
| ENSG00000138750 | PTEN | 1,534163 | 1,737146 | 2,702853 | metabolic process |
| ENSG00000176510 | PUS1 | 0,095367 | -0,07469 | -0,33561 | metabolic process |
| ENSG00000110048 | PYGL | -0,2622 | -0,05357 | 2,804893 | metabolic process |
| ENSG00000134996 | QSOX2 | -0,48809 | 0,042588 | 1,13901 | metabolic process |
| ENSG00000138964 | RAP1A | -1,53732 | -0,61405 | 0,003217 | metabolic process |
| ENSG00000173889 | RFC3 | -0,18372 | 0,108084 | -0,58681 | metabolic process |
| ENSG00000132825 | RPIA | -0,49845 | -0,07209 | 1,406558 | metabolic process |
| ENSG00000174231 | RRM1 | 0,468834 | 1,002793 | 0,266508 | metabolic process |
| ENSG00000174231 | RUVBL2 | 0,826664 | 0,68257 | -0,29899 | metabolic process |
| ENSG00000196415 | SAT1 | 1,611276 | 1,423865 | 3,057043 | metabolic process |
| ENSG00000108294 | SCO2 | -0,11115 | -0,30893 | 0,31764 | metabolic process |
| ENSG00000013275 | SDHA | 2,401545 | 2,374158 | 1,647689 | metabolic process |
| ENSG00000108671 | SEC11A | 0,203948 | 0,13341 | 0,676639 | metabolic process |
| ENSG00000196396 | SEPX1 | -0,07275 | 0,257395 | 2,311221 | metabolic process |
| ENSG00000134242 | SERINC1 | -0,274 | -0,14161 | 1,237418 | metabolic process |
| ENSG00000143851 | SERINC5 | -0,23839 | 0,031611 | 0,192935 | metabolic process |
| ENSG00000081237 | SERP1 | 0,81527 | 0,806091 | 1,400555 | metabolic process |
| ENSG00000169228 | SGMS1 | -1,05828 | -0,51946 | 0,903358 | metabolic process |
| ENSG00000102317 | SLC25A1 | 0,482487 | 0,296475 | 0,120318 | metabolic process |
| ENSG00000184863 | SLC25A11 | 0,653006 | 0,932578 | 0,820157 | metabolic process |
| ENSG00000173933 | SLC25A3 | 3,157035 | 2,983933 | 2,972593 | metabolic process |
| ENSG00000170748 | SLC25A5 | 2,835955 | 2,654373 | 2,491889 | metabolic process |
| ENSG00000139194 | SLC25A6 | 4,233195 | 4,037683 | 3,699853 | metabolic process |
| ENSG00000135870 | SLC2A1 | 0,961291 | 2,512187 | 5,284353 | metabolic process |
| ENSG00000104856 | SLC37A4 | 0,200044 | 0,883384 | 0,377441 | metabolic process |
| ENSG00000177105 | SMARCA5 | 0,38415 | 0,471203 | 0,551736 | metabolic process |
| ENSG00000169397 | SMPD2 | -1,06686 | -0,77199 | 0,593819 | metabolic process |
| ENSG00000225178 | SOD2 | -0,26943 | -0,43738 | 0,91811 | metabolic process |
| ENSG00000228981 | SORD | 0,075728 | -0,62624 | -1,36937 | metabolic process |
| ENSG00000232203 | SPCS1 | 0,840692 | 0,824482 | 0,775611 | metabolic process |
| ENSG00000212664 | SPCS3 | 0,540104 | 0,403458 | 0,67417 | metabolic process |
| ENSG00000219682 | SREBF1 | 0,623009 | 1,164031 | 0,886621 | metabolic process |
| ENSG00000233558 | SREBF2 | -0,53243 | -0,64555 | 0,074191 | metabolic process |
| ENSG00000063177 | ST6GAL1 | -0,61143 | -0,18817 | 0,116545 | metabolic process |
| ENSG00000063177 | STARD3 | 0,621222 | 0,714943 | 0,862334 | metabolic process |
| ENSG00000108107 | STK11 | 0,439918 | 0,748099 | 1,675565 | metabolic process |
| ENSG00000175634 | TALDO1 | 1,982734 | 2,167204 | 2,816433 | metabolic process |
| ENSG00000052749 | TAZ | 0,152584 | -0,11867 | 0,657057 | metabolic process |
| ENSG00000197747 | TBXAS1 | -0,82299 | -1,18595 | 0,579644 | metabolic process |
| ENSG00000136715 | TECR | 2,052165 | 2,278183 | 2,001585 | metabolic process |
| ENSG00000175467 | TGFB1 | -0,06435 | 0,069337 | 1,570909 | metabolic process |
| ENSG00000075856 | TGM2 | 1,24576 | 0,990112 | 1,026158 | metabolic process |
| ENSG00000212352 | TKT | 1,507446 | 1,666293 | 3,339863 | metabolic process |
| ENSG00000237515 | TPI1 | 0,732817 | 0,72026 | 0,667592 | metabolic process |
| ENSG00000108061 | TPP1 | 0,139398 | 0,764877 | 1,560782 | metabolic process |
| ENSG00000108528 | TRPT1 | -0,10115 | -0,33173 | 0,20317 | metabolic process |
| ENSG00000164889 | TXN | -0,06932 | 0,387357 | -0,01184 | metabolic process |
| ENSG00000109062 | TYMP | 0,007595 | 0,017003 | 0,639131 | metabolic process |
| ENSG00000127616 | UAP1 | -0,37727 | -0,6413 | 0,24399 | metabolic process |
| ENSG00000153147 | UAP1L1 | 0,06567 | -0,09613 | 0,36083 | metabolic process |
| ENSG00000212395 | UCK1 | 0,380201 | 0,152034 | 0,471348 | metabolic process |
| ENSG00000207152 | UCK2 | 0,757952 | 0,55225 | 0,313015 | metabolic process |
| ENSG00000207166 | UCKL1 | 0,163493 | -0,36858 | -0,19974 | metabolic process |
| ENSG00000201512 | UFSP1 | -0,02061 | -0,17048 | 0,515476 | metabolic process |
| ENSG00000201067 | UNG | 0,611778 | 0,294293 | -0,91791 | metabolic process |
| ENSG00000201199 | UQCRC2 | 1,232927 | 1,119762 | 0,689704 | metabolic process |
| ENSG00000239002 | UROD | 2,154428 | 2,650178 | 2,886213 | metabolic process |
| ENSG00000238741 | USF1 | 0,328028 | 0,33098 | 0,841793 | metabolic process |
| ENSG00000144028 | USP15 | 1,120084 | 1,52727 | 2,797373 | metabolic process |
| ENSG00000112335 | VAMP2 | -0,1674 | 0,053001 | 1,039189 | metabolic process |
| ENSG00000159140 | VCP | 1,44419 | 1,6991 | 1,847994 | metabolic process |
| ENSG00000129128 | VPS4B | -0,77544 | -0,64368 | 0,069826 | metabolic process |
| ENSG00000072310 | WBSCR22 | 1,117978 | 1,381727 | 1,058082 | metabolic process |
| ENSG00000087087 | WNT10B | -0,28551 | -0,4559 | 0,059043 | metabolic process |
| ENSG00000138385 | WRN | 0,833839 | 0,453612 | 0,861978 | metabolic process |
| ENSG00000106028 | WRNIP1 | 0,114494 | 0,067407 | 0,574313 | metabolic process |
| ENSG00000126561 | YPEL5 | 0,989435 | 1,186596 | 3,042293 | metabolic process |
| ENSG00000213246 | ZDHHC18 | 0,171245 | 0,357895 | 1,605775 | metabolic process |
| ENSG00000199806 | ABCF1 | -4,31484 | -4,4672 | -4,21775 | immune response |
| ENSG00000213726 | ADAM8 | -0,3791 | -0,57777 | 0,670667 | immune response |
| ENSG00000180843 | ADRBK1 | 0,469244 | 0,514096 | 1,669784 | immune response |
| ENSG00000234386 | AGER | -0,26758 | -0,54054 | 0,298349 | immune response |
| ENSG00000100813 | AIF1 | -1,52785 | -1,04022 | 1,092618 | immune response |
| ENSG00000154930 | AKIRIN2 | 0,423453 | 0,243456 | 1,61507 | immune response |
| ENSG00000181786 | AKT1 | 1,744731 | 1,7493 | 1,843017 | immune response |
| ENSG00000243173 | ANXA1 | 1,757694 | 1,972089 | 5,086203 | immune response |
| ENSG00000243212 | AOAH | -2,17787 | -2,12423 | 0,676775 | immune response |
| ENSG00000244591 | AOC3 | -2,57959 | -2,7293 | -2,08134 | immune response |
| ENSG00000116337 | APOBEC3A | -1,0241 | -0,8372 | 1,327151 | immune response |
| ENSG00000089053 | APP | -0,82592 | -0,80844 | 0,718683 | immune response |
| ENSG00000089053 | AQP9 | -2,23794 | -1,8757 | 0,533126 | immune response |
| ENSG00000213337 | ARHGDIB | 1,467802 | 1,340886 | 3,782263 | immune response |
| ENSG00000240583 | ATF2 | 1,052116 | 0,629382 | 0,941958 | immune response |
| ENSG00000143761 | ATOX1 | -0,08206 | -0,26577 | 0,1681 | immune response |
| ENSG00000182196 | AZU1 | 0,236817 | 1,109149 | 2,958423 | immune response |
| ENSG00000144746 | B2M | 1,907089 | 1,9058 | 3,898293 | immune response |
| ENSG00000100325 | BCAP31 | -0,20421 | -0,28482 | 0,320774 | immune response |
| ENSG00000169696 | BCL2L1 | -0,04684 | -0,00566 | 3,185443 | immune response |
| ENSG00000138138 | BCL6 | -2,02711 | -1,43335 | 1,082549 | immune response |
| ENSG00000124172 | BPI | 0,003594 | 1,192553 | 3,668063 | immune response |
| ENSG00000113732 | BTG3 | 0,029902 | -0,66031 | -0,53371 | immune response |
| ENSG00000109743 | C1orf38 | 0,908481 | 0,731734 | 1,847133 | immune response |
| ENSG00000197180 | C1QBP | 1,945156 | 1,77221 | 0,977558 | immune response |
| ENSG00000087302 | C3AR1 | -2,18893 | -1,74095 | 0,495276 | immune response |
| ENSG00000172366 | C5AR1 | -1,02772 | -0,40023 | 1,174554 | immune response |
| ENSG00000130775 | CAPZA1 | 0,616311 | 0,507446 | 1,654698 | immune response |
| ENSG00000143612 | CAPZA2 | -0,14992 | 0,032427 | 0,814163 | immune response |
| ENSG00000143751 | CARD9 | -0,19037 | -0,37764 | 0,141074 | immune response |
| ENSG00000100220 | CCL5 | -1,72907 | -1,78776 | 0,118568 | immune response |
| ENSG00000115649 | CCR7 | -1,42372 | -0,95976 | 0,144359 | immune response |
| ENSG00000173950 | CD164 | 1,515959 | 1,63235 | 2,750373 | immune response |
| ENSG00000197405 | CD46 | 1,073007 | 0,907689 | 1,532239 | immune response |
| ENSG00000120306 | CD48 | -1,78578 | -1,46982 | 0,320446 | immune response |
| ENSG00000240900 | CD7 | 1,244167 | 1,10817 | 1,905948 | immune response |
| ENSG00000112308 | CD74 | 2,334515 | 1,855249 | 3,374823 | immune response |
| ENSG00000146826 | CD97 | 0,208697 | 0,481365 | 2,870933 | immune response |
| ENSG00000127022 | CEACAM8 | 0,080986 | 1,202222 | 3,914003 | immune response |
| ENSG00000127022 | CEBPB | 0,224206 | 0,215422 | 2,296169 | immune response |
| ENSG00000116489 | CHID1 | 0,705235 | 0,676789 | 0,666 | immune response |
| ENSG00000198898 | CHIT1 | -1,50565 | -1,50357 | 0,463734 | immune response |
| ENSG00000121691 | CHST2 | 0,500206 | 0,604689 | 1,658271 | immune response |
| ENSG00000145386 | CLEC4D | -3,02942 | -2,50193 | 0,597676 | immune response |
| ENSG00000026508 | CNPY3 | 1,209044 | 1,557112 | 2,623034 | immune response |
| ENSG00000110848 | COL4A3BP | -0,90944 | -0,89378 | 0,444541 | immune response |
| ENSG00000136807 | CREB1 | 0,284631 | 0,585755 | 1,217715 | immune response |
| ENSG00000129757 | CREBBP | 0,872804 | 1,050262 | 2,530726 | immune response |
| ENSG00000101290 | CRISP3 | 0,076443 | 0,983501 | 4,548583 | immune response |
| ENSG00000138092 | CSF1 | 1,419153 | 0,523943 | -0,32511 | immune response |
| ENSG00000166582 | CSF3 | 0,666419 | 0,301941 | 0,842744 | immune response |
| ENSG00000101639 | CSF3R | -0,98373 | -0,1969 | 2,890133 | immune response |
| ENSG00000163320 | CST3 | 2,721195 | 1,881301 | 2,009755 | immune response |
| ENSG00000167670 | CST7 | 0,912428 | 1,444412 | 2,946703 | immune response |
| ENSG00000169105 | CTSG | -0,58122 | 0,865539 | 2,676653 | immune response |
| ENSG00000175040 | CTSS | -1,57181 | -1,47176 | 1,397607 | immune response |
| ENSG00000155508 | DDIT3 | 0,021351 | -0,49467 | 1,091603 | immune response |
| ENSG00000173786 | DDOST | 1,16228 | 0,912348 | 0,85626 | immune response |
| ENSG00000230673 | DUSP1 | 2,877685 | 2,997073 | 3,321373 | immune response |
| ENSG00000137770 | DUSP6 | -1,54955 | -1,01247 | 1,319974 | immune response |
| ENSG00000150281 | DUSP7 | 0,240088 | 0,337013 | 0,463548 | immune response |
| ENSG00000226574 | ECSIT | 0,351159 | 0,001417 | 0,310775 | immune response |
| ENSG00000165732 | ELK1 | 0,698393 | 0,500423 | 0,632796 | immune response |
| ENSG00000108654 | EP300 | 0,856371 | 0,804801 | 2,852863 | immune response |
| ENSG00000168350 | EPX | -0,55109 | 0,287205 | 3,561953 | immune response |
| ENSG00000198837 | ERCC1 | 0,079642 | 0,030975 | -0,06454 | immune response |
| ENSG00000184014 | ERCC2 | -0,18662 | -0,07666 | 0,218708 | immune response |
| ENSG00000136986 | ERCC3 | 0,278499 | -0,13482 | -0,61082 | immune response |
| ENSG00000116675 | F11R | -0,09648 | -0,19133 | 0,636938 | immune response |
| ENSG00000111361 | FCAR | -0,25829 | 0,146293 | 3,753833 | immune response |
| ENSG00000125977 | FCGRT | 0,850237 | 1,197149 | 1,236109 | immune response |
| ENSG00000110321 | FOS | 1,041568 | 0,989906 | 1,95571 | immune response |
| ENSG00000142227 | FYB | -2,46681 | -2,12534 | 0,773448 | immune response |
| ENSG00000100393 | GAB1 | 0,423881 | 0,832003 | 0,893612 | immune response |
| ENSG00000144488 | GCLC | -0,18691 | 0,344415 | 2,373697 | immune response |
| ENSG00000183114 | GPR44 | -0,05644 | -0,26903 | 0,265301 | immune response |
| ENSG00000143409 | GPX1 | 1,048289 | 1,01409 | 1,663225 | immune response |
| ENSG00000039523 | GPX4 | 2,472475 | 2,080876 | 1,726283 | immune response |
| ENSG00000178974 | GTPBP1 | 1,438922 | 1,722067 | 2,796373 | immune response |
| ENSG00000132004 | GZMM | -0,23524 | -0,51996 | 0,566286 | immune response |
| ENSG00000105701 | HCK | -1,01274 | -0,98611 | 0,876603 | immune response |
| ENSG00000090554 | HDAC4 | -0,5099 | -0,39276 | 0,348999 | immune response |
| ENSG00000059122 | HDAC5 | -0,80719 | -0,79956 | 0,534399 | immune response |
| ENSG00000143458 | HLA-B | 3,745695 | 3,508548 | 5,204923 | immune response |
| ENSG00000116717 | HLA-C | 3,096715 | 3,190833 | 4,661603 | immune response |
| ENSG00000099860 | HLA-DMA | 0,555582 | -0,13835 | 0,563832 | immune response |
| ENSG00000130222 | HLA-DPA1 | 0,042968 | -0,20432 | 1,256505 | immune response |
| ENSG00000179271 | HLA-DPB1 | 0,206148 | -0,47622 | 1,058551 | immune response |
| ENSG00000178950 | HLA-DRA | 0,144781 | -0,30124 | 1,21051 | immune response |
| ENSG00000054983 | HLA-E | 2,068598 | 1,857098 | 3,992233 | immune response |
| ENSG00000141012 | HLA-F | -0,12979 | -0,22499 | 1,882191 | immune response |
| ENSG00000175857 | HMGB1 | 0,325895 | 0,184994 | 0,305622 | immune response |
| ENSG00000111780 | HMOX2 | -0,3745 | -0,68397 | 0,079601 | immune response |
| ENSG00000107862 | HNRNPA0 | 0,607202 | 0,488469 | 0,683604 | immune response |
| ENSG00000162676 | HP | -2,31322 | -1,60079 | 2,044272 | immune response |
| ENSG00000087338 | HSP90AB1 | 2,070254 | 2,237733 | 0,857948 | immune response |
| ENSG00000068394 | IFI6 | -0,3496 | -0,44692 | 0,347403 | immune response |
| ENSG00000125734 | IFITM1 | -0,26667 | -0,30007 | 1,275023 | immune response |
| ENSG00000183484 | IFITM2 | 0,747117 | 1,106389 | 2,632797 | immune response |
| ENSG00000158292 | IGF1R | 0,31585 | 0,358973 | 1,46631 | immune response |
| ENSG00000183134 | IGFBP4 | 0,470154 | 0,134242 | 0,767319 | immune response |
| ENSG00000182885 | IGLL1 | 0,348141 | 0,057194 | 0,738313 | immune response |
| ENSG00000132522 | IGSF6 | -1,23381 | -0,8264 | 1,235488 | immune response |
| ENSG00000233276 | IL16 | -1,41289 | -1,48065 | 0,038229 | immune response |
| ENSG00000167468 | IL17C | -0,1045 | -0,33275 | 0,195349 | immune response |
| ENSG00000089351 | IL17D | 0,291432 | 0,076032 | 0,504263 | immune response |
| ENSG00000116032 | IL18RAP | -3,06464 | -2,43451 | 1,112563 | immune response |
| ENSG00000178719 | IL1R2 | -1,89527 | -1,53606 | 0,218937 | immune response |
| ENSG00000197245 | IL4R | -0,9388 | -0,98937 | 0,920008 | immune response |
| ENSG00000228963 | IL8 | 0,47711 | 0,622657 | 3,389943 | immune response |
| ENSG00000063515 | ILF2 | 2,21086 | 2,715543 | 1,760386 | immune response |
| ENSG00000184674 | INHBB | 0,055211 | -0,19449 | 0,159783 | immune response |
| ENSG00000143774 | IPCEF1 | -1,65572 | -1,59757 | 0,778473 | immune response |
| ENSG00000170180 | IRAK1 | 0,255255 | 0,605751 | 0,614264 | immune response |
| ENSG00000187166 | IRF3 | 0,341893 | 0,362983 | 0,891721 | immune response |
| ENSG00000184897 | IRF7 | -0,02477 | -0,23625 | 0,708715 | immune response |
| ENSG00000105968 | IRF8 | -1,70504 | -1,50285 | 0,057895 | immune response |
| ENSG00000188375 | ITCH | 0,327569 | -0,15833 | 0,42053 | immune response |
| ENSG00000103253 | ITGAL | -1,8003 | -1,8497 | 0,332699 | immune response |
| ENSG00000164818 | JAK2 | -1,06168 | -0,16865 | 0,033743 | immune response |
| ENSG00000144485 | JUN | 0,378418 | 0,385137 | 1,170919 | immune response |
| ENSG00000064393 | KCNN4 | -0,15229 | 0,100442 | 0,116121 | immune response |
| ENSG00000124693 | KDM6B | -0,3967 | -0,30987 | 0,938318 | immune response |
| ENSG00000107521 | LAT | -0,0739 | -0,63458 | 0,435872 | immune response |
| ENSG00000199090 | LCN2 | 1,45788 | 2,190947 | 4,837443 | immune response |
| ENSG00000207809 | LCP2 | 0,179162 | 0,477054 | 2,668963 | immune response |
| ENSG00000096384 | LGALS3 | -0,85702 | -0,32128 | 1,37772 | immune response |
| ENSG00000096384 | LIAS | 0,976847 | 0,554549 | 1,137283 | immune response |
| ENSG00000166598 | LILRA3 | -1,32148 | -1,00794 | 2,200434 | immune response |
| ENSG00000132622 | LILRA5 | -1,84357 | -1,43244 | 1,431129 | immune response |
| ENSG00000163464 | LSP1 | 0,099341 | 0,415317 | 1,979967 | immune response |
| ENSG00000143621 | LST1 | 0,702751 | 0,4892 | 2,768503 | immune response |
| ENSG00000143621 | LTA4H | 0,106109 | 0,552946 | 2,266382 | immune response |
| ENSG00000143621 | LTB | 0,695732 | 0,570841 | 1,366748 | immune response |
| ENSG00000143621 | LTB4R | -0,88738 | -0,88366 | 0,003994 | immune response |
| ENSG00000166333 | LYN | -0,33403 | -0,36232 | 1,632926 | immune response |
| ENSG00000106348 | LYZ | 2,941125 | 3,793763 | 5,689053 | immune response |
| ENSG00000114933 | MADCAM1 | 0,732354 | 0,254938 | 0,866939 | immune response |
| ENSG00000198700 | MAP2K1 | 0,256413 | 0,11135 | 0,759268 | immune response |
| ENSG00000106012 | MAP2K2 | 1,798829 | 1,738887 | 1,588758 | immune response |
| ENSG00000140575 | MAP2K3 | 0,041426 | 0,865917 | 3,080833 | immune response |
| ENSG00000145703 | MAP3K1 | -0,30771 | -0,31125 | 0,397946 | immune response |
| ENSG00000090376 | MAP3K5 | -0,10312 | 0,03711 | 0,040707 | immune response |
| ENSG00000185507 | MAPK1 | 0,453091 | 1,116785 | 2,384759 | immune response |
| ENSG00000140968 | MAPK14 | -0,34239 | -0,34944 | 0,8961 | immune response |
| ENSG00000185950 | MAPK3 | -0,15226 | -0,02108 | 1,102017 | immune response |
| ENSG00000136003 | MAPKAPK2 | 0,110108 | -0,088 | 1,366485 | immune response |
| ENSG00000172183 | MAPKAPK3 | 0,373107 | 0,406058 | 1,493582 | immune response |
| ENSG00000169896 | MARK2 | -0,39949 | -0,09403 | 0,670777 | immune response |
| ENSG00000148841 | MAVS | 0,180958 | 0,053162 | 0,373348 | immune response |
| ENSG00000065427 | MEF2A | -0,32384 | -0,4377 | 0,380704 | immune response |
| ENSG00000173120 | MICB | -0,47734 | -0,54124 | -0,07289 | immune response |
| ENSG00000121774 | MLF2 | 1,120822 | 1,037276 | 1,904355 | immune response |
| ENSG00000081791 | MMP25 | -0,48274 | -0,1915 | 1,020694 | immune response |
| ENSG00000131149 | MMP9 | 0,619537 | 1,557727 | 4,460793 | immune response |
| ENSG00000109265 | MPO | -0,1946 | 1,540662 | 3,079153 | immune response |
| ENSG00000111057 | MSRA | -0,93298 | -0,98974 | 0,405146 | immune response |
| ENSG00000205869 | MTF1 | -0,86848 | -0,54493 | 1,056934 | immune response |
| ENSG00000172155 | MYD88 | 0,191777 | 0,35892 | 1,574111 | immune response |
| ENSG00000121897 | NAPRT1 | 0,909431 | 1,007356 | 1,867097 | immune response |
| ENSG00000077454 | NDST1 | -0,80106 | -0,84075 | 0,524487 | immune response |
| ENSG00000198799 | NDUFA12 | 0,584111 | 0,340766 | 0,228029 | immune response |
| ENSG00000130881 | NDUFA6 | 0,724493 | 0,993385 | 0,896746 | immune response |
| ENSG00000132128 | NDUFS2 | 0,40684 | 0,203542 | 0,654889 | immune response |
| ENSG00000108829 | NDUFS8 | 0,914866 | 0,68611 | 0,791149 | immune response |
| ENSG00000183011 | NFAM1 | 0,052239 | -0,00161 | 1,258151 | immune response |
| ENSG00000227507 | NFATC3 | 0,343071 | 0,608223 | 0,832574 | immune response |
| ENSG00000007392 | NFIL3 | -2,16589 | -1,8973 | 1,279949 | immune response |
| ENSG00000108848 | NFKB1 | -0,33621 | -0,73829 | 0,464338 | immune response |
| ENSG00000104903 | NFKB2 | -0,87946 | -0,77183 | 0,523592 | immune response |
| ENSG00000147507 | NFKBIA | 2,952725 | 2,516024 | 3,730603 | immune response |
| ENSG00000011009 | NFKBIB | 0,312933 | 0,239054 | 0,625249 | immune response |
| ENSG00000143669 | NFKBID | -0,68147 | -0,7114 | 1,003532 | immune response |
| ENSG00000003056 | NFKBIZ | -0,85832 | -1,20449 | 0,472877 | immune response |
| ENSG00000002822 | NFX1 | -0,74402 | -0,42226 | 0,207261 | immune response |
| ENSG00000104774 | NLRC5 | -0,25334 | -0,30956 | 1,155643 | immune response |
| ENSG00000100030 | NOTCH1 | -1,10204 | -1,10389 | 0,107911 | immune response |
| ENSG00000076003 | NUDT1 | 0,071204 | -0,29878 | 0,299521 | immune response |
| ENSG00000165792 | OLR1 | -2,41673 | -2,06071 | 1,037174 | immune response |
| ENSG00000151690 | ORM1 | -2,67334 | -2,15477 | 0,256297 | immune response |
| ENSG00000214922 | OTUD5 | -0,22233 | -0,0223 | 0,925303 | immune response |
| ENSG00000198160 | OXSR1 | -0,18961 | 0,109531 | 0,654434 | immune response |
| ENSG00000100985 | PARK7 | -1,20596 | -1,37703 | -1,91069 | immune response |
| ENSG00000130675 | PARP4 | 0,69475 | 0,302467 | 1,069393 | immune response |
| ENSG00000123562 | PCBP2 | 3,194945 | 2,832173 | 3,386823 | immune response |
| ENSG00000048544 | PEBP1 | 1,762122 | 1,316999 | 0,428471 | immune response |
| ENSG00000181991 | PELI1 | 0,201273 | -0,89426 | 1,126829 | immune response |
| ENSG00000147065 | PGLYRP1 | -0,02348 | 0,426014 | 2,910373 | immune response |
| ENSG00000214114 | PIK3C3 | -0,56003 | -0,38169 | 0,180755 | immune response |
| ENSG00000172936 | PIK3CD | -1,04216 | -0,88229 | 0,502601 | immune response |
| ENSG00000133026 | PIK3CG | -1,03197 | -0,7522 | 2,046126 | immune response |
| ENSG00000092841 | PIK3R4 | -0,12048 | 0,21886 | -0,11237 | immune response |
| ENSG00000099331 | PIN1 | 0,641048 | 0,509241 | 0,673606 | immune response |
| ENSG00000111300 | PLAA | -0,21075 | 0,047421 | 0,513302 | immune response |
| ENSG00000158747 | PNKP | 0,881525 | 0,849257 | 1,229043 | immune response |
| ENSG00000131495 | POLR3E | 1,114619 | 0,773546 | 0,517669 | immune response |
| ENSG00000170906 | POLR3H | -0,25545 | 0,053269 | -0,12395 | immune response |
| ENSG00000184983 | POLR3K | 0,934116 | 1,055079 | 0,939934 | immune response |
| ENSG00000116044 | PPP1R15B | 0,940046 | 1,217382 | 2,933373 | immune response |
| ENSG00000136448 | PRDX2 | -0,30753 | 0,202068 | 0,428842 | immune response |
| ENSG00000112992 | PRDX3 | 1,01917 | 1,536892 | 0,648504 | immune response |
| ENSG00000141101 | PRDX5 | -0,19594 | 0,150457 | 0,989923 | immune response |
| ENSG00000188976 | PRDX6 | 0,287243 | 0,920539 | 1,548393 | immune response |
| ENSG00000147140 | PRG2 | 0,641698 | 1,155716 | 4,059823 | immune response |
| ENSG00000147140 | PRG3 | -1,5574 | -0,94377 | 1,986877 | immune response |
| ENSG00000147140 | PRKCSH | 2,588185 | 2,531373 | 2,427929 | immune response |
| ENSG00000087269 | PRNP | 0,713078 | 0,902652 | 0,975146 | immune response |
| ENSG00000111641 | PROK2 | -0,45496 | -0,31675 | 1,875448 | immune response |
| ENSG00000124789 | PSTPIP1 | -1,0661 | -1,00584 | 0,589797 | immune response |
| ENSG00000110713 | PTGER4 | -0,84297 | -0,70349 | 0,9833 | immune response |
| ENSG00000137804 | PTGS2 | -2,3826 | -2,07263 | 0,15783 | immune response |
| ENSG00000177989 | PTPRCAP | -0,43858 | -0,4616 | 0,826764 | immune response |
| ENSG00000147162 | PTX3 | -2,44144 | -2,64993 | 0,746934 | immune response |
| ENSG00000182500 | PXK | -0,5312 | -0,27684 | 1,258485 | immune response |
| ENSG00000085840 | PYCARD | -0,21847 | 0,024743 | 1,930272 | immune response |
| ENSG00000142657 | RELA | 0,519434 | 0,336723 | 0,898974 | immune response |
| ENSG00000172943 | RGS14 | -0,03185 | -0,28116 | 0,685156 | immune response |
| ENSG00000141506 | RIPK2 | 0,679384 | 0,450826 | 0,974048 | immune response |
| ENSG00000175325 | RPS6KA1 | 0,826289 | 1,362559 | 1,915614 | immune response |
| ENSG00000110107 | RPS6KA3 | -0,42137 | -0,13962 | 0,18504 | immune response |
| ENSG00000174231 | RXRA | 0,056242 | 0,639451 | 1,75434 | immune response |
| ENSG00000174231 | S100A12 | 1,634002 | 1,877168 | 4,603923 | immune response |
| ENSG00000174231 | S100A8 | 3,906835 | 4,004153 | 5,793893 | immune response |
| ENSG00000174231 | S100A9 | 2,286125 | 2,513213 | 5,100173 | immune response |
| ENSG00000174231 | S1PR4 | -0,39995 | -0,15756 | 0,868218 | immune response |
| ENSG00000205155 | SBNO2 | -0,2047 | -0,14081 | 0,720004 | immune response |
| ENSG00000125384 | SEMA4D | -1,4626 | -1,17799 | -0,67523 | immune response |
| ENSG00000171522 | SEMA7A | -1,043 | -0,21028 | 1,3985 | immune response |
| ENSG00000213402 | SERPINA1 | -0,47845 | -0,19396 | 2,0912 | immune response |
| ENSG00000169228 | SGMS1 | -1,05828 | -0,51946 | 0,903358 | immune response |
| ENSG00000172613 | SIGIRR | -0,11158 | -0,49576 | 0,27713 | immune response |
| ENSG00000116473 | SIRT1 | -0,41654 | -0,40145 | 0,127446 | immune response |
| ENSG00000023287 | SLC11A1 | -1,02402 | -0,82734 | 0,740286 | immune response |
| ENSG00000235244 | SPN | 1,443186 | 1,370412 | 0,861922 | immune response |
| ENSG00000185290 | SRPK1 | 1,187043 | 0,926124 | 1,748019 | immune response |
| ENSG00000108107 | STK25 | 1,86226 | 1,700689 | 0,688308 | immune response |
| ENSG00000138326 | SYK | 0,24391 | -0,05709 | 1,227964 | immune response |
| ENSG00000144580 | TAP1 | -0,24265 | -0,29358 | 1,144412 | immune response |
| ENSG00000160214 | TAX1BP1 | -0,34622 | -0,30181 | 1,051889 | immune response |
| ENSG00000183207 | TBK1 | -0,51179 | -0,70316 | 0,065558 | immune response |
| ENSG00000163220 | TCF12 | 1,411817 | 1,494712 | 1,548125 | immune response |
| ENSG00000142230 | TCF7 | -0,95762 | -1,04753 | 0,025032 | immune response |
| ENSG00000175467 | TGFB1 | -0,06435 | 0,069337 | 1,570909 | immune response |
| ENSG00000075856 | THBS1 | -2,0025 | -1,02608 | 0,866713 | immune response |
| ENSG00000126524 | TIAL1 | -0,06491 | -0,50759 | 0,274613 | immune response |
| ENSG00000168385 | TMEM173 | -0,12359 | -0,45058 | 0,188147 | immune response |
| ENSG00000183431 | TNFAIP3 | -0,53885 | -0,48525 | 1,760881 | immune response |
| ENSG00000143368 | TNFRSF14 | -0,6107 | -0,26589 | 0,556472 | immune response |
| ENSG00000198089 | TNFRSF1A | -0,41858 | -0,41417 | 0,322465 | immune response |
| ENSG00000116754 | TNFRSF4 | -0,11847 | -0,26804 | 0,32678 | immune response |
| ENSG00000064607 | TNFSF8 | -1,64945 | -1,50688 | 0,39284 | immune response |
| ENSG00000156304 | TNIP1 | -0,0454 | -0,15175 | 0,676165 | immune response |
| ENSG00000111786 | TOLLIP | 0,176162 | 0,246348 | 0,790199 | immune response |
| ENSG00000182199 | TPM4 | -0,61123 | -0,50855 | 0,138222 | immune response |
| ENSG00000105738 | TRAF2 | -0,18352 | -0,10462 | 0,134143 | immune response |
| ENSG00000198053 | TRAF6 | 0,005267 | -0,52772 | 0,376704 | immune response |
| ENSG00000018280 | TRIM25 | 0,858363 | 0,375795 | 0,605507 | immune response |
| ENSG00000164889 | TXN | -0,06932 | 0,387357 | -0,01184 | immune response |
| ENSG00000114923 | TXN2 | 0,265646 | 0,179162 | -0,43704 | immune response |
| ENSG00000130821 | TXNIP | 3,471265 | 3,051753 | 3,753223 | immune response |
| ENSG00000139613 | UBA52 | 2,730905 | 2,673263 | 2,979033 | immune response |
| ENSG00000066117 | UBA7 | 0,142883 | -0,35918 | -0,28604 | immune response |
| ENSG00000198887 | UBB | 4,588115 | 4,657338 | 5,213323 | immune response |
| ENSG00000196787 | UBC | 5,120515 | 4,831483 | 5,873363 | immune response |
| ENSG00000135587 | UBE2L6 | 0,878624 | 0,972105 | 1,005947 | immune response |
| ENSG00000102172 | UBE2N | 0,416016 | 0,015038 | 0,579026 | immune response |
| ENSG00000207088 | UCN | 0,187383 | -0,03547 | 0,31481 | immune response |
| ENSG00000206928 | UNC93B1 | -0,20818 | -0,40492 | 1,068301 | immune response |
| ENSG00000130340 | VAV1 | -1,14382 | -0,91728 | 0,384009 | immune response |
| ENSG00000110693 | VNN1 | -2,57556 | -2,0549 | 0,394496 | immune response |
| ENSG00000115306 | WAS | -0,40565 | -0,29186 | 1,589188 | immune response |
| ENSG00000138385 | WRN | 0,833839 | 0,453612 | 0,861978 | immune response |
| ENSG00000197558 | XBP1 | 1,32245 | 1,161278 | 1,378079 | immune response |
| ENSG00000200649 | AAAS | 0,514471 | 0,751675 | 0,341378 | DNA/RNA processing |
| ENSG00000201369 | AARS | -0,23556 | 0,353308 | -0,26459 | DNA/RNA processing |
| ENSG00000199806 | ABCF1 | -4,31484 | -4,4672 | -4,21775 | DNA/RNA processing |
| ENSG00000241883 | ACHE | -0,74433 | -0,4186 | 0,234686 | DNA/RNA processing |
| ENSG00000187728 | ADAD2 | 0,137258 | 0,072532 | 0,562227 | DNA/RNA processing |
| ENSG00000244605 | ADAR | 0,861461 | 0,711913 | 1,104463 | DNA/RNA processing |
| ENSG00000236296 | AFF2 | -1,52519 | -1,74949 | 1,217542 | DNA/RNA processing |
| ENSG00000185294 | AGFG1 | 1,806102 | 1,894934 | 2,983283 | DNA/RNA processing |
| ENSG00000114331 | AHCYL1 | 0,562929 | 0,05574 | -0,17555 | DNA/RNA processing |
| ENSG00000100412 | AIMP2 | 0,385726 | 0,541809 | -0,20368 | DNA/RNA processing |
| ENSG00000181786 | AKT1 | 1,744731 | 1,7493 | 1,843017 | DNA/RNA processing |
| ENSG00000130402 | AKT2 | 0,845838 | 1,114623 | 1,466699 | DNA/RNA processing |
| ENSG00000241802 | ANKRD54 | 0,027689 | 0,131285 | 0,135697 | DNA/RNA processing |
| ENSG00000243451 | ANP32A | 1,507006 | 1,398521 | 1,677815 | DNA/RNA processing |
| ENSG00000104899 | APEX1 | 1,660278 | 1,881638 | 1,026232 | DNA/RNA processing |
| ENSG00000089053 | APP | -0,82592 | -0,80844 | 0,718683 | DNA/RNA processing |
| ENSG00000089053 | AQR | 0,200899 | -0,01739 | -0,6404 | DNA/RNA processing |
| ENSG00000011426 | ARID1A | 1,580174 | 1,500081 | 1,926967 | DNA/RNA processing |
| ENSG00000160746 | ARID1B | 0,783826 | 0,860596 | 0,835328 | DNA/RNA processing |
| ENSG00000140350 | ARID2 | 0,309959 | 0,035915 | 0,449949 | DNA/RNA processing |
| ENSG00000122359 | ARL2 | 1,685634 | 1,665557 | 0,532962 | DNA/RNA processing |
| ENSG00000138279 | ARL6IP4 | 1,586532 | 1,394169 | 1,506808 | DNA/RNA processing |
| ENSG00000131471 | ARL8A | 0,907524 | 0,82394 | 2,372664 | DNA/RNA processing |
| ENSG00000182351 | ARL8B | 0,096891 | 0,009751 | 0,718073 | DNA/RNA processing |
| ENSG00000239936 | ARNT | 0,177068 | 0,836678 | 0,76152 | DNA/RNA processing |
| ENSG00000100823 | ASF1B | -0,20339 | 0,216339 | 0,633563 | DNA/RNA processing |
| ENSG00000084234 | ASH1L | 0,095877 | -0,12064 | 0,443716 | DNA/RNA processing |
| ENSG00000163382 | ASH2L | 1,199837 | 1,306302 | 1,561478 | DNA/RNA processing |
| ENSG00000243811 | ASXL1 | 0,24124 | 0,304421 | 0,650305 | DNA/RNA processing |
| ENSG00000078061 | ATF7IP | 1,206908 | 1,18613 | 1,773153 | DNA/RNA processing |
| ENSG00000117713 | ATXN2 | 0,067204 | 0,523497 | 0,317282 | DNA/RNA processing |
| ENSG00000213465 | AXIN1 | -0,2756 | -0,5623 | 0,152226 | DNA/RNA processing |
| ENSG00000128989 | BANP | -0,56474 | -0,6331 | 0,081038 | DNA/RNA processing |
| ENSG00000137486 | BAP1 | 0,836435 | 1,078119 | 1,187603 | DNA/RNA processing |
| ENSG00000173409 | BAX | 0,734194 | 0,704375 | 0,974735 | DNA/RNA processing |
| ENSG00000004848 | BAZ1A | 0,42706 | 1,098095 | 1,191644 | DNA/RNA processing |
| ENSG00000104763 | BAZ1B | 0,521194 | 0,477889 | 0,31946 | DNA/RNA processing |
| ENSG00000153317 | BAZ2A | 0,972388 | 0,783929 | 2,243178 | DNA/RNA processing |
| ENSG00000118217 | BCOR | -0,15029 | -0,04382 | 0,025637 | DNA/RNA processing |
| ENSG00000165629 | BOP1 | 0,458613 | 0,908028 | 0,438028 | DNA/RNA processing |
| ENSG00000180389 | BPTF | -0,10061 | 0,019394 | 0,630818 | DNA/RNA processing |
| ENSG00000135390 | BRD1 | 0,003298 | -0,17743 | 0,263144 | DNA/RNA processing |
| ENSG00000241468 | BRD4 | 0,906906 | 0,97307 | 1,55134 | DNA/RNA processing |
| ENSG00000167283 | BRE | 0,04066 | -0,05278 | 0,07321 | DNA/RNA processing |
| ENSG00000241837 | BRF1 | -0,10531 | -0,22036 | 0,072082 | DNA/RNA processing |
| ENSG00000117410 | BRPF1 | 0,206689 | 0,096293 | 0,513361 | DNA/RNA processing |
| ENSG00000117410 | BRPF3 | -0,4595 | -0,49036 | 0,096336 | DNA/RNA processing |
| ENSG00000113732 | BTG2 | 0,108047 | 0,416328 | 2,315779 | DNA/RNA processing |
| ENSG00000131100 | BZW2 | 0,120605 | 0,063527 | -0,97208 | DNA/RNA processing |
| ENSG00000204469 | C15orf42 | -0,26337 | 0,112428 | -0,5515 | DNA/RNA processing |
| ENSG00000198604 | C16orf42 | -0,13025 | -0,12606 | 0,230941 | DNA/RNA processing |
| ENSG00000069399 | C17orf49 | 1,196389 | 1,092255 | 1,337602 | DNA/RNA processing |
| ENSG00000186716 | C17orf70 | -0,01809 | -0,01072 | 0,158108 | DNA/RNA processing |
| ENSG00000117475 | C19orf29 | 0,076777 | 0,214765 | 0,298855 | DNA/RNA processing |
| ENSG00000110696 | C20orf20 | -0,13133 | -0,3353 | 0,167553 | DNA/RNA processing |
| ENSG00000089916 | C2orf29 | 0,414198 | 0,467798 | 0,208522 | DNA/RNA processing |
| ENSG00000176912 | C9orf102 | -1,9062 | -1,73221 | -1,42018 | DNA/RNA processing |
| ENSG00000105700 | CABIN1 | 0,889934 | 1,152878 | 0,453742 | DNA/RNA processing |
| ENSG00000143443 | CARHSP1 | 0,000795 | 0,363354 | 0,881751 | DNA/RNA processing |
| ENSG00000162819 | CARM1 | 1,180119 | 1,43784 | 1,941211 | DNA/RNA processing |
| ENSG00000117616 | CARS2 | -0,09483 | 0,086152 | 0,082541 | DNA/RNA processing |
| ENSG00000160679 | CASC3 | 0,734485 | 0,498638 | 1,124382 | DNA/RNA processing |
| ENSG00000162585 | CASC5 | -0,49207 | -0,17928 | 0,174972 | DNA/RNA processing |
| ENSG00000149609 | CBX3 | -1,0896 | -1,46634 | -1,10095 | DNA/RNA processing |
| ENSG00000174407 | CBX4 | 0,317664 | 0,077387 | 1,390457 | DNA/RNA processing |
| ENSG00000177410 | CCAR1 | 2,308055 | 2,078237 | 1,506589 | DNA/RNA processing |
| ENSG00000101189 | CCDC101 | 0,536259 | 0,30481 | -0,1817 | DNA/RNA processing |
| ENSG00000168014 | CCNE1 | -0,5174 | 0,437797 | -0,26777 | DNA/RNA processing |
| ENSG00000115649 | CCNL1 | 1,775192 | 1,3444 | 2,494528 | DNA/RNA processing |
| ENSG00000115649 | CCNL2 | 0,928479 | 0,736019 | 1,206682 | DNA/RNA processing |
| ENSG00000115649 | CCNT1 | 0,836382 | 0,391109 | 0,684281 | DNA/RNA processing |
| ENSG00000115649 | CCNT2 | 0,32514 | -0,04134 | 0,542711 | DNA/RNA processing |
| ENSG00000144647 | CD2BP2 | -0,31155 | -0,55432 | 0,107517 | DNA/RNA processing |
| ENSG00000137038 | CDC34 | 0,438722 | 0,795453 | 2,33751 | DNA/RNA processing |
| ENSG00000148362 | CDC40 | -0,33267 | -0,3186 | 0,306402 | DNA/RNA processing |
| ENSG00000165233 | CDC42SE2 | 1,076142 | 0,343671 | 1,164027 | DNA/RNA processing |
| ENSG00000104267 | CDC5L | 0,588667 | 0,464377 | 0,266589 | DNA/RNA processing |
| ENSG00000142408 | CDCA8 | 0,072781 | 0,213654 | 0,207552 | DNA/RNA processing |
| ENSG00000136436 | CDK2 | 0,31564 | 0,90757 | 0,262399 | DNA/RNA processing |
| ENSG00000138172 | CDK2AP1 | 2,387445 | 1,856849 | 1,588838 | DNA/RNA processing |
| ENSG00000130643 | CDK5RAP2 | 0,161564 | 0,247605 | 0,023911 | DNA/RNA processing |
| ENSG00000110931 | CDK9 | 1,054163 | 0,974569 | 2,007733 | DNA/RNA processing |
| ENSG00000127022 | CDKN2D | -0,07326 | 0,164599 | 1,268876 | DNA/RNA processing |
| ENSG00000127022 | CDT1 | 0,521919 | 0,990134 | 0,275687 | DNA/RNA processing |
| ENSG00000127022 | CENPF | 0,675305 | 1,061737 | 0,850759 | DNA/RNA processing |
| ENSG00000127022 | CENPO | -0,36805 | 0,140569 | -0,14177 | DNA/RNA processing |
| ENSG00000126247 | CHAF1A | 1,039531 | 1,39772 | 0,842912 | DNA/RNA processing |
| ENSG00000126247 | CHD1 | 0,609775 | -0,18188 | 0,587808 | DNA/RNA processing |
| ENSG00000126247 | CHD3 | 0,353347 | 0,034905 | 0,415159 | DNA/RNA processing |
| ENSG00000126247 | CHD4 | 1,166263 | 0,950431 | 1,244349 | DNA/RNA processing |
| ENSG00000126247 | CHD9 | 0,190418 | -0,15372 | 0,519193 | DNA/RNA processing |
| ENSG00000126247 | CHEK1 | -0,28637 | 0,122963 | -0,49661 | DNA/RNA processing |
| ENSG00000126247 | CHERP | 1,226505 | 1,274568 | 0,924309 | DNA/RNA processing |
| ENSG00000187796 | CHMP1A | 0,445823 | 0,501365 | 1,044277 | DNA/RNA processing |
| ENSG00000108349 | CHRAC1 | 0,081561 | -0,17249 | 0,218061 | DNA/RNA processing |
| ENSG00000105879 | CHTF18 | 0,416869 | 0,793516 | 0,819414 | DNA/RNA processing |
| ENSG00000108468 | CHTF8 | 0,620224 | 0,666312 | 0,343511 | DNA/RNA processing |
| ENSG00000122565 | CIAO1 | 0,609339 | 0,498733 | 0,29948 | DNA/RNA processing |
| ENSG00000141582 | CIRBP | 1,488127 | 1,434811 | 1,664491 | DNA/RNA processing |
| ENSG00000100147 | CKAP5 | 0,735059 | 0,730227 | 0,354834 | DNA/RNA processing |
| ENSG00000198087 | CNOT1 | 1,094661 | 1,608164 | 1,839353 | DNA/RNA processing |
| ENSG00000169217 | CNOT2 | 0,881485 | 0,555025 | 0,843531 | DNA/RNA processing |
| ENSG00000167775 | CNOT3 | 0,209735 | 0,314445 | 0,574541 | DNA/RNA processing |
| ENSG00000105383 | CNOT4 | -0,62537 | -0,71215 | 0,032188 | DNA/RNA processing |
| ENSG00000135218 | CNOT8 | 0,375602 | 0,288678 | 0,690767 | DNA/RNA processing |
| ENSG00000104894 | CNP | 0,843693 | 0,852792 | 0,867374 | DNA/RNA processing |
| ENSG00000117091 | COBRA1 | 1,383562 | 1,438074 | 1,03838 | DNA/RNA processing |
| ENSG00000103502 | CPSF1 | 1,057825 | 1,107358 | 0,784068 | DNA/RNA processing |
| ENSG00000123374 | CPSF4 | 0,38575 | 0,312299 | 0,051394 | DNA/RNA processing |
| ENSG00000111328 | CPSF6 | 0,805744 | 0,57572 | 1,005766 | DNA/RNA processing |
| ENSG00000167797 | CPSF7 | -0,23021 | 0,013927 | 0,443758 | DNA/RNA processing |
| ENSG00000129757 | CREBBP | 0,872804 | 1,050262 | 2,530726 | DNA/RNA processing |
| ENSG00000221869 | CRY2 | -0,38586 | -0,46954 | 0,220293 | DNA/RNA processing |
| ENSG00000172757 | CSNK1D | 1,708628 | 1,451727 | 2,009397 | DNA/RNA processing |
| ENSG00000172757 | CSNK1E | 0,365984 | -0,16636 | 0,052773 | DNA/RNA processing |
| ENSG00000111642 | CTCF | 0,34323 | 0,783693 | 0,732781 | DNA/RNA processing |
| ENSG00000085872 | CTDP1 | 0,387707 | 0,281251 | 1,211592 | DNA/RNA processing |
| ENSG00000122966 | CUL4A | 0,210534 | 0,156687 | 0,868145 | DNA/RNA processing |
| ENSG00000232553 | DARS | 0,530385 | 0,756522 | 0,000845 | DNA/RNA processing |
| ENSG00000070371 | DCP1A | 1,288421 | 0,787457 | 1,227768 | DNA/RNA processing |
| ENSG00000111726 | DCP2 | -0,43493 | -0,15972 | 0,405286 | DNA/RNA processing |
| ENSG00000111596 | DDB1 | 1,383941 | 1,299063 | 1,871604 | DNA/RNA processing |
| ENSG00000155508 | DDIT3 | 0,021351 | -0,49467 | 1,091603 | DNA/RNA processing |
| ENSG00000068120 | DDX1 | 0,449262 | 0,429286 | -0,58019 | DNA/RNA processing |
| ENSG00000188986 | DDX17 | 2,100121 | 2,228276 | 2,647933 | DNA/RNA processing |
| ENSG00000166685 | DDX19B | 0,658916 | 0,039647 | -0,18805 | DNA/RNA processing |
| ENSG00000121058 | DDX23 | 0,261582 | 0,642295 | 0,248281 | DNA/RNA processing |
| ENSG00000084636 | DDX24 | 1,228111 | 1,021327 | 0,706861 | DNA/RNA processing |
| ENSG00000080573 | DDX41 | 0,970734 | 1,530957 | 0,710129 | DNA/RNA processing |
| ENSG00000049089 | DDX47 | 0,425636 | 0,109541 | -0,18362 | DNA/RNA processing |
| ENSG00000092758 | DDX5 | 3,311945 | 3,450608 | 4,125823 | DNA/RNA processing |
| ENSG00000173163 | DDX54 | 0,891984 | 1,293368 | 0,889953 | DNA/RNA processing |
| ENSG00000140365 | DDX56 | 0,230899 | 0,646035 | 1,0589 | DNA/RNA processing |
| ENSG00000188243 | DDX6 | 1,84942 | 1,492268 | 2,192613 | DNA/RNA processing |
| ENSG00000122218 | DEDD2 | 0,699057 | 0,968203 | 2,331395 | DNA/RNA processing |
| ENSG00000168090 | DEK | 1,60639 | 1,772135 | 1,525624 | DNA/RNA processing |
| ENSG00000135940 | DGCR8 | 0,041449 | 0,264546 | 0,508432 | DNA/RNA processing |
| ENSG00000140848 | DHX15 | 1,384555 | 0,948438 | 0,771771 | DNA/RNA processing |
| ENSG00000103381 | DHX8 | 0,406395 | 0,560139 | 0,747095 | DNA/RNA processing |
| ENSG00000071894 | DHX9 | 0,801056 | 0,686577 | 1,033917 | DNA/RNA processing |
| ENSG00000160917 | DICER1 | 0,412768 | -0,31033 | -0,25695 | DNA/RNA processing |
| ENSG00000149532 | DKC1 | 0,966492 | 0,628528 | -0,17157 | DNA/RNA processing |
| ENSG00000099942 | DNAJC1 | 0,270914 | -0,30916 | 0,313122 | DNA/RNA processing |
| ENSG00000176390 | DNAJC2 | 0,641007 | -0,06697 | -0,15489 | DNA/RNA processing |
| ENSG00000100058 | DNMT1 | 2,130996 | 2,048527 | 1,403691 | DNA/RNA processing |
| ENSG00000213218 | DOT1L | 0,794927 | 0,872145 | 1,177415 | DNA/RNA processing |
| ENSG00000141551 | DPH1 | 0,159576 | 0,201273 | 0,394266 | DNA/RNA processing |
| ENSG00000204435 | DPY30 | 0,33144 | 0,461655 | -0,09202 | DNA/RNA processing |
| ENSG00000160213 | DSN1 | -0,3074 | 0,420639 | 0,147981 | DNA/RNA processing |
| ENSG00000236002 | DTL | 1,217166 | 1,667 | 0,58631 | DNA/RNA processing |
| ENSG00000060069 | DUSP11 | 0,564916 | 0,337474 | 0,413482 | DNA/RNA processing |
| ENSG00000164733 | DYNC1H1 | 1,295057 | 0,942178 | 0,659223 | DNA/RNA processing |
| ENSG00000142544 | E2F1 | 1,398456 | 1,577908 | 1,057178 | DNA/RNA processing |
| ENSG00000107874 | E4F1 | -0,11428 | -0,04007 | 0,226671 | DNA/RNA processing |
| ENSG00000171604 | EDC4 | 1,188749 | 0,962016 | 0,860154 | DNA/RNA processing |
| ENSG00000065615 | EEF1A1 | -0,33338 | -0,44043 | 0,34373 | DNA/RNA processing |
| ENSG00000165168 | EEF1D | 2,104668 | 2,324098 | 2,415045 | DNA/RNA processing |
| ENSG00000071967 | EEF2 | 3,959995 | 3,697318 | 3,320283 | DNA/RNA processing |
| ENSG00000108669 | EFTUD2 | 1,592483 | 1,510078 | 1,135974 | DNA/RNA processing |
| ENSG00000128487 | EHMT1 | 0,17599 | 0,069442 | 0,206006 | DNA/RNA processing |
| ENSG00000129562 | EIF1 | 4,119565 | 3,986903 | 4,818673 | DNA/RNA processing |
| ENSG00000129562 | EIF1AX | 0,801819 | 0,726532 | 0,729828 | DNA/RNA processing |
| ENSG00000164535 | EIF2A | 0,975273 | 0,895524 | 0,0523 | DNA/RNA processing |
| ENSG00000035664 | EIF2AK2 | -0,20741 | -0,80803 | 0,128387 | DNA/RNA processing |
| ENSG00000115866 | EIF2B1 | 0,462407 | 0,127278 | -0,17606 | DNA/RNA processing |
| ENSG00000071626 | EIF2C1 | -0,56917 | -0,77101 | 0,085064 | DNA/RNA processing |
| ENSG00000183283 | EIF2C4 | -1,91055 | -1,52724 | 0,09682 | DNA/RNA processing |
| ENSG00000003249 | EIF2S2 | 0,069042 | 0,406101 | 0,428023 | DNA/RNA processing |
| ENSG00000136279 | EIF2S3 | 2,343645 | 1,895206 | 2,064589 | DNA/RNA processing |
| ENSG00000105516 | EIF3A | 2,696185 | 2,713828 | 2,053979 | DNA/RNA processing |
| ENSG00000132017 | EIF3B | 1,853965 | 1,711303 | 1,439433 | DNA/RNA processing |
| ENSG00000139990 | EIF3D | 1,746211 | 1,657655 | 0,845489 | DNA/RNA processing |
| ENSG00000136485 | EIF3E | 0,11304 | 0,319856 | -0,04913 | DNA/RNA processing |
| ENSG00000172992 | EIF3F | 1,329571 | 1,334545 | 0,469693 | DNA/RNA processing |
| ENSG00000167969 | EIF3G | 1,775135 | 1,769634 | 1,376269 | DNA/RNA processing |
| ENSG00000156136 | EIF3H | 1,554534 | 1,397512 | 1,258489 | DNA/RNA processing |
| ENSG00000162290 | EIF3I | 1,227978 | 1,246447 | 0,602966 | DNA/RNA processing |
| ENSG00000172795 | EIF3J | 0,162776 | -0,32604 | 0,042093 | DNA/RNA processing |
| ENSG00000204843 | EIF3K | 1,895609 | 1,807172 | 1,630036 | DNA/RNA processing |
| ENSG00000132912 | EIF4A1 | 2,308925 | 2,283523 | 1,649035 | DNA/RNA processing |
| ENSG00000166847 | EIF4A2 | 1,10195 | 1,030298 | 1,452521 | DNA/RNA processing |
| ENSG00000179958 | EIF4A3 | 0,607979 | 0,794265 | 0,935664 | DNA/RNA processing |
| ENSG00000043093 | EIF4E1B | 0,055676 | -0,43004 | 0,004847 | DNA/RNA processing |
| ENSG00000169738 | EIF4E3 | -1,2106 | -0,6906 | 1,286703 | DNA/RNA processing |
| ENSG00000130311 | EIF4EBP1 | 1,316937 | 1,083514 | 0,734489 | DNA/RNA processing |
| ENSG00000213722 | EIF4EBP2 | 1,307325 | 1,71557 | 1,976771 | DNA/RNA processing |
| ENSG00000167986 | EIF4G1 | 0,726335 | 0,756476 | 0,477349 | DNA/RNA processing |
| ENSG00000100523 | EIF4G2 | 3,718245 | 3,110903 | 3,578943 | DNA/RNA processing |
| ENSG00000197312 | EIF4G3 | -0,59594 | -0,39362 | 0,276595 | DNA/RNA processing |
| ENSG00000175197 | EIF5 | 0,238497 | 0,180459 | 1,89628 | DNA/RNA processing |
| ENSG00000079785 | ELAVL1 | 0,145443 | 0,288229 | 0,31688 | DNA/RNA processing |
| ENSG00000174243 | ELL | -0,64072 | -0,50134 | 0,766863 | DNA/RNA processing |
| ENSG00000108654 | EME2 | 0,602093 | 0,292091 | 0,564151 | DNA/RNA processing |
| ENSG00000108654 | ENDOG | 0,222472 | 0,047535 | 0,445108 | DNA/RNA processing |
| ENSG00000108654 | ENY2 | -0,0046 | -0,10439 | 0,303654 | DNA/RNA processing |
| ENSG00000108654 | EP400 | 0,291349 | 0,373458 | 0,415948 | DNA/RNA processing |
| ENSG00000110367 | EPC1 | 0,469547 | -0,14314 | 0,180502 | DNA/RNA processing |
| ENSG00000198837 | ERCC1 | 0,079642 | 0,030975 | -0,06454 | DNA/RNA processing |
| ENSG00000184014 | ERCC2 | -0,18662 | -0,07666 | 0,218708 | DNA/RNA processing |
| ENSG00000136986 | ERCC3 | 0,278499 | -0,13482 | -0,61082 | DNA/RNA processing |
| ENSG00000162496 | ESPL1 | -0,89472 | -0,09935 | 0,322015 | DNA/RNA processing |
| ENSG00000132153 | ESRRA | 0,230251 | 0,033677 | 0,640174 | DNA/RNA processing |
| ENSG00000131504 | ETF1 | 0,248962 | -0,08558 | 0,37735 | DNA/RNA processing |
| ENSG00000164031 | EXOSC1 | 0,340277 | 0,501096 | -0,44011 | DNA/RNA processing |
| ENSG00000135924 | EXOSC10 | 2,196389 | 1,884205 | 1,340032 | DNA/RNA processing |
| ENSG00000105993 | EXOSC2 | 0,441638 | 0,904897 | -0,19867 | DNA/RNA processing |
| ENSG00000136770 | EXOSC4 | -0,01902 | 0,204173 | 0,320145 | DNA/RNA processing |
| ENSG00000007923 | EXOSC6 | 1,928996 | 1,607422 | 1,729753 | DNA/RNA processing |
| ENSG00000120675 | EXOSC9 | 0,388618 | 0,011751 | -0,10781 | DNA/RNA processing |
| ENSG00000102580 | EZH1 | -0,58142 | -0,09212 | 1,228289 | DNA/RNA processing |
| ENSG00000101152 | EZH2 | 0,935559 | 0,881674 | 1,019894 | DNA/RNA processing |
| ENSG00000130159 | FAM96B | 0,601007 | 0,618151 | 0,653106 | DNA/RNA processing |
| ENSG00000038358 | FANCA | 0,138764 | 0,055469 | -0,29043 | DNA/RNA processing |
| ENSG00000134109 | FANCD2 | 0,072922 | 0,11226 | -0,21902 | DNA/RNA processing |
| ENSG00000116406 | FANCI | 0,527141 | 0,933534 | 0,338429 | DNA/RNA processing |
| ENSG00000107223 | FARSA | 1,838377 | 2,145648 | 1,716076 | DNA/RNA processing |
| ENSG00000156508 | FARSB | 0,508305 | 0,69346 | 0,082376 | DNA/RNA processing |
| ENSG00000167658 | FAU | -3,69451 | -4,30436 | -3,89507 | DNA/RNA processing |
| ENSG00000100353 | FEN1 | 0,583033 | 1,080505 | 0,425803 | DNA/RNA processing |
| ENSG00000175390 | FIP1L1 | 0,477642 | 0,314455 | 0,130642 | DNA/RNA processing |
| ENSG00000110321 | FOS | 1,041568 | 0,989906 | 1,95571 | DNA/RNA processing |
| ENSG00000110321 | FOXM1 | 0,550772 | 0,794923 | 0,236571 | DNA/RNA processing |
| ENSG00000118985 | FTSJD2 | -0,48614 | -0,64003 | 0,401049 | DNA/RNA processing |
| ENSG00000049540 | FUS | 2,666515 | 2,580758 | 2,56078 | DNA/RNA processing |
| ENSG00000074800 | FZR1 | 0,251097 | 0,416797 | 2,04755 | DNA/RNA processing |
| ENSG00000197217 | G3BP2 | -0,04314 | -0,14778 | 0,20391 | DNA/RNA processing |
| ENSG00000072134 | GADD45A | -0,75855 | -0,57711 | 1,2342 | DNA/RNA processing |
| ENSG00000187266 | GADD45G | -1,38471 | -1,0714 | 0,359524 | DNA/RNA processing |
| ENSG00000100632 | GAR1 | 0,235808 | 0,073529 | 0,192712 | DNA/RNA processing |
| ENSG00000100632 | GARS | 1,137741 | 0,923392 | 0,242223 | DNA/RNA processing |
| ENSG00000100632 | GATAD2A | 1,429858 | 1,114907 | 1,453137 | DNA/RNA processing |
| ENSG00000173153 | GCN1L1 | 0,820953 | 0,491571 | 0,222074 | DNA/RNA processing |
| ENSG00000117036 | GFI1B | 0,729887 | 1,387495 | 1,068634 | DNA/RNA processing |
| ENSG00000171311 | GINS2 | 0,61086 | 0,528901 | -0,02189 | DNA/RNA processing |
| ENSG00000184731 | GNAS | 1,636058 | 1,513349 | 1,921752 | DNA/RNA processing |
| ENSG00000179115 | GSPT1 | 1,304549 | 1,536653 | 1,992368 | DNA/RNA processing |
| ENSG00000099364 | GTF2B | 0,063003 | -0,33764 | 0,872542 | DNA/RNA processing |
| ENSG00000108306 | GTF2F1 | 1,284026 | 1,218583 | 0,818951 | DNA/RNA processing |
| ENSG00000118564 | GTF3A | 1,196672 | 0,858504 | 0,287694 | DNA/RNA processing |
| ENSG00000135722 | GTF3C1 | -0,06751 | 0,050328 | 0,361931 | DNA/RNA processing |
| ENSG00000186431 | H1F0 | 3,044165 | 3,390778 | 4,005253 | DNA/RNA processing |
| ENSG00000158869 | H1FNT | 0,212638 | -0,25382 | 0,277826 | DNA/RNA processing |
| ENSG00000143226 | H1FX | 1,736396 | 1,919896 | 2,018665 | DNA/RNA processing |
| ENSG00000104870 | H2AFV | 1,741193 | 1,495237 | 2,141273 | DNA/RNA processing |
| ENSG00000157107 | H2AFX | 1,438229 | 1,669707 | 1,594408 | DNA/RNA processing |
| ENSG00000085265 | H2AFY | 1,664596 | 1,63916 | 2,155515 | DNA/RNA processing |
| ENSG00000079459 | H2AFZ | 0,862001 | 0,598248 | 0,538212 | DNA/RNA processing |
| ENSG00000066926 | H2BFM | 0,47151 | 0,236122 | 0,654324 | DNA/RNA processing |
| ENSG00000169018 | H3F3A | 4,478455 | 4,600463 | 4,936703 | DNA/RNA processing |
| ENSG00000168496 | H3F3C | 1,347743 | 1,58366 | 2,171413 | DNA/RNA processing |
| ENSG00000111790 | HARS | 0,489356 | 0,707718 | -0,28983 | DNA/RNA processing |
| ENSG00000000938 | HAUS4 | 0,571827 | 0,787013 | 0,041878 | DNA/RNA processing |
| ENSG00000137460 | HAUS6 | 0,570041 | -0,31454 | -0,50617 | DNA/RNA processing |
| ENSG00000214253 | HBS1L | 1,139448 | 0,775798 | 0,187072 | DNA/RNA processing |
| ENSG00000196924 | HDAC1 | 0,330075 | 0,330649 | 0,501639 | DNA/RNA processing |
| ENSG00000137312 | HDAC2 | 0,819787 | 0,724962 | 0,492751 | DNA/RNA processing |
| ENSG00000132589 | HDAC3 | 1,193067 | 1,621457 | 1,08631 | DNA/RNA processing |
| ENSG00000090554 | HDAC4 | -0,5099 | -0,39276 | 0,348999 | DNA/RNA processing |
| ENSG00000059122 | HDAC5 | -0,80719 | -0,79956 | 0,534399 | DNA/RNA processing |
| ENSG00000162076 | HDAC6 | -0,1784 | -0,20888 | 0,382153 | DNA/RNA processing |
| ENSG00000184922 | HDAC7 | 1,140687 | 1,032267 | 0,416279 | DNA/RNA processing |
| ENSG00000109920 | HEATR1 | 0,071639 | -0,2763 | -0,7911 | DNA/RNA processing |
| ENSG00000118689 | HIF1A | 0,710207 | 0,763259 | 2,997173 | DNA/RNA processing |
| ENSG00000107130 | HIRA | 0,47426 | 0,483281 | 1,394074 | DNA/RNA processing |
| ENSG00000075618 | HIST1H1B | 2,906385 | 2,395794 | 1,707479 | DNA/RNA processing |
| ENSG00000070404 | HIST1H1C | 2,172402 | 1,846444 | 1,738441 | DNA/RNA processing |
| ENSG00000160282 | HIST1H1D | 2,362735 | 2,254059 | 1,287179 | DNA/RNA processing |
| ENSG00000213453 | HIST1H1E | 5,932725 | 5,528783 | 5,722303 | DNA/RNA processing |
| ENSG00000087086 | HIST1H2AB | 0,356836 | 0,590394 | 0,071284 | DNA/RNA processing |
| ENSG00000137200 | HIST1H2AC | 2,192917 | 2,194073 | 2,072637 | DNA/RNA processing |
| ENSG00000188529 | HIST1H2BB | 0,986823 | 0,840255 | 0,251793 | DNA/RNA processing |
| ENSG00000196371 | HIST1H2BH | 4,575205 | 4,235048 | 3,421973 | DNA/RNA processing |
| ENSG00000010361 | HIST1H2BK | -0,74762 | -0,94683 | 0,113091 | DNA/RNA processing |
| ENSG00000132286 | HIST1H2BM | 4,371515 | 4,564878 | 4,015893 | DNA/RNA processing |
| ENSG00000165060 | HIST1H2BN | 0,628999 | 0,044387 | -0,11662 | DNA/RNA processing |
| ENSG00000163251 | HIST1H4A | 0,070503 | 0,213081 | -0,03071 | DNA/RNA processing |
| ENSG00000177283 | HIST1H4B | 1,550363 | 1,734282 | 1,257511 | DNA/RNA processing |
| ENSG00000160211 | HIST2H2AB | 2,703135 | 2,830028 | 2,787833 | DNA/RNA processing |
| ENSG00000109458 | HIST2H2AC | 2,970845 | 2,920253 | 3,008353 | DNA/RNA processing |
| ENSG00000033327 | HIST2H2BE | 0,009488 | -0,49237 | 0,437384 | DNA/RNA processing |
| ENSG00000170296 | HJURP | 0,053264 | 0,585955 | 1,103712 | DNA/RNA processing |
| ENSG00000164574 | HLTF | 0,525413 | 0,306271 | -1,05634 | DNA/RNA processing |
| ENSG00000130005 | HMG20B | 0,660359 | 0,481476 | 0,686047 | DNA/RNA processing |
| ENSG00000089597 | HMGA1 | 2,349515 | 2,266652 | 2,045224 | DNA/RNA processing |
| ENSG00000175857 | HMGB1 | 0,325895 | 0,184994 | 0,305622 | DNA/RNA processing |
| ENSG00000106105 | HMGB2 | 2,196495 | 2,415733 | 2,817743 | DNA/RNA processing |
| ENSG00000185340 | HMGB3 | 0,716944 | 0,349075 | 0,201684 | DNA/RNA processing |
| ENSG00000179348 | HMGN2 | 3,445065 | 3,461793 | 3,905073 | DNA/RNA processing |
| ENSG00000107862 | HNRNPA0 | 0,607202 | 0,488469 | 0,683604 | DNA/RNA processing |
| ENSG00000148288 | HNRNPA1 | -1,68402 | -1,02078 | -1,44357 | DNA/RNA processing |
| ENSG00000168505 | HNRNPA2B1 | 1,162051 | 1,466143 | 2,035374 | DNA/RNA processing |
| ENSG00000105607 | HNRNPC | -0,02651 | 0,392324 | 0,751685 | DNA/RNA processing |
| ENSG00000001084 | HNRNPD | 3,366495 | 3,456658 | 3,226593 | DNA/RNA processing |
| ENSG00000089154 | HNRNPF | 1,400484 | 1,447033 | 1,277338 | DNA/RNA processing |
| ENSG00000006007 | HNRNPH1 | 2,949385 | 2,482778 | 2,824553 | DNA/RNA processing |
| ENSG00000143869 | HNRNPH2 | -0,0725 | -0,21532 | 0,042651 | DNA/RNA processing |
| ENSG00000203879 | HNRNPH3 | 0,582576 | 0,302465 | 0,531047 | DNA/RNA processing |
| ENSG00000057608 | HNRNPK | 1,058757 | 0,494922 | 1,450301 | DNA/RNA processing |
| ENSG00000057608 | HNRNPL | 3,416575 | 3,182058 | 3,012363 | DNA/RNA processing |
| ENSG00000057608 | HNRNPM | 0,817102 | 0,488438 | 0,460399 | DNA/RNA processing |
| ENSG00000057608 | HNRNPR | 1,337214 | 1,103129 | 0,747311 | DNA/RNA processing |
| ENSG00000057608 | HNRNPU | 2,07772 | 1,785637 | 1,990633 | DNA/RNA processing |
| ENSG00000057608 | HNRNPUL1 | 1,963275 | 1,983714 | 2,187365 | DNA/RNA processing |
| ENSG00000057608 | HNRPDL | 2,043138 | 1,58861 | 1,708853 | DNA/RNA processing |
| ENSG00000165702 | HP1BP3 | 1,720909 | 1,672645 | 1,916719 | DNA/RNA processing |
| ENSG00000137198 | HSPA8 | 4,062365 | 4,496873 | 3,797413 | DNA/RNA processing |
| ENSG00000146535 | HSPB1 | 1,756866 | 2,218378 | 1,186864 | DNA/RNA processing |
| ENSG00000134697 | IARS | 1,6138 | 1,35136 | 0,273332 | DNA/RNA processing |
| ENSG00000183134 | IGFBP4 | 0,470154 | 0,134242 | 0,767319 | DNA/RNA processing |
| ENSG00000121957 | IKZF1 | 0,584283 | 1,121804 | 1,857 | DNA/RNA processing |
| ENSG00000082701 | ILK | -0,11427 | 0,504137 | 1,331286 | DNA/RNA processing |
| ENSG00000103342 | IMP4 | 0,587106 | 0,609934 | 0,014926 | DNA/RNA processing |
| ENSG00000134184 | INCENP | 0,007328 | 0,106435 | 0,227353 | DNA/RNA processing |
| ENSG00000169840 | INO80 | 0,099762 | -0,19205 | 0,095676 | DNA/RNA processing |
| ENSG00000180613 | INO80D | 0,43359 | 0,335463 | 0,539624 | DNA/RNA processing |
| ENSG00000137947 | INO80E | -0,27367 | -0,49996 | 0,013956 | DNA/RNA processing |
| ENSG00000172432 | INTS3 | 0,464638 | 0,713679 | 0,585836 | DNA/RNA processing |
| ENSG00000163041 | ISG20 | -1,05202 | -1,03469 | 0,218363 | DNA/RNA processing |
| ENSG00000172534 | IVNS1ABP | -0,17031 | 0,472311 | 2,196755 | DNA/RNA processing |
| ENSG00000164818 | JAK2 | -1,06168 | -0,16865 | 0,033743 | DNA/RNA processing |
| ENSG00000179832 | JARID2 | 1,704136 | 1,283622 | 1,19549 | DNA/RNA processing |
| ENSG00000112406 | JHDM1D | -1,77286 | -1,42739 | 1,033444 | DNA/RNA processing |
| ENSG00000092148 | JMJD1C | 0,611495 | -0,07305 | 2,241097 | DNA/RNA processing |
| ENSG00000126107 | JMJD6 | -0,29757 | -0,50427 | 0,9667 | DNA/RNA processing |
| ENSG00000136929 | JMY | -0,52612 | -0,9664 | 0,243982 | DNA/RNA processing |
| ENSG00000165102 | KARS | -1,06718 | -0,55095 | -1,43302 | DNA/RNA processing |
| ENSG00000156875 | KAT2A | 1,357626 | 0,842454 | 0,031347 | DNA/RNA processing |
| ENSG00000148110 | KAT2B | 0,078964 | 0,711434 | 2,296135 | DNA/RNA processing |
| ENSG00000196747 | KDM2A | 0,711469 | 0,66508 | 1,500522 | DNA/RNA processing |
| ENSG00000184348 | KDM3A | 0,411015 | -0,08724 | 0,816272 | DNA/RNA processing |
| ENSG00000198374 | KDM3B | 0,341527 | 0,550077 | 1,273494 | DNA/RNA processing |
| ENSG00000196226 | KDM4B | -0,42303 | -0,37719 | 0,52575 | DNA/RNA processing |
| ENSG00000197846 | KDM4C | -0,65535 | 0,035184 | 0,77735 | DNA/RNA processing |
| ENSG00000197459 | KDM5A | -0,17244 | -0,54952 | 0,496858 | DNA/RNA processing |
| ENSG00000197903 | KDM5B | -0,30502 | -0,29566 | 0,431277 | DNA/RNA processing |
| ENSG00000196374 | KDM5C | 0,698363 | 0,584327 | 1,234765 | DNA/RNA processing |
| ENSG00000233822 | KDM5D | 0,070798 | -2,74616 | -2,34746 | DNA/RNA processing |
| ENSG00000198366 | KDM6A | -0,07342 | 0,183899 | 1,712342 | DNA/RNA processing |
| ENSG00000124693 | KDM6B | -0,3967 | -0,30987 | 0,938318 | DNA/RNA processing |
| ENSG00000196176 | KHDRBS1 | 2,958055 | 2,828553 | 2,734203 | DNA/RNA processing |
| ENSG00000197061 | KHSRP | 1,588595 | 1,483525 | 0,939855 | DNA/RNA processing |
| ENSG00000234745 | KIAA0415 | -0,44809 | -0,49021 | 0,146843 | DNA/RNA processing |
| ENSG00000135486 | KIF22 | 1,748175 | 1,971055 | 1,812531 | DNA/RNA processing |
| ENSG00000092199 | KLF1 | 2,231737 | 2,318318 | 2,157391 | DNA/RNA processing |
| ENSG00000169045 | KPNA1 | -0,63512 | -0,29379 | 0,254994 | DNA/RNA processing |
| ENSG00000126945 | KPNA2 | 1,391184 | 1,682661 | 1,623127 | DNA/RNA processing |
| ENSG00000165119 | KPNB1 | 0,770783 | 0,862003 | 0,638906 | DNA/RNA processing |
| ENSG00000165119 | KRR1 | 0,465994 | -0,07488 | -0,26352 | DNA/RNA processing |
| ENSG00000099783 | L3MBTL2 | 0,013773 | 0,1212 | -0,2338 | DNA/RNA processing |
| ENSG00000197711 | LARS | -0,4731 | 0,014889 | -1,27473 | DNA/RNA processing |
| ENSG00000107521 | LAT | -0,0739 | -0,63458 | 0,435872 | DNA/RNA processing |
| ENSG00000096384 | LGALS3 | -0,85702 | -0,32128 | 1,37772 | DNA/RNA processing |
| ENSG00000096384 | LIG1 | 0,954649 | 1,11835 | 0,734964 | DNA/RNA processing |
| ENSG00000096384 | LIG3 | 0,199345 | -0,23005 | 0,176955 | DNA/RNA processing |
| ENSG00000197081 | LRPPRC | 0,827049 | 0,433306 | -0,91199 | DNA/RNA processing |
| ENSG00000115607 | LRWD1 | 0,19992 | 0,221732 | 0,499938 | DNA/RNA processing |
| ENSG00000197110 | LSM14A | 0,780524 | 0,496633 | 0,908604 | DNA/RNA processing |
| ENSG00000147168 | LSM3 | 0,091682 | 0,304195 | 0,042692 | DNA/RNA processing |
| ENSG00000077238 | LSM4 | 1,731033 | 1,527562 | 1,378181 | DNA/RNA processing |
| ENSG00000160712 | LSM7 | 0,082924 | -0,20125 | -0,20592 | DNA/RNA processing |
| ENSG00000143621 | LTB | 0,695732 | 0,570841 | 1,366748 | DNA/RNA processing |
| ENSG00000143621 | LUC7L3 | 0,339805 | -0,17878 | 0,320947 | DNA/RNA processing |
| ENSG00000128908 | MAD2L2 | 0,240379 | 0,571026 | 0,613666 | DNA/RNA processing |
| ENSG00000140968 | MAPK14 | -0,34239 | -0,34944 | 0,8961 | DNA/RNA processing |
| ENSG00000136003 | MAPKAPK2 | 0,110108 | -0,088 | 1,366485 | DNA/RNA processing |
| ENSG00000150093 | MARS | 0,432807 | 0,518102 | 0,61875 | DNA/RNA processing |
| ENSG00000210049 | MBD4 | -0,07229 | 0,141309 | 0,597946 | DNA/RNA processing |
| ENSG00000210196 | MBNL1 | 2,597765 | 2,664583 | 2,922963 | DNA/RNA processing |
| ENSG00000210191 | MBNL2 | 0,141512 | -0,06595 | 1,207033 | DNA/RNA processing |
| ENSG00000210117 | MBNL3 | 0,629046 | 0,845358 | 2,590745 | DNA/RNA processing |
| ENSG00000198695 | MCM10 | -0,3977 | 0,234222 | -0,88282 | DNA/RNA processing |
| ENSG00000198840 | MCM2 | 1,726608 | 1,892582 | 1,283077 | DNA/RNA processing |
| ENSG00000162434 | MCM3 | 1,76279 | 1,877501 | 0,7237 | DNA/RNA processing |
| ENSG00000096968 | MCM4 | 1,050541 | 1,134409 | 0,262602 | DNA/RNA processing |
| ENSG00000008083 | MCM5 | 2,212511 | 2,08434 | 1,189466 | DNA/RNA processing |
| ENSG00000153814 | MCM6 | -0,22456 | 0,041178 | -0,31483 | DNA/RNA processing |
| ENSG00000140044 | MCM7 | 2,715955 | 2,846253 | 2,204454 | DNA/RNA processing |
| ENSG00000006459 | MCM8 | -0,05878 | 0,345163 | -0,03538 | DNA/RNA processing |
| ENSG00000070495 | MCRS1 | 0,353826 | 0,328398 | 0,345443 | DNA/RNA processing |
| ENSG00000143543 | MED1 | -0,13325 | 0,145915 | 0,031828 | DNA/RNA processing |
| ENSG00000143543 | MED12 | 0,794817 | 0,721746 | 0,783087 | DNA/RNA processing |
| ENSG00000143543 | MED13 | 0,550711 | 0,554876 | 1,761864 | DNA/RNA processing |
| ENSG00000143543 | MED14 | 0,451029 | 0,349876 | 0,750429 | DNA/RNA processing |
| ENSG00000143543 | MED15 | 1,303465 | 1,067591 | 1,92826 | DNA/RNA processing |
| ENSG00000143543 | MED16 | 0,974098 | 1,093357 | 1,024888 | DNA/RNA processing |
| ENSG00000177606 | MED23 | -0,02355 | -0,41298 | 0,174233 | DNA/RNA processing |
| ENSG00000171223 | MED24 | 1,529718 | 1,407791 | 0,8225 | DNA/RNA processing |
| ENSG00000130522 | MED25 | 1,138322 | 1,449739 | 1,836562 | DNA/RNA processing |
| ENSG00000197256 | MED26 | 0,038909 | -0,1075 | 0,603821 | DNA/RNA processing |
| ENSG00000114166 | METTL3 | 0,022988 | 0,169548 | 0,110368 | DNA/RNA processing |
| ENSG00000147050 | MKI67 | 0,562582 | 1,183645 | 1,7273 | DNA/RNA processing |
| ENSG00000132510 | MKI67IP | 0,451657 | 0,574198 | 0,253743 | DNA/RNA processing |
| ENSG00000121774 | MKNK1 | 0,153773 | 0,126728 | 1,10089 | DNA/RNA processing |
| ENSG00000121774 | MKNK2 | 1,262593 | 2,025401 | 2,877363 | DNA/RNA processing |
| ENSG00000121774 | MLF1IP | 1,083946 | 0,786839 | -0,01848 | DNA/RNA processing |
| ENSG00000121774 | MLL | 0,526996 | 0,775153 | 0,788817 | DNA/RNA processing |
| ENSG00000121774 | MLL2 | 0,496048 | 0,589629 | 1,580298 | DNA/RNA processing |
| ENSG00000121774 | MLL3 | 1,155791 | 1,070641 | 1,779327 | DNA/RNA processing |
| ENSG00000121774 | MLL5 | 0,801038 | 0,893521 | 2,054167 | DNA/RNA processing |
| ENSG00000088247 | MLX | 0,006558 | 0,305766 | 0,808367 | DNA/RNA processing |
| ENSG00000177728 | MMS19 | 0,271026 | 0,237519 | 0,06035 | DNA/RNA processing |
| ENSG00000132361 | MORF4L1 | 1,56765 | 1,481451 | 1,92564 | DNA/RNA processing |
| ENSG00000126775 | MORF4L2 | 0,066653 | 0,038354 | -0,19345 | DNA/RNA processing |
| ENSG00000138688 | MPG | 0,056492 | -0,13085 | 0,428718 | DNA/RNA processing |
| ENSG00000144320 | MRPL11 | 0,564895 | 0,682919 | 0,497293 | DNA/RNA processing |
| ENSG00000158941 | MRPL14 | 0,119095 | 0,006729 | 0,159552 | DNA/RNA processing |
| ENSG00000116685 | MRPL16 | 0,700018 | 0,337535 | -0,54437 | DNA/RNA processing |
| ENSG00000176542 | MRPL17 | 0,848594 | 0,859773 | 0,731741 | DNA/RNA processing |
| ENSG00000183354 | MRPL19 | 0,015172 | 0,071504 | -0,27201 | DNA/RNA processing |
| ENSG00000134313 | MRPL2 | 0,114778 | 0,626181 | -0,26122 | DNA/RNA processing |
| ENSG00000137177 | MRPL20 | 1,316229 | 1,592523 | 0,841644 | DNA/RNA processing |
| ENSG00000186185 | MRPL21 | 0,787689 | 0,627676 | -0,34632 | DNA/RNA processing |
| ENSG00000054523 | MRPL23 | 0,84988 | 0,993134 | 1,054523 | DNA/RNA processing |
| ENSG00000116852 | MRPL24 | 0,127805 | -0,02402 | -0,61173 | DNA/RNA processing |
| ENSG00000079616 | MRPL28 | 0,649135 | 0,769605 | 0,627827 | DNA/RNA processing |
| ENSG00000066735 | MRPL3 | 0,397499 | 0,326948 | -0,36494 | DNA/RNA processing |
| ENSG00000170759 | MRPL34 | 0,499871 | 0,670221 | 0,623338 | DNA/RNA processing |
| ENSG00000237649 | MRPL36 | 3,389085 | 3,458993 | 3,860193 | DNA/RNA processing |
| ENSG00000237649 | MRPL37 | 1,809204 | 2,081489 | 1,367065 | DNA/RNA processing |
| ENSG00000167702 | MRPL4 | 0,827964 | 0,574041 | 0,2083 | DNA/RNA processing |
| ENSG00000157404 | MRPL41 | 0,621883 | 0,493538 | 0,652395 | DNA/RNA processing |
| ENSG00000105610 | MRPL45 | 1,305515 | 1,507031 | 0,315348 | DNA/RNA processing |
| ENSG00000155090 | MRPL47 | 0,434767 | 0,119692 | 0,059207 | DNA/RNA processing |
| ENSG00000172059 | MRPL51 | 2,084887 | 2,504893 | 1,844008 | DNA/RNA processing |
| ENSG00000169926 | MRPL55 | 0,102382 | -0,07776 | 0,272997 | DNA/RNA processing |
| ENSG00000129911 | MRPL9 | 0,749414 | 0,419791 | -0,18962 | DNA/RNA processing |
| ENSG00000127528 | MRPS10 | -0,12741 | 0,02111 | -0,3001 | DNA/RNA processing |
| ENSG00000109787 | MRPS11 | 1,681184 | 1,665574 | 1,040965 | DNA/RNA processing |
| ENSG00000102554 | MRPS15 | 1,935392 | 1,74172 | 0,888293 | DNA/RNA processing |
| ENSG00000067082 | MRPS16 | 0,025443 | 0,057251 | 0,137577 | DNA/RNA processing |
| ENSG00000118263 | MRPS18A | 0,544163 | 0,692839 | 0,352434 | DNA/RNA processing |
| ENSG00000119138 | MRPS18B | 0,726983 | 0,63889 | 0,063551 | DNA/RNA processing |
| ENSG00000165516 | MRPS2 | 1,330151 | 1,330891 | 0,511135 | DNA/RNA processing |
| ENSG00000124702 | MRPS21 | 1,151456 | 0,939591 | 0,341494 | DNA/RNA processing |
| ENSG00000174010 | MRPS24 | 2,080027 | 2,054941 | 1,125691 | DNA/RNA processing |
| ENSG00000187961 | MRPS25 | 0,159611 | 0,186353 | -0,38921 | DNA/RNA processing |
| ENSG00000114648 | MRPS26 | 0,207555 | 0,070126 | 0,195846 | DNA/RNA processing |
| ENSG00000114796 | MRPS5 | 0,403552 | 0,420813 | 0,144518 | DNA/RNA processing |
| ENSG00000179454 | MRPS7 | 0,549092 | 0,280766 | -0,34168 | DNA/RNA processing |
| ENSG00000025800 | MSL1 | 0,613532 | 0,744605 | 1,729483 | DNA/RNA processing |
| ENSG00000221837 | MTA2 | 1,619124 | 1,184896 | 1,171739 | DNA/RNA processing |
| ENSG00000221864 | MTCH1 | 0,349596 | 0,834738 | 1,884286 | DNA/RNA processing |
| ENSG00000185896 | MTPN | 1,894147 | 2,113356 | 3,126403 | DNA/RNA processing |
| ENSG00000150457 | MYB | 2,691525 | 2,415873 | 0,364855 | DNA/RNA processing |
| ENSG00000213626 | MYBBP1A | 0,858085 | 0,743756 | -0,18851 | DNA/RNA processing |
| ENSG00000188779 | MYC | 2,287605 | 1,903519 | 0,140081 | DNA/RNA processing |
| ENSG00000043462 | MYO18A | -0,90231 | -0,39809 | 0,473523 | DNA/RNA processing |
| ENSG00000243709 | MYSM1 | 0,622741 | 0,177666 | 0,192581 | DNA/RNA processing |
| ENSG00000168924 | NACA | 2,724915 | 2,571813 | 1,880253 | DNA/RNA processing |
| ENSG00000104826 | NANOS3 | 0,371363 | 0,17527 | 0,806903 | DNA/RNA processing |
| ENSG00000145685 | NAP1L1 | 0,587912 | 0,75303 | 0,750427 | DNA/RNA processing |
| ENSG00000107187 | NAP1L4 | 0,821859 | 0,746684 | 1,350117 | DNA/RNA processing |
| ENSG00000170866 | NARS | 1,16707 | 1,134759 | 1,452997 | DNA/RNA processing |
| ENSG00000187116 | NASP | 0,878185 | 0,5217 | -0,23268 | DNA/RNA processing |
| ENSG00000220008 | NCAPD2 | 1,028115 | 1,770305 | 1,197993 | DNA/RNA processing |
| ENSG00000236850 | NCAPG2 | -0,3774 | 0,150706 | 0,286512 | DNA/RNA processing |
| ENSG00000139233 | NCAPH | 0,030882 | 0,549714 | 0,27579 | DNA/RNA processing |
| ENSG00000074695 | NCAPH2 | -0,12834 | 0,068764 | 0,357504 | DNA/RNA processing |
| ENSG00000169223 | NCBP1 | 0,408811 | 0,306647 | 0,242028 | DNA/RNA processing |
| ENSG00000113368 | NCK2 | -0,30579 | -0,15477 | 1,184009 | DNA/RNA processing |
| ENSG00000164715 | NCLN | 1,063574 | 1,061344 | 1,062574 | DNA/RNA processing |
| ENSG00000102910 | NCOR1 | 1,640521 | 1,238188 | 1,450557 | DNA/RNA processing |
| ENSG00000123684 | NDEL1 | 1,047764 | 0,723226 | 2,430355 | DNA/RNA processing |
| ENSG00000213903 | NFE2 | -0,31362 | 0,398597 | 1,835274 | DNA/RNA processing |
| ENSG00000168056 | NFIA | 1,728349 | 2,337933 | 0,822634 | DNA/RNA processing |
| ENSG00000012223 | NFIC | 0,595877 | 0,533055 | 1,011426 | DNA/RNA processing |
| ENSG00000146963 | NFIX | 0,351465 | 0,541831 | 0,594067 | DNA/RNA processing |
| ENSG00000133315 | NFRKB | 0,168077 | -0,10656 | -0,011 | DNA/RNA processing |
| ENSG00000110514 | NHP2L1 | 0,581754 | 0,057995 | -0,27423 | DNA/RNA processing |
| ENSG00000173327 | NOC2L | 0,709337 | 0,938668 | 0,420421 | DNA/RNA processing |
| ENSG00000169967 | NOL3 | 0,496521 | 0,103498 | 0,558145 | DNA/RNA processing |
| ENSG00000198909 | NOLC1 | 1,279901 | 0,98016 | -0,78025 | DNA/RNA processing |
| ENSG00000085511 | NONO | 3,033745 | 2,312873 | 2,073054 | DNA/RNA processing |
| ENSG00000197442 | NOP10 | 1,750583 | 1,924799 | 1,276138 | DNA/RNA processing |
| ENSG00000157625 | NOP14 | 0,788826 | 0,845697 | 0,195529 | DNA/RNA processing |
| ENSG00000107968 | NOP2 | 0,313833 | 0,503373 | -0,08134 | DNA/RNA processing |
| ENSG00000071054 | NOP58 | 1,913384 | 1,47059 | -0,05317 | DNA/RNA processing |
| ENSG00000100030 | NOTCH1 | -1,10204 | -1,10389 | 0,107911 | DNA/RNA processing |
| ENSG00000101367 | NPM1 | -1,54685 | -1,66528 | -1,30858 | DNA/RNA processing |
| ENSG00000099785 | NPM3 | 0,498519 | 0,459865 | -0,28528 | DNA/RNA processing |
| ENSG00000155130 | NR1H2 | 0,196131 | 0,495323 | 1,330901 | DNA/RNA processing |
| ENSG00000175130 | NR2C2 | 0,286827 | 0,521946 | 1,151526 | DNA/RNA processing |
| ENSG00000072518 | NR2F6 | 0,344582 | 0,334567 | 0,339099 | DNA/RNA processing |
| ENSG00000075413 | NR3C1 | -0,57173 | -0,24055 | 0,660994 | DNA/RNA processing |
| ENSG00000166986 | NR4A2 | -1,08426 | -1,28489 | 0,557853 | DNA/RNA processing |
| ENSG00000099308 | NRBP1 | 0,350887 | 0,303103 | 0,980015 | DNA/RNA processing |
| ENSG00000103495 | NSD1 | -0,07004 | 0,15557 | 0,215487 | DNA/RNA processing |
| ENSG00000134046 | NSL1 | -0,0553 | 0,063443 | 0,287795 | DNA/RNA processing |
| ENSG00000076770 | NT5M | 0,788352 | 0,759183 | 0,479656 | DNA/RNA processing |
| ENSG00000076003 | NUDT1 | 0,071204 | -0,29878 | 0,299521 | DNA/RNA processing |
| ENSG00000125885 | NUDT21 | 1,086178 | 0,971158 | 0,825045 | DNA/RNA processing |
| ENSG00000135272 | NUFIP1 | 0,420683 | 0,487271 | -0,11842 | DNA/RNA processing |
| ENSG00000198625 | NUP153 | 1,631077 | 1,125477 | 1,668134 | DNA/RNA processing |
| ENSG00000112159 | NUP155 | 0,258202 | 0,244464 | -0,03279 | DNA/RNA processing |
| ENSG00000151376 | NUP188 | 0,388135 | 0,608854 | -0,01737 | DNA/RNA processing |
| ENSG00000124733 | NUP210 | 1,947645 | 2,167353 | 1,336694 | DNA/RNA processing |
| ENSG00000169057 | NUP214 | 1,36269 | 1,173023 | 1,312334 | DNA/RNA processing |
| ENSG00000125686 | NUP43 | 0,188264 | 0,330487 | -0,80236 | DNA/RNA processing |
| ENSG00000184634 | NUP50 | -0,80769 | -0,69665 | 0,136984 | DNA/RNA processing |
| ENSG00000108510 | NUP54 | 0,055795 | -0,39548 | -0,71648 | DNA/RNA processing |
| ENSG00000123066 | NUP85 | 1,226301 | 1,041264 | 0,595631 | DNA/RNA processing |
| ENSG00000180182 | NUP88 | 1,238035 | 1,037386 | 1,578455 | DNA/RNA processing |
| ENSG00000099917 | NUP93 | 1,141312 | 1,022695 | 0,321576 | DNA/RNA processing |
| ENSG00000175221 | NUP98 | 1,152875 | 0,943034 | 1,949397 | DNA/RNA processing |
| ENSG00000152944 | NUPL1 | 0,204015 | -0,00465 | 0,160986 | DNA/RNA processing |
| ENSG00000104973 | NXF1 | 1,847462 | 1,595601 | 2,692623 | DNA/RNA processing |
| ENSG00000118579 | NXT1 | 0,150148 | -0,08091 | 0,3656 | DNA/RNA processing |
| ENSG00000116604 | OBFC2A | -1,22519 | -1,36847 | 0,431264 | DNA/RNA processing |
| ENSG00000162591 | OBFC2B | 0,412215 | 0,548692 | 0,18851 | DNA/RNA processing |
| ENSG00000196588 | PA2G4 | 2,849175 | 2,657703 | 2,401057 | DNA/RNA processing |
| ENSG00000128585 | PABPC1 | 3,043525 | 2,955188 | 3,333363 | DNA/RNA processing |
| ENSG00000079277 | PABPC4 | 1,777243 | 1,57738 | 0,529388 | DNA/RNA processing |
| ENSG00000099875 | PABPN1 | 2,213697 | 1,797921 | 1,740802 | DNA/RNA processing |
| ENSG00000151725 | PADI4 | -1,4113 | -0,60005 | 2,588479 | DNA/RNA processing |
| ENSG00000005483 | PAIP2 | 1,283745 | 1,03859 | 1,587996 | DNA/RNA processing |
| ENSG00000167965 | PAN3 | 0,858778 | 0,051211 | 1,03144 | DNA/RNA processing |
| ENSG00000196549 | PAPD4 | 0,126266 | 0,089597 | 0,744374 | DNA/RNA processing |
| ENSG00000008516 | PAPOLA | 0,851465 | 1,383268 | 1,489917 | DNA/RNA processing |
| ENSG00000155229 | PARP1 | 0,41472 | 0,052587 | -0,58905 | DNA/RNA processing |
| ENSG00000130675 | PARP4 | 0,69475 | 0,302467 | 1,069393 | DNA/RNA processing |
| ENSG00000103111 | PATL1 | 1,28321 | 1,195781 | 1,974425 | DNA/RNA processing |
| ENSG00000061987 | PAXIP1 | -0,01025 | 0,160708 | -0,61187 | DNA/RNA processing |
| ENSG00000159256 | PBRM1 | -0,64186 | -0,05189 | 0,301232 | DNA/RNA processing |
| ENSG00000185787 | PCBP1 | 2,244634 | 2,070794 | 2,42428 | DNA/RNA processing |
| ENSG00000123562 | PCBP2 | 3,194945 | 2,832173 | 3,386823 | DNA/RNA processing |
| ENSG00000106330 | PCF11 | -0,59421 | -0,02743 | 0,521493 | DNA/RNA processing |
| ENSG00000103152 | PCM1 | 1,388155 | 0,71978 | 1,073566 | DNA/RNA processing |
| ENSG00000186732 | PCNA | 0,942073 | 1,465449 | 0,787595 | DNA/RNA processing |
| ENSG00000122140 | PES1 | 0,228878 | 0,332694 | -0,66016 | DNA/RNA processing |
| ENSG00000087250 | PHB | 1,875405 | 1,713283 | 0,332783 | DNA/RNA processing |
| ENSG00000109919 | PHF1 | -0,47789 | -0,67741 | 0,758253 | DNA/RNA processing |
| ENSG00000188786 | PHF2 | 0,277209 | 0,296789 | 0,344578 | DNA/RNA processing |
| ENSG00000148824 | PHF21A | -0,17689 | -0,25176 | 0,651982 | DNA/RNA processing |
| ENSG00000150712 | PHF5A | -0,20107 | 0,003398 | 0,01782 | DNA/RNA processing |
| ENSG00000163719 | PHF8 | -0,25185 | -0,34876 | 0,556576 | DNA/RNA processing |
| ENSG00000188428 | PHRF1 | 0,104832 | 0,172854 | 0,542742 | DNA/RNA processing |
| ENSG00000083168 | PKN1 | 0,802242 | 0,356765 | 1,236651 | DNA/RNA processing |
| ENSG00000134440 | PLK1 | 1,012142 | 1,740949 | 1,367717 | DNA/RNA processing |
| ENSG00000135372 | PMF1 | 0,58979 | 0,43563 | 0,496512 | DNA/RNA processing |
| ENSG00000158747 | PNKP | 0,881525 | 0,849257 | 1,229043 | DNA/RNA processing |
| ENSG00000151503 | PNN | 2,272116 | 1,91998 | 1,245695 | DNA/RNA processing |
| ENSG00000100365 | POLA2 | 0,41011 | 0,268199 | -0,07907 | DNA/RNA processing |
| ENSG00000071051 | POLB | -0,5859 | -0,75435 | 0,060368 | DNA/RNA processing |
| ENSG00000123338 | POLD1 | 0,363616 | 0,536308 | -0,20143 | DNA/RNA processing |
| ENSG00000115053 | POLD2 | 1,442955 | 1,335425 | 0,354134 | DNA/RNA processing |
| ENSG00000125912 | POLD4 | -0,30289 | -0,18546 | 1,017191 | DNA/RNA processing |
| ENSG00000141027 | POLE | 0,840543 | 0,26824 | 0,674455 | DNA/RNA processing |
| ENSG00000196498 | POLE2 | -0,14333 | 0,186395 | -1,15304 | DNA/RNA processing |
| ENSG00000225880 | POLE3 | 1,400171 | 1,095503 | 0,495624 | DNA/RNA processing |
| ENSG00000072864 | POLR1A | 0,635472 | 0,34856 | -0,79234 | DNA/RNA processing |
| ENSG00000188566 | POLR2B | 0,537779 | 0,330284 | 0,010868 | DNA/RNA processing |
| ENSG00000104419 | POLR2C | 0,099631 | -0,08693 | 0,027037 | DNA/RNA processing |
| ENSG00000070614 | POLR2E | 1,735542 | 1,630723 | 1,305682 | DNA/RNA processing |
| ENSG00000166507 | POLR2F | 0,631584 | 0,708456 | 0,467489 | DNA/RNA processing |
| ENSG00000130414 | POLR2H | 0,060599 | 0,870182 | -0,14127 | DNA/RNA processing |
| ENSG00000184752 | POLR2J | -0,21398 | -0,29109 | 0,14725 | DNA/RNA processing |
| ENSG00000131495 | POLR2L | 2,366485 | 2,347468 | 2,388065 | DNA/RNA processing |
| ENSG00000151366 | PPA1 | 0,448988 | 0,52646 | -0,28445 | DNA/RNA processing |
| ENSG00000160194 | PPIL1 | 0,157582 | 0,123191 | -0,49437 | DNA/RNA processing |
| ENSG00000176953 | PPP1R12A | 1,314625 | 1,052684 | 1,848561 | DNA/RNA processing |
| ENSG00000123405 | PPP1R15A | 1,501226 | 1,882269 | 3,280573 | DNA/RNA processing |
| ENSG00000116044 | PPP1R15B | 0,940046 | 1,217382 | 2,933373 | DNA/RNA processing |
| ENSG00000165030 | PPP1R2 | 1,12892 | 0,89793 | 2,207018 | DNA/RNA processing |
| ENSG00000077150 | PPP1R8 | 0,088874 | 0,007073 | -0,57869 | DNA/RNA processing |
| ENSG00000104825 | PPP2CA | 0,801002 | 0,89556 | 1,927892 | DNA/RNA processing |
| ENSG00000167604 | PPP2R1A | 1,738742 | 1,342914 | 1,426215 | DNA/RNA processing |
| ENSG00000146232 | PPP2R2A | -0,79634 | -1,18377 | 0,005639 | DNA/RNA processing |
| ENSG00000171840 | PPP4R2 | -0,3125 | -0,41667 | 0,158892 | DNA/RNA processing |
| ENSG00000105374 | PPT1 | 0,174459 | 0,441306 | 1,340084 | DNA/RNA processing |
| ENSG00000147140 | PRIM1 | -0,73233 | 0,073357 | -0,51139 | DNA/RNA processing |
| ENSG00000147140 | PRKCB | -0,69932 | -0,70973 | 1,571204 | DNA/RNA processing |
| ENSG00000147140 | PRKCD | -1,26456 | -0,81925 | 1,869165 | DNA/RNA processing |
| ENSG00000182117 | PRMT5 | 0,731802 | 1,091293 | -0,33837 | DNA/RNA processing |
| ENSG00000198929 | PRPF19 | 2,222108 | 2,143266 | 1,107646 | DNA/RNA processing |
| ENSG00000142546 | PRPF3 | 0,499068 | -0,01344 | 0,794608 | DNA/RNA processing |
| ENSG00000148400 | PRPF31 | 0,660715 | 0,573093 | 0,484089 | DNA/RNA processing |
| ENSG00000188747 | PRPF38A | 0,722504 | 0,666815 | 1,061109 | DNA/RNA processing |
| ENSG00000198805 | PRPF38B | 0,574188 | 0,188988 | 0,650676 | DNA/RNA processing |
| ENSG00000149308 | PRPF40A | 0,902609 | 0,680701 | 0,585361 | DNA/RNA processing |
| ENSG00000183979 | PRPF4B | 0,663915 | 0,363549 | 0,669659 | DNA/RNA processing |
| ENSG00000119655 | PRPF6 | 0,472708 | 0,558286 | 0,145304 | DNA/RNA processing |
| ENSG00000215440 | PRPF8 | 1,706207 | 1,222498 | 1,250123 | DNA/RNA processing |
| ENSG00000154146 | PSMA1 | 0,511412 | 0,307757 | 0,075845 | DNA/RNA processing |
| ENSG00000171119 | PSMA2 | 0,539221 | 0,590382 | 0,710682 | DNA/RNA processing |
| ENSG00000165671 | PSMA6 | 0,188446 | -0,02429 | -0,07836 | DNA/RNA processing |
| ENSG00000088833 | PSMA7 | 0,111888 | 0,600691 | 0,916087 | DNA/RNA processing |
| ENSG00000117697 | PSMB1 | 0,124262 | -0,34835 | -0,36765 | DNA/RNA processing |
| ENSG00000107672 | PSMB10 | -0,65802 | -0,82371 | 0,048595 | DNA/RNA processing |
| ENSG00000037474 | PSMB2 | 1,87463 | 1,698844 | 1,47286 | DNA/RNA processing |
| ENSG00000130305 | PSMB3 | 1,202671 | 1,410919 | 1,311083 | DNA/RNA processing |
| ENSG00000125458 | PSMB4 | 1,850704 | 1,364658 | 1,8872 | DNA/RNA processing |
| ENSG00000076685 | PSMB6 | 1,125764 | 1,287579 | 0,717843 | DNA/RNA processing |
| ENSG00000168268 | PSMB7 | 1,006554 | 0,885053 | 0,62839 | DNA/RNA processing |
| ENSG00000205309 | PSMC2 | 0,54618 | 0,754569 | 0,539639 | DNA/RNA processing |
| ENSG00000065320 | PSMC3 | 0,937396 | 0,919445 | 0,428637 | DNA/RNA processing |
| ENSG00000196358 | PSMC4 | 0,912199 | 0,982313 | 0,745915 | DNA/RNA processing |
| ENSG00000013374 | PSMC5 | 0,397263 | 0,242087 | -0,23687 | DNA/RNA processing |
| ENSG00000095906 | PSMD1 | 0,44281 | 0,717374 | 0,525304 | DNA/RNA processing |
| ENSG00000104805 | PSMD11 | 0,557114 | 0,632246 | 0,252652 | DNA/RNA processing |
| ENSG00000070081 | PSMD3 | 1,03587 | 0,972807 | 0,752389 | DNA/RNA processing |
| ENSG00000069275 | PSMD7 | 1,412024 | 1,606718 | 1,649406 | DNA/RNA processing |
| ENSG00000090273 | PSMD8 | 1,95467 | 1,976999 | 1,544596 | DNA/RNA processing |
| ENSG00000015676 | PSME1 | 0,935881 | 0,9253 | 1,146034 | DNA/RNA processing |
| ENSG00000106268 | PSME2 | 1,077808 | 0,306033 | 0,230474 | DNA/RNA processing |
| ENSG00000168101 | PSME3 | 0,012481 | 0,146398 | 0,011534 | DNA/RNA processing |
| ENSG00000167005 | PSME4 | 0,778421 | 0,064242 | 1,319399 | DNA/RNA processing |
| ENSG00000149761 | PSMF1 | 1,470156 | 1,960602 | 2,593608 | DNA/RNA processing |
| ENSG00000132182 | PTBP1 | 3,159305 | 2,978308 | 2,641883 | DNA/RNA processing |
| ENSG00000132661 | PTMS | 0,743127 | 0,422914 | 1,148216 | DNA/RNA processing |
| ENSG00000104904 | PTPN1 | 0,802706 | 0,346428 | 0,952795 | DNA/RNA processing |
| ENSG00000060491 | PTTG1 | 0,045789 | 0,575961 | 0,57646 | DNA/RNA processing |
| ENSG00000102837 | PUF60 | 0,941452 | 0,899724 | 1,035907 | DNA/RNA processing |
| ENSG00000173391 | PUM1 | 2,054204 | 1,301807 | 1,72243 | DNA/RNA processing |
| ENSG00000169856 | PUM2 | 0,837201 | 0,821871 | 1,281174 | DNA/RNA processing |
| ENSG00000198836 | PURA | 0,711594 | 0,925191 | 0,953553 | DNA/RNA processing |
| ENSG00000130703 | QARS | 2,285705 | 1,838694 | 0,864163 | DNA/RNA processing |
| ENSG00000090060 | RAD21 | 0,668707 | 0,413374 | 1,062095 | DNA/RNA processing |
| ENSG00000116288 | RAD23B | 2,105615 | 1,766407 | 2,115192 | DNA/RNA processing |
| ENSG00000116288 | RAD50 | 0,073933 | -0,04903 | -0,49899 | DNA/RNA processing |
| ENSG00000116288 | RAD9A | 0,311428 | 0,347806 | 0,320269 | DNA/RNA processing |
| ENSG00000143799 | RALY | 1,1727 | 1,214904 | 1,725832 | DNA/RNA processing |
| ENSG00000059378 | RAN | 1,30845 | 1,361538 | 1,097822 | DNA/RNA processing |
| ENSG00000114054 | RARA | -0,5493 | -0,73365 | 0,167543 | DNA/RNA processing |
| ENSG00000160299 | RB1 | -0,06454 | 0,664042 | 0,555391 | DNA/RNA processing |
| ENSG00000173126 | RBBP4 | 2,244287 | 2,242883 | 2,152041 | DNA/RNA processing |
| ENSG00000160613 | RBBP7 | 2,635025 | 2,517298 | 2,37953 | DNA/RNA processing |
| ENSG00000141179 | RBL1 | -0,89773 | 0,080044 | -0,06357 | DNA/RNA processing |
| ENSG00000106244 | RBM10 | 0,802216 | 0,899623 | 0,825888 | DNA/RNA processing |
| ENSG00000188389 | RBM14 | 1,1973 | 1,135327 | 1,601977 | DNA/RNA processing |
| ENSG00000131828 | RBM22 | 0,084851 | -0,01885 | 0,964713 | DNA/RNA processing |
| ENSG00000155660 | RBM23 | -0,81095 | -0,10649 | 0,301199 | DNA/RNA processing |
| ENSG00000143870 | RBM25 | 1,252346 | 0,924001 | 1,01961 | DNA/RNA processing |
| ENSG00000120913 | RBM26 | 0,098806 | -0,27527 | 0,118921 | DNA/RNA processing |
| ENSG00000196923 | RBM27 | 0,387239 | 0,035452 | 0,298706 | DNA/RNA processing |
| ENSG00000140992 | RBM3 | 0,881197 | 0,673157 | 0,898003 | DNA/RNA processing |
| ENSG00000148459 | RBM38 | 0,737707 | 1,144354 | 3,071273 | DNA/RNA processing |
| ENSG00000179889 | RBM39 | 2,778095 | 2,259398 | 3,062343 | DNA/RNA processing |
| ENSG00000160209 | RBM4 | 1,325145 | 1,186446 | 1,603136 | DNA/RNA processing |
| ENSG00000165650 | RBM4B | 0,320806 | 0,052084 | 0,347467 | DNA/RNA processing |
| ENSG00000162366 | RBM5 | 1,427451 | 0,882217 | 1,747932 | DNA/RNA processing |
| ENSG00000089220 | RBM8A | 0,422869 | 0,646854 | 0,46506 | DNA/RNA processing |
| ENSG00000197329 | RBMS1 | -1,5143 | -1,29631 | -0,33977 | DNA/RNA processing |
| ENSG00000100029 | RC3H1 | 0,250453 | -0,09568 | 0,738682 | DNA/RNA processing |
| ENSG00000104883 | RCBTB1 | 0,047187 | -0,49025 | -0,34095 | DNA/RNA processing |
| ENSG00000215193 | RCL1 | 0,315959 | 0,898525 | 1,214042 | DNA/RNA processing |
| ENSG00000178921 | RCOR1 | 1,080475 | 1,080188 | 1,396195 | DNA/RNA processing |
| ENSG00000204220 | RDBP | 0,543857 | 0,293968 | 0,747106 | DNA/RNA processing |
| ENSG00000184207 | REPIN1 | 1,24707 | 1,065244 | 1,238398 | DNA/RNA processing |
| ENSG00000087157 | RERE | 0,427731 | 0,044864 | 0,867944 | DNA/RNA processing |
| ENSG00000173889 | RFC3 | -0,18372 | 0,108084 | -0,58681 | DNA/RNA processing |
| ENSG00000112511 | RFC4 | 0,404133 | 1,054761 | 0,423084 | DNA/RNA processing |
| ENSG00000197724 | RFWD3 | 1,810642 | 1,669455 | 1,39378 | DNA/RNA processing |
| ENSG00000172943 | RGS14 | -0,03185 | -0,28116 | 0,685156 | DNA/RNA processing |
| ENSG00000044446 | RGS2 | -0,60193 | -0,21023 | 2,301952 | DNA/RNA processing |
| ENSG00000105647 | RING1 | 0,182507 | 0,308409 | 0,073875 | DNA/RNA processing |
| ENSG00000196455 | RIOK3 | 0,643571 | 0,311775 | 2,679473 | DNA/RNA processing |
| ENSG00000085514 | RLF | 0,42609 | 0,016981 | 0,897386 | DNA/RNA processing |
| ENSG00000198355 | RNASE2 | 0,24297 | 1,652884 | 4,828243 | DNA/RNA processing |
| ENSG00000127445 | RNASE3 | -0,56037 | 0,800861 | 4,067563 | DNA/RNA processing |
| ENSG00000229359 | RNASEH2A | 0,43678 | 0,629564 | 0,19808 | DNA/RNA processing |
| ENSG00000186111 | RNASEH2C | 1,044826 | 1,535197 | 1,748527 | DNA/RNA processing |
| ENSG00000150867 | RNASEK | 1,643741 | 1,803095 | 2,918993 | DNA/RNA processing |
| ENSG00000110697 | RNASET2 | -0,94114 | -1,14315 | 0,052497 | DNA/RNA processing |
| ENSG00000225190 | RNF40 | 0,098022 | 0,435302 | 0,858258 | DNA/RNA processing |
| ENSG00000105355 | RNPC3 | 0,42475 | -0,30587 | 0,430146 | DNA/RNA processing |
| ENSG00000142731 | RNPS1 | 0,521442 | 0,345818 | -0,24204 | DNA/RNA processing |
| ENSG00000102007 | ROD1 | 0,586595 | 0,520093 | 1,180895 | DNA/RNA processing |
| ENSG00000101445 | RPA1 | -0,19424 | 0,072554 | -0,04809 | DNA/RNA processing |
| ENSG00000184203 | RPAIN | 0,725385 | 0,650659 | 0,717317 | DNA/RNA processing |
| ENSG00000115685 | RPL10 | 1,487297 | 1,970002 | 1,490544 | DNA/RNA processing |
| ENSG00000117751 | RPL10A | 2,461475 | 2,492308 | 1,332826 | DNA/RNA processing |
| ENSG00000113575 | RPL11 | 3,105635 | 2,891413 | 2,32817 | DNA/RNA processing |
| ENSG00000105568 | RPL12 | 0,561664 | 0,169866 | 0,682661 | DNA/RNA processing |
| ENSG00000066027 | RPL14 | 0,96644 | 0,986797 | 0,450059 | DNA/RNA processing |
| ENSG00000068971 | RPL15 | 1,718632 | 1,685297 | 1,366837 | DNA/RNA processing |
| ENSG00000078304 | RPL17 | -4,64583 | -4,56012 | -4,09235 | DNA/RNA processing |
| ENSG00000112640 | RPL18 | 5,756405 | 5,540078 | 5,479143 | DNA/RNA processing |
| ENSG00000154001 | RPL19 | 5,518985 | 5,240628 | 5,048943 | DNA/RNA processing |
| ENSG00000138814 | RPL24 | -4,47807 | -4,54231 | -4,09242 | DNA/RNA processing |
| ENSG00000221823 | RPL26 | 2,702075 | 2,119612 | 1,048884 | DNA/RNA processing |
| ENSG00000149923 | RPL27 | 1,697551 | 1,21335 | 1,251479 | DNA/RNA processing |
| ENSG00000163605 | RPL28 | -1,13406 | -1,97519 | -0,92747 | DNA/RNA processing |
| ENSG00000119414 | RPL29 | 1,144395 | 1,107067 | 0,67892 | DNA/RNA processing |
| ENSG00000100418 | RPL3 | 4,625325 | 4,309538 | 3,579053 | DNA/RNA processing |
| ENSG00000148840 | RPL30 | 3,722925 | 3,794073 | 3,925593 | DNA/RNA processing |
| ENSG00000131238 | RPL34 | 4,149665 | 3,731288 | 3,019623 | DNA/RNA processing |
| ENSG00000122490 | RPL35 | 4,726805 | 4,582188 | 4,211033 | DNA/RNA processing |
| ENSG00000040487 | RPL35A | 3,891405 | 3,419028 | 3,368583 | DNA/RNA processing |
| ENSG00000133246 | RPL36 | 2,630695 | 2,369633 | 2,142328 | DNA/RNA processing |
| ENSG00000197870 | RPL36AL | 0,675496 | 0,379087 | 0,220687 | DNA/RNA processing |
| ENSG00000198901 | RPL4 | 3,337385 | 3,551148 | 2,767173 | DNA/RNA processing |
| ENSG00000143294 | RPL8 | 4,548485 | 4,382173 | 4,077463 | DNA/RNA processing |
| ENSG00000130711 | RPLP0 | 3,595875 | 3,612738 | 3,235583 | DNA/RNA processing |
| ENSG00000112238 | RPLP1 | 4,403385 | 4,085078 | 4,061953 | DNA/RNA processing |
| ENSG00000116731 | RPLP2 | 4,015775 | 3,946113 | 3,912783 | DNA/RNA processing |
| ENSG00000124126 | RPS12 | 1,62024 | 1,481268 | 0,830617 | DNA/RNA processing |
| ENSG00000186652 | RPS13 | 1,295097 | 1,284299 | 0,874008 | DNA/RNA processing |
| ENSG00000156575 | RPS14 | 3,494535 | 3,160178 | 3,034343 | DNA/RNA processing |
| ENSG00000198056 | RPS15 | 3,214655 | 3,030423 | 2,597605 | DNA/RNA processing |
| ENSG00000111725 | RPS16 | 2,491935 | 2,571248 | 2,16348 | DNA/RNA processing |
| ENSG00000072062 | RPS19 | 3,741445 | 3,531678 | 3,290773 | DNA/RNA processing |
| ENSG00000188191 | RPS20 | 4,407815 | 4,049693 | 3,948773 | DNA/RNA processing |
| ENSG00000114302 | RPS23 | 1,199003 | 0,963922 | 0,696009 | DNA/RNA processing |
| ENSG00000005249 | RPS24 | 3,914635 | 3,290918 | 2,761503 | DNA/RNA processing |
| ENSG00000166501 | RPS25 | 4,701605 | 4,280983 | 4,036583 | DNA/RNA processing |
| ENSG00000071677 | RPS3 | 4,827735 | 4,821038 | 4,425813 | DNA/RNA processing |
| ENSG00000100462 | RPS4X | 3,631145 | 3,960428 | 2,431492 | DNA/RNA processing |
| ENSG00000171867 | RPS5 | 3,835105 | 3,580203 | 2,636483 | DNA/RNA processing |
| ENSG00000163421 | RPS6 | 4,170835 | 3,853483 | 3,175263 | DNA/RNA processing |
| ENSG00000105618 | RPS6KB2 | 0,290322 | 0,580262 | 0,611862 | DNA/RNA processing |
| ENSG00000134748 | RPS9 | 3,118245 | 3,159933 | 3,236063 | DNA/RNA processing |
| ENSG00000196504 | RQCD1 | 0,686862 | 0,902068 | 0,371883 | DNA/RNA processing |
| ENSG00000174231 | RRM1 | 0,468834 | 1,002793 | 0,266508 | DNA/RNA processing |
| ENSG00000174231 | RRM2 | -0,2397 | 0,030216 | 0,903801 | DNA/RNA processing |
| ENSG00000174231 | RRP1 | 0,150204 | 0,066463 | -0,20554 | DNA/RNA processing |
| ENSG00000174231 | RSL1D1 | 1,52164 | 1,488135 | 0,183905 | DNA/RNA processing |
| ENSG00000174231 | RSL24D1 | -0,01625 | -0,49224 | 0,11309 | DNA/RNA processing |
| ENSG00000174231 | RSRC1 | -0,09908 | -0,39206 | 0,163222 | DNA/RNA processing |
| ENSG00000174231 | RUVBL1 | 0,336223 | 0,059824 | -1,06815 | DNA/RNA processing |
| ENSG00000174231 | RUVBL2 | 0,826664 | 0,68257 | -0,29899 | DNA/RNA processing |
| ENSG00000174231 | RXRA | 0,056242 | 0,639451 | 1,75434 | DNA/RNA processing |
| ENSG00000174231 | RXRB | -0,02307 | 0,069153 | 0,570267 | DNA/RNA processing |
| ENSG00000174231 | SAFB | 0,949135 | 0,795929 | 1,23843 | DNA/RNA processing |
| ENSG00000167157 | SARS | 0,18609 | 0,464128 | 0,004618 | DNA/RNA processing |
| ENSG00000172382 | SART1 | 0,371706 | 0,966803 | 0,902506 | DNA/RNA processing |
| ENSG00000103355 | SART3 | -0,88312 | 0,273383 | -0,11609 | DNA/RNA processing |
| ENSG00000105227 | SATB1 | -0,21383 | -0,39214 | 0,530808 | DNA/RNA processing |
| ENSG00000197746 | SBDS | 1,003723 | 1,198771 | 1,802158 | DNA/RNA processing |
| ENSG00000159792 | SCAF1 | 0,015574 | -0,02538 | 0,411274 | DNA/RNA processing |
| ENSG00000126067 | SCNM1 | 0,709065 | 0,478235 | 0,780337 | DNA/RNA processing |
| ENSG00000108344 | SEC13 | 0,273425 | 0,154361 | 0,247931 | DNA/RNA processing |
| ENSG00000152229 | SECISBP2 | -0,31942 | -0,38262 | 0,520894 | DNA/RNA processing |
| ENSG00000188647 | SEH1L | 0,277839 | -0,14677 | -0,98123 | DNA/RNA processing |
| ENSG00000179295 | SERBP1 | 0,106989 | 0,122587 | -0,34795 | DNA/RNA processing |
| ENSG00000179950 | SET | 2,985345 | 2,864843 | 2,372636 | DNA/RNA processing |
| ENSG00000134644 | SETD1A | 0,964535 | 0,848702 | 0,852419 | DNA/RNA processing |
| ENSG00000055917 | SETD1B | 0,529735 | 0,301445 | 0,918862 | DNA/RNA processing |
| ENSG00000185129 | SETD2 | 1,452614 | 1,14276 | 1,656598 | DNA/RNA processing |
| ENSG00000177192 | SETX | -0,78873 | -0,33442 | 0,118763 | DNA/RNA processing |
| ENSG00000169972 | SF1 | 2,131502 | 1,822323 | 2,570668 | DNA/RNA processing |
| ENSG00000170234 | SF3A1 | 1,54758 | 1,583731 | 1,297771 | DNA/RNA processing |
| ENSG00000168297 | SF3A2 | 1,413832 | 1,7224 | 1,603834 | DNA/RNA processing |
| ENSG00000176894 | SF3A3 | 1,095349 | 1,085296 | 0,844426 | DNA/RNA processing |
| ENSG00000176894 | SF3B1 | 1,438786 | 1,206103 | 2,133884 | DNA/RNA processing |
| ENSG00000089159 | SF3B2 | 1,358624 | 1,209667 | 1,0358 | DNA/RNA processing |
| ENSG00000103490 | SF3B3 | 1,493885 | 1,834903 | 1,633354 | DNA/RNA processing |
| ENSG00000183010 | SF3B4 | 0,548228 | 0,451397 | 0,611418 | DNA/RNA processing |
| ENSG00000143811 | SF3B5 | 1,420207 | 1,362461 | 1,141036 | DNA/RNA processing |
| ENSG00000237575 | SFPQ | 3,264335 | 2,747863 | 2,901373 | DNA/RNA processing |
| ENSG00000132329 | SIN3A | 0,353628 | 0,43445 | 0,798914 | DNA/RNA processing |
| ENSG00000116473 | SIRT1 | -0,41654 | -0,40145 | 0,127446 | DNA/RNA processing |
| ENSG00000132359 | SIRT2 | -0,16778 | 0,04837 | 0,494568 | DNA/RNA processing |
| ENSG00000107263 | SIRT7 | -0,36497 | -0,34442 | 0,495263 | DNA/RNA processing |
| ENSG00000161847 | SLBP | 1,711505 | 1,733409 | 1,368874 | DNA/RNA processing |
| ENSG00000023287 | SLC11A1 | -1,02402 | -0,82734 | 0,740286 | DNA/RNA processing |
| ENSG00000170748 | SLC25A5 | 2,835955 | 2,654373 | 2,491889 | DNA/RNA processing |
| ENSG00000067560 | SMAD3 | 0,18712 | -0,24852 | -0,31358 | DNA/RNA processing |
| ENSG00000143878 | SMARCA2 | -0,03394 | -0,11977 | 0,052194 | DNA/RNA processing |
| ENSG00000139725 | SMARCA4 | 1,461782 | 1,14047 | 0,579124 | DNA/RNA processing |
| ENSG00000177105 | SMARCA5 | 0,38415 | 0,471203 | 0,551736 | DNA/RNA processing |
| ENSG00000168421 | SMARCB1 | 0,940201 | 0,737363 | 0,514198 | DNA/RNA processing |
| ENSG00000119729 | SMARCC1 | 1,338079 | 0,79167 | 0,04537 | DNA/RNA processing |
| ENSG00000126858 | SMARCC2 | 1,247125 | 1,22068 | 1,463963 | DNA/RNA processing |
| ENSG00000140983 | SMARCD1 | 0,9011 | 0,641456 | 0,428626 | DNA/RNA processing |
| ENSG00000104140 | SMARCD2 | 0,187025 | 0,206757 | 0,379172 | DNA/RNA processing |
| ENSG00000177963 | SMARCE1 | -3,34908 | -3,30754 | -2,97386 | DNA/RNA processing |
| ENSG00000164327 | SMC1A | 0,434868 | 1,02861 | 0,645721 | DNA/RNA processing |
| ENSG00000080345 | SMC3 | 0,682151 | 0,790777 | 0,702766 | DNA/RNA processing |
| ENSG00000167705 | SMC4 | 0,493703 | 0,938474 | 1,271807 | DNA/RNA processing |
| ENSG00000150977 | SMC5 | -0,06914 | -0,42246 | 0,006253 | DNA/RNA processing |
| ENSG00000100599 | SMCHD1 | 0,642271 | 0,331186 | 0,39341 | DNA/RNA processing |
| ENSG00000117000 | SMG1 | 0,054318 | 0,306982 | 1,222914 | DNA/RNA processing |
| ENSG00000153561 | SMG5 | 0,149309 | 0,011057 | 0,605691 | DNA/RNA processing |
| ENSG00000239907 | SMG7 | 1,553477 | 0,818052 | 1,197517 | DNA/RNA processing |
| ENSG00000199916 | SMNDC1 | -1,27574 | -0,85571 | -0,90243 | DNA/RNA processing |
| ENSG00000205937 | SNRNP200 | -2,78732 | -2,73924 | -2,29419 | DNA/RNA processing |
| ENSG00000205937 | SNRNP25 | 0,510066 | 0,619427 | 0,502952 | DNA/RNA processing |
| ENSG00000205937 | SNRNP27 | 0,034583 | -0,14845 | -0,02514 | DNA/RNA processing |
| ENSG00000067900 | SNRNP70 | 0,974545 | 1,137017 | 1,045451 | DNA/RNA processing |
| ENSG00000119314 | SNRPA | 1,630607 | 1,802723 | 1,100402 | DNA/RNA processing |
| ENSG00000067836 | SNRPB | 1,996898 | 1,708013 | 1,597054 | DNA/RNA processing |
| ENSG00000125995 | SNRPB2 | 0,024677 | 0,142861 | 0,205061 | DNA/RNA processing |
| ENSG00000229638 | SNRPD2 | 0,703798 | 0,508119 | 0,50522 | DNA/RNA processing |
| ENSG00000229932 | SNW1 | 0,065369 | -0,01165 | 0,078982 | DNA/RNA processing |
| ENSG00000197067 | SOD1 | 1,296588 | 1,526291 | 0,918932 | DNA/RNA processing |
| ENSG00000212695 | SON | 2,034219 | 1,811847 | 2,728343 | DNA/RNA processing |
| ENSG00000229945 | SOX4 | 0,427385 | -0,00458 | 0,633189 | DNA/RNA processing |
| ENSG00000244313 | SOX6 | -1,16388 | -0,69032 | 1,742015 | DNA/RNA processing |
| ENSG00000223803 | SOX9 | 0,785073 | 0,250017 | 0,964454 | DNA/RNA processing |
| ENSG00000237991 | SPRY1 | 0,886636 | -0,16244 | 0,0925 | DNA/RNA processing |
| ENSG00000073169 | SRCAP | 0,417738 | 0,460748 | 0,711733 | DNA/RNA processing |
| ENSG00000185290 | SRPK1 | 1,187043 | 0,926124 | 1,748019 | DNA/RNA processing |
| ENSG00000235605 | SRRM1 | 2,301265 | 1,961327 | 1,837114 | DNA/RNA processing |
| ENSG00000177776 | SRRM2 | 3,097585 | 2,885358 | 3,428073 | DNA/RNA processing |
| ENSG00000132383 | SRRT | 1,145313 | 1,132043 | 1,010217 | DNA/RNA processing |
| ENSG00000153574 | SSB | 2,325285 | 1,957668 | 0,951309 | DNA/RNA processing |
| ENSG00000147403 | SSBP1 | 0,281646 | -0,0955 | -0,59708 | DNA/RNA processing |
| ENSG00000236992 | SSRP1 | 1,239937 | 1,20071 | 0,313103 | DNA/RNA processing |
| ENSG00000174748 | SSU72 | -0,07154 | -0,12033 | 0,30885 | DNA/RNA processing |
| ENSG00000109475 | STRA13 | 0,329523 | 0,471589 | 0,584667 | DNA/RNA processing |
| ENSG00000231333 | STRAP | 0,148376 | -0,02998 | 0,087833 | DNA/RNA processing |
| ENSG00000130255 | STUB1 | 0,746764 | 0,50745 | 0,289763 | DNA/RNA processing |
| ENSG00000101413 | SUDS3 | -0,57982 | -0,86175 | 0,132737 | DNA/RNA processing |
| ENSG00000101278 | SUPT16H | 0,545125 | 0,343605 | -0,70652 | DNA/RNA processing |
| ENSG00000112306 | SUPT4H1 | 0,361335 | 0,31554 | 0,610793 | DNA/RNA processing |
| ENSG00000110700 | SUPT5H | -0,06452 | -0,11165 | 0,495091 | DNA/RNA processing |
| ENSG00000110700 | SUPT6H | 1,086916 | 1,042621 | 1,374307 | DNA/RNA processing |
| ENSG00000164587 | SUPV3L1 | 0,780034 | 0,403875 | -0,26269 | DNA/RNA processing |
| ENSG00000105372 | SUV420H2 | 0,26682 | 0,319064 | 1,028359 | DNA/RNA processing |
| ENSG00000187051 | SUZ12 | 1,433435 | 1,320896 | 1,413713 | DNA/RNA processing |
| ENSG00000138326 | SYMPK | 0,476638 | 0,187615 | 0,102208 | DNA/RNA processing |
| ENSG00000138326 | SYNCRIP | 0,646195 | 0,821851 | 0,804381 | DNA/RNA processing |
| ENSG00000178429 | TAF1 | 0,290288 | -0,03501 | 0,338649 | DNA/RNA processing |
| ENSG00000178429 | TAF10 | 2,620215 | 2,638593 | 3,301123 | DNA/RNA processing |
| ENSG00000083845 | TAF4 | 0,523393 | 0,623991 | 0,842096 | DNA/RNA processing |
| ENSG00000083845 | TAF4B | 0,196464 | -0,17656 | -1,9052 | DNA/RNA processing |
| ENSG00000083845 | TAF6 | 0,153309 | 0,347405 | -0,03817 | DNA/RNA processing |
| ENSG00000156990 | TAOK3 | -0,37664 | -0,27204 | 0,109349 | DNA/RNA processing |
| ENSG00000167325 | TARDBP | -1,86258 | -1,53924 | -0,62128 | DNA/RNA processing |
| ENSG00000186350 | TBL1XR1 | -0,72238 | -0,62353 | 0,391824 | DNA/RNA processing |
| ENSG00000204231 | TBL3 | 0,046667 | 0,015209 | -0,68579 | DNA/RNA processing |
| ENSG00000163785 | TBRG1 | 0,368097 | 0,319043 | 0,432619 | DNA/RNA processing |
| ENSG00000163221 | TCEB1 | -0,49513 | -0,00589 | 0,09383 | DNA/RNA processing |
| ENSG00000196154 | TCEB2 | -4,42111 | -4,31751 | -3,90297 | DNA/RNA processing |
| ENSG00000175467 | TFIP11 | 0,588362 | 0,245659 | 0,816425 | DNA/RNA processing |
| ENSG00000075856 | TH1L | 1,041061 | 0,773183 | -0,04522 | DNA/RNA processing |
| ENSG00000075856 | THOC1 | 0,545195 | 0,219667 | 0,075193 | DNA/RNA processing |
| ENSG00000075856 | THOC2 | 0,249897 | -0,09802 | -0,19833 | DNA/RNA processing |
| ENSG00000075856 | THOC4 | 2,279205 | 2,529663 | 1,831115 | DNA/RNA processing |
| ENSG00000075856 | THOC5 | 0,21578 | -0,36165 | -0,32644 | DNA/RNA processing |
| ENSG00000075856 | THOC6 | 1,779647 | 1,568566 | 1,050358 | DNA/RNA processing |
| ENSG00000156876 | THRA | 1,056008 | 1,027652 | 0,625284 | DNA/RNA processing |
| ENSG00000212137 | TK1 | 0,460047 | 0,775616 | 0,681457 | DNA/RNA processing |
| ENSG00000099194 | TLK1 | 0,30825 | 0,318421 | 0,352889 | DNA/RNA processing |
| ENSG00000107290 | TMX1 | -0,13274 | 0,160064 | -0,0811 | DNA/RNA processing |
| ENSG00000116560 | TNFRSF1B | -4,41574 | -4,20394 | -3,90817 | DNA/RNA processing |
| ENSG00000156304 | TNIP1 | -0,0454 | -0,15175 | 0,676165 | DNA/RNA processing |
| ENSG00000132424 | TNKS1BP1 | 0,093486 | 0,052582 | 0,010802 | DNA/RNA processing |
| ENSG00000139218 | TNPO1 | 0,209317 | 0,064318 | 0,605369 | DNA/RNA processing |
| ENSG00000115875 | TNRC6A | -0,04082 | 0,040289 | 0,229809 | DNA/RNA processing |
| ENSG00000061936 | TNRC6B | -0,75602 | -0,28118 | 1,536878 | DNA/RNA processing |
| ENSG00000126821 | TOP1 | 1,83039 | 1,847226 | 3,656643 | DNA/RNA processing |
| ENSG00000141258 | TOP1MT | 0,382329 | 0,630801 | 0,679374 | DNA/RNA processing |
| ENSG00000104969 | TOP2A | 0,154579 | 0,952675 | 0,521793 | DNA/RNA processing |
| ENSG00000111252 | TOP2B | 0,314098 | 0,10597 | 0,459049 | DNA/RNA processing |
| ENSG00000131171 | TOP3B | 0,106154 | -0,29762 | -0,31668 | DNA/RNA processing |
| ENSG00000142669 | TOPBP1 | 0,725061 | 0,769884 | 0,792081 | DNA/RNA processing |
| ENSG00000148341 | TP53 | 1,540879 | 1,614751 | 0,599299 | DNA/RNA processing |
| ENSG00000149577 | TPR | 0,333972 | 0,100931 | -0,06355 | DNA/RNA processing |
| ENSG00000169375 | TRA2A | 2,661255 | 2,086991 | 2,783503 | DNA/RNA processing |
| ENSG00000213445 | TRA2B | 2,393175 | 2,553093 | 2,744713 | DNA/RNA processing |
| ENSG00000113558 | TREX2 | -0,29465 | -0,77995 | -0,19783 | DNA/RNA processing |
| ENSG00000110911 | TRIM28 | 2,902785 | 2,430658 | 1,880387 | DNA/RNA processing |
| ENSG00000144136 | TRMT2A | 0,38377 | 0,268377 | 0,42144 | DNA/RNA processing |
| ENSG00000155287 | TRRAP | 0,381798 | 0,182706 | 0,459796 | DNA/RNA processing |
| ENSG00000197119 | TRUB2 | 0,292044 | 0,554246 | -0,70558 | DNA/RNA processing |
| ENSG00000075415 | TSC1 | -0,86913 | -0,52824 | 0,56489 | DNA/RNA processing |
| ENSG00000075415 | TSEN54 | 0,107282 | -0,01942 | 0,154395 | DNA/RNA processing |
| ENSG00000005022 | TSPYL1 | 1,146844 | 1,031174 | 1,379159 | DNA/RNA processing |
| ENSG00000129353 | TUT1 | 4,826555 | 4,644743 | 4,279473 | DNA/RNA processing |
| ENSG00000139514 | TXNL4A | 0,151989 | 0,38374 | 0,006207 | DNA/RNA processing |
| ENSG00000109062 | TYMP | 0,007595 | 0,017003 | 0,639131 | DNA/RNA processing |
| ENSG00000197818 | TYMS | 2,221712 | 2,384658 | 1,917101 | DNA/RNA processing |
| ENSG00000124107 | U2AF1 | 2,415555 | 2,468663 | 2,462182 | DNA/RNA processing |
| ENSG00000137776 | U2AF2 | 1,852136 | 1,669508 | 1,574521 | DNA/RNA processing |
| ENSG00000139613 | UBA52 | 2,730905 | 2,673263 | 2,979033 | DNA/RNA processing |
| ENSG00000198887 | UBB | 4,588115 | 4,657338 | 5,213323 | DNA/RNA processing |
| ENSG00000196787 | UBC | 5,120515 | 4,831483 | 5,873363 | DNA/RNA processing |
| ENSG00000177427 | UBE2A | -0,23556 | -0,28984 | 0,566122 | DNA/RNA processing |
| ENSG00000176994 | UBE2B | -0,97728 | -0,80408 | 0,421321 | DNA/RNA processing |
| ENSG00000119953 | UBE2I | 0,084686 | 0,163065 | 0,436247 | DNA/RNA processing |
| ENSG00000099940 | UBE2T | -0,51747 | 0,25398 | -0,54736 | DNA/RNA processing |
| ENSG00000206823 | UBN1 | 0,162186 | 0,434895 | 1,221722 | DNA/RNA processing |
| ENSG00000200792 | UBR2 | -1,07334 | -0,4199 | 1,816422 | DNA/RNA processing |
| ENSG00000209582 | UBTF | 1,609617 | 1,82581 | 1,297227 | DNA/RNA processing |
| ENSG00000209042 | UHRF1 | 1,40165 | 1,391586 | 1,514453 | DNA/RNA processing |
| ENSG00000207445 | UIMC1 | -0,53609 | -0,67196 | 0,427801 | DNA/RNA processing |
| ENSG00000201067 | UNG | 0,611778 | 0,294293 | -0,91791 | DNA/RNA processing |
| ENSG00000238311 | UPF1 | 0,807637 | 0,808779 | 1,174599 | DNA/RNA processing |
| ENSG00000239035 | UPF3A | 1,192158 | 1,30125 | 1,445956 | DNA/RNA processing |
| ENSG00000144028 | USP10 | -0,10712 | 0,111895 | 0,094565 | DNA/RNA processing |
| ENSG00000144028 | USP21 | 0,40135 | 0,406746 | 0,070516 | DNA/RNA processing |
| ENSG00000144028 | USP22 | -0,27465 | -1,27138 | -1,22546 | DNA/RNA processing |
| ENSG00000144028 | USP3 | 0,654207 | 0,389072 | 1,47259 | DNA/RNA processing |
| ENSG00000125870 | USP39 | 0,447752 | -0,1363 | -0,47156 | DNA/RNA processing |
| ENSG00000115234 | USP9X | 0,497592 | 0,343836 | 1,70628 | DNA/RNA processing |
| ENSG00000120451 | UTP18 | 0,297724 | 0,059641 | 0,141317 | DNA/RNA processing |
| ENSG00000157734 | UVRAG | -0,13816 | -0,35387 | 0,348342 | DNA/RNA processing |
| ENSG00000004777 | UXT | 0,391326 | 0,678029 | 0,483544 | DNA/RNA processing |
| ENSG00000159140 | VCP | 1,44419 | 1,6991 | 1,847994 | DNA/RNA processing |
| ENSG00000115904 | VEGFA | -0,97489 | -0,62991 | 0,206981 | DNA/RNA processing |
| ENSG00000197912 | VPS72 | 0,16026 | 0,157139 | -0,29759 | DNA/RNA processing |
| ENSG00000162032 | WAC | 0,904623 | 0,973415 | 1,903193 | DNA/RNA processing |
| ENSG00000070182 | WARS | -0,21576 | -0,17484 | 0,482813 | DNA/RNA processing |
| ENSG00000161011 | WBP11 | 2,122365 | 1,667909 | 0,964703 | DNA/RNA processing |
| ENSG00000222106 | WDR5 | 0,471123 | 0,600387 | -0,14446 | DNA/RNA processing |
| ENSG00000153037 | WHSC1 | -0,08639 | 0,430356 | 0,339302 | DNA/RNA processing |
| ENSG00000174780 | WHSC1L1 | -0,07433 | 0,212964 | 0,782505 | DNA/RNA processing |
| ENSG00000096063 | WHSC2 | 0,640128 | 0,610785 | 0,503731 | DNA/RNA processing |
| ENSG00000138385 | WRN | 0,833839 | 0,453612 | 0,861978 | DNA/RNA processing |
| ENSG00000106028 | WRNIP1 | 0,114494 | 0,067407 | 0,574313 | DNA/RNA processing |
| ENSG00000130511 | WTAP | 0,669471 | 0,377456 | 1,367572 | DNA/RNA processing |
| ENSG00000176101 | XAB2 | 0,854791 | 0,857073 | 1,062186 | DNA/RNA processing |
| ENSG00000180879 | XPO1 | 0,25478 | 0,333269 | -0,03788 | DNA/RNA processing |
| ENSG00000173465 | XPO7 | -0,19121 | 0,241507 | 3,870203 | DNA/RNA processing |
| ENSG00000160075 | XRCC1 | 0,698226 | 0,68563 | 0,505084 | DNA/RNA processing |
| ENSG00000117155 | XRCC2 | -0,10796 | 0,015485 | -0,81193 | DNA/RNA processing |
| ENSG00000008513 | XRCC5 | 1,24585 | 1,335415 | 1,139314 | DNA/RNA processing |
| ENSG00000157350 | XRCC6 | 1,477271 | 1,314818 | 0,555402 | DNA/RNA processing |
| ENSG00000073849 | XRN1 | -0,58898 | -0,45857 | 1,02221 | DNA/RNA processing |
| ENSG00000136738 | YARS | 0,372833 | 0,028811 | -0,18955 | DNA/RNA processing |
| ENSG00000131748 | YBX2 | 1,241686 | 0,846435 | 1,244113 | DNA/RNA processing |
| ENSG00000084090 | YEATS4 | 0,058309 | -0,05382 | -0,16529 | DNA/RNA processing |
| ENSG00000166888 | YTHDC1 | 0,986949 | 0,358144 | 1,574138 | DNA/RNA processing |
| ENSG00000167323 | YWHAB | 1,294485 | 1,656333 | 2,169249 | DNA/RNA processing |
| ENSG00000072786 | YWHAG | 1,456389 | 1,315218 | 0,667055 | DNA/RNA processing |
| ENSG00000102572 | YY1 | 4,231875 | 4,072103 | 4,573533 | DNA/RNA processing |
| ENSG00000135604 | ZC3H14 | 0,014053 | -0,10283 | -1,1399 | DNA/RNA processing |
| ENSG00000103496 | ZC3H3 | -0,49735 | -0,53477 | 0,210678 | DNA/RNA processing |
| ENSG00000129103 | ZCCHC8 | 0,270036 | 0,172277 | 1,818488 | DNA/RNA processing |
| ENSG00000165025 | ZFC3H1 | -0,13861 | -0,52526 | 0,96139 | DNA/RNA processing |
| ENSG00000125755 | ZFP36 | 0,582181 | 0,943508 | 2,116536 | DNA/RNA processing |
| ENSG00000135316 | ZFP36L1 | 1,300436 | 1,293788 | 0,581293 | DNA/RNA processing |
| ENSG00000131018 | ZFP36L2 | 3,339505 | 3,307623 | 3,501953 | DNA/RNA processing |
| ENSG00000142765 | ZFYVE26 | 0,059081 | -0,12748 | 0,309734 | DNA/RNA processing |
| ENSG00000169762 | ZNF384 | 1,083698 | 0,937547 | 1,534134 | DNA/RNA processing |
| ENSG00000154582 | ZRANB2 | 1,143092 | 0,267616 | 0,370784 | DNA/RNA processing |
| ENSG00000201492 | AATF | 0,649128 | 0,261702 | 0,564653 | Cycle cell |
| ENSG00000241883 | ACHE | -0,74433 | -0,4186 | 0,234686 | Cycle cell |
| ENSG00000241433 | ACTA1 | 0,630012 | 0,456847 | 0,885873 | Cycle cell |
| ENSG00000173517 | ACTR1A | 0,582576 | 0,449459 | 1,247861 | Cycle cell |
| ENSG00000240619 | AES | 1,165322 | 1,109215 | 1,260814 | Cycle cell |
| ENSG00000100813 | AIF1 | -1,52785 | -1,04022 | 1,092618 | Cycle cell |
| ENSG00000151726 | AKAP8 | 0,882251 | 0,829082 | 1,51954 | Cycle cell |
| ENSG00000181786 | AKT1 | 1,744731 | 1,7493 | 1,843017 | Cycle cell |
| ENSG00000240166 | ANAPC2 | -0,16706 | -0,2488 | 0,117943 | Cycle cell |
| ENSG00000241301 | ANAPC5 | -0,47021 | -0,35678 | -1,15428 | Cycle cell |
| ENSG00000240700 | ANAPC7 | 0,301374 | -0,06037 | -0,46282 | Cycle cell |
| ENSG00000241802 | ANKRD54 | 0,027689 | 0,131285 | 0,135697 | Cycle cell |
| ENSG00000243028 | ANLN | -0,4997 | -0,01922 | 0,794225 | Cycle cell |
| ENSG00000242275 | ANO10 | -1,20191 | -0,71536 | 0,328976 | Cycle cell |
| ENSG00000243173 | ANXA1 | 1,757694 | 1,972089 | 5,086203 | Cycle cell |
| ENSG00000243471 | ANXA11 | 0,75075 | 0,524443 | 2,471211 | Cycle cell |
| ENSG00000244066 | ANXA7 | -0,08201 | -0,68386 | 0,36166 | Cycle cell |
| ENSG00000089053 | APP | -0,82592 | -0,80844 | 0,718683 | Cycle cell |
| ENSG00000089053 | APPL2 | -0,13525 | 0,046139 | 0,675988 | Cycle cell |
| ENSG00000198944 | ARHGEF1 | 0,074227 | 0,083886 | 1,413473 | Cycle cell |
| ENSG00000064999 | ARHGEF2 | 0,277696 | 0,558861 | 1,424291 | Cycle cell |
| ENSG00000122359 | ARL2 | 1,685634 | 1,665557 | 0,532962 | Cycle cell |
| ENSG00000131471 | ARL8A | 0,907524 | 0,82394 | 2,372664 | Cycle cell |
| ENSG00000182351 | ARL8B | 0,096891 | 0,009751 | 0,718073 | Cycle cell |
| ENSG00000100280 | ARPP19 | 0,118305 | -0,47745 | 0,341403 | Cycle cell |
| ENSG00000042753 | ARX | -0,03572 | -0,26133 | 0,083124 | Cycle cell |
| ENSG00000134884 | ATP6AP1 | 1,140433 | 1,119582 | 2,146997 | Cycle cell |
| ENSG00000225485 | ATP6V0E1 | -0,53148 | 0,671495 | 1,105413 | Cycle cell |
| ENSG00000145819 | ATP6V0E2 | -0,12928 | -0,28042 | 0,094801 | Cycle cell |
| ENSG00000141522 | ATP7A | -1,39929 | -0,60355 | 0,265894 | Cycle cell |
| ENSG00000116584 | ATXN10 | 0,360566 | 0,598452 | -0,01924 | Cycle cell |
| ENSG00000117713 | ATXN2 | 0,067204 | 0,523497 | 0,317282 | Cycle cell |
| ENSG00000189079 | ATXN7 | 1,942572 | 1,491191 | 2,090981 | Cycle cell |
| ENSG00000177479 | AURKB | 0,883562 | 1,249247 | 1,1735 | Cycle cell |
| ENSG00000213465 | AXIN1 | -0,2756 | -0,5623 | 0,152226 | Cycle cell |
| ENSG00000102931 | AZI1 | -0,10637 | -0,31777 | 0,261272 | Cycle cell |
| ENSG00000128989 | BANP | -0,56474 | -0,6331 | 0,081038 | Cycle cell |
| ENSG00000137486 | BAP1 | 0,836435 | 1,078119 | 1,187603 | Cycle cell |
| ENSG00000170653 | BCR | 0,041941 | -0,42103 | 0,59849 | Cycle cell |
| ENSG00000130734 | BID | 0,845608 | 0,802377 | 1,525496 | Cycle cell |
| ENSG00000138363 | BIN3 | -0,7854 | -0,6886 | 0,348232 | Cycle cell |
| ENSG00000177556 | BLCAP | 0,708741 | 0,685111 | 1,143647 | Cycle cell |
| ENSG00000129244 | BLZF1 | -0,49527 | -0,47223 | 0,88866 | Cycle cell |
| ENSG00000165629 | BOP1 | 0,458613 | 0,908028 | 0,438028 | Cycle cell |
| ENSG00000167283 | BRE | 0,04066 | -0,05278 | 0,07321 | Cycle cell |
| ENSG00000113732 | BTRC | -1,24373 | -0,92234 | 0,321769 | Cycle cell |
| ENSG00000113732 | BUB1B | 0,44896 | 0,577264 | 0,444271 | Cycle cell |
| ENSG00000178999 | C12orf32 | 0,320322 | 1,084746 | 0,973331 | Cycle cell |
| ENSG00000166710 | C13orf15 | 0,266126 | 1,141394 | 3,306793 | Cycle cell |
| ENSG00000204469 | C15orf42 | -0,26337 | 0,112428 | -0,5515 | Cycle cell |
| ENSG00000110696 | C20orf20 | -0,13133 | -0,3353 | 0,167553 | Cycle cell |
| ENSG00000089916 | C2orf29 | 0,414198 | 0,467798 | 0,208522 | Cycle cell |
| ENSG00000103121 | C6orf108 | 0,892512 | 0,822074 | 0,118298 | Cycle cell |
| ENSG00000123144 | CAB39 | 1,572712 | 1,228239 | 1,560049 | Cycle cell |
| ENSG00000183019 | CALR | 2,767475 | 2,733513 | 2,998293 | Cycle cell |
| ENSG00000119280 | CAPRIN2 | -0,40968 | 0,102781 | -0,4884 | Cycle cell |
| ENSG00000162585 | CASC5 | -0,49207 | -0,17928 | 0,174972 | Cycle cell |
| ENSG00000177410 | CCAR1 | 2,308055 | 2,078237 | 1,506589 | Cycle cell |
| ENSG00000100220 | CCDC85B | 0,583093 | 0,129048 | 0,401612 | Cycle cell |
| ENSG00000100220 | CCNA2 | 0,839693 | 1,269929 | 1,012097 | Cycle cell |
| ENSG00000100220 | CCNB1 | -0,62496 | 0,029889 | -0,56356 | Cycle cell |
| ENSG00000100220 | CCNB2 | 0,494833 | 1,209043 | 0,520615 | Cycle cell |
| ENSG00000183172 | CCND2 | 2,315445 | 1,99457 | 0,914143 | Cycle cell |
| ENSG00000100364 | CCND3 | 1,097833 | 1,711995 | 3,270123 | Cycle cell |
| ENSG00000172375 | CCNDBP1 | 0,150055 | 0,519934 | 2,539591 | Cycle cell |
| ENSG00000168014 | CCNE1 | -0,5174 | 0,437797 | -0,26777 | Cycle cell |
| ENSG00000183186 | CCNG1 | 0,888812 | 0,571336 | 0,085769 | Cycle cell |
| ENSG00000115649 | CCNK | 0,971889 | 0,629851 | 1,326223 | Cycle cell |
| ENSG00000115649 | CCNT1 | 0,836382 | 0,391109 | 0,684281 | Cycle cell |
| ENSG00000115649 | CCNT2 | 0,32514 | -0,04134 | 0,542711 | Cycle cell |
| ENSG00000115649 | CCNY | 0,12496 | -0,03917 | 0,63023 | Cycle cell |
| ENSG00000183624 | CD2AP | -0,55671 | -0,4937 | 0,142684 | Cycle cell |
| ENSG00000181744 | CD320 | 0,981604 | 0,825953 | 0,350368 | Cycle cell |
| ENSG00000112308 | CD74 | 2,334515 | 1,855249 | 3,374823 | Cycle cell |
| ENSG00000146576 | CD81 | 1,633828 | 1,366139 | 1,403704 | Cycle cell |
| ENSG00000146540 | CDC123 | 0,078803 | 0,197753 | 0,213903 | Cycle cell |
| ENSG00000188186 | CDC14A | -0,30077 | -0,60597 | 0,341875 | Cycle cell |
| ENSG00000182307 | CDC20 | 0,69799 | 1,304721 | 0,300037 | Cycle cell |
| ENSG00000130193 | CDC25B | -0,5142 | -0,34155 | 0,787994 | Cycle cell |
| ENSG00000241852 | CDC27 | 0,266341 | 0,621744 | 1,759775 | Cycle cell |
| ENSG00000137038 | CDC34 | 0,438722 | 0,795453 | 2,33751 | Cycle cell |
| ENSG00000148362 | CDC40 | -0,33267 | -0,3186 | 0,306402 | Cycle cell |
| ENSG00000171159 | CDC42 | 0,822311 | 0,676139 | 1,412197 | Cycle cell |
| ENSG00000104267 | CDC5L | 0,588667 | 0,464377 | 0,266589 | Cycle cell |
| ENSG00000135932 | CDC73 | 1,306844 | 0,986858 | 1,56656 | Cycle cell |
| ENSG00000099991 | CDCA5 | -0,2434 | 0,035243 | -0,54627 | Cycle cell |
| ENSG00000167791 | CDCA7L | -0,69463 | -0,39653 | 0,581452 | Cycle cell |
| ENSG00000142408 | CDCA8 | 0,072781 | 0,213654 | 0,207552 | Cycle cell |
| ENSG00000012822 | CDK10 | 0,254799 | 0,361538 | 0,052587 | Cycle cell |
| ENSG00000136436 | CDK2 | 0,31564 | 0,90757 | 0,262399 | Cycle cell |
| ENSG00000138172 | CDK2AP1 | 2,387445 | 1,856849 | 1,588838 | Cycle cell |
| ENSG00000143933 | CDK4 | 2,003161 | 2,230078 | 0,758548 | Cycle cell |
| ENSG00000160014 | CDK5R1 | -0,42972 | -0,41698 | 0,071149 | Cycle cell |
| ENSG00000130643 | CDK5RAP2 | 0,161564 | 0,247605 | 0,023911 | Cycle cell |
| ENSG00000163888 | CDK6 | 1,513185 | 1,222627 | -0,72135 | Cycle cell |
| ENSG00000110931 | CDK9 | 1,054163 | 0,974569 | 2,007733 | Cycle cell |
| ENSG00000164047 | CDKN1B | 0,448234 | 0,29123 | 2,116854 | Cycle cell |
| ENSG00000108509 | CDKN1C | 0,747693 | 0,591506 | 1,019472 | Cycle cell |
| ENSG00000127022 | CDKN2D | -0,07326 | 0,164599 | 1,268876 | Cycle cell |
| ENSG00000127022 | CDT1 | 0,521919 | 0,990134 | 0,275687 | Cycle cell |
| ENSG00000127022 | CDV3 | 2,376745 | 2,15489 | 2,574864 | Cycle cell |
| ENSG00000127022 | CENPC1 | 0,640076 | 0,3144 | 0,802159 | Cycle cell |
| ENSG00000127022 | CENPF | 0,675305 | 1,061737 | 0,850759 | Cycle cell |
| ENSG00000127022 | CENPM | 0,576594 | 0,804088 | 1,082407 | Cycle cell |
| ENSG00000127022 | CENPO | -0,36805 | 0,140569 | -0,14177 | Cycle cell |
| ENSG00000127022 | CENPT | 0,268294 | 0,434834 | 0,521115 | Cycle cell |
| ENSG00000127022 | CENPV | 0,432841 | 0,370907 | -0,93183 | Cycle cell |
| ENSG00000127022 | CEP192 | -0,35997 | -0,12446 | 0,305818 | Cycle cell |
| ENSG00000131236 | CETN2 | 0,064315 | 0,535359 | 0,363212 | Cycle cell |
| ENSG00000126247 | CHAF1A | 1,039531 | 1,39772 | 0,842912 | Cycle cell |
| ENSG00000126247 | CHEK1 | -0,28637 | 0,122963 | -0,49661 | Cycle cell |
| ENSG00000135387 | CHFR | -0,39174 | -0,29283 | 0,574574 | Cycle cell |
| ENSG00000187796 | CHMP1A | 0,445823 | 0,501365 | 1,044277 | Cycle cell |
| ENSG00000134905 | CHPT1 | 1,978573 | 2,109681 | 2,563682 | Cycle cell |
| ENSG00000137812 | CHST11 | 1,924226 | 2,130609 | 2,658533 | Cycle cell |
| ENSG00000105879 | CHTF18 | 0,416869 | 0,793516 | 0,819414 | Cycle cell |
| ENSG00000108468 | CHTF8 | 0,620224 | 0,666312 | 0,343511 | Cycle cell |
| ENSG00000060339 | CIT | 0,448096 | 0,71722 | 0,536321 | Cycle cell |
| ENSG00000176476 | CITED2 | 1,887047 | 1,831663 | 2,996803 | Cycle cell |
| ENSG00000160799 | CKAP2 | 0,81978 | 0,979541 | 1,233522 | Cycle cell |
| ENSG00000100147 | CKAP5 | 0,735059 | 0,730227 | 0,354834 | Cycle cell |
| ENSG00000108588 | CKS1B | 0,327981 | 0,731744 | 0,769248 | Cycle cell |
| ENSG00000114654 | CKS2 | 2,068615 | 2,252691 | 1,565831 | Cycle cell |
| ENSG00000108296 | CLASP1 | 0,419837 | 0,329002 | 1,093833 | Cycle cell |
| ENSG00000166946 | CLK1 | 1,318394 | 1,052675 | 2,209405 | Cycle cell |
| ENSG00000135083 | CLN3 | -0,32254 | -0,39398 | 0,111373 | Cycle cell |
| ENSG00000090061 | CLN6 | 0,797756 | 0,747702 | 0,728321 | Cycle cell |
| ENSG00000163660 | CLN8 | -0,80243 | -1,14025 | 0,080366 | Cycle cell |
| ENSG00000126353 | CLTC | 1,588052 | 1,855365 | 2,076391 | Cycle cell |
| ENSG00000151014 | CLTCL1 | -1,56052 | -1,44192 | 0,163322 | Cycle cell |
| ENSG00000177697 | CNN2 | 0,873537 | 0,635483 | 2,832943 | Cycle cell |
| ENSG00000110848 | COL4A3BP | -0,90944 | -0,89378 | 0,444541 | Cycle cell |
| ENSG00000129757 | CREBBP | 0,872804 | 1,050262 | 2,530726 | Cycle cell |
| ENSG00000129355 | CREG1 | 0,93165 | 0,768254 | 1,830748 | Cycle cell |
| ENSG00000183307 | CRYAA | 0,727103 | 0,200046 | 0,738935 | Cycle cell |
| ENSG00000100162 | CSE1L | 0,476326 | 0,600227 | -0,08929 | Cycle cell |
| ENSG00000138092 | CSF1 | 1,419153 | 0,523943 | -0,32511 | Cycle cell |
| ENSG00000135837 | CSGALNACT1 | -2,59927 | -2,68883 | 0,009936 | Cycle cell |
| ENSG00000172757 | CSNK1D | 1,708628 | 1,451727 | 2,009397 | Cycle cell |
| ENSG00000172757 | CSNK1E | 0,365984 | -0,16636 | 0,052773 | Cycle cell |
| ENSG00000172757 | CSNK2A1 | 1,078124 | 1,043054 | 0,786296 | Cycle cell |
| ENSG00000172757 | CSNK2A2 | 1,451911 | 1,349229 | 1,20129 | Cycle cell |
| ENSG00000173575 | CTBP1 | 1,575714 | 1,70446 | 1,794402 | Cycle cell |
| ENSG00000177830 | CTF1 | 0,238277 | 0,175981 | 0,669258 | Cycle cell |
| ENSG00000133063 | CTNNA1 | 0,106453 | 0,30814 | 0,110241 | Cycle cell |
| ENSG00000171310 | CTSD | 1,420984 | 2,131276 | 3,721303 | Cycle cell |
| ENSG00000145354 | CUL3 | 0,976359 | 0,747572 | 1,376645 | Cycle cell |
| ENSG00000122966 | CUL4A | 0,210534 | 0,156687 | 0,868145 | Cycle cell |
| ENSG00000182372 | DBP | -0,30902 | -0,33988 | 0,278445 | Cycle cell |
| ENSG00000162368 | DCTN1 | 0,362768 | -0,08654 | -0,0064 | Cycle cell |
| ENSG00000134326 | DCTN2 | 0,510754 | 0,647256 | 0,395698 | Cycle cell |
| ENSG00000091317 | DCTN3 | -0,04326 | 0,54067 | 1,011584 | Cycle cell |
| ENSG00000111596 | DDB1 | 1,383941 | 1,299063 | 1,871604 | Cycle cell |
| ENSG00000155508 | DDIT3 | 0,021351 | -0,49467 | 1,091603 | Cycle cell |
| ENSG00000092758 | DDX5 | 3,311945 | 3,450608 | 4,125823 | Cycle cell |
| ENSG00000126267 | DGKD | -0,69779 | -0,43658 | 1,61257 | Cycle cell |
| ENSG00000115944 | DGKZ | 0,072067 | -0,21111 | 0,4071 | Cycle cell |
| ENSG00000149532 | DKC1 | 0,966492 | 0,628528 | -0,17157 | Cycle cell |
| ENSG00000110090 | DLG1 | -0,15683 | 0,0433 | 0,736947 | Cycle cell |
| ENSG00000205560 | DLGAP5 | -0,04896 | 0,680383 | 0,1312 | Cycle cell |
| ENSG00000176390 | DNAJC2 | 0,641007 | -0,06697 | -0,15489 | Cycle cell |
| ENSG00000170275 | DNAJC5 | -0,18735 | 0,127643 | 0,909833 | Cycle cell |
| ENSG00000160202 | DNM2 | 1,979092 | 1,938032 | 2,46743 | Cycle cell |
| ENSG00000100058 | DNMT1 | 2,130996 | 2,048527 | 1,403691 | Cycle cell |
| ENSG00000141551 | DPH1 | 0,159576 | 0,201273 | 0,394266 | Cycle cell |
| ENSG00000160213 | DSN1 | -0,3074 | 0,420639 | 0,147981 | Cycle cell |
| ENSG00000236002 | DTL | 1,217166 | 1,667 | 0,58631 | Cycle cell |
| ENSG00000230673 | DUSP1 | 2,877685 | 2,997073 | 3,321373 | Cycle cell |
| ENSG00000164733 | DYNC1H1 | 1,295057 | 0,942178 | 0,659223 | Cycle cell |
| ENSG00000196188 | DYNLL1 | -0,09546 | 0,07288 | -0,82347 | Cycle cell |
| ENSG00000142544 | E2F1 | 1,398456 | 1,577908 | 1,057178 | Cycle cell |
| ENSG00000178531 | E2F2 | -1,12901 | -0,46658 | 1,597879 | Cycle cell |
| ENSG00000180891 | E2F4 | 2,014344 | 2,590673 | 2,647203 | Cycle cell |
| ENSG00000107874 | E4F1 | -0,11428 | -0,04007 | 0,226671 | Cycle cell |
| ENSG00000115165 | EGLN2 | -0,62446 | -0,44675 | 0,061637 | Cycle cell |
| ENSG00000129562 | EID2 | 1,013155 | 0,731901 | 1,326997 | Cycle cell |
| ENSG00000130311 | EIF4EBP1 | 1,316937 | 1,083514 | 0,734489 | Cycle cell |
| ENSG00000167986 | EIF4G1 | 0,726335 | 0,756476 | 0,477349 | Cycle cell |
| ENSG00000100523 | EIF4G2 | 3,718245 | 3,110903 | 3,578943 | Cycle cell |
| ENSG00000215301 | ELN | -0,0956 | -0,19178 | 0,262088 | Cycle cell |
| ENSG00000108654 | EML4 | -0,37726 | -0,6064 | 1,100151 | Cycle cell |
| ENSG00000108654 | EMP3 | 1,345605 | 1,06453 | 1,623218 | Cycle cell |
| ENSG00000108654 | ENG | 1,990459 | 2,254978 | 0,202363 | Cycle cell |
| ENSG00000108654 | EP300 | 0,856371 | 0,804801 | 2,852863 | Cycle cell |
| ENSG00000110367 | EPC1 | 0,469547 | -0,14314 | 0,180502 | Cycle cell |
| ENSG00000140995 | EPS15 | 1,35944 | 1,073918 | 1,092304 | Cycle cell |
| ENSG00000174485 | ERBB2IP | -0,07076 | 0,118329 | 0,71929 | Cycle cell |
| ENSG00000198837 | ERCC1 | 0,079642 | 0,030975 | -0,06454 | Cycle cell |
| ENSG00000184014 | ERCC2 | -0,18662 | -0,07666 | 0,218708 | Cycle cell |
| ENSG00000136986 | ERCC3 | 0,278499 | -0,13482 | -0,61082 | Cycle cell |
| ENSG00000070413 | ERH | 0,264705 | 0,543934 | 0,53197 | Cycle cell |
| ENSG00000162496 | ESPL1 | -0,89472 | -0,09935 | 0,322015 | Cycle cell |
| ENSG00000132153 | ESRRA | 0,230251 | 0,033677 | 0,640174 | Cycle cell |
| ENSG00000087470 | FAF1 | 0,34645 | -0,11893 | 0,314086 | Cycle cell |
| ENSG00000147155 | FAM83D | 1,070223 | 1,269213 | 1,725622 | Cycle cell |
| ENSG00000038358 | FANCA | 0,138764 | 0,055469 | -0,29043 | Cycle cell |
| ENSG00000134109 | FANCD2 | 0,072922 | 0,11226 | -0,21902 | Cycle cell |
| ENSG00000116406 | FANCI | 0,527141 | 0,933534 | 0,338429 | Cycle cell |
| ENSG00000149547 | FBXO5 | -1,06601 | -0,36114 | 0,269795 | Cycle cell |
| ENSG00000176396 | FBXO7 | 2,872925 | 2,831493 | 3,658773 | Cycle cell |
| ENSG00000100353 | FEN1 | 0,583033 | 1,080505 | 0,425803 | Cycle cell |
| ENSG00000100353 | FES | -0,98478 | -0,85539 | 0,102736 | Cycle cell |
| ENSG00000100353 | FGFRL1 | 0,43533 | 0,342499 | 0,572103 | Cycle cell |
| ENSG00000110321 | FOSL2 | -0,8296 | -0,45886 | 0,814802 | Cycle cell |
| ENSG00000110321 | FOXC2 | 0,648688 | 0,237607 | 0,586436 | Cycle cell |
| ENSG00000110321 | FOXM1 | 0,550772 | 0,794923 | 0,236571 | Cycle cell |
| ENSG00000110321 | FOXN3 | 0,947458 | 0,687607 | 1,472977 | Cycle cell |
| ENSG00000110321 | FOXO1 | 0,97159 | 0,668033 | 1,43042 | Cycle cell |
| ENSG00000110321 | FOXO3 | 0,632537 | 0,465749 | 1,572566 | Cycle cell |
| ENSG00000110321 | FOXO4 | -0,60288 | 0,014947 | 1,52324 | Cycle cell |
| ENSG00000242372 | FRAT2 | 0,62112 | 0,573367 | 0,925731 | Cycle cell |
| ENSG00000066044 | FSCN1 | 1,364417 | 0,647479 | 0,425136 | Cycle cell |
| ENSG00000105656 | FTL | 2,889705 | 3,057693 | 4,182573 | Cycle cell |
| ENSG00000062598 | FURIN | 0,89902 | 0,551051 | 1,992159 | Cycle cell |
| ENSG00000049540 | FUS | 2,666515 | 2,580758 | 2,56078 | Cycle cell |
| ENSG00000074800 | FZR1 | 0,251097 | 0,416797 | 2,04755 | Cycle cell |
| ENSG00000132199 | G0S2 | 0,624473 | 0,749917 | 2,39821 | Cycle cell |
| ENSG00000100393 | GAB1 | 0,423881 | 0,832003 | 0,893612 | Cycle cell |
| ENSG00000072134 | GADD45A | -0,75855 | -0,57711 | 1,2342 | Cycle cell |
| ENSG00000049283 | GADD45B | -0,02859 | -0,20438 | 0,605152 | Cycle cell |
| ENSG00000187266 | GADD45G | -1,38471 | -1,0714 | 0,359524 | Cycle cell |
| ENSG00000085832 | GADD45GIP1 | 0,894735 | 0,739421 | 0,734392 | Cycle cell |
| ENSG00000127527 | GAK | 0,546918 | 0,720896 | 1,359314 | Cycle cell |
| ENSG00000100632 | GARS | 1,137741 | 0,923392 | 0,242223 | Cycle cell |
| ENSG00000100632 | GAS2L1 | 0,680658 | 0,710899 | 1,08775 | Cycle cell |
| ENSG00000100632 | GATAD2A | 1,429858 | 1,114907 | 1,453137 | Cycle cell |
| ENSG00000107566 | GBA | -0,76884 | -0,52919 | 0,668551 | Cycle cell |
| ENSG00000023318 | GBX2 | 0,033126 | -0,2152 | 0,105628 | Cycle cell |
| ENSG00000117036 | GFI1B | 0,729887 | 1,387495 | 1,068634 | Cycle cell |
| ENSG00000182473 | GIGYF2 | 0,100938 | 0,179945 | 0,673032 | Cycle cell |
| ENSG00000171311 | GINS2 | 0,61086 | 0,528901 | -0,02189 | Cycle cell |
| ENSG00000178896 | GJC2 | 0,278968 | 0,068524 | 0,647391 | Cycle cell |
| ENSG00000153443 | GLUL | 1,597591 | 1,770431 | 2,423323 | Cycle cell |
| ENSG00000232150 | GNAI2 | 1,530631 | 1,474584 | 3,364273 | Cycle cell |
| ENSG00000121104 | GNB1 | 2,701015 | 2,793803 | 3,123313 | Cycle cell |
| ENSG00000100376 | GNB2L1 | 4,866295 | 4,529793 | 4,363483 | Cycle cell |
| ENSG00000048828 | GNG2 | -0,94799 | -1,14524 | 0,508175 | Cycle cell |
| ENSG00000124019 | GNL3 | 0,427395 | 0,142827 | -1,1646 | Cycle cell |
| ENSG00000144567 | GOLPH3 | 0,917335 | 0,965946 | 1,253794 | Cycle cell |
| ENSG00000172775 | GPR132 | -2,13072 | -1,91024 | 0,092139 | Cycle cell |
| ENSG00000071859 | GPS1 | 0,984084 | 1,042273 | 0,88984 | Cycle cell |
| ENSG00000189319 | GPS2 | 0,508589 | 0,250237 | 0,979796 | Cycle cell |
| ENSG00000179115 | GSPT1 | 1,304549 | 1,536653 | 1,992368 | Cycle cell |
| ENSG00000157107 | H2AFX | 1,438229 | 1,669707 | 1,594408 | Cycle cell |
| ENSG00000000938 | HAUS4 | 0,571827 | 0,787013 | 0,041878 | Cycle cell |
| ENSG00000137460 | HAUS6 | 0,570041 | -0,31454 | -0,50617 | Cycle cell |
| ENSG00000172500 | HBP1 | -0,73395 | -0,88316 | 0,701242 | Cycle cell |
| ENSG00000132589 | HDAC3 | 1,193067 | 1,621457 | 1,08631 | Cycle cell |
| ENSG00000167363 | HDGF | 2,943285 | 3,172653 | 3,520583 | Cycle cell |
| ENSG00000171051 | HIP1 | -2,22226 | -2,47624 | -1,57939 | Cycle cell |
| ENSG00000181274 | HIPK2 | -0,21543 | -0,39928 | 1,541122 | Cycle cell |
| ENSG00000170296 | HJURP | 0,053264 | 0,585955 | 1,103712 | Cycle cell |
| ENSG00000130005 | HMG20B | 0,660359 | 0,481476 | 0,686047 | Cycle cell |
| ENSG00000005955 | HRAS | 0,691947 | 0,46913 | 0,939411 | Cycle cell |
| ENSG00000130309 | HSD17B10 | 0,074858 | 0,38529 | 0,138968 | Cycle cell |
| ENSG00000105373 | HSD3B7 | 0,193973 | 0,243914 | 0,119894 | Cycle cell |
| ENSG00000196743 | HSP90AA1 | 2,814285 | 3,197468 | 2,508311 | Cycle cell |
| ENSG00000137198 | HSPA8 | 4,062365 | 4,496873 | 3,797413 | Cycle cell |
| ENSG00000146535 | HSPB1 | 1,756866 | 2,218378 | 1,186864 | Cycle cell |
| ENSG00000060558 | HSPD1 | 1,251503 | 1,363158 | -0,16699 | Cycle cell |
| ENSG00000156052 | HTRA1 | -0,35553 | -0,53125 | 0,265224 | Cycle cell |
| ENSG00000078369 | HTRA3 | -0,19831 | -0,29221 | 0,644043 | Cycle cell |
| ENSG00000172354 | HTT | 0,112716 | -0,16812 | 0,343767 | Cycle cell |
| ENSG00000170075 | IGFBP1 | 0,041842 | -0,44127 | 0,197876 | Cycle cell |
| ENSG00000183134 | IGFBP4 | 0,470154 | 0,134242 | 0,767319 | Cycle cell |
| ENSG00000205336 | IGFBP7 | 0,130081 | -0,33973 | 0,619849 | Cycle cell |
| ENSG00000181656 | IGFBPL1 | 0,113193 | -0,06892 | 0,550154 | Cycle cell |
| ENSG00000121957 | IKZF1 | 0,584283 | 1,121804 | 1,857 | Cycle cell |
| ENSG00000213654 | IL15RA | 0,505036 | 0,429329 | 0,760207 | Cycle cell |
| ENSG00000197245 | IL4R | -0,9388 | -0,98937 | 0,920008 | Cycle cell |
| ENSG00000235581 | IL6R | -1,20592 | -1,03196 | 0,094357 | Cycle cell |
| ENSG00000228963 | IL8 | 0,47711 | 0,622657 | 3,389943 | Cycle cell |
| ENSG00000105723 | ILF3 | 2,028506 | 1,789046 | 1,523699 | Cycle cell |
| ENSG00000082701 | ILK | -0,11427 | 0,504137 | 1,331286 | Cycle cell |
| ENSG00000134184 | INCENP | 0,007328 | 0,106435 | 0,227353 | Cycle cell |
| ENSG00000169840 | INO80 | 0,099762 | -0,19205 | 0,095676 | Cycle cell |
| ENSG00000115207 | INSIG1 | -0,66406 | -0,60706 | 0,267349 | Cycle cell |
| ENSG00000104812 | IRF2 | -0,64838 | -0,35083 | 0,371708 | Cycle cell |
| ENSG00000163041 | ISG20 | -1,05202 | -1,03469 | 0,218363 | Cycle cell |
| ENSG00000188375 | ITCH | 0,327569 | -0,15833 | 0,42053 | Cycle cell |
| ENSG00000092036 | ITGB1 | 1,598123 | 1,290912 | 1,042398 | Cycle cell |
| ENSG00000136929 | JMY | -0,52612 | -0,9664 | 0,243982 | Cycle cell |
| ENSG00000188290 | JTB | 0,483804 | -0,17624 | 0,234892 | Cycle cell |
| ENSG00000144485 | JUN | 0,378418 | 0,385137 | 1,170919 | Cycle cell |
| ENSG00000186834 | JUNB | 1,779187 | 2,149203 | 3,675993 | Cycle cell |
| ENSG00000156875 | KAT2A | 1,357626 | 0,842454 | 0,031347 | Cycle cell |
| ENSG00000148110 | KAT2B | 0,078964 | 0,711434 | 2,296135 | Cycle cell |
| ENSG00000177374 | KATNB1 | -0,00085 | 0,090193 | 0,105423 | Cycle cell |
| ENSG00000169567 | KCNC3 | -0,35817 | -0,50426 | 0,04467 | Cycle cell |
| ENSG00000196176 | KHDRBS1 | 2,958055 | 2,828553 | 2,734203 | Cycle cell |
| ENSG00000184270 | KIAA0174 | 1,688762 | 1,22031 | 1,93523 | Cycle cell |
| ENSG00000234745 | KIAA0415 | -0,44809 | -0,49021 | 0,146843 | Cycle cell |
| ENSG00000137309 | KIAA1109 | -0,57738 | -0,74723 | 0,525865 | Cycle cell |
| ENSG00000177733 | KIF13A | 0,701635 | -0,24969 | -0,17587 | Cycle cell |
| ENSG00000135486 | KIF18B | -0,20799 | 0,223576 | -0,09416 | Cycle cell |
| ENSG00000135486 | KIF22 | 1,748175 | 1,971055 | 1,812531 | Cycle cell |
| ENSG00000092199 | KIFC1 | -0,23431 | 0,173563 | 0,265662 | Cycle cell |
| ENSG00000092199 | KIT | 2,165599 | 0,463205 | -0,75781 | Cycle cell |
| ENSG00000092199 | KLF10 | 0,362196 | 0,278023 | 0,614082 | Cycle cell |
| ENSG00000138668 | KLHL21 | -1,11575 | -0,73201 | 0,072946 | Cycle cell |
| ENSG00000126945 | KPNA2 | 1,391184 | 1,682661 | 1,623127 | Cycle cell |
| ENSG00000165119 | KRT18 | 0,299882 | -0,08821 | 0,512881 | Cycle cell |
| ENSG00000100099 | LATS2 | -0,20217 | -0,32941 | 0,150006 | Cycle cell |
| ENSG00000198189 | LEFTY1 | -0,17495 | -0,48408 | 0,230196 | Cycle cell |
| ENSG00000108785 | LEFTY2 | -0,39045 | -0,25801 | 0,431145 | Cycle cell |
| ENSG00000096384 | LIG1 | 0,954649 | 1,11835 | 0,734964 | Cycle cell |
| ENSG00000096384 | LIG3 | 0,199345 | -0,23005 | 0,176955 | Cycle cell |
| ENSG00000106211 | LIMS1 | 0,716371 | 0,811275 | 1,210759 | Cycle cell |
| ENSG00000115607 | LRWD1 | 0,19992 | 0,221732 | 0,499938 | Cycle cell |
| ENSG00000178035 | LZTS2 | 0,019478 | 0,070116 | 0,121579 | Cycle cell |
| ENSG00000203485 | MAD1L1 | 0,383448 | 0,210683 | 0,332212 | Cycle cell |
| ENSG00000163083 | MAD2L1BP | -0,15385 | -0,57038 | 0,523434 | Cycle cell |
| ENSG00000128908 | MAD2L2 | 0,240379 | 0,571026 | 0,613666 | Cycle cell |
| ENSG00000169592 | MADD | 0,141432 | -0,14142 | 0,368801 | Cycle cell |
| ENSG00000204084 | MAEA | 0,240075 | 0,650175 | 1,189599 | Cycle cell |
| ENSG00000165458 | MAFG | -0,39637 | -0,2941 | 0,93369 | Cycle cell |
| ENSG00000198700 | MAP2K1 | 0,256413 | 0,11135 | 0,759268 | Cycle cell |
| ENSG00000144711 | MAP3K11 | -0,04336 | -0,07834 | 0,790828 | Cycle cell |
| ENSG00000168310 | MAP3K8 | -0,69898 | -0,75612 | 0,385625 | Cycle cell |
| ENSG00000185507 | MAPK1 | 0,453091 | 1,116785 | 2,384759 | Cycle cell |
| ENSG00000185950 | MAPK3 | -0,15226 | -0,02108 | 1,102017 | Cycle cell |
| ENSG00000136003 | MAPKAPK2 | 0,110108 | -0,088 | 1,366485 | Cycle cell |
| ENSG00000105655 | MAPRE1 | 1,360084 | 1,019508 | 1,407106 | Cycle cell |
| ENSG00000136156 | MASTL | -0,11281 | 0,042623 | 0,527508 | Cycle cell |
| ENSG00000210176 | MBD2 | 0,676431 | 0,906711 | 1,587851 | Cycle cell |
| ENSG00000198695 | MCM10 | -0,3977 | 0,234222 | -0,88282 | Cycle cell |
| ENSG00000198840 | MCM2 | 1,726608 | 1,892582 | 1,283077 | Cycle cell |
| ENSG00000162434 | MCM3 | 1,76279 | 1,877501 | 0,7237 | Cycle cell |
| ENSG00000096968 | MCM4 | 1,050541 | 1,134409 | 0,262602 | Cycle cell |
| ENSG00000008083 | MCM5 | 2,212511 | 2,08434 | 1,189466 | Cycle cell |
| ENSG00000153814 | MCM6 | -0,22456 | 0,041178 | -0,31483 | Cycle cell |
| ENSG00000140044 | MCM7 | 2,715955 | 2,846253 | 2,204454 | Cycle cell |
| ENSG00000006459 | MCM8 | -0,05878 | 0,345163 | -0,03538 | Cycle cell |
| ENSG00000161677 | MDM2 | 0,195265 | 0,104547 | 0,823845 | Cycle cell |
| ENSG00000092051 | MDM4 | 0,953677 | 0,203691 | 0,821608 | Cycle cell |
| ENSG00000143543 | MED1 | -0,13325 | 0,145915 | 0,031828 | Cycle cell |
| ENSG00000069424 | MFN2 | 0,554636 | 0,611255 | 1,824882 | Cycle cell |
| ENSG00000147050 | MKI67 | 0,562582 | 1,183645 | 1,7273 | Cycle cell |
| ENSG00000121774 | MLF1IP | 1,083946 | 0,786839 | -0,01848 | Cycle cell |
| ENSG00000121774 | MLH1 | 0,02107 | 0,370605 | 0,223641 | Cycle cell |
| ENSG00000121774 | MLL5 | 0,801038 | 0,893521 | 2,054167 | Cycle cell |
| ENSG00000131149 | MMP9 | 0,619537 | 1,557727 | 4,460793 | Cycle cell |
| ENSG00000100647 | MNT | -0,06838 | -0,05576 | 0,719809 | Cycle cell |
| ENSG00000135709 | MORC3 | -0,99877 | -0,77622 | 0,684701 | Cycle cell |
| ENSG00000132361 | MORF4L1 | 1,56765 | 1,481451 | 1,92564 | Cycle cell |
| ENSG00000126775 | MORF4L2 | 0,066653 | 0,038354 | -0,19345 | Cycle cell |
| ENSG00000122203 | MPHOSPH9 | 1,032449 | 0,847575 | 1,068189 | Cycle cell |
| ENSG00000157404 | MRPL41 | 0,621883 | 0,493538 | 0,652395 | Cycle cell |
| ENSG00000205445 | MT3 | 0,5679 | 0,185986 | 0,811964 | Cycle cell |
| ENSG00000167613 | MTOR | 0,033583 | 0,142173 | 0,194849 | Cycle cell |
| ENSG00000185896 | MTPN | 1,894147 | 2,113356 | 3,126403 | Cycle cell |
| ENSG00000068697 | MUC12 | -1,07686 | -1,1678 | -0,06716 | Cycle cell |
| ENSG00000107929 | MXD1 | 1,332422 | 0,976585 | 2,444546 | Cycle cell |
| ENSG00000188779 | MYC | 2,287605 | 1,903519 | 0,140081 | Cycle cell |
| ENSG00000172155 | MYD88 | 0,191777 | 0,35892 | 1,574111 | Cycle cell |
| ENSG00000186226 | MYH10 | 0,4479 | 0,678829 | -0,34274 | Cycle cell |
| ENSG00000104826 | NANOS3 | 0,371363 | 0,17527 | 0,806903 | Cycle cell |
| ENSG00000187116 | NASP | 0,878185 | 0,5217 | -0,23268 | Cycle cell |
| ENSG00000220008 | NCAPD2 | 1,028115 | 1,770305 | 1,197993 | Cycle cell |
| ENSG00000189067 | NCAPD3 | -0,13631 | 0,552287 | 0,492545 | Cycle cell |
| ENSG00000152022 | NCAPG | 0,344239 | 0,626094 | 0,582989 | Cycle cell |
| ENSG00000236850 | NCAPG2 | -0,3774 | 0,150706 | 0,286512 | Cycle cell |
| ENSG00000139233 | NCAPH | 0,030882 | 0,549714 | 0,27579 | Cycle cell |
| ENSG00000087253 | NDE1 | 0,112334 | 0,024451 | 0,893592 | Cycle cell |
| ENSG00000123684 | NDEL1 | 1,047764 | 0,723226 | 2,430355 | Cycle cell |
| ENSG00000145012 | NDOR1 | 0,091543 | 0,06775 | 0,187632 | Cycle cell |
| ENSG00000170860 | NET1 | 1,319872 | 0,526525 | 0,154022 | Cycle cell |
| ENSG00000160285 | NFATC1 | 0,036857 | -0,27535 | 0,098853 | Cycle cell |
| ENSG00000147507 | NFKBIA | 2,952725 | 2,516024 | 3,730603 | Cycle cell |
| ENSG00000197063 | NIPBL | -0,05781 | -0,20292 | 0,620525 | Cycle cell |
| ENSG00000198909 | NOLC1 | 1,279901 | 0,98016 | -0,78025 | Cycle cell |
| ENSG00000071054 | NOP58 | 1,913384 | 1,47059 | -0,05317 | Cycle cell |
| ENSG00000102882 | NPAT | -0,41994 | 0,125013 | 0,145608 | Cycle cell |
| ENSG00000101367 | NPM1 | -1,54685 | -1,66528 | -1,30858 | Cycle cell |
| ENSG00000075413 | NR3C1 | -0,57173 | -0,24055 | 0,660994 | Cycle cell |
| ENSG00000120539 | NRD1 | 0,596015 | 1,08122 | 1,171698 | Cycle cell |
| ENSG00000134046 | NSL1 | -0,0553 | 0,063443 | 0,287795 | Cycle cell |
| ENSG00000104738 | NUDC | 1,032259 | 0,969506 | 0,017219 | Cycle cell |
| ENSG00000110492 | NUMA1 | 0,762907 | 0,696635 | 0,988625 | Cycle cell |
| ENSG00000169057 | NUP214 | 1,36269 | 1,173023 | 1,312334 | Cycle cell |
| ENSG00000125686 | NUP43 | 0,188264 | 0,330487 | -0,80236 | Cycle cell |
| ENSG00000123066 | NUP85 | 1,226301 | 1,041264 | 0,595631 | Cycle cell |
| ENSG00000175221 | NUP98 | 1,152875 | 0,943034 | 1,949397 | Cycle cell |
| ENSG00000112282 | NUSAP1 | 0,839493 | 1,229289 | 1,525649 | Cycle cell |
| ENSG00000146834 | ODF2 | -0,20516 | 0,209703 | 0,306925 | Cycle cell |
| ENSG00000106484 | OGFR | 0,333803 | 0,104756 | 0,732459 | Cycle cell |
| ENSG00000165792 | OLR1 | -2,41673 | -2,06071 | 1,037174 | Cycle cell |
| ENSG00000176624 | OPTN | -0,44314 | -0,42736 | 0,213045 | Cycle cell |
| ENSG00000143198 | OSGIN1 | 0,156381 | -0,33077 | 0,166705 | Cycle cell |
| ENSG00000196588 | PA2G4 | 2,849175 | 2,657703 | 2,401057 | Cycle cell |
| ENSG00000171843 | PAK4 | 0,089085 | -0,03057 | 0,114496 | Cycle cell |
| ENSG00000108788 | PANK2 | 0,202423 | 0,135091 | 0,616327 | Cycle cell |
| ENSG00000130675 | PARP4 | 0,69475 | 0,302467 | 1,069393 | Cycle cell |
| ENSG00000159256 | PBRM1 | -0,64186 | -0,05189 | 0,301232 | Cycle cell |
| ENSG00000103152 | PCM1 | 1,388155 | 0,71978 | 1,073566 | Cycle cell |
| ENSG00000186732 | PCNA | 0,942073 | 1,465449 | 0,787595 | Cycle cell |
| ENSG00000133030 | PCNP | 1,389523 | 1,489372 | 1,798138 | Cycle cell |
| ENSG00000128309 | PCNT | 0,088698 | 0,054556 | 1,180306 | Cycle cell |
| ENSG00000158042 | PDAP1 | 0,541391 | 0,312799 | 0,377 | Cycle cell |
| ENSG00000112651 | PDCD4 | 1,025643 | 1,48013 | 1,439636 | Cycle cell |
| ENSG00000197345 | PDCD6IP | 0,579414 | 0,522718 | 0,505793 | Cycle cell |
| ENSG00000105364 | PDS5A | 1,072297 | 1,142745 | 1,109913 | Cycle cell |
| ENSG00000136522 | PDXK | -1,02813 | -1,11812 | 0,228612 | Cycle cell |
| ENSG00000182180 | PEMT | 0,192465 | 0,031291 | -0,04906 | Cycle cell |
| ENSG00000122140 | PES1 | 0,228878 | 0,332694 | -0,66016 | Cycle cell |
| ENSG00000074071 | PFDN1 | 0,617238 | 0,433099 | 0,370309 | Cycle cell |
| ENSG00000163719 | PHF8 | -0,25185 | -0,34876 | 0,556576 | Cycle cell |
| ENSG00000100330 | PHIP | 1,271597 | 0,624388 | 1,085994 | Cycle cell |
| ENSG00000214114 | PIK3C3 | -0,56003 | -0,38169 | 0,180755 | Cycle cell |
| ENSG00000141140 | PIM1 | 0,278452 | 1,143462 | 4,226263 | Cycle cell |
| ENSG00000142347 | PIM2 | -0,29292 | -0,38477 | 1,310457 | Cycle cell |
| ENSG00000136286 | PIM3 | -0,19739 | -0,47558 | 0,224977 | Cycle cell |
| ENSG00000099331 | PIN1 | 0,641048 | 0,509241 | 0,673606 | Cycle cell |
| ENSG00000136504 | PKD1 | -0,54903 | -0,28785 | 0,011165 | Cycle cell |
| ENSG00000136504 | PKM2 | -0,7698 | -0,03269 | 2,284863 | Cycle cell |
| ENSG00000136504 | PKMYT1 | 0,1847 | 0,611707 | 0,658407 | Cycle cell |
| ENSG00000134440 | PLK1 | 1,012142 | 1,740949 | 1,367717 | Cycle cell |
| ENSG00000134440 | PLK3 | -0,95772 | -1,429 | 0,148211 | Cycle cell |
| ENSG00000134440 | PLK4 | 0,251506 | 0,123679 | -0,25045 | Cycle cell |
| ENSG00000135372 | PMF1 | 0,58979 | 0,43563 | 0,496512 | Cycle cell |
| ENSG00000121579 | PML | 0,500705 | 0,126281 | 0,002846 | Cycle cell |
| ENSG00000146918 | PNPLA6 | -0,47859 | -0,04811 | 1,238433 | Cycle cell |
| ENSG00000116701 | POGZ | 0,346915 | 0,407142 | 0,938526 | Cycle cell |
| ENSG00000100365 | POLA2 | 0,41011 | 0,268199 | -0,07907 | Cycle cell |
| ENSG00000071051 | POLB | -0,5859 | -0,75435 | 0,060368 | Cycle cell |
| ENSG00000123338 | POLD1 | 0,363616 | 0,536308 | -0,20143 | Cycle cell |
| ENSG00000115053 | POLD2 | 1,442955 | 1,335425 | 0,354134 | Cycle cell |
| ENSG00000125912 | POLD4 | -0,30289 | -0,18546 | 1,017191 | Cycle cell |
| ENSG00000141027 | POLE | 0,840543 | 0,26824 | 0,674455 | Cycle cell |
| ENSG00000196498 | POLE2 | -0,14333 | 0,186395 | -1,15304 | Cycle cell |
| ENSG00000089818 | PPM1A | 0,492014 | -0,3291 | 0,608946 | Cycle cell |
| ENSG00000157191 | PPM1D | -0,07193 | -0,47157 | 0,031728 | Cycle cell |
| ENSG00000117691 | PPM1G | 1,22749 | 1,116597 | 0,65784 | Cycle cell |
| ENSG00000186575 | PPP1CA | 1,511916 | 1,739941 | 1,925138 | Cycle cell |
| ENSG00000235568 | PPP1CB | 0,475246 | 0,508929 | 1,449426 | Cycle cell |
| ENSG00000102908 | PPP1CC | 1,734339 | 1,59313 | 1,576234 | Cycle cell |
| ENSG00000176953 | PPP1R12A | 1,314625 | 1,052684 | 1,848561 | Cycle cell |
| ENSG00000123405 | PPP1R15A | 1,501226 | 1,882269 | 3,280573 | Cycle cell |
| ENSG00000077150 | PPP1R8 | 0,088874 | 0,007073 | -0,57869 | Cycle cell |
| ENSG00000104825 | PPP2CA | 0,801002 | 0,89556 | 1,927892 | Cycle cell |
| ENSG00000167604 | PPP2R1A | 1,738742 | 1,342914 | 1,426215 | Cycle cell |
| ENSG00000151092 | PPP3CA | -0,45153 | -0,43139 | 0,117643 | Cycle cell |
| ENSG00000132603 | PPP6C | 0,978 | 0,823862 | 1,48943 | Cycle cell |
| ENSG00000105374 | PPT1 | 0,174459 | 0,441306 | 1,340084 | Cycle cell |
| ENSG00000140853 | PRC1 | 1,662457 | 1,979723 | 1,811924 | Cycle cell |
| ENSG00000123009 | PRCC | 0,817591 | 0,781647 | 0,90231 | Cycle cell |
| ENSG00000153406 | PRDX1 | 0,780713 | 0,678133 | 0,432038 | Cycle cell |
| ENSG00000147140 | PRIM1 | -0,73233 | 0,073357 | -0,51139 | Cycle cell |
| ENSG00000147140 | PRKAB1 | 1,5712 | 1,843947 | 1,63375 | Cycle cell |
| ENSG00000147140 | PRKACA | -0,42229 | -0,41008 | 0,281813 | Cycle cell |
| ENSG00000147140 | PRKAG1 | -0,00852 | 0,651373 | 0,306271 | Cycle cell |
| ENSG00000147140 | PRKAR2B | 1,79859 | 1,576574 | 0,774779 | Cycle cell |
| ENSG00000147140 | PRKD2 | -1,08162 | -0,62886 | 0,538332 | Cycle cell |
| ENSG00000182117 | PRMT5 | 0,731802 | 1,091293 | -0,33837 | Cycle cell |
| ENSG00000087269 | PRNP | 0,713078 | 0,902652 | 0,975146 | Cycle cell |
| ENSG00000111641 | PROK2 | -0,45496 | -0,31675 | 1,875448 | Cycle cell |
| ENSG00000149308 | PRPF40A | 0,902609 | 0,680701 | 0,585361 | Cycle cell |
| ENSG00000107833 | PRR5 | 0,099237 | 0,072434 | 1,116283 | Cycle cell |
| ENSG00000154146 | PSMA1 | 0,511412 | 0,307757 | 0,075845 | Cycle cell |
| ENSG00000171119 | PSMA2 | 0,539221 | 0,590382 | 0,710682 | Cycle cell |
| ENSG00000165671 | PSMA6 | 0,188446 | -0,02429 | -0,07836 | Cycle cell |
| ENSG00000088833 | PSMA7 | 0,111888 | 0,600691 | 0,916087 | Cycle cell |
| ENSG00000117697 | PSMB1 | 0,124262 | -0,34835 | -0,36765 | Cycle cell |
| ENSG00000107672 | PSMB10 | -0,65802 | -0,82371 | 0,048595 | Cycle cell |
| ENSG00000037474 | PSMB2 | 1,87463 | 1,698844 | 1,47286 | Cycle cell |
| ENSG00000130305 | PSMB3 | 1,202671 | 1,410919 | 1,311083 | Cycle cell |
| ENSG00000125458 | PSMB4 | 1,850704 | 1,364658 | 1,8872 | Cycle cell |
| ENSG00000076685 | PSMB6 | 1,125764 | 1,287579 | 0,717843 | Cycle cell |
| ENSG00000168268 | PSMB7 | 1,006554 | 0,885053 | 0,62839 | Cycle cell |
| ENSG00000205309 | PSMC2 | 0,54618 | 0,754569 | 0,539639 | Cycle cell |
| ENSG00000065320 | PSMC3 | 0,937396 | 0,919445 | 0,428637 | Cycle cell |
| ENSG00000196358 | PSMC4 | 0,912199 | 0,982313 | 0,745915 | Cycle cell |
| ENSG00000013374 | PSMC5 | 0,397263 | 0,242087 | -0,23687 | Cycle cell |
| ENSG00000095906 | PSMD1 | 0,44281 | 0,717374 | 0,525304 | Cycle cell |
| ENSG00000104805 | PSMD11 | 0,557114 | 0,632246 | 0,252652 | Cycle cell |
| ENSG00000070081 | PSMD3 | 1,03587 | 0,972807 | 0,752389 | Cycle cell |
| ENSG00000069275 | PSMD7 | 1,412024 | 1,606718 | 1,649406 | Cycle cell |
| ENSG00000090273 | PSMD8 | 1,95467 | 1,976999 | 1,544596 | Cycle cell |
| ENSG00000015676 | PSME1 | 0,935881 | 0,9253 | 1,146034 | Cycle cell |
| ENSG00000106268 | PSME2 | 1,077808 | 0,306033 | 0,230474 | Cycle cell |
| ENSG00000168101 | PSME3 | 0,012481 | 0,146398 | 0,011534 | Cycle cell |
| ENSG00000167005 | PSME4 | 0,778421 | 0,064242 | 1,319399 | Cycle cell |
| ENSG00000149761 | PSMF1 | 1,470156 | 1,960602 | 2,593608 | Cycle cell |
| ENSG00000083635 | PSMG2 | 1,336038 | 0,922283 | 1,168609 | Cycle cell |
| ENSG00000138750 | PTEN | 1,534163 | 1,737146 | 2,702853 | Cycle cell |
| ENSG00000102900 | PTGER2 | -1,39065 | -1,70743 | 0,630274 | Cycle cell |
| ENSG00000137804 | PTGS2 | -2,3826 | -2,07263 | 0,15783 | Cycle cell |
| ENSG00000104904 | PTP4A1 | 0,260141 | 0,162448 | 1,785636 | Cycle cell |
| ENSG00000139579 | PTPN6 | 0,034339 | -0,08319 | 2,089326 | Cycle cell |
| ENSG00000136811 | PTPRC | -0,93829 | -1,17525 | 1,408099 | Cycle cell |
| ENSG00000060491 | PTTG1 | 0,045789 | 0,575961 | 0,57646 | Cycle cell |
| ENSG00000068308 | RAB11A | 0,386392 | 0,181603 | 0,57803 | Cycle cell |
| ENSG00000115507 | RAB11B | 0,952369 | 0,963462 | 1,502615 | Cycle cell |
| ENSG00000164329 | RACGAP1 | -0,05123 | 0,629578 | 0,573696 | Cycle cell |
| ENSG00000090060 | RAD21 | 0,668707 | 0,413374 | 1,062095 | Cycle cell |
| ENSG00000116288 | RAD50 | 0,073933 | -0,04903 | -0,49899 | Cycle cell |
| ENSG00000116288 | RAD9A | 0,311428 | 0,347806 | 0,320269 | Cycle cell |
| ENSG00000116288 | RAF1 | 1,003384 | 0,785839 | 1,551141 | Cycle cell |
| ENSG00000116288 | RALB | -1,66578 | -1,81732 | 0,319172 | Cycle cell |
| ENSG00000059378 | RAN | 1,30845 | 1,361538 | 1,097822 | Cycle cell |
| ENSG00000188677 | RANGAP1 | 0,606219 | 1,002588 | 0,633761 | Cycle cell |
| ENSG00000100982 | RASGRP4 | -1,37766 | -0,98446 | 1,289624 | Cycle cell |
| ENSG00000078674 | RASSF1 | 0,592479 | 0,36392 | 0,691969 | Cycle cell |
| ENSG00000120265 | RASSF2 | -1,77979 | -0,79242 | 2,188169 | Cycle cell |
| ENSG00000160299 | RB1 | -0,06454 | 0,664042 | 0,555391 | Cycle cell |
| ENSG00000100731 | RB1CC1 | 0,213485 | -1,0434 | 1,188518 | Cycle cell |
| ENSG00000173126 | RBBP4 | 2,244287 | 2,242883 | 2,152041 | Cycle cell |
| ENSG00000160613 | RBBP7 | 2,635025 | 2,517298 | 2,37953 | Cycle cell |
| ENSG00000141179 | RBL1 | -0,89773 | 0,080044 | -0,06357 | Cycle cell |
| ENSG00000148459 | RBM38 | 0,737707 | 1,144354 | 3,071273 | Cycle cell |
| ENSG00000104883 | RCBTB1 | 0,047187 | -0,49025 | -0,34095 | Cycle cell |
| ENSG00000121680 | RCC2 | 2,084734 | 2,075756 | 1,630769 | Cycle cell |
| ENSG00000170525 | REC8 | 0,810626 | 0,847389 | 0,889033 | Cycle cell |
| ENSG00000173889 | RFC3 | -0,18372 | 0,108084 | -0,58681 | Cycle cell |
| ENSG00000112511 | RFC4 | 0,404133 | 1,054761 | 0,423084 | Cycle cell |
| ENSG00000197724 | RFWD3 | 1,810642 | 1,669455 | 1,39378 | Cycle cell |
| ENSG00000172943 | RGS14 | -0,03185 | -0,28116 | 0,685156 | Cycle cell |
| ENSG00000044446 | RGS2 | -0,60193 | -0,21023 | 2,301952 | Cycle cell |
| ENSG00000241973 | RHOA | 3,880905 | 3,782198 | 4,566043 | Cycle cell |
| ENSG00000171608 | RIF1 | 1,002621 | 0,577981 | 0,335698 | Cycle cell |
| ENSG00000127564 | RNF130 | 1,466652 | 0,758475 | 1,436557 | Cycle cell |
| ENSG00000101445 | RPA1 | -0,19424 | 0,072554 | -0,04809 | Cycle cell |
| ENSG00000138814 | RPL24 | -4,47807 | -4,54231 | -4,09242 | Cycle cell |
| ENSG00000163421 | RPS6 | 4,170835 | 3,853483 | 3,175263 | Cycle cell |
| ENSG00000175325 | RPS6KA1 | 0,826289 | 1,362559 | 1,915614 | Cycle cell |
| ENSG00000110107 | RPS6KA3 | -0,42137 | -0,13962 | 0,18504 | Cycle cell |
| ENSG00000117360 | RPS6KB1 | -0,00109 | -0,25632 | 0,017769 | Cycle cell |
| ENSG00000112739 | RRAGA | -0,08599 | -0,3131 | 0,089701 | Cycle cell |
| ENSG00000174231 | RRM2 | -0,2397 | 0,030216 | 0,903801 | Cycle cell |
| ENSG00000174231 | RUVBL1 | 0,336223 | 0,059824 | -1,06815 | Cycle cell |
| ENSG00000174231 | RUVBL2 | 0,826664 | 0,68257 | -0,29899 | Cycle cell |
| ENSG00000174231 | SAC3D1 | 0,827697 | 0,710901 | 1,3369 | Cycle cell |
| ENSG00000172382 | SART1 | 0,371706 | 0,966803 | 0,902506 | Cycle cell |
| ENSG00000185198 | SASS6 | 0,050833 | -0,56054 | -0,72291 | Cycle cell |
| ENSG00000196415 | SAT1 | 1,611276 | 1,423865 | 3,057043 | Cycle cell |
| ENSG00000197746 | SBDS | 1,003723 | 1,198771 | 1,802158 | Cycle cell |
| ENSG00000108344 | SEC13 | 0,273425 | 0,154361 | 0,247931 | Cycle cell |
| ENSG00000188647 | SEH1L | 0,277839 | -0,14677 | -0,98123 | Cycle cell |
| ENSG00000142538 | SENP5 | 0,389629 | -0,16857 | 0,661249 | Cycle cell |
| ENSG00000104960 | SEPT11 | 0,537728 | -0,14174 | -0,39342 | Cycle cell |
| ENSG00000112245 | SEPT2 | -0,42532 | -0,44636 | -0,79925 | Cycle cell |
| ENSG00000184007 | SEPT5 | 0,403799 | 0,265366 | 0,789241 | Cycle cell |
| ENSG00000074696 | SEPT6 | 0,520251 | -0,01785 | 0,532764 | Cycle cell |
| ENSG00000188921 | SEPT7 | -0,02963 | -0,29653 | 0,469503 | Cycle cell |
| ENSG00000110536 | SEPT9 | -0,75049 | -0,69817 | 0,520612 | Cycle cell |
| ENSG00000183255 | SESN1 | -0,30641 | -1,0646 | 0,705219 | Cycle cell |
| ENSG00000163661 | SESN3 | -2,20985 | -2,04554 | 1,10962 | Cycle cell |
| ENSG00000177192 | SETX | -0,78873 | -0,33442 | 0,118763 | Cycle cell |
| ENSG00000100504 | SFI1 | -0,04125 | 0,046672 | 0,559591 | Cycle cell |
| ENSG00000169228 | SGMS1 | -1,05828 | -0,51946 | 0,903358 | Cycle cell |
| ENSG00000166128 | SHC1 | 0,745853 | 0,714629 | 1,123673 | Cycle cell |
| ENSG00000119318 | SIAH2 | 1,183588 | 1,223272 | 1,852194 | Cycle cell |
| ENSG00000160271 | SIK1 | -0,31737 | -0,43788 | 0,322453 | Cycle cell |
| ENSG00000132341 | SIPA1 | 0,012049 | 0,245423 | 1,750781 | Cycle cell |
| ENSG00000116473 | SIRT1 | -0,41654 | -0,40145 | 0,127446 | Cycle cell |
| ENSG00000132359 | SIRT2 | -0,16778 | 0,04837 | 0,494568 | Cycle cell |
| ENSG00000158987 | SIX2 | 0,58316 | 0,365995 | 0,687199 | Cycle cell |
| ENSG00000068831 | SKI | 0,934097 | 0,912557 | 1,37916 | Cycle cell |
| ENSG00000068028 | SKP1 | -0,33826 | -0,17733 | 0,809617 | Cycle cell |
| ENSG00000101265 | SKP2 | -0,16338 | 0,277476 | -0,30836 | Cycle cell |
| ENSG00000079313 | SLC3A2 | 1,115888 | 1,063204 | 1,431069 | Cycle cell |
| ENSG00000005486 | SLC9A1 | -1,24389 | -1,3217 | 0,161989 | Cycle cell |
| ENSG00000067560 | SMAD3 | 0,18712 | -0,24852 | -0,31358 | Cycle cell |
| ENSG00000168421 | SMARCB1 | 0,940201 | 0,737363 | 0,514198 | Cycle cell |
| ENSG00000164327 | SMC1A | 0,434868 | 1,02861 | 0,645721 | Cycle cell |
| ENSG00000080345 | SMC3 | 0,682151 | 0,790777 | 0,702766 | Cycle cell |
| ENSG00000167705 | SMC4 | 0,493703 | 0,938474 | 1,271807 | Cycle cell |
| ENSG00000121848 | SNCA | 1,016413 | 1,509554 | 3,699533 | Cycle cell |
| ENSG00000197067 | SOD1 | 1,296588 | 1,526291 | 0,918932 | Cycle cell |
| ENSG00000212695 | SON | 2,034219 | 1,811847 | 2,728343 | Cycle cell |
| ENSG00000223803 | SOX9 | 0,785073 | 0,250017 | 0,964454 | Cycle cell |
| ENSG00000218426 | SPAG5 | 0,010869 | 0,402717 | 0,010793 | Cycle cell |
| ENSG00000227212 | SPG21 | -0,30456 | 0,296477 | 1,525965 | Cycle cell |
| ENSG00000243779 | SPG7 | -0,16166 | -0,33328 | 0,140865 | Cycle cell |
| ENSG00000239470 | SPIN1 | 0,348445 | -0,04973 | -0,29261 | Cycle cell |
| ENSG00000132383 | SRRT | 1,145313 | 1,132043 | 1,010217 | Cycle cell |
| ENSG00000142676 | SSNA1 | 0,545442 | 0,574169 | 0,518446 | Cycle cell |
| ENSG00000236552 | SSSCA1 | 0,644399 | 0,815039 | 0,875575 | Cycle cell |
| ENSG00000063177 | STAG2 | 0,270627 | 0,349664 | 1,059537 | Cycle cell |
| ENSG00000108298 | STAT3 | 1,235879 | 1,052138 | 2,423877 | Cycle cell |
| ENSG00000114391 | STAT6 | 0,353745 | 0,361522 | 1,613582 | Cycle cell |
| ENSG00000131469 | STEAP3 | 0,33235 | 0,693943 | 0,49398 | Cycle cell |
| ENSG00000108107 | STK10 | -0,20316 | 0,009847 | 1,304592 | Cycle cell |
| ENSG00000108107 | STK11 | 0,439918 | 0,748099 | 1,675565 | Cycle cell |
| ENSG00000108107 | STK17B | 1,66504 | 1,490502 | 3,250983 | Cycle cell |
| ENSG00000156482 | STMN1 | 1,752942 | 1,187602 | 0,566115 | Cycle cell |
| ENSG00000110700 | SUPT5H | -0,06452 | -0,11165 | 0,495091 | Cycle cell |
| ENSG00000138326 | SYK | 0,24391 | -0,05709 | 1,227964 | Cycle cell |
| ENSG00000138326 | SYNE1 | -2,28421 | -1,88072 | 0,705838 | Cycle cell |
| ENSG00000196183 | TACC1 | 0,815187 | 0,986721 | 1,60053 | Cycle cell |
| ENSG00000149273 | TACC3 | -0,00652 | 0,447976 | 0,647874 | Cycle cell |
| ENSG00000178429 | TAF1 | 0,290288 | -0,03501 | 0,338649 | Cycle cell |
| ENSG00000137154 | TAF9B | 0,023905 | 0,097396 | -0,93632 | Cycle cell |
| ENSG00000108443 | TAL1 | 0,499998 | 1,030526 | 2,262306 | Cycle cell |
| ENSG00000170889 | TAOK2 | -0,09706 | -0,49007 | 0,010464 | Cycle cell |
| ENSG00000156990 | TAOK3 | -0,37664 | -0,27204 | 0,109349 | Cycle cell |
| ENSG00000167325 | TARDBP | -1,86258 | -1,53924 | -0,62128 | Cycle cell |
| ENSG00000163785 | TBRG1 | 0,368097 | 0,319043 | 0,432619 | Cycle cell |
| ENSG00000198838 | TBRG4 | 0,293127 | 0,442663 | -0,31473 | Cycle cell |
| ENSG00000180739 | TCF3 | 1,14689 | 1,249409 | 1,196327 | Cycle cell |
| ENSG00000142230 | TCF7 | -0,95762 | -1,04753 | 0,025032 | Cycle cell |
| ENSG00000105063 | TERF1 | 0,191327 | -0,38878 | -0,35518 | Cycle cell |
| ENSG00000100239 | TERF2 | 0,007937 | 0,05059 | 0,225801 | Cycle cell |
| ENSG00000175467 | TET2 | -0,06167 | -0,43841 | 0,75275 | Cycle cell |
| ENSG00000175467 | TFDP1 | 2,858615 | 3,379183 | 3,682233 | Cycle cell |
| ENSG00000175467 | TFDP2 | 0,674274 | 0,393815 | 1,764226 | Cycle cell |
| ENSG00000175467 | TGFB1 | -0,06435 | 0,069337 | 1,570909 | Cycle cell |
| ENSG00000175467 | TGFBR2 | -0,74445 | -0,60434 | 1,100291 | Cycle cell |
| ENSG00000075856 | THBS1 | -2,0025 | -1,02608 | 0,866713 | Cycle cell |
| ENSG00000212352 | TKT | 1,507446 | 1,666293 | 3,339863 | Cycle cell |
| ENSG00000099194 | TLK1 | 0,30825 | 0,318421 | 0,352889 | Cycle cell |
| ENSG00000021355 | TMEM97 | 0,463843 | 0,259779 | -0,46161 | Cycle cell |
| ENSG00000107290 | TMX1 | -0,13274 | 0,160064 | -0,0811 | Cycle cell |
| ENSG00000115524 | TNFRSF12A | 0,411636 | 0,206564 | 0,988535 | Cycle cell |
| ENSG00000116560 | TNFRSF1B | -4,41574 | -4,20394 | -3,90817 | Cycle cell |
| ENSG00000064607 | TNFSF8 | -1,64945 | -1,50688 | 0,39284 | Cycle cell |
| ENSG00000126821 | TOP1 | 1,83039 | 1,847226 | 3,656643 | Cycle cell |
| ENSG00000104969 | TOP2A | 0,154579 | 0,952675 | 0,521793 | Cycle cell |
| ENSG00000111252 | TOP2B | 0,314098 | 0,10597 | 0,459049 | Cycle cell |
| ENSG00000148341 | TP53 | 1,540879 | 1,614751 | 0,599299 | Cycle cell |
| ENSG00000107338 | TP53INP1 | 1,863642 | -0,26417 | 0,988881 | Cycle cell |
| ENSG00000187902 | TPD52L2 | 0,237037 | 0,436465 | 1,190741 | Cycle cell |
| ENSG00000108061 | TPP1 | 0,139398 | 0,764877 | 1,560782 | Cycle cell |
| ENSG00000149577 | TPR | 0,333972 | 0,100931 | -0,06355 | Cycle cell |
| ENSG00000160584 | TPX2 | 0,145378 | 0,872411 | 0,583284 | Cycle cell |
| ENSG00000004809 | TRNP1 | 0,37849 | 0,115844 | 0,542243 | Cycle cell |
| ENSG00000155287 | TRRAP | 0,381798 | 0,182706 | 0,459796 | Cycle cell |
| ENSG00000075415 | TSC1 | -0,86913 | -0,52824 | 0,56489 | Cycle cell |
| ENSG00000075415 | TSC2 | 0,363093 | 0,372683 | 0,697923 | Cycle cell |
| ENSG00000181035 | TSPO | 1,48347 | 1,803591 | 3,162573 | Cycle cell |
| ENSG00000181856 | TTC19 | 0,035399 | -0,79859 | -0,43316 | Cycle cell |
| ENSG00000137700 | TUBA1A | -0,27724 | -0,35483 | 0,774084 | Cycle cell |
| ENSG00000111371 | TUBA1B | 2,774675 | 2,923088 | 2,903473 | Cycle cell |
| ENSG00000157637 | TUBA1C | 1,121784 | 1,605301 | 1,23729 | Cycle cell |
| ENSG00000134294 | TUBB | 4,167375 | 4,484843 | 4,386993 | Cycle cell |
| ENSG00000017483 | TUBB2A | 0,969177 | 0,832237 | 0,841476 | Cycle cell |
| ENSG00000141873 | TUBB2C | 3,176345 | 3,709963 | 3,923293 | Cycle cell |
| ENSG00000029364 | TUBG1 | 2,308565 | 3,134313 | 2,066789 | Cycle cell |
| ENSG00000168003 | TUBGCP3 | -0,22302 | 0,147072 | 0,191733 | Cycle cell |
| ENSG00000138449 | TUBGCP4 | -0,51009 | 0,123776 | -0,62109 | Cycle cell |
| ENSG00000133065 | TUBGCP6 | -0,10813 | 0,119281 | 0,456482 | Cycle cell |
| ENSG00000134802 | TUSC2 | -0,38988 | -0,39045 | 0,09412 | Cycle cell |
| ENSG00000004939 | TXLNA | 0,082372 | -0,2556 | -0,2571 | Cycle cell |
| ENSG00000164889 | TXN | -0,06932 | 0,387357 | -0,01184 | Cycle cell |
| ENSG00000130821 | TXNIP | 3,471265 | 3,051753 | 3,753223 | Cycle cell |
| ENSG00000139514 | TXNL4A | 0,151989 | 0,38374 | 0,006207 | Cycle cell |
| ENSG00000197818 | TYMS | 2,221712 | 2,384658 | 1,917101 | Cycle cell |
| ENSG00000099956 | UBA1 | 1,925578 | 1,475205 | 1,972884 | Cycle cell |
| ENSG00000139613 | UBA52 | 2,730905 | 2,673263 | 2,979033 | Cycle cell |
| ENSG00000198887 | UBB | 4,588115 | 4,657338 | 5,213323 | Cycle cell |
| ENSG00000196787 | UBC | 5,120515 | 4,831483 | 5,873363 | Cycle cell |
| ENSG00000100796 | UBE2C | 0,211213 | 0,314993 | 0,465387 | Cycle cell |
| ENSG00000198952 | UBE2E1 | 0,731349 | 0,319062 | 0,50982 | Cycle cell |
| ENSG00000116698 | UBE2E3 | 0,526585 | 0,110804 | 0,273961 | Cycle cell |
| ENSG00000119953 | UBE2I | 0,084686 | 0,163065 | 0,436247 | Cycle cell |
| ENSG00000200394 | UBQLN2 | 0,292708 | 0,138638 | 0,470697 | Cycle cell |
| ENSG00000207475 | UBR5 | -0,20451 | -0,49092 | 0,340283 | Cycle cell |
| ENSG00000207174 | UHMK1 | -0,27106 | 0,030824 | 0,330397 | Cycle cell |
| ENSG00000209042 | UHRF1 | 1,40165 | 1,391586 | 1,514453 | Cycle cell |
| ENSG00000202252 | UHRF2 | 0,710276 | 0,485159 | 0,579846 | Cycle cell |
| ENSG00000207445 | UIMC1 | -0,53609 | -0,67196 | 0,427801 | Cycle cell |
| ENSG00000238311 | UPF1 | 0,807637 | 0,808779 | 1,174599 | Cycle cell |
| ENSG00000144028 | USP22 | -0,27465 | -1,27138 | -1,22546 | Cycle cell |
| ENSG00000144028 | USP3 | 0,654207 | 0,389072 | 1,47259 | Cycle cell |
| ENSG00000115234 | USP9X | 0,497592 | 0,343836 | 1,70628 | Cycle cell |
| ENSG00000112335 | VAPA | 0,482728 | 0,064336 | 0,915272 | Cycle cell |
| ENSG00000159140 | VCP | 1,44419 | 1,6991 | 1,847994 | Cycle cell |
| ENSG00000140263 | VCPIP1 | 0,296116 | -0,07389 | 0,772308 | Cycle cell |
| ENSG00000105866 | VPS24 | -0,25663 | -0,0259 | 0,39173 | Cycle cell |
| ENSG00000114902 | VPS4A | 0,404891 | 0,37212 | 0,211837 | Cycle cell |
| ENSG00000129128 | VPS4B | -0,77544 | -0,64368 | 0,069826 | Cycle cell |
| ENSG00000066336 | VRK1 | -0,4259 | 0,288776 | 0,66505 | Cycle cell |
| ENSG00000162032 | WAC | 0,904623 | 0,973415 | 1,903193 | Cycle cell |
| ENSG00000163554 | WAPAL | -0,15014 | 0,169955 | 0,573048 | Cycle cell |
| ENSG00000222368 | WDR6 | 0,478995 | 0,801006 | 1,090583 | Cycle cell |
| ENSG00000174780 | WHSC1L1 | -0,07433 | 0,212964 | 0,782505 | Cycle cell |
| ENSG00000100104 | WISP2 | 0,080198 | -0,35701 | 0,167633 | Cycle cell |
| ENSG00000087087 | WNT10B | -0,28551 | -0,4559 | 0,059043 | Cycle cell |
| ENSG00000138385 | WRN | 0,833839 | 0,453612 | 0,861978 | Cycle cell |
| ENSG00000130511 | WTAP | 0,669471 | 0,377456 | 1,367572 | Cycle cell |
| ENSG00000180879 | XPO1 | 0,25478 | 0,333269 | -0,03788 | Cycle cell |
| ENSG00000008513 | XRCC5 | 1,24585 | 1,335415 | 1,139314 | Cycle cell |
| ENSG00000084090 | YEATS4 | 0,058309 | -0,05382 | -0,16529 | Cycle cell |
| ENSG00000168439 | YWHAE | 2,514555 | 2,513953 | 1,73775 | Cycle cell |
| ENSG00000072786 | YWHAG | 1,456389 | 1,315218 | 0,667055 | Cycle cell |
| ENSG00000115694 | YY1AP1 | 2,216196 | 1,845864 | 1,797207 | Cycle cell |
| ENSG00000131018 | ZFP36L2 | 3,339505 | 3,307623 | 3,501953 | Cycle cell |
| ENSG00000142765 | ZFYVE26 | 0,059081 | -0,12748 | 0,309734 | Cycle cell |
| ENSG00000162298 | ZFYVE27 | -0,76611 | -0,70221 | 0,031319 | Cycle cell |
| ENSG00000147133 | ZMIZ1 | -0,25775 | -0,36561 | 0,808254 | Cycle cell |
| ENSG00000137413 | ZNF259 | 0,569495 | 0,097854 | 0,44472 | Cycle cell |
| ENSG00000175463 | ZNF592 | 0,759072 | 0,893964 | 1,222605 | Cycle cell |
| ENSG00000103363 | ZWINT | 0,112844 | 0,478924 | 0,157054 | Cycle cell |
| ENSG00000200873 | ABHD5 | 0,611242 | 1,246371 | 2,404884 | Cell differentiation |
| ENSG00000240649 | ACIN1 | 0,84472 | 0,636077 | 1,132513 | Cell differentiation |
| ENSG00000123908 | ACTN4 | 2,437775 | 2,628628 | 3,074963 | Cell differentiation |
| ENSG00000239735 | ADD1 | 0,724013 | 1,137125 | 1,665387 | Cell differentiation |
| ENSG00000239935 | ADD2 | 1,916837 | 2,801753 | 1,385947 | Cell differentiation |
| ENSG00000113141 | ADM | -0,15634 | -0,51955 | 0,702664 | Cell differentiation |
| ENSG00000185294 | AGFG1 | 1,806102 | 1,894934 | 2,983283 | Cell differentiation |
| ENSG00000122971 | AGTRAP | 0,052775 | 0,451765 | 1,852723 | Cell differentiation |
| ENSG00000100412 | AIMP2 | 0,385726 | 0,541809 | -0,20368 | Cell differentiation |
| ENSG00000181786 | AKT1 | 1,744731 | 1,7493 | 1,843017 | Cell differentiation |
| ENSG00000170776 | ALAD | 1,010342 | 1,708756 | 1,68316 | Cell differentiation |
| ENSG00000105127 | ALAS1 | -0,14067 | 0,051115 | 1,252006 | Cell differentiation |
| ENSG00000011243 | ALAS2 | 0,434754 | 2,2882 | 4,423633 | Cell differentiation |
| ENSG00000244353 | AMBRA1 | 0,466556 | 0,184798 | 1,12279 | Cell differentiation |
| ENSG00000237007 | AMH | 0,430819 | -0,01114 | 0,526217 | Cell differentiation |
| ENSG00000240166 | ANAPC2 | -0,16706 | -0,2488 | 0,117943 | Cell differentiation |
| ENSG00000243482 | ANPEP | -1,11249 | -0,27443 | 1,555631 | Cell differentiation |
| ENSG00000196510 | ARF6 | 1,411891 | 1,36479 | 1,772039 | Cell differentiation |
| ENSG00000239936 | ARNT | 0,177068 | 0,836678 | 0,76152 | Cell differentiation |
| ENSG00000042753 | ARX | -0,03572 | -0,26133 | 0,083124 | Cell differentiation |
| ENSG00000100823 | ASF1B | -0,20339 | 0,216339 | 0,633563 | Cell differentiation |
| ENSG00000163382 | ASH2L | 1,199837 | 1,306302 | 1,561478 | Cell differentiation |
| ENSG00000143761 | ATP1B2 | 0,211138 | 0,88431 | 1,094578 | Cell differentiation |
| ENSG00000102931 | AZI1 | -0,10637 | -0,31777 | 0,261272 | Cell differentiation |
| ENSG00000105676 | B4GALT1 | -0,66414 | -0,77718 | 0,675326 | Cell differentiation |
| ENSG00000198356 | BCL11A | 0,008091 | 0,448514 | 0,105687 | Cell differentiation |
| ENSG00000138138 | BCL6 | -2,02711 | -1,43335 | 1,082549 | Cell differentiation |
| ENSG00000133657 | BLVRA | 0,057875 | 0,342839 | -0,08875 | Cell differentiation |
| ENSG00000163399 | BLVRB | 2,363055 | 2,750248 | 2,917293 | Cell differentiation |
| ENSG00000099624 | BPGM | -0,67015 | -0,51739 | 1,920607 | Cell differentiation |
| ENSG00000117410 | BSG | 2,478835 | 2,997613 | 4,430553 | Cell differentiation |
| ENSG00000131100 | BZW2 | 0,120605 | 0,063527 | -0,97208 | Cell differentiation |
| ENSG00000108578 | C19orf20 | 0,403424 | 0,210088 | 0,781781 | Cell differentiation |
| ENSG00000006015 | CAMK2G | -0,24589 | -0,3872 | 0,516296 | Cell differentiation |
| ENSG00000183397 | C&1 | -0,17385 | 0,109303 | -0,03581 | Cell differentiation |
| ENSG00000162490 | CAPRIN1 | 2,233888 | 1,892213 | 1,595666 | Cell differentiation |
| ENSG00000119280 | CAPRIN2 | -0,40968 | 0,102781 | -0,4884 | Cell differentiation |
| ENSG00000172247 | CAT | 2,427965 | 2,529258 | 3,650483 | Cell differentiation |
| ENSG00000100220 | CCDC85B | 0,583093 | 0,129048 | 0,401612 | Cell differentiation |
| ENSG00000100220 | CCL5 | -1,72907 | -1,78776 | 0,118568 | Cell differentiation |
| ENSG00000173950 | CD164 | 1,515959 | 1,63235 | 2,750373 | Cell differentiation |
| ENSG00000134986 | CD47 | 0,418721 | 0,599345 | 1,364788 | Cell differentiation |
| ENSG00000120306 | CD48 | -1,78578 | -1,46982 | 0,320446 | Cell differentiation |
| ENSG00000196821 | CD58 | -0,73819 | -0,48425 | 0,225967 | Cell differentiation |
| ENSG00000182307 | CDC20 | 0,69799 | 1,304721 | 0,300037 | Cell differentiation |
| ENSG00000163888 | CDK6 | 1,513185 | 1,222627 | -0,72135 | Cell differentiation |
| ENSG00000127022 | CENPF | 0,675305 | 1,061737 | 0,850759 | Cell differentiation |
| ENSG00000122565 | CIAO1 | 0,609339 | 0,498733 | 0,29948 | Cell differentiation |
| ENSG00000060339 | CIT | 0,448096 | 0,71722 | 0,536321 | Cell differentiation |
| ENSG00000176476 | CITED2 | 1,887047 | 1,831663 | 2,996803 | Cell differentiation |
| ENSG00000157456 | CLIC4 | 0,962842 | 0,63525 | 0,754271 | Cell differentiation |
| ENSG00000129315 | CLPTM1 | 1,018892 | 0,888551 | 1,306761 | Cell differentiation |
| ENSG00000177697 | CNN2 | 0,873537 | 0,635483 | 2,832943 | Cell differentiation |
| ENSG00000134690 | CPOX | 0,073352 | 0,571183 | 1,300815 | Cell differentiation |
| ENSG00000129757 | CREBBP | 0,872804 | 1,050262 | 2,530726 | Cell differentiation |
| ENSG00000183307 | CRYAA | 0,727103 | 0,200046 | 0,738935 | Cell differentiation |
| ENSG00000138092 | CSF1 | 1,419153 | 0,523943 | -0,32511 | Cell differentiation |
| ENSG00000126759 | CSRP2 | 0,326467 | 0,22007 | 0,459023 | Cell differentiation |
| ENSG00000163320 | CST3 | 2,721195 | 1,881301 | 2,009755 | Cell differentiation |
| ENSG00000173575 | CTBP1 | 1,575714 | 1,70446 | 1,794402 | Cell differentiation |
| ENSG00000110721 | CTNNB1 | 1,019751 | 0,511799 | 1,89983 | Cell differentiation |
| ENSG00000148337 | CXADR | 0,70764 | -0,22354 | -0,92405 | Cell differentiation |
| ENSG00000175505 | CYBRD1 | -0,6764 | 0,3734 | 1,040409 | Cell differentiation |
| ENSG00000179335 | DAZAP1 | 0,999567 | 0,953937 | 0,722287 | Cell differentiation |
[truncated: 22,509 more chars]
